# Intragenic homogenization and multiple copies of prey-wrapping silk genes in *Argiope* garden spiders

**DOI:** 10.1186/1471-2148-14-31

**Published:** 2014-02-20

**Authors:** R Crystal Chaw, Yonghui Zhao, Jie Wei, Nadia A Ayoub, Ryan Allen, Kirmanj Atrushi, Cheryl Y Hayashi

**Affiliations:** 1 Department of Biology, University of California, 900 University Avenue, Riverside 92507, Riverside, CA, USA; 2 Faculty of Life Science, Liaoning University, Province Shenyang, Liaoning 110036, China; 3 Department of Biology, Washington and Lee University, 204 W. Washington St., Lexington, VA 24450, USA

**Keywords:** AcSp1, Concerted evolution, Intragenic homogenization, Multiple loci, Silk, Spidroin, Spider

## Abstract

**Background:**

Spider silks are spectacular examples of phenotypic diversity arising from adaptive molecular evolution. An individual spider can produce an array of specialized silks, with the majority of constituent silk proteins encoded by members of the spidroin gene family. Spidroins are dominated by tandem repeats flanked by short, non-repetitive N- and C-terminal coding regions. The remarkable mechanical properties of spider silks have been largely attributed to the repeat sequences. However, the molecular evolutionary processes acting on spidroin terminal and repetitive regions remain unclear due to a paucity of complete gene sequences and sampling of genetic variation among individuals. To better understand spider silk evolution, we characterize a complete aciniform spidroin gene from an *Argiope* orb-weaving spider and survey aciniform gene fragments from congeneric individuals.

**Results:**

We present the complete aciniform spidroin (*AcSp1*) gene from the silver garden spider *Argiope argentata* (*Aar_AcSp1*), and document multiple *AcSp1* loci in individual genomes of *A. argentata* and the congeneric *A. trifasciata* and *A. aurantia*. We find that *Aar_AcSp1* repeats have >98% pairwise nucleotide identity. By comparing AcSp1 repeat amino acid sequences between Argiope species and with other genera, we identify regions of conservation over vast amounts of evolutionary time. Through a PCR survey of individual *A. argentata*, *A. trifasciata*, and *A. aurantia* genomes, we ascertain that *AcSp1* repeats show limited variation between species whereas terminal regions are more divergent. We also find that average dN/dS across codons in the N-terminal, repetitive, and C-terminal encoding regions indicate purifying selection that is strongest in the N-terminal region.

**Conclusions:**

Using the complete *A. argentata AcSp1* gene and spidroin genetic variation between individuals, this study clarifies some of the molecular evolutionary processes underlying the spectacular mechanical attributes of aciniform silk. It is likely that intragenic concerted evolution and functional constraints on *A. argentata AcSp1* repeats result in extreme repeat homogeneity. The maintenance of multiple *AcSp1* encoding loci in *Argiope* genomes supports the hypothesis that *Argiope* spiders require rapid and efficient protein production to support their prolific use of aciniform silk for prey-wrapping and web-decorating. In addition, multiple gene copies may represent the early stages of spidroin diversification.

## Background

Spider silks are ideal for studying the molecular evolutionary processes that create and maintain adaptive characteristics. An individual spider can produce and use different silk types singly or in combination for specific tasks, with each silk type having mechanical properties well-suited to its function. For example, aciniform silk is used in prey immobilization and egg sac construction [1,2]. The mechanical properties of aciniform silk include impressive extensibility and toughness [1], making it excellent for swathing struggling prey because it is easy to stretch but difficult to break. Orb-weaving garden spiders from the genus *Argiope* are renowned for their use of aciniform silk. *Argiope* employ many layers of aciniform silk to completely immobilize and envelop their prey (e.g. [3,4]), and *Argiope* are also a model system for studying the purpose of aciniform-silk web decorations, known as stabilimenta, that have been implicated in predator avoidance, prey attraction, and web stability (for review see [5,6]).

Of the five fibrous silks spun by the silver garden orb-weaver *Argiope argentata*, aciniform silk is the toughest and one of the most extensible [7]. However, little is known about the evolution of aciniform silk’s physical attributes. Spider silk mechanical properties are related to the suite of proteins that compose each silk type. The majority of spider silk proteins, or spidroins (a contraction of “spider-fibroins” [8]), are encoded by members of a single gene family. Spidroins are typically very large (>200 kDa), and are dominated by a series of iterated repeats flanked by short amino (N)- and carboxy (C)-terminal regions [9,10]. The length, number, and amino acid (aa) composition of the iterated repeats are silk-type specific, whereas phylogenetic analyses have shown that aa residues in the N- and C-terminal regions are more conserved across spidroins [11,12]. Repeat aa sequence corresponds to secondary structures that are partly responsible for silk mechanical properties (e.g. [13-15]), and conservation of the N- and C-terminal regions [12] and their presence in spun silk fibers suggests an important role in spider silk biology [16-18].

The evolutionary maintenance of spidroin repeat sequences within a silk type and the divergence of those repeat sequences between silk types is central to spider silk function and diversity. Within a particular spidroin, repeat units tend to be highly similar, or homogeneous, in amino acid and nucleotide sequence. The gene encoding aciniform spidroin (*AcSp1*) has repeats that are relatively complex among spidroin family members, however, despite this complexity, *AcSp1* repeats are also spectacularly homogenized [1,19]. A recent analysis of a complete AcSp1 from the western black widow *Latrodectus hesperus* showed that its repetitive region, like those of other spidroins, is dominated by the amino acids glycine (G), alanine (A), and serine (S) [19]. However, *L. hesperus* AcSp1 repeats have few or none of the short glycine and alanine-rich subunits, such as GGX, poly-GA, and poly-A, that can be the bulk of other spidroin repeats [9]. Nevertheless, *L. hesperus AcSp1* repeats are remarkably homogenized (>99% identity at the nucleotide level [19]). This is consistent with results from a partial length *AcSp1* cDNA from the banded garden orb-weaver *Argiope trifasciata*, which has 14 repeats that are each 600 bp and share 99.9% identity at the nucleotide level [1].

The high level of *AcSp1* repeat homogeneity is frequently attributed to gene conversion or unequal crossing over resulting in intragenic concerted evolution (e.g. [1,19,20]). Concerted evolution usually refers to homogenization among gene family members, such as rDNA gene copies [21], but it can also occur within a gene [22,23]. Stabilizing selection alone would maintain protein sequence, resulting in a high level of repeat identity at the aa level. However, the extreme level of homogenization reported for *AcSp1* repeats provides evidence for concerted evolution because it exists at both the protein and nucleotide levels [1,19].

In addition to concerted evolution, repeat homogeneity in AcSp1 may be maintained by functional constraints. Recent nuclear magnetic resonance (NMR) studies of AcSp1 repeats from both *Nephila antipodiana* and *A. trifasciata* delineate different domains in each repeat unit, one domain that is rich in alpha helices and one that is not [24,25]. Xu et al. [25] used NMR and dihedral angles from global likelihood estimate (DANGLE) analyses to predict the chemical shift indices of a 199 aa recombinant *A. trifasciata* AcSp1 repeat. The consensus secondary structure assignments specified that the last quarter of the protein was unstructured, but that the first three-quarters of the repeat contained six major helical regions. Protein structures such as these six alpha helices are considered the foundation for silk mechanical properties (e.g. [25,26]).

Assessing the extent to which a spidroin is homogenized within a single gene or among individuals is difficult because the repetitive region makes it exceptionally challenging to sequence complete spidroin genes. Indeed, partial length sequences that are biased toward the C-terminus greatly dominate the number of published spidroins [12]. Additionally, the evolutionary processes leading to spidroin divergence between species and silk types are often unclear due to a lack of knowledge about spidroin genetic variation among individuals.

Here, we address these issues by presenting a complete spidroin gene from an *Argiope* spider, the *AcSp1* sequence of *A. argentata* (*Aar*_*AcSp1*), and by screening for *AcSp1* variation among individual *A. argentata*, *A. trifasciata*, and *A. aurantia* spider genomes. Sequencing the full array of *Aar*_*AcSp1* repeats enabled us to test hypotheses of concerted evolution and functional constraints. Based on previous spidroin research, *Aar*_*AcSp1* repeats should be extremely homogenous at the nucleotide and amino acid levels. In addition, amino acid sequences that are predicted to correspond to the structural motifs that contribute to the toughness and extensibility of aciniform silk should be more conserved between *Argiope* species relative to surrounding regions. Among the surveyed *A. argentata* individuals, we expected *Aar*_*AcSp1* to be a single-copy gene, similar to *L. hesperus AcSp1*[19]. Between species, previous research suggests that the spidroin repeats within each silk type are highly conserved, but that the terminal regions show more variation [12], and we hypothesized that *Aar*_*AcSp1* would also follow this pattern.

## Results and discussion

### *Argiope argentata AcSp1* complete sequence and phylogenetic placement

**Figure 1:**
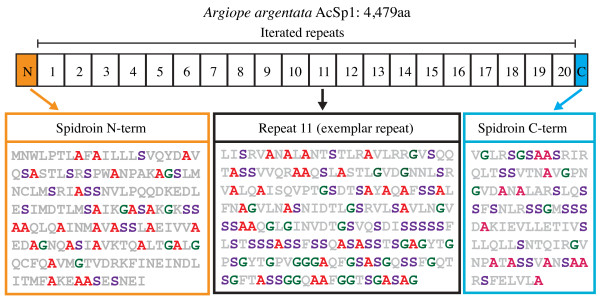
**Schematic of the protein encoded by the complete *Argiope argentata *aciniform spidroin 1 gene (GenBank KJ206620).** Predicted protein is 4,479 aa. Conserved spidroin N- (orange) and C- terminal (blue) domains (shaded boxes) flank 20 iterated repeats (numbered boxes). Boxes are drawn to scale and standardized to the 204 aa length of the first 19 repeats. Arrows point to corresponding amino acid sequences for each domain. Exemplar repeat 11 has 100% identity to the majority rule consensus of the repeat sequences. Alanine (red), serine (purple), and glycine (green) are shaded to emphasize the abundance of those amino acids.

Despite obtaining 59 *AcSp1* cDNA clones, including one that was >8 kb [1], a complete *Argiope* AcSp1 remained elusive until the present study. By screening a large-insert genomic DNA library, we sequenced and assembled 18,080 bp of *A. argentata* DNA including a complete open reading frame that is 13,440 bp long and predicted to encode a 4,479 aa *A. argentata* AcSp1 (*Aar*_AcSp1; Figure 1; GenBank KJ206620). No introns were detected. The putative protein has a predicted size of ~430 kDa, and the most abundant amino acids are serine (22.6%), alanine (14.4%) and glycine (13.3%). *Aar*_AcSp1 has three regions, a central repetitive region that is flanked by conserved N- and C-terminal regions. The repetitive region dominates ~90% of the protein and is composed of 20 iterated repeats (Figure 1). The first 19 repeats are each 204 aa, and the last repeat is 186 aa due to truncation at the end (Figure 1). The length, amino acid composition, and organization of AcSp1 are all consistent with other spidroin family members [9].

**Figure 2:**
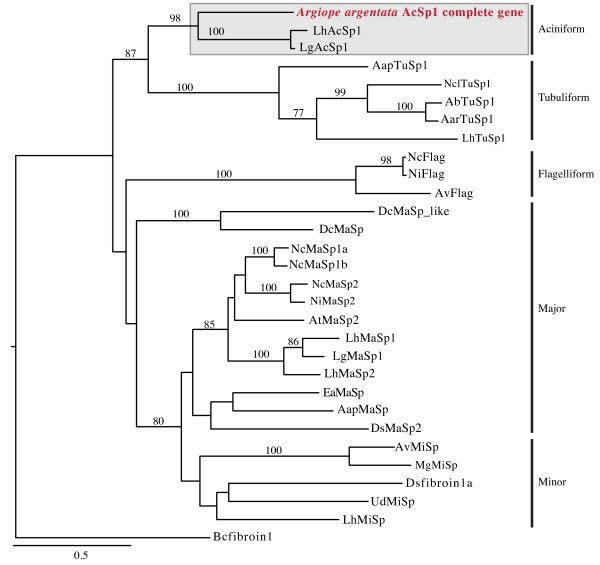
**Maximum likelihood tree of concatenated N- and C-terminal coding regions from 29 published spidroins and *Aar*_*AcSp1 *from this study (accession numbers in Additional file ****2****: Table S2).** Box highlights aciniform clade, with *Argiope argentata AcSp1* further indicated in red. Vertical bars identify clades by silk type. Bootstrap values greater than 70% are shown. Abbreviations defined in Additional file 2: Table S2. Scale bar represents substitutions per site.

Phylogenetic analyses of *Aar*_*AcSp1* N- and C-terminal coding regions with those from other spidroins grouped *Aar*_*AcSp1* with the *Latrodectus* (widow spider) *AcSp1* sequences in a well-supported clade (bootstrap value = 98%; Figure 2). *Latrodectus* and *Argiope* are estimated to have diverged from each other ~175 million years ago (MYA) [27]. Despite this lengthy time period, the recovery of an AcSp1 clade was consistent with prior studies in which spidroin sequences nearly always grouped based on silk type (e.g. [9,10]). The sister group to the AcSp1 clade was TuSp1, tubuliform (egg-case) spidroin, suggesting that these paralogs have a relatively recent common ancestor [19]. Further potential evidence of their shared ancestry is that both of these silk types are used in egg-case construction and both have repeats that are relatively complex compared to other spidroins [28]. In our phylogenetic analysis, a large, weakly supported assemblage of spidroins is sister to the combined AcSp1 and TuSp1 clade. Given the low support, it is unclear which spidroins are most closely related to AcSp1 and TuSp1.

### *Argiope argentata AcSp1* repeat homogeneity

As expected, *Aar*_AcSp1 repeats are complex and spectacularly homogenized. Although glycine, alanine, and serine account for ~50% of its repetitive region composition, *Aar*_AcSp1 has few of the glycine/alanine-rich motifs such as GGX, GPG, poly-GA, and poly-A that are dominant in the dragline major ampullate spidroins (MaSp1, MaSp2) from *Argiope* and other taxa [9]. At the nucleotide level, the average pairwise percent identity between *Aar*_*AcSp1* repeats is an astonishing 98.7%. Complexity and extreme homogenization are also features of previously described *AcSp1* sequences [1,19].

The extreme nucleotide identity of *Aar*_*AcSp1* is consistent with concerted evolution, and cannot be easily explained by codon usage bias. For example, *Aar*_*AcSp1* codon use is strongly influenced by amino acid position within a repeat. In our repeat alignment (Additional file 1: Figure S1), the neighboring alanine codons at nucleotide positions 103–105 and 106–108 are GCC and GCT, respectively. GCCGCT is present in the same relative location in all twenty repeats. Similarly, the glycine codons that appear at nucleotide positions 64–66 and 130–132 also consistently use different codons (GGT and GGA, respectively). The same alternative codons are used at the same exact positions throughout most, if not all, the repeats. Despite a slight skew toward alanine codons that end in adenine (A) or thymine (T) (55.0% GCW, W being the IUPAC ambiguity code for A or T; Additional file 2: Table S5), it is difficult to postulate that selective forces acting at the level of codon usage are responsible for the extensive homogeneity of codon positions found throughout the 612 bp *Aar*_*AcSp1* repeat. Concerted evolution that fixes particular codons at particular locations across repeats provides a clearer explanation.

**Figure 3:**
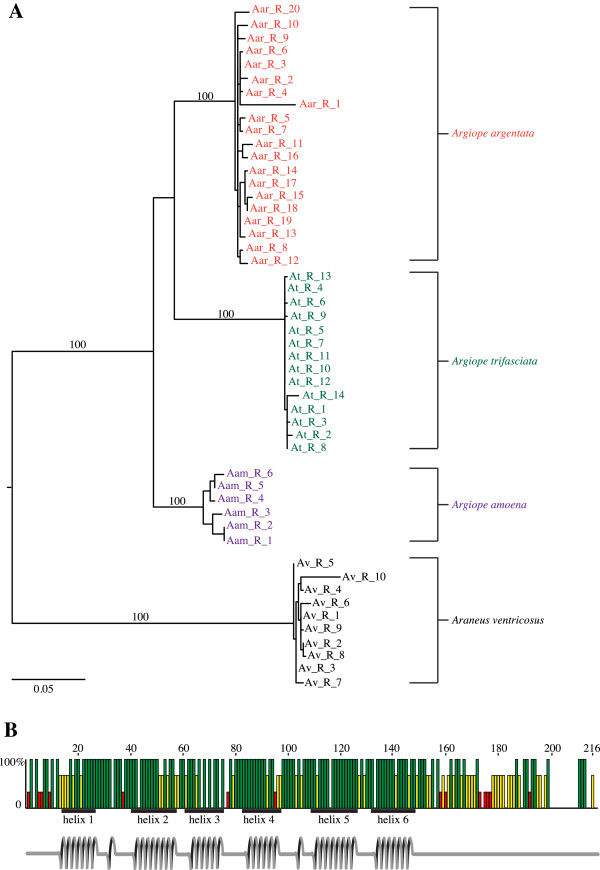
**Concerted evolution and selection on the repetitive region of *AcSp1*. (A)** Iterated *AcSp1* repeats show intra-specific homogenization in the family Araneidae. Midpoint-rooted maximum likelihood tree of *AcSp1* DNA repeats (R) from *A. amoena* (Aam; purple), *A. argentata* (Aar; orange), *A. trifasciata* (At; green), and *Araneus ventricosus* (Av; black). Repeats are numbered from 5’ to 3’. Bootstrap values for species-specific groups are shown. Scale bar indicates substitutions per site. **(B)** Functional constraint on repeat sequence. Graph of the pairwise identities of consensus AcSp1 repeat sequences from two *Argiope* species (*A. argentata* and *A. amoena*) and *Araneus ventricosus* to that of *Argiope trifasciata*. Bars show 100% (green), 66% (yellow), or 33% (red) identity at each position, helical domains found by NMR and DANGLE analyses of *A. trifasciata* AcSp1 repeat sequence shown in schematic under the graph (helix redrawn from [25]).

Analyses of the full array of *Aar*_*AcSp1* iterated repeats were also consistent with two concerted evolution predictions. First, ML analysis grouped araneid *AcSp1* repeats into well-supported, species-specific clades rather than grouping the repeats across species (Figure 3A). Furthermore, nucleotide pairwise identity within each species averaged 98%, but pairwise identity between *Aar*_*AcSp1* repeats and repeats from other species averaged only 78.5% (73.6% vs. *Araneus ventricosus*, 79.1% vs. *A. trifasciata*, and 82.8% vs. *A. amoena*). That repeats are more similar within species than between species regardless of intragenic repeat position can be explained by rapid intra-specific spread of genetic variation via unequal crossing over during recombination [22,23].

Second, the average nucleotide pairwise identity of the first and last *Aar*_*AcSp1* repeats to the rest of the array is slightly lower at 96% and 93%, respectively. Less similar first and last repeats are consistent with some models of concerted evolution [29]. However, araneid *AcSp1* first and last repeats still grouped within species-specific clades (Figure 3A), suggesting that these repeat sequences are more homogeneous within a gene than those of previously analyzed spidroins. For example, in an analysis of repetitive units from the flagelliform spidroin (*Flag*) of the golden orb-weaver *Nephila clavipes* and the congeneric *Nephila inaurata madagascariensis*, the first repeats grouped together across species, and the last repeats also formed their own clade. By contrast, the central (not first or last) repeats formed species-specific clades because each repetitive unit was nearly identical within each species yet divergent across species [30]. Longer estimated divergence times between the species in our present study may explain the more thorough homogenization of araneid *AcSp1* sequences compared to that of the previously studied *Nephila Flag* sequences. The estimated divergence time between the *Nephila* species is ~7.4 MYA [31], whereas *Araneus* and *Argiope* are thought to have diverged ~30 MYA and within *Argiope*, ~23 MYA between *A. argentata* and *A. trifasciata*[32].

### Functional constraints on *AcSp1* repeats

We predicted that functional constraints would result in greater aa sequence conservation in the portion of each repeat proposed by Xu et al. to contain six alpha helices [25]. To test this, we first compared known Araneidae repetitive regions. We aligned consensus AcSp1 repeat sequences from three *Argiope* species (*A. argentata*, *A. trifasciata*, *A. amoena*) and *Araneus ventricosus*. We then graphed pairwise identities for each aligned position between the *A. trifasciata* repeat sequence and the other species, and plotted it against the predicted *A. trifasciata* domains from Xu et al. [25] (Figure 3B). Using amino acid positions from Xu et al. [25], the average percent pairwise identity over the 150 aa helix-rich domain was 84.0%, but only 54.1% over the remaining 49 aa. Xu et al. [25] also noted a major alpha-helical domain from 102–151 aa, encompassing the region denoted as helix 5 and 6 in Figure 3B. Consistent with being structurally important, the average percent identity in this domain was 90.7%. Moreover, our alignment was slightly longer (216 aa) than the *A. trifasciata* recombinant repeat length (199 aa) due to indels that only appeared in the unstructured region. Notably, in the region from 200–209 aa (our alignment), the *A. trifasciata* repeat has a deletion (Figure 3B). These indels further indicate that the final quarter of AcSp1 repeats is less conserved than the first three-quarters.

To investigate amino acid conservation in the predicted AcSp1 helical regions across greater evolutionary time, we also aligned consensus amino acid AcSp1 repeat sequences from *L. hesperus* and *Uloborus diversus* to the *A. trifasciata* repeat from Xu et al. [19,25]. Araneidae, represented here by *Argiope* and *Araneus*, and Theridiidae, represented by *L. hesperus*, are members of the superfamily Araneoidea, with araneids and theridiids estimated to have last shared a common ancestor ~175 MYA [27]. *U. diversus* is within the Deinopoidea, the sister-group to the Araneoidea. Araneoids and deinopoids diverged from each other ~210 MYA [27]. Together, Araneoidea and Deinopoidea compose the Orbiculariae (orb-web weaving spiders and their relatives).

The AcSp1 repeat units from *L. hesperus* and *U. diversus* are almost twice as long as the araneid AcSp1 repeat units. The *L. hesperus* and *U. diversus* repeat units can be further subdivided into two parts that align with each other [19]. We aligned each part from each species (two parts per species) to the *A. trifasciata* repeat separately. We then calculated the average pairwise percent identity for each comparison and for each of the six putative alpha-helical regions predicted by Xu et al. ([25]; Additional file 2: Table S6). The overall pairwise identities between *L. hesperus* repeat part 1 and *U. diversus* repeat part 1 with the *A. trifasciata* repeat was 30% and 29%, respectively. Of note, the percent pairwise identity between *L. hesperus* repeat part 1 and the *A. trifasciata* repeat was 47% in the *A. trifasciata* region associated with helix 4, and it was 41% against *U. diversus* repeat part 1 in the region associated with helix 6. 47% and 41% were the highest pairwise identity percentages.

Our results strongly support the hypothesis that functional constraints are acting to conserve protein sequence in the repetitive region of AcSp1. Our comparison of *A. trifasciata* AcSp1 repeat sequence with that of other araneid species indicates that a specific amino acid sequence is maintained in the predicted helix-rich domain of AcSp1 repeats across Araneidae. In contrast, comparison between the *A.trifasciata* repeat with part 1 of repeats from *L. hesperus* and *U. diversus* indicates that amino acid sequence in the regions associated with alpha-helices 4 and 6 are the most highly conserved across Orbiculariae. The higher level of conservation in the amino acid sequences corresponding to helices 4 and 6 may indicate that these regions impart the same general function across Orbiculariae whereas the other predicted helical regions of *A. trifasciata* impart functions unique to Araneidae. Sequencing *AcSp1* from other genera of Araneidae and other families of Orbiculariae will enable further elucidation of these hypotheses.

To our knowledge, there are no current predictions about the secondary structures of *L. hesperus* or *U. diversus* AcSp1 repeats. It is feasible that, like the AcSp1 domains of *N. antipodiana* and *A. trifasciata*, *L. hesperus* and *U. diversus* AcSp1 repeats also feature distinct structural regions. Finally, our analysis may be an underestimation of sequence conservation because it does not include amino acid replacements that are functionally equivalent. However, predicting functional protein similarity is difficult given the extensive physicochemical changes that spider silk undergoes as it is processed from a liquid into dry silk (e.g. [33,34]).

### Delineation of *AcSp1* variants in individual *Argiope* spiders

**Figure 4:**
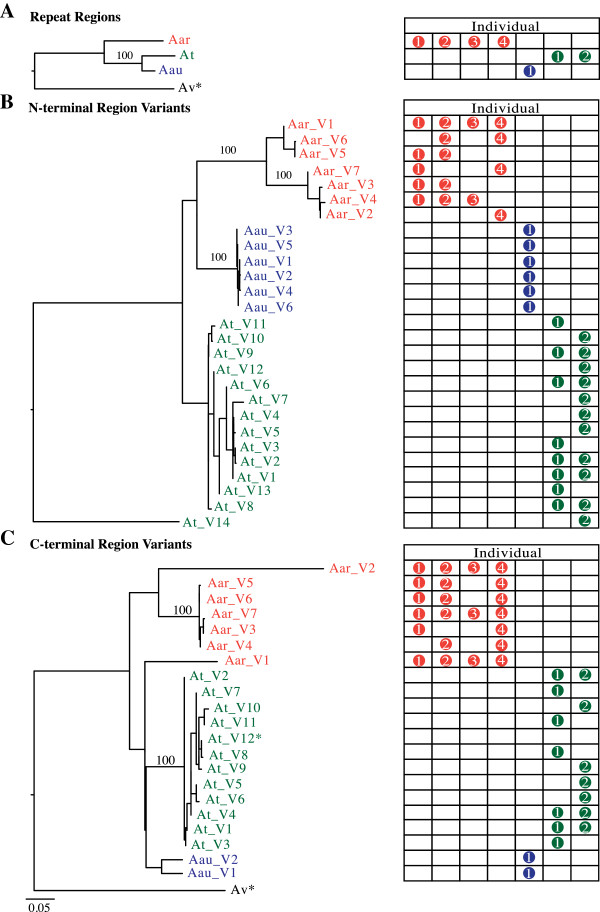
**Multiple *AcSp1 *loci in *Argiope*.** Maximum likelihood nucleotide trees of PCR amplified sequences from the **(A)** repetitive, **(B)** N-terminal, and **(C)** C-terminal coding regions of *AcSp1* from three *Argiope* species, *A. argentata* (Aarg; orange), *A. aurantia* (Aau; blue), and *A. trifasciata* (At; green). *Araneus ventricosus* (Av; black) sequence (GenBank HQ008714) was used to root repeat and C-terminal trees. N-terminal tree is midpoint-rooted **(B)**. For each variant, the adjacent table row indicates the status of that variant in individual spiders. Each individual per species was assigned a number that appears in the corresponding row if a variant was detected. Bootstrap values of 100% are shown. * denotes outgroup or published *A. trifasciata* sequence (GenBank AY426339), scale bar represents substitutions per site. Accession numbers for sequence generated in this study are given in Additional file 2: Table S4.

Spidroin sequence variation between individual spiders is an important source of genetic variation for the evolution of different silk types within and between species. To investigate genetic variation in *AcSp1* between individuals of *A. argentata* and the congeneric *A. trifasciata* and *A. aurantia*, we first designed PCR primers targeting the repetitive region of *Aar*_*AcSp1*. Amplification of genomic DNA across species and individuals resulted in *AcSp1* repeat sequences that did not show intraspecific variation but had significant inter-specific variation (Figure 4A). Intraspecific homogenization of the repeats could be explained by biased PCR amplification of a single repeat type in the repetitive region, however, our results are consistent with the high degree of repeat sequence conservation in *AcSp1* sequences from araneids (Figure 3A) and *L. hesperus*[19].

Next, we designed PCR primers targeting N- and C-terminal coding regions of *Aar*_*AcSp1*. We then amplified the same individual genomic DNAs that were surveyed for the repetitive region (Figure 4). Unlike the repetitive region PCR, direct sequencing of terminal region PCR products resulted in extensive numbers of multiple peaks and in some cases, poor sequencing reads due to length differences. Thus, all terminal region PCR products were cloned, a total of 385 amplicons were sequenced, and *AcSp1* variants were diagnosed. Each variant was supported by at least two amplicons with sequences that had greater than 95% identical bases (Figure 4; see Methods). Unlike the repetitive region sequences, which showed no intra-specific or allelic variation, all terminal region amplifications were heterogeneous.

The number of variants characterized was surprising because all of the individual spiders surveyed were found to have more than two terminal region variants, indicating that these *Argiope* species must have multiple AcSp1 encoding loci. *Argiope* spiders are not known to be polyploid, thus multiple gene copies per genome is the only explanation for more than two N- or C- terminal region variants in a single individual. For example, we found seven C-terminal variants in one *A. argentata*, suggesting at least four AcSp1 gene copies (Figure 4C). Each *A. trifasciata* individual possessed a minimum of seven N- or C-terminal variants, again indicating at least four gene copies (Figure 4B, C). Likewise, an *A. aurantia* individual possessed six N-terminal variants but only two C-terminal variants (Figure 4B, C). The smaller number of C-terminal variants could be explained by lack of variation in the C-terminal region or by incomplete sampling of variants by PCR survey.

ML analysis of sequences from the PCR survey shows that the branch lengths in the repetitive region (Figure 4A) are shorter than the branch lengths of the terminal region trees (Figure 4B, C). The majority of N- and C-terminal variants cluster into well-supported, species-specific groups, and intra-specific branch lengths are very short compared to inter-specific branch lengths (Figure 4B, C). One exception is *A. argentata* C-terminal coding region variant V1, which forms a weakly supported group with *A. trifasciata* and *A. aurantia* C-terminal coding region variants (Figure 4C). Given the weak clade support, this variant is probably an outlier that is not as homogenized as the other *A. argentata* variants.

The shorter branch lengths of the repetitive region variants tree compared to those of the N- and C-terminal region trees suggest that the repetitive region is the most conserved araneid AcSp1 region (Figure 4). Yet, comparison of the average ratio of non-synonymous to synonymous substitution rates (dN/dS) across codons implies that the N-terminal region has been subject to slightly stronger purifying selection than the repetitive and C-terminal regions (0.20 vs. 0.30 and 0.42 dN/dS, respectively). dN/dS estimations, however, assume independence of sites and thus are confounded by factors such as concerted evolution and recombination. The full-length *Aar*_*AcSp1* and other *AcSp1* provide extensive evidence that the repetitive region units are most likely not evolving independently from each other (Figure 3A; [1,19]). Thus concerted evolution and purifying selection both must play a role in the near-perfect homogeneity of *Argiope AcSp1* iterated repeats. Recombination can also affect tests of selection [35]. Because we could not conclusively determine the exact number of loci within an individual or assign alleles to specific loci, we were unable to ascertain recombination between loci. Subsequent analyses with additional data could address the impact of recombination on dN/dS estimates.

Previous work with *AcSp1* sequences did not find evidence for multiple loci [1,19]. The lack of variation among *A. trifasciata AcSp1* cDNA clones [1] may be due to overexpression or preferential cloning of one variant and thus its preponderance in the characterized cDNAs. Alternatively, consistent depletion of aciniform silk may be required to stimulate transcription of multiple *AcSp1* loci. This hypothesis is supported by a significant increase in aciniform-silk dependent web-decorating behavior in three species of *Argiope* in response to a two-week period of aciniform silk depletion [4]. Future work could focus on comparing the number of *AcSp1* variants expressed by spiders consistently depleted of aciniform silk versus that from spiders that are not depleted.

Survey of individual *L. hesperus* genomes also did not find AcSp1 variants [19]. However, the detection of multiple *AcSp1* loci in *Argiope* but not *Latrodectus* is consistent with the hypothesis that *Argiope* spiders maintain multiple gene copies as a strategy for efficiently producing large amounts of protein. In contrast with *Argiope* spiders, *Latrodectus* spiders use markedly fewer strands of aciniform silk during prey capture [3] and do not make stabilimenta. Increased *AcSp1* copy number in *Argiope* spiders may therefore be a strategy for increasing protein production [36]. Because spidroins are costly, highly expressed proteins [37,38], resource abundance in the form of prey availability may also stimulate aciniform spidroin production in *Argiope* to prepare for resource scarcity [39,40]. Precedent for this strategy exists. In the bacteria *Escherichia coli*, multiple copies of rRNA operons provide a competitive advantage by enabling increased growth rates and decreased cell division lag time in environments where resources fluctuate rapidly [41,42].

Previous research has found variants for other spidroins [43-46], and that the dragline spidroin MaSp1 is encoded by multiple loci in several species [47,48]. Unlike *A. argentata AcSp1* variants, the C-terminal coding region of *L. hesperus MaSp1* is nearly identical across loci [47]. This difference could indicate functional constraints on the C-terminal coding region of *MaSp1* that either differ from or are not acting on *AcSp1*. A comparison of structural component predictions from the amino acid sequences of terminal regions across different spidroins would greatly inform our understanding of the contribution of the terminal regions to the evolution of different spider silk types.

## Conclusions

The highly similar iterated repeat array of our complete *Argiope argentata AcSp1* gene combined with sequence conservation of functionally important regions of individual repeats supports a hypothesis of concerted evolution and functional constraints acting together to homogenize *Aar*_*AcSp1* repeats. In addition, several terminal region variants per individual *Argiope* genome indicate multiple *Argiope AcSp1* loci. Across *AcSp1* loci within an individual, we found homogenization of the repetitive region, but variation at the terminal coding regions. We also found evidence for stronger purifying selection in the N-terminal region versus the repetitive or C-terminal region, suggesting that the N-terminal region is the most constrained portion of the aciniform spidroin. The maintenance of multiple copies of *AcSp1* in *Argiope* genomes underscores the importance of aciniform silk in *Argiope* ecology and evolution. Indeed, variation between individuals and multiple gene copies within individuals could provide a method for the rapid synthesis of aciniform silk in this genus, and may represent the early stages of the differentiation that led to the extraordinary sequence and functional diversity of spider silks.

## Methods

### Isolation and sequencing of *AcSp1* containing BAC clone

A bacterial artificial chromosome (BAC) library was constructed by Rx BioSciences (Rockville, MD) with *Argiope argentata* genomic DNA inserted into pCC1BAC vector (Epicentre, Madison, WI). Colony pools were PCR screened for *AcSp1* with primers designed from the repetitive region of *Argiope trifasciata AcSp1* (Additional file 2: Table S1), resulting in one positive clone. The positive clone was restriction enzyme digested and a ~17 kb Hind III fragment of the full insert was found to contain the complete *AcSp1* gene.

The 17 kb fragment was gel purified with the S.N.A.P. UV-Free Gel Purification Kit (Invitrogen, Carlsbad, CA), ligated into HindIII digested pZErO™-2 plasmid (Invitrogen), and transformed into TOP10 cells (Invitrogen). Seven plasmid clones with the expected insert size and restriction enzyme digest patterns were end-sequenced with M13 and Sp6 primers to identify orientation of the inserts. Two clones (one of each insert orientation) were triple-digested with SpeI, XbaI, and XhoI and the fragments were gel-purified. The two clones were also single-digested with BamHI and the largest fragment from each digest (5.5 kb or 5.9 kb, composed of the vector and either a 2.2 kb or 2.6 kb insert fragment) was gel-purified and re-circularized to produce subclones. The triple digest produced a 12.4 kb SpeI/XbaI fragment that was gel-purified and subcloned into SpeI digested pZErO-2 plasmid. End-sequencing the subclones revealed that the 2.6, 12.4, and 2.2 kb inserts corresponded to the AcSp1 N-terminal, repetitive, and C-terminal encoding regions, respectively. The 2.6 and 2.2 kb fragments were sequenced in their entirety using primer walking (Additional file 2: Table S1). Because the 12.4 kb fragment contained repetitive nucleotide sequence, primer walking was not feasible. Instead, the 12.4 kb fragment was bidirectionally sequenced in its entirety using the transposon-based EZ-Tn5 < TET-1 > Insertion Kit (Epicentre Biotechnologies). The complete contig of the 17 kb genomic fragment was manually assembled with Sequencher 4.5 (Gene Codes, Ann Arbor, MI). An additional 1 kb of genomic sequence immediately adjacent to the 3’ end of the 17 kb fragment was determined by primer walking (Additional file 2: Table S1) using the original BAC clone as template DNA. The complete *Argiope argentata* AcSp1 gene was uploaded to GenBank with the accession number KJ206620.

### Inter- and Intraspecific sampling of N-, repetitive, and C-terminal coding region fragments

Genomic DNA was extracted from single legs removed from four *A. argentata*, one *A. aurantia*, and two *A. trifasciata* individuals using the DNeasy Blood & Tissue Kit (Qiagen, Valencia, CA). N-terminal, repetitive, and C-terminal encoding fragments of *AcSp1* were PCR amplified using primers designed from the *A. argentata AcSp1* complete gene (Additional file 2: Table S1).

PCR products of the expected size were purified using the AccuPrep Gel Purification Kit (Bioneer Inc., Alameda, CA). Products were directly sequenced. If a chromatograph had overlapping peaks, indicative of heterogeneous amplification, then the product was ligated into pJET 1.2 plasmid (ThermoScientific) and transformed into TOP10 cells. Individual colonies were PCR amplified using pJET1.2 Forward and Reverse sequencing primers. Inserts of the expected size were gel purified and sequenced. If one variant was highly abundant, then additional colonies were PCR amplified and digested with restriction enzymes to identify the abundant variants. The remaining undigested PCR products containing the rare variant were purified and sequenced.

### Diagnosing variants

Nucleotide sequences from the PCR fragments from each species were aligned as described below. For variant diagnosis, single nucleotide polymorphisms (SNPs) that were present in only one individual clone were attributed to *Taq* polymerase error and that SNP was ignored. If a sequence had a pattern of polymorphism that was not present in at least one other clone, the sequence was discarded. Neighbor joining trees were then used to visualize highly similar sequences. Clusters that had greater than 95% identical sites were considered a variant group. With the exception of the cluster for *A. trifasciata* C-terminal coding variant 14 (95.2% identical sites), all clusters had greater than 98% identical bases. Clustered sequences were extracted and aligned to derive the majority rule consensus for that variant. Each variant is therefore supported by at least two sequences. Variants were uploaded to GenBank with accession numbers KJ206570–KJ206619.

### Phylogenetic analyses

The conserved spidroin N- and C-terminal regions from the complete *A. argentata* AcSp1 were aligned to 29 published spidroins that also have both N- and C-terminal regions (accession numbers in Additional file 2: Table S2) using ClustalW [49] implemented in Geneious v6.1.6 (Biomatters Ltd., Auckland, NZ). The N- and C-terminal regions were separately aligned with default settings and the alignments were adjusted by eye. The aa alignments dictated nucleotide alignments. N- and C-terminal encoding region alignments were concatenated for phylogenetic analyses of spidroin paralogs (Additional file 1: Figure S2). Despite potential recombination and convergence in the N- and C-terminal encoding regions, previous research found no conflict between the strongly supported nodes between separate N- and C-terminal trees and that concatenation of the terminal regions provides greater evolutionary resolution [12].

The 20 repeat units from the complete *A. argentata* AcSp1 repetitive region were divided into individual files and aligned as above with individual repeat units from published araneid *AcSp1* sequences (Additional file 1: Figure S3, accession numbers in Additional file 2: Table S3). Alignments for the N- and C-terminal encoding sequences obtained from the PCR survey of individual genomes were created as above using diagnosed variants. Repetitive region alignments from the PCR survey also used the above method (alignments in Additional file 1: Figures S4–S6).

For each nucleotide alignment, bootstrap and maximum likelihood (ML) searches for optimal trees were simultaneously conducted over 5,000 replicates using RAxML 7.2.8 with the GTRGAMMA model [50,51] through the CIPRES webserver [52]. As implemented through CIPRES, RAxML has two substitution models: GTRGAMMA and GTRCAT. GTRGAMMA is considered more thorough [50-52]. Accession numbers given in Additional file 2: Tables S2–S4.

### Selection analyses

Estimates of the number of nonsynonymous substitutions per nonsynonymous sites (dN) and synonymous substitutions per synonymous sites (dS) were produced for three *AcSp1* nucleotide alignments using MEGA5 [53]: N-terminal encoding variants (Figure 4B; Additional file 1: Figure S4), iterated repeats (Figure 3; Additional file 1: Figure S3), and C-terminal encoding variants (Figure 4C; Additional file 1: Figure S5). CodonTest [54] implemented through the Datamonkey webserver [55,56] indicated the Felsenstein 1981 (F81) [57] model of codon substitution as the best-fit for all analyzed datasets. dN/dS ratios less than, equal to, or greater than 1 were interpreted as purifying selection, neutrality, or positive selection, respectively.

### Availability of supporting data

All sequences generated in this study are deposited in GenBank (KJ206570–KJ206620). Alignments used in ML analyses are available shown in the additional files. Alignments and the corresponding trees for this study are available at TreeBASE (http://purl.org/phylo/treebase/phylows/study/TB2:S15355).

## Abbreviations

aa: amino acid; BAC: bacterial artificial chromosome; bp: base pairs; cDNA: complementary deoxyribonucleic acid; C-terminal: carboxy terminal; dN: non-synonymous substitution rate; dS: synonymous substitution rate; kb: kilobases; kDa: kilodaltons; ML: maximum likelihood; MYA: million years ago; NMR: nuclear magnetic resonance; N-terminal: amino-terminal; PCR: polymerase chain reaction; rDNA: DNA sequence that codes for ribosomal RNA; rRNA: ribosomal ribonucleic acid; SNP: single nucleotide polymorphism.

## Competing interests

The authors declare that they have no competing interests.

## Authors’ contributions

RCC collected and analyzed data and drafted the manuscript, YZ, JW, RA, and KA collected data. NA contributed to conception and design of the study. CYH conceived the study, collected data, and drafted the manuscript. All authors reviewed and approved the final manuscript.

## Supplementary Material

Additional file 1: Figure S1 Nucleotide alignment of the 20 repeat units (Aar_R) from the complete *A. argentata* AcSp1 repetitive region. Alignment position numbers shown in increments of ten. Frame 1 translation is shown under nucleotide sequences. Alignment prepared with Geneious v6.1.6 (Biomatters Ltd., Auckland, NZ). **Figure S2.** Nucleotide alignment of 30 concatenated *AcSp1* N- and C- terminal coding regions. Alignment position numbers shown in increments of ten. Frame 1 translation is shown under nucleotide sequences. Alignment positions 1–510 encompass the N-terminal coding region, 511–840 the C-terminal coding region. Alignment prepared with Geneious v6.1.6 (Biomatters Ltd., Auckland, NZ) and available on TreeBASE (http://purl.org/phylo/treebase/phylows/study/TB2:S15355). Abbreviatons and accession numbers in Additional file 2: Table S2. **Figure S3.** Nucleotide alignment of Araneidae *AcSp1* iterated repeats. Alignment position numbers shown in increments of ten. Frame 1 translation is shown under nucleotide sequences. Alignment prepared with Geneious v6.1.6 (Biomatters Ltd., Auckland, NZ) and available on TreeBASE (http://purl.org/phylo/treebase/phylows/study/TB2:S15355). Abbreviations and accession numbers in Additional file 2: Table S3. **Figure S4.** Nucleotide alignment of *AcSp1* N-terminal coding variants from PCR survey of individual *Argiope* genomes. Nucleotide alignment in FASTA format. Alignment available on TreeBASE (http://purl.org/phylo/treebase/phylows/study/TB2:S15355). Abbreviations: *A. argentata* (Aarg), *A. aurantia* (Aau), and *A. trifasciata* (At). **Figure S5.** Nucleotide alignment of *AcSp1* C-terminal coding variants from PCR survey of individual *Argiope* genomes. Nucleotide alignment in FASTA format. Alignment available on TreeBASE (http://purl.org/phylo/treebase/phylows/study/TB2:S15355). Abbreviations: *A. argentata* (Aarg), *A. aurantia* (Aau), *A. trifasciata* (At), and *Araneus ventricous* (Av). **Figure S6.** Nucleotide alignment of *AcSp1* repeat region from PCR survey of individual *Argiope* genomes. Nucleotide alignment in FASTA format. Alignment available on TreeBASE (http://purl.org/phylo/treebase/phylows/study/TB2:S15355). Abbreviations: *A. argentata* (Aarg), *A. aurantia* (Aau), *A. trifasciata* (At), and *Araneus ventricosus* (Av). Click here for file

Additional file 2: Table S1 Primers used for full-length *Aar*_*AcSp1* sequencing and targeted amplification of N-terminal, repetitive, and C-terminal coding regions. The name and sequence of primers designed for primer walking during BAC clone sequencing and primers designed to amplify N-terminal, repetitive, and C-terminal regions of *Aar*_*AcSp1* are given. **Table S2.** Accession numbers of spidroin sequences used for maximum likelihood analysis of terminal regions (Figure 2). Spidroin name is the name used in this manuscript. Species is the spider species that corresponds to the N- or C-terminal accession number. If a full length gene was used, only one accession number appears. **Table S3.** Accession numbers for sequences used in maximum likelihood analyses of iterated repeats (Figure 3). Spidroin name is the name used in this manuscript. Species is the spider species that corresponds to the N- or C-terminal accession number. **Table S4.** Accession numbers for *AcSp1* sequences generated in this study and used in maximum likelihood analyses of repeat region and N- and C-terminal encoding variants (Figure 4). GenBank abbreviations, species, and accession number are given. **Table S5.** Predicted amino acid composition and codon usage of the coding region of *Aar*_*AcSp1*. The percentage *Aar*_*AcSp1* composed of each amino acid and percentage of each codon used for each amino acid. **Table S6.** Overall and putative helical region pairwise identities of *A. trifasciata* consensus AcSp1 repeat aligned to consensus AcSp1 repeat subparts of *L. hesperus* and *U. diversus*. Consensus repeat sequences from each subpart of AcSp1 repeats from *L. hesperus* and *U. diversus* were aligned to a consensus repeat from *A. trifasciata*. Overall pairwise percent identity and percent identity shown for each of six helical regions as predicted by Xu et al [32]. Click here for file

## References

[B1] Hayashi CY Blackledge TA Lewis RV Molecular and mechanical characterization of aciniform silk: uniformity of iterated sequence modules in a novel member of the spider silk fibroin gene family Mol Biol Evol 2004 21 1950 1959 10.1093/molbev/msh204 15240839

[B2] Vasanthavada K Hu X Falick A Mattina CL Moore A Jones P Yee R Reza R Tuton T Vierra C Aciniform spidroin, a constituent of egg case sacs and wrapping silk fibers from the black widow spider *Latrodectus hesperus* J Biol Chem 2007 282 35088 35097 10.1074/jbc.M705791200 17921147

[B3] Griswold CE Coddington JA Hormiga G Scharff N Phylogeny of the orb-web building spiders (Araneae, Orbiculariae: Deinopoidea, Araneoidea) Zool J Linn Soc-Lond 1998 123 1 99 10.1111/j.1096-3642.1998.tb01290.x

[B4] Walter A Elgar MA Bliss P Moritz RFA Wrap attack activates web-decorating behavior in *Argiope* spiders Behav Ecol 2008 19 799 804 10.1093/beheco/arn030

[B5] Walter A Elgar MA The evolution of novel animal signals: silk decorations as a model system Biol Rev 2012 87 686 700 10.1111/j.1469-185X.2012.00219.x 22309051

[B6] Herberstein ME Craig CL Coddington JA Elgar MA The function significance of silk decorations of orb-web spiders: a critical review of the empirical evidence Biol Rev Camb Philos Soc 2000 75 649 669 11117202 10.1111/j.1469-185x.2000.tb00056.x

[B7] Blackledge TA Hayashi CY Silken toolkits: biomechanics of silk fibers spun by the orb web spider *Argiope argentata* (Fabricius 1775) J Exp Biol 2006 209 2452 2461 10.1242/jeb.02275 16788028

[B8] Hinman MB Lewis RV Isolation of a clone encoding a second dragline silk fibroin. *Nephila clavipes* dragline silk is a two-protein fiber J Biol Chem 1992 267 19320 19324 1527052

[B9] Gatesy J Hayashi C Motriuk D Woods J Lewis R Extreme diversity, conservation, and convergence of spider silk fibroin sequences Science 2001 291 2603 2605 10.1126/science.1057561 11283372

[B10] Garb JE DiMauro T Lewis RV Hayashi CY Expansion and intragenic homogenization of spider silk genes since the Triassic: evidence from Mygalomorphae (tarantulas and their kin) spidroins Mol Biol Evol 2007 24 2454 2464 10.1093/molbev/msm179 17728281

[B11] Rising A Hjälm G Engström W Johansson J N-terminal nonrepetitive domain common to dragline, flagelliform, and cylindriform spider silk proteins Biomacromolecules 2006 7 3120 3124 10.1021/bm060693x 17096540

[B12] Garb JE Ayoub NA Hayashi CY Untangling spider silk evolution with spidroin terminal domains BMC Evol Biol 2010 10 243 259 10.1186/1471-2148-10-243 20696068 PMC2928236

[B13] Xu M Lewis RV Structure of a protein superfiber: spider dragline silk Proc Natl Acad Sci U S A 1990 87 7120 7124 10.1073/pnas.87.18.7120 2402494 PMC54695

[B14] Jenkins JE Creager MS Butler EB Lewis RV Yarger JL Holland GP Solid-state NMR evidence for elastin-like beta-turn structure in spider dragline silk Chem Commun (Camb) 2010 46 6714 6716 10.1039/c0cc00829j 20733981

[B15] Xu L Tremblay M-L Orrell KE Leclerc J Meng Q Liu X-Q Rainey JK Nanoparticle self-assembly by a highly stable recombinant spider wrapping silk protein subunit FEBS Lett 2013 587 3273 3280 10.1016/j.febslet.2013.08.024 23994530

[B16] Beckwitt R Arcidiacono S Sequence conservation in the C-terminal region of spider silk proteins (Spidroin) from *Nephila clavipes* (Tetragnathidae) and *Araneus bicentenarius* (Araneidae) J Biol Chem 1994 269 6661 6663 8120021

[B17] Sponner A Unger E Grosse F Weisshart K Conserved C-termini of spidroins are secreted by the major ampullate glands and retained in the silk thread Biomacromolecules 2004 5 840 845 10.1021/bm034378b 15132670

[B18] Hu X Kohler K Falick AM Moore AMF Jones PR Vierra C Spider egg case core fibers: trimeric complexes assembled from TuSp1, ECP-1, and ECP-2 Biochemistry 2006 45 3506 3516 10.1021/bi052105q 16533031

[B19] Ayoub NA Garb JE Kuelbs A Hayashi CY Ancient properties of spider silks revealed by the complete gene sequence of the prey-wrapping silk protein (AcSp1) Mol Biol Evol 2012 30 589 601 23155003 10.1093/molbev/mss254 PMC3563967

[B20] Beckwitt R Arcidiacono S Stote R Evolution of repetitive proteins: spider silks from *Nephila clavipes* (Tetragnathidae) and *Araneus bicentenarius* (Araneidae) Insect Biochem Mol Biol 1998 28 121 130 10.1016/S0965-1748(97)00083-0 9654736

[B21] Ganley ARD Kobayashi T Highly efficient concerted evolution in the ribosomal DNA repeats: Total rDNA repeat variation revealed by whole-genome shotgun sequence data Genome Res 2007 17 184 191 10.1101/gr.5457707 17200233 PMC1781350

[B22] Swanson WJ Vacquier VD Concerted evolution in an egg receptor for a rapidly evolving abalone sperm protein Science 1998 281 710 712 9685267 10.1126/science.281.5377.710

[B23] Hayashi CY Lewis RV Molecular architecture and evolution of a modular spider silk protein gene Science 2000 287 1477 1479 10.1126/science.287.5457.1477 10688794

[B24] Wang S Huang W Yang D NMR structure note: repetitive domain of aciniform spidroin 1 from *Nephila antipodiana* J Biomol NMR 2012 54 415 420 10.1007/s10858-012-9679-5 23129012

[B25] Xu L Tremblay ML Meng Q Liu XQ 1H, 13C and 15N NMR assignments of the aciniform spidroin (AcSp1) repetitive domain of *Argiope trifasciata* wrapping silk Biomol NMR Assigm 2012 6 147 151 10.1007/s12104-011-9344-z 21989955

[B26] Hayashi CY Lewis RV Evidence from flagelliform silk cDNA for the structural basis of elasticity and modular nature of spider silks J Mol Biol 1998 275 773 784 10.1006/jmbi.1997.1478 9480768

[B27] Ayoub NA Hayashi CY Hedges SB, Kumar S Spiders (Araneae) The Timetree of Life 2009 Oxford University Press 255 259

[B28] Tian M Lewis RV Molecular characterization and evolutionary study of spider Tubuliform (eggcase) silk protein Biochemistry 2005 44 8006 8012 10.1021/bi050366u 15924419

[B29] McAllister BF Werren JH Evolution of tandemly repeated sequences: what happens at the end of an array? J Mol Evol 1999 48 469 481 10.1007/PL00006491 10079285

[B30] Hayashi CY Lewis RV Spider flagelliform silk: lessons in protein design, gene structure, and molecular evolution Bioessays 2001 23 750 756 10.1002/bies.1105 11494324

[B31] Su YC Chang YH Smith D Zhu MS Kuntner M Tso IM Biogeography and speciation patterns of the golden orb spider genus *Nephila* (Araneae:Nephilidae) in Asia Zool Sci 2011 28 1 47 55 10.2108/zsj.28.47 21186947

[B32] Elices M Plaza GR Arnedo MA Perez-Riguerio J Torres FG Guinea GV Mechanical behavior of silk during the evolution of orb-web spinning spiders Biomacromolecules 2009 10 7 1904 1910 10.1021/bm900312c 19505138

[B33] Lefèvre T Boudreault S Cloutier C Pézolet M Diversity of molecular transformations involved in the formation of spider silks J Mol Biol 2011 405 238 253 10.1016/j.jmb.2010.10.052 21050860

[B34] Gosline JM Guerette PA Ortlepp CS Savage KN The mechanical design of spider silks: from fibroin sequence to mechanical function J Exp Biol 1999 202 3295 3303 10562512 10.1242/jeb.202.23.3295

[B35] Anisimova M Nielsen R Yang Z Effect of recombination on the accuracy of the likelihood method for detecting positive selection at amino acid sites Genetics 2002 164 1229 1236 12871927 10.1093/genetics/164.3.1229 PMC1462615

[B36] Ohno S Evolution by Gene Duplication 1970 New York: Springer-Verlag

[B37] Guehrs K-H Schlott B Grosse F Weisshart K Environmental conditions impinge on dragline silk protein composition Insect Mol Biol 2008 17 553 564 10.1111/j.1365-2583.2008.00826.x 18828841

[B38] Blamires SJ Wu C-L Tso I-M Variation in protein intake induces variation in spider silk expression PLoS ONE 2012 7 e31626 10.1371/journal.pone.0031626 22363691 PMC3282770

[B39] Tso IM The effect of food and silk reserve manipulation on decoration-building of *Argiope aetheroides* Behaviour 2004 141 5 603 616 10.1163/1568539041166690

[B40] Craig CL Wolf SG Davis JLD Hauber ME Maas JL Signal polymorphism in the web-decorating spider *Argiope argentata* is correlated with reduced survivorship and the presence of stingless bees, its primary prey Evolution 2001 55 5 986 993 10.1554/0014-3820(2001)055[0986:SPITWD]2.0.CO;2 11430658

[B41] Condon C Liveris D Squires C Schwartz I Squires CL rRNA operon multiplicity in *Escherichia coli* and the physiological implications of rrn inactivation J Bacteriol 1995 177 4152 4156 7608093 10.1128/jb.177.14.4152-4156.1995 PMC177152

[B42] Stevenson BS Schmidt TM Life history implications of rRNA gene copy number in *Escherichia coli* Appl Environ Microb 2004 70 6670 6677 10.1128/AEM.70.11.6670-6677.2004 PMC525164 15528533

[B43] Guerette PA Ginzinger DG Weber BH Gosline JM Silk properties determined by gland-specific expression of a spider fibroin gene family Science 1996 272 112 115 10.1126/science.272.5258.112 8600519

[B44] Colgin MA Lewis RV Spider minor ampullate silk proteins contain new repetitive sequences and highly conserved non-silk-like “spacer regions”. Protein Sci 1998 7 667 672 10.1002/pro.5560070315 9541398 PMC2143960

[B45] Garb JE DiMauro T Vo V Hayashi CY Silk genes support the single origin of orb webs Science 2006 312 1762 10.1126/science.1127946 16794073

[B46] Higgins LE White S Nuñez-Farfán J Vargas J Patterns of variation among distinct alleles of the Flag silk gene from *Nephila clavipes* Int J Biol Macromol 2007 40 201 216 10.1016/j.ijbiomac.2006.07.007 16982088

[B47] Ayoub NA Hayashi CY Multiple recombining loci encode MaSp1, the primary constituent of dragline silk, in widow spiders (*Latrodectus*: Theridiidae) Mol Biol Evol 2008 25 277 286 10.1093/molbev/msm246 18048404

[B48] Gaines WA IV Marcotte WR Jr Identification and characterization of multiple Spidroin 1 genes encoding major ampullate silk proteins in *Nephila clavipes* Insect Mol Biol 2008 17 465 474 10.1111/j.1365-2583.2008.00828.x 18828837 PMC2831225

[B49] Thompson JD Higgins DG Gibson TJ CLUSTAL W: improving the sensitivity of progressive multiple sequence alignment through sequence weighting, position-specific gap penalties and weight matrix choice Nucleic Acids Res 1994 22 4673 4680 10.1093/nar/22.22.4673 7984417 PMC308517

[B50] Stamatakis A RAxML-VI-HPC: maximum likelihood-based phylogenetic analyses with thousands of taxa and mixed models Bioinformatics 2006 22 2688 2690 10.1093/bioinformatics/btl446 16928733

[B51] Stamatakis A Hoover P Rougemont J A rapid bootstrap algorithm for the RAxML Web servers Syst Biol 2008 57 758 771 10.1080/10635150802429642 18853362

[B52] Miller MA Pfeiffer W Schwartz T The CIPRES science gateway Proceedings of the Gateway Computing Environments Workshop (GCE) 2010 New Orleans, LA: ACM Press 1 8

[B53] Tamura K Peterson D Peterson N Stecher G Nei M Kumar S MEGA5: molecular evolutionary genetics analysis using maximum likelihood, evolutionary distance, and maximum parsimony methods Mol Biol Evol 2011 28 10 2731 2739 10.1093/molbev/msr121 21546353 PMC3203626

[B54] Delport W Scheffler K Botha G Gravenor MB Muse SV Pond SLK CodonTest: modeling amino acid substitution preferences in coding sequences PLoS Comput Biol 2010 6 8 e1000885 10.1371/journal.pcbi.1000885 20808876 PMC2924240

[B55] Pond SLK Frost SD Datamonkey: rapid detection of selective pressure on individual sites of codon alignments Bioinformatics 2005 21 10 2531 2533 10.1093/bioinformatics/bti320 15713735

[B56] Delport W Poon AF Frost SD Pond SLK Datamonkey 2010: a suite of phylogenetic analysis tools for evolutionary biology Bioinformatics 2010 26 19 2455 2457 10.1093/bioinformatics/btq429 20671151 PMC2944195

[B57] Felsenstein J Evolutionary trees from DNA sequences: a maximum likelihood approach J Mol Evol 1981 17 368 376 10.1007/BF01734359 7288891

