# Supplementary material for: Intragenic homogenization and multiple copies of prey-wrapping silk genes in Argiope garden spiders
Source: BMC Evol Biol. 2014 Feb 20;14:31. doi: 10.1186/1471-2148-14-31 (PMC3933166; doi:10.1186/1471-2148-14-31)
Supplement: Additional file 2: Table S1 — Primers used for full-length Aar_AcSp1 sequencing and targeted amplification of N-terminal, repetitive, and C-terminal coding regions. The name and sequence of primers designed for primer walking during BAC clone sequencing and primers designed to amplify N-terminal, repetitive, and C-terminal regions of Aar_AcSp1 are given. Table S2. Accession numbers of spidroin sequences used for maximum likelihood analysis of terminal regions (Figure 2). Spidroin name is the name used in this manuscript. Species is the spider species that corresponds to the N- or C-terminal accession number. If a full length gene was used, only one accession number appears. Table S3. Accession numbers for sequences used in maximum likelihood analyses of iterated repeats (Figure 3). Spidroin name is the name used in this manuscript. Species is the spider species that corresponds to the N- or C-terminal accession number. Table S4. Accession numbers for AcSp1 sequences generated in this study and used in maximum likelihood analyses of repeat region and N- and C-terminal encoding variants (Figure 4). GenBank abbreviations, species, and accession number are given. Table S5. Predicted amino acid composition and codon usage of the coding region of Aar_AcSp1. The percentage Aar_AcSp1 composed of each amino acid and percentage of each codon used for each amino acid. Table S6. Overall and putative helical region pairwise identities of A. trifasciata consensus AcSp1 repeat aligned to consensus AcSp1 repeat subparts of L. hesperus and U. diversus. Consensus repeat sequences from each subpart of AcSp1 repeats from L. hesperus and U. diversus were aligned to a consensus repeat from A. trifasciata. Overall pairwise percent identity and percent identity shown for each of six helical regions as predicted by Xu et al [32]. [file 1471-2148-14-31-S2.doc]

**Table S1. Primers used for full-length *Aar_AcSp1* sequencing and targeted amplification of N-terminal, repetitive, and C-terminal coding regions.**

| **Primer name** | **Sequence** |
| --- | --- |
| AcSp1_1902F^a^ | GCTTTTGCGATTCTACTGCTGTC |
| AcSp1_2700R^a*^ | CGTTTGCTACTCTGGAGATAAG |
| AcSp1_Rep_F3^b^ | ATCAGCTTCTTCTACCAGCGGTGC |
| AcSp1_Rep_R3^b^ | TAATGTCACTCTGTACACTGCCGGT |
| AcSp1_Rep_F1^c^ | GCAAGCAACATTGACACATT |
| AcSp1_8635R^c^ | CATCGCTTGAAGACATGCCGGA |
| AcSp1_R499^d^ | CGAGAAACTAGATTGAAGACTACGG |
| AcSp1-Rep-R1^d^ | GAGGTCCAGAAGGTCCTGTG |
| AcSp1_18731R* | TGCATTCCGGGCCCAACTTAGA |
| AcSp1_15200F* | TGGGTGGGTGTTTCATCCTC |

^a^targets N-terminal region

^b^targets Repetitive-region

^c^targets C-terminal region

^d^specific to the C-terminal region of the full-length *Aar_AcSp1* in the BAC clone

^*^used for primer walking of BAC clone

**Table S2. Accession numbers of spidroin sequences used for maximum likelihood analysis (Fig.2**).

| Spidroin name | Species | N-term accession | C-term accession |
| --- | --- | --- | --- |
| LhAcSp1 | *Latrodectus hesperus* | JX978171 | |
| LgAcSp1 | *Latrodectus geometricus* | JX978180 | JX978181 |
| AarAcSp1 complete gene | *Argiope argentata* | KJ206620^a^ | |
| AapTuSp1 | *Agelenopsis aperta* | HM752576 | HM752572 |
| AarTuSp1 | *Argiope argentata* | HM752577 | AY953071 |
| AbTuSp1 | *Argiope bruennichi* | AB242144 | |
| NclTuSp1 | *Nephila clavata* | AB218974 | AB218973 |
| LhTuSp1 | *Latrodectus hesperus* | DQ379383 | AY953070 |
| NcFlag | *Nephila clavipes* | AF027972^b^ | AF027973 |
| NiFlag | *Nephila inaurata madagascariensis* | AF218623S1 | AF218623S2 |
| AvFlag | *Araneus ventricosus* | AY945306 | AY587193 |
| DcMaSp | *Diguetia canities* | HM752564 | HM752565 |
| DcMaSp_like | *Diguetia canities* | HM752566 | HM752567 |
| DsMaSp2 | *Deinopis spinosa* | HM752568 | DQ399328, DQ399329^c^ |
| AapMaSp | *Agelenopsis aperta* | HM752573 | AAT08436 |
| AtMaSp2 | *Argiope trifasciata* | DQ059136S1 | DQ059136S2 |
| NcMaSp2 | *Nephila clavipes* | EU599240 | AY654297 |
| NiMaSp2 | *Nephila inaurata madagascariensis* | DQ059135 | AF350278 |
| NcMaSp1a | *Nephila clavipes* | EU599238 | AY654292 |
| NcMaSp1b | *Nephila clavipes* | EU599239 | AY654291 |
| LhMaSp1 | *Latrodectus hesperus* | EF595246 | |
| LhMaSp2 | *Latrodectus hesperus* | EF595245 | |
| LgMaSp1 | *Latrodectus geometricus* | DQ059133S1^b^ | DQ059133S2 |
| EaMaSp | *Euprosthenops australis* | AM259067 | AJ973155 |
| UdMiSp | *Uloborus diversus* | HM752574 | ABD61597 |
| MgMiSp | *Metepeira grandiosa* | HM752575 | HM752569 |
| LhMiSp | *Latrodectus hesperus* | HM752570 | HM752571 |
| AvMiSp | *Araneus ventricosus* | JX513956 | |
| Dsfibroin1a | *Deinopis spinosa* | JX978170 | DQ399326 |
| Bcfibroin1 | *Bothriocyrtum californicum* | HM752562 | EU117162 |

^a^Generated in this study.

^b^DQ399329 has DsMaSp2a C-terminal sequence and DQ399328 has DsMaSp2b C-terminal sequence.

^c^DQ059133S1 and AF027972 were edited to reflect corrections from Rising et al. (2006): Rising A, Hjälm G, Engström W, Johansson J: **N-terminal nonrepetitive domain common to dragline, flagelliform, and cylindriform spider silk proteins**. *Biomacromolecules* 2006, **7**:3120-3124.

Table S3. Accession numbers for sequences used in maximum likelihood analyses of iterated repeats (Fig. 3).

| Spidroin name | Species | Accession |
| --- | --- | --- |
| AvAcSp1 | *Araneus ventricosus* | HQ008714 |
| AamAcSp1 | *Argiope amoena* | HQ008715 |
| AtAcSp1 | *Argiope trifasciata* | AY426339 |
| AarAcSp1 | *Argiope argentata* | KJ206620^a^ |

^a^Generated in this study. Accession number will be available once the manuscript is accepted for publication.

**Table S4. Accession numbers for *AcSp1* sequences generated in this study and** used in maximum likelihood analyses of repeat region and N- and C-terminal encoding variants (Fig. 4).

|  | **GenBank abbreviation** | **Species** | **Accession** |
| --- | --- | --- | --- |
| **Repeat region** | Aar_AcSp1_R | *Argiope argentata* | KJ206597 |
|  | Aau_AcSp1_R | *Argiope aurantia* | KJ206598 |
|  | At_AcSp1_R | *Argiope trifasciata* | KJ206599 |
| **N-terminal region variants** | Aar_AcSp1_N_v1 | *Argiope argentata* | KJ206570 |
|  | Aar_AcSp1_N_v2 | *Argiope argentata* | KJ206571 |
|  | Aar_AcSp1_N_v3 | *Argiope argentata* | KJ206572 |
|  | Aar_AcSp1_N_v4 | *Argiope argentata* | KJ206573 |
|  | Aar_AcSp1_N_v5 | *Argiope argentata* | KJ206574 |
|  | Aar_AcSp1_N_v6 | *Argiope argentata* | KJ206575 |
|  | Aar_AcSp1_N_v7 | *Argiope argentata* | KJ206576 |
|  | Aau_AcSp1_N_v1 | *Argiope aurantia* | KJ206577 |
|  | Aau_AcSp1_N_v2 | *Argiope aurantia* | KJ206578 |
|  | Aau_AcSp1_N_v3 | *Argiope aurantia* | KJ206579 |
|  | Aau_AcSp1_N_v4 | *Argiope aurantia* | KJ206580 |
|  | Aau_AcSp1_N_v5 | *Argiope aurantia* | KJ206581 |
|  | Aau_AcSp1_N_v6 | *Argiope aurantia* | KJ206582 |
|  | At_AcSp1_N_v1 | *Argiope trifasciata* | KJ206583 |
|  | At_AcSp1_N_v2 | *Argiope trifasciata* | KJ206584 |
|  | At_AcSp1_N_v3 | *Argiope trifasciata* | KJ206585 |
|  | At_AcSp1_N_v4 | *Argiope trifasciata* | KJ206586 |
|  | At_AcSp1_N_v5 | *Argiope trifasciata* | KJ206587 |
|  | At_AcSp1_N_v6 | *Argiope trifasciata* | KJ206588 |
|  | At_AcSp1_N_v7 | *Argiope trifasciata* | KJ206589 |
|  | At_AcSp1_N_v8 | *Argiope trifasciata* | KJ206590 |
|  | At_AcSp1_N_v9 | *Argiope trifasciata* | KJ206591 |
|  | At_AcSp1_N_v10 | *Argiope trifasciata* | KJ206592 |
|  | At_AcSp1_N_v11 | *Argiope trifasciata* | KJ206593 |
|  | At_AcSp1_N_v12 | *Argiope trifasciata* | KJ206594 |
|  | At_AcSp1_N_v13 | *Argiope trifasciata* | KJ206595 |
|  | At_AcSp1_N_v14 | *Argiope trifasciata* | KJ206596 |
| **C-terminal region variants** | Aar_AcSp1_C_v1 | *Argiope argentata* | KJ206600 |
|  | Aar_AcSp1_C_v2 | *Argiope argentata* | KJ206601 |
|  | Aar_AcSp1_C_v3 | *Argiope argentata* | KJ206602 |
|  | Aar_AcSp1_C_v4 | *Argiope argentata* | KJ206603 |
|  | Aar_AcSp1_C_v5 | *Argiope argentata* | KJ206604 |
|  | Aar_AcSp1_C_v6 | *Argiope argentata* | KJ206605 |
|  | Aar_AcSp1_C_v7 | *Argiope argentata* | KJ206606 |
|  | Aau_AcSp1_C_v1 | *Argiope aurantia* | KJ206607 |
|  | Aau_AcSp1_C_v2 | *Argiope aurantia* | KJ206608 |
|  | At_AcSp1_C_v1 | *Argiope trifasciata* | KJ206609 |
|  | At_AcSp1_C_v2 | *Argiope trifasciata* | KJ206610 |
|  | At_AcSp1_C_v3 | *Argiope trifasciata* | KJ206611 |
|  | At_AcSp1_C_v4 | *Argiope trifasciata* | KJ206612 |
|  | At_AcSp1_C_v5 | *Argiope trifasciata* | KJ206613 |
|  | At_AcSp1_C_v6 | *Argiope trifasciata* | KJ206614 |
|  | At_AcSp1_C_v7 | *Argiope trifasciata* | KJ206615 |
|  | At_AcSp1_C_v8 | *Argiope trifasciata* | KJ206616 |
|  | At_AcSp1_C_v9 | *Argiope trifasciata* | KJ206617 |
|  | At_AcSp1_C_v10 | *Argiope trifasciata* | KJ206618 |
|  | At_AcSp1_C_v11 | *Argiope trifasciata* | KJ206619 |

***Table S5. Amino acid content and codon usage of the coding region of*** Aar_AcSp1.

| AA | % of AcSp1 | Codon | % of AA |
| --- | --- | --- | --- |
| Ser | 22.6 | AGC | 14.5 |
|  |  | AGT | 19.0 |
|  |  | TCA | 15.6 |
|  |  | TCC | 18.9 |
|  |  | TCG | 4.4 |
|  |  | TCT | 27.6 |
| Ala | 14.4 | GCA | 21.3 |
|  |  | GCC | 26.0 |
|  |  | GCG | 19.0 |
|  |  | GCT | 33.7 |
| Gly | 13.3 | GGA | 43.6 |
|  |  | GGC | 26.1 |
|  |  | GGG | 4.2 |
|  |  | GGT | 26.1 |
| Leu | 7.5 | CTA | 0.6 |
|  |  | CTC | 41.5 |
|  |  | CTG | 3.6 |
|  |  | CTT | 13.6 |
|  |  | TTA | 8.6 |
|  |  | TTG | 32.0 |
| Thr | 7.4 | ACA | 40.0 |
|  |  | ACC | 36.7 |
|  |  | ACG | 1.8 |
|  |  | ACT | 21.5 |
| Val | 7.1 | GTA | 42.3 |
|  |  | GTC | 13.8 |
|  |  | GTG | 21.6 |
|  |  | GTT | 22.3 |
| Gln | 6.8 | CAA | 52.3 |
|  |  | CAG | 47.7 |
| Asn | 4.5 | AAC | 54.5 |
|  |  | AAT | 45.5 |
| Phe | 3.8 | TTC | 86.5 |
|  |  | TTT | 13.5 |
| Arg | 3.3 | AGA | 68.7 |
|  |  | AGG | 2.7 |
|  |  | CGA | 27.3 |
|  |  | CGC | 0.7 |
|  |  | CGG | 0.0 |
|  |  | CGT | 0.7 |

**Table S5 (continued). Amino acid content and codon usage of the coding region of *Aar_AcSp1.***

| AA | % of AcSp1 | Codon | % of AA |
| --- | --- | --- | --- |
| Ile | 2.7 | ATA | 8.1 |
|  |  | ATC | 52.8 |
|  |  | ATT | 39.0 |
| Asp | 2.6 | GAC | 73.0 |
|  |  | GAT | 27.0 |
| Pro | 1.6 | CCA | 31.0 |
|  |  | CCC | 32.4 |
|  |  | CCG | 2.8 |
|  |  | CCT | 33.8 |
| Tyr | 1.5 | TAC | 94.0 |
|  |  | TAT | 6.0 |
| Glu | 0.3 | GAA | 100.0 |
|  |  | GAG | 0.0 |
| Lys | 0.3 | AAA | 76.9 |
|  |  | AAG | 23.1 |
| Met | 0.2 | ATG | 100.0 |
| Cys | 0.0 | TGC | 50.0 |
|  |  | TGT | 50.0 |
| His | 0.0 | CAC | 0.0 |
|  |  | CAT | 0.0 |
| Trp | 0.0 | TGG | 100.0 |

**Table S6. Overall and putative helical region pairwise identities of *A. trifasciata* consensus AcSp1 repeat aligned to consensus AcSp1 repeat subparts of *L. hesperus* and *U. diversus*.***

| Pairwise identity vs. A. trifasciata consensus repeat (%) | | | | | | | |
| --- | --- | --- | --- | --- | --- | --- | --- |
|  | Overall | Helix 1 | Helix 2 | Helix 3 | Helix 4 | Helix 5 | Helix 6 |
| *L.he* part 1 | 30 | 38 | 24 | 35 | 47 | 28 | 29 |
| *L.he* part 2 | 30 | 29 | 35 | 33 | 32 | 28 | 35 |
| *U.di* part 1 | 29 | 23 | 18 | 27 | 32 | 22 | 41 |
| *U.di* part 2 | 19 | 31 | 24 | 11 | 16 | 28 | 12 |

^*^Consensus repeat sequences and demarcation of parts from *L. hesperus* and *U. diversus* obtained from Ayoub et al. (2012): Ayoub NA, Garb JE, Kuelbs A, Hayashi CY: **Ancient properties of spider silks revealed by the complete gene sequence of the prey-wrapping silk protein (AcSp1).** *Mol Biol Evol* 2012, **30**:589–601.
