# Supplementary material for: Intragenic homogenization and multiple copies of prey-wrapping silk genes in Argiope garden spiders
Source: BMC Evol Biol. 2014 Feb 20;14:31. doi: 10.1186/1471-2148-14-31 (PMC3933166; doi:10.1186/1471-2148-14-31)
Supplement: Additional file 1: Figure S1 — Nucleotide alignment of the 20 repeat units (Aar_R) from the complete A. argentata AcSp1 repetitive region. Alignment position numbers shown in increments of ten. Frame 1 translation is shown under nucleotide sequences. Alignment prepared with Geneious v6.1.6 (Biomatters Ltd., Auckland, NZ). Figure S2. Nucleotide alignment of 30 concatenated AcSp1 N- and C- terminal coding regions. Alignment position numbers shown in increments of ten. Frame 1 translation is shown under nucleotide sequences. Alignment positions 1–510 encompass the N-terminal coding region, 511–840 the C-terminal coding region. Alignment prepared with Geneious v6.1.6 (Biomatters Ltd., Auckland, NZ) and available on TreeBASE (http://purl.org/phylo/treebase/phylows/study/TB2:S15355). Abbreviatons and accession numbers in Additional file 2: Table S2. Figure S3. Nucleotide alignment of Araneidae AcSp1 iterated repeats. Alignment position numbers shown in increments of ten. Frame 1 translation is shown under nucleotide sequences. Alignment prepared with Geneious v6.1.6 (Biomatters Ltd., Auckland, NZ) and available on TreeBASE (http://purl.org/phylo/treebase/phylows/study/TB2:S15355). Abbreviations and accession numbers in Additional file 2: Table S3. Figure S4. Nucleotide alignment of AcSp1 N-terminal coding variants from PCR survey of individual Argiope genomes. Nucleotide alignment in FASTA format. Alignment available on TreeBASE (http://purl.org/phylo/treebase/phylows/study/TB2:S15355). Abbreviations: A. argentata (Aarg), A. aurantia (Aau), and A. trifasciata (At). Figure S5. Nucleotide alignment of AcSp1 C-terminal coding variants from PCR survey of individual Argiope genomes. Nucleotide alignment in FASTA format. Alignment available on TreeBASE (http://purl.org/phylo/treebase/phylows/study/TB2:S15355). Abbreviations: A. argentata (Aarg), A. aurantia (Aau), A. trifasciata (At), and Araneus ventricous (Av). Figure S6. Nucleotide alignment of AcSp1 repeat region from PCR survey of individual Argiope gen [file 1471-2148-14-31-S1.doc]

**Supplementary Figure S1**

**1	10	20	30	40	50	60	70**

Aar_R_1

Aar_R_2

Aar_R_3

Aar_R_4

Aar_R_5

Aar_R_6

Aar_R_7

Aar_R_8

Aar_R_9

Aar_R_10

Aar_R_11

Aar_R_12

Aar_R_13

Aar_R_14

Aar_R_15

Aar_R_16

Aar_R_17

Aar_R_18

Aar_R_19

Aar_R_20

```
CTTATCTCCAGAGTAGCAAACGCACTTTCCAATACGTCCACATTGAGGACAGTTCTCCGCAGAGGTGTGTCCCAACAG L I 	S 	R 	V 	A 	N 	A 	L S 	N 	T S 	T 	L R 	T V 	L R 	R 	G 	V 	S 	Q 	Q
CTCATCTCCAGAGTAGCAAACGCACTTGCCAATACATCCACATTGAGAGCTGTCCTCCGAAGAGGTGTATCCCAACAG L I 	S 	R 	V 	A 	N 	A 	L A 	N 	T S 	T 	L R 	A 	V 	L R 	R 	G 	V 	S 	Q 	Q
CTCATCTCCAGAGTAGCAAACGCACTTGCCAATACATCCACATTGAGAGCTGTCCTCCGAAGAGGTGTATCCCAACAG L I 	S 	R 	V 	A 	N 	A 	L A 	N 	T S 	T 	L R 	A 	V 	L R 	R 	G 	V 	S 	Q 	Q
CTCATCTCCAGAGTAGCAAACGCACTTGCCAATACATCCACATTGAGAGCTGTCCTCCGAAGAGGTGTATCCCAACAG L I 	S 	R 	V 	A 	N 	A 	L A 	N 	T S 	T 	L R 	A 	V 	L R 	R 	G 	V 	S 	Q 	Q
CTCATCTCCAGAGTAGCAAACGCACTTGCCAATACATCCACATTGAAAGCTGTTCTCCGAAGAGGTGTATCCCAACAG L I 	S 	R 	V 	A 	N 	A 	L A N 	T S 	T 	L K 	A 	V 	L R 	R 	G 	V 	S 	Q 	Q
CTCATCTCCAGAGTAGCAAACGCACTTGCCAATACATCCACATTGAGAGCTGTCCTCCGAAGAGGTGTATCCCAACAG L I 	S 	R 	V 	A 	N 	A 	L A 	N 	T S 	T 	L R 	A 	V 	L R 	R 	G 	V 	S 	Q 	Q
CTCATCTCCAGAGTAGCAAACGCACTTGCCAATACATCCACATTGAAAGCTGTCCTCCGAAGAGGTGTATCCCAACAG L I 	S 	R 	V 	A 	N 	A 	L A 	N 	T S 	T 	L K 	A 	V 	L R 	R 	G 	V 	S 	Q 	Q
CTCATCTCCAGAGTAGCAAACGCACTTGCCAATACATCCACATTGAGAGCTGTCTTCCGAAGAGGTGTATCCCAACAG L I 	S 	R 	V 	A 	N 	A 	L A 	N 	T S 	T 	L R 	A 	V 	F R 	R 	G 	V 	S 	Q 	Q
CTCATCTCCAGAGTAGCAAACGCACTTGCCAATACATCCACATTGAGAGCTGTCCTCCGAAGAGGTGTATCCCAACAG L I 	S 	R 	V 	A 	N 	A 	L A 	N 	T S 	T 	L R 	A 	V 	L R 	R 	G 	V 	S 	Q 	Q
CTCATCTCCAGAGTAGCAAACGCACTTGCCAATACATCCACATTGAGAGCTGTCCTCCGAAGAGGTGTATCCCAACAG L I 	S 	R 	V 	A 	N 	A 	L A 	N 	T S 	T 	L R 	A 	V 	L R 	R 	G 	V 	S 	Q 	Q
CTCATCTCCAGAGTAGCAAACGCACTTGCCAATACATCCACATTGAGAGCTGTCCTCCGAAGAGGTGTATCCCAACAG L I 	S 	R 	V 	A 	N 	A 	L A 	N 	T S 	T 	L R 	A 	V 	L R 	R 	G 	V 	S 	Q 	Q
CTCATCTCCGGAGTAGCAAACGCACTTGCCAATACATCCACATTGAGAGCTGTCTTCCGAAGAGGTGTATCCCAACAG L I 	S 	G 	V 	A 	N 	A 	L A 	N 	T S 	T 	L R 	A 	V 	F R 	R 	G 	V 	S 	Q 	Q
CTCATCTCCAGAGTAGCAAACGCACTTGCCAATACATCCACATTGAGAGCTGTCCTCCGAAGAGGTGTATCCCAACAG L I 	S 	R 	V 	A 	N 	A 	L A 	N 	T S 	T 	L R 	A 	V 	L R 	R 	G 	V 	S 	Q 	Q
CTCATCTCCAGAGTAGCAAACGCACTTGCCAATACATCCACATTGAGAGCTGTCCTCCGAAGAGGTGTATCCCAACAG L I 	S 	R 	V 	A 	N 	A 	L A 	N 	T S 	T 	L R 	A 	V 	L R 	R 	G 	V 	S 	Q 	Q
CTCATCTCCAGAGTAGCAAACGCACTTGCCAATACATCCACATTGAGAGCTGTCCTCCGAAGAGGTGTATCCCAACAG L I 	S 	R 	V 	A 	N 	A 	L A 	N 	T S 	T 	L R 	A 	V 	L R 	R 	G 	V 	S 	Q 	Q CTCATCTCCAGAGTAGCAAACGCACTTGCCAATACATCCACATTGAGAGCTGTCCTCCGAAGAGGTGTATCCCAACAG L I 	S 	R 	V 	A 	N 	A 	L A 	N 	T S 	T 	L R 	A 	V 	L R 	R 	G 	V 	S 	Q 	Q
CTCATCTCCAGAGTAGCAAACGCACTTGCCAATACATCCACATTGAGAGCTGTCCTCCGAAGAGGTGTATCCCAACAG L I 	S 	R 	V 	A 	N 	A 	L A 	N 	T S 	T 	L R 	A 	V 	L R 	R 	G 	V 	S 	Q 	Q
CTCATCTCCAGAGTAGCAAACGCACTTGCCAATACATCCACATTGAGAGCTGTCCTCCGAAGAGGTGTATCCCAACAG L I 	S 	R 	V 	A 	N 	A 	L A 	N 	T S 	T 	L R 	A 	V 	L R 	R 	G 	V 	S 	Q 	Q
CTCATCTCCAGAGTAGCAAACGCACTTGCCAATACATCCACATTGAGAGCTGTCCTCCGAAGAGGTGTATCCCAACAG L I 	S 	R 	V 	A 	N 	A 	L A 	N 	T S 	T 	L R 	A 	V 	L R 	R 	G 	V 	S 	Q 	Q
CTCATCTCCAGAGTAGCAAACGCACTTGCCAATACATCCACATTGAGAGCTGTCCTCCGAAGAGGTGTATCCCAACAG L I 	S 	R 	V 	A 	N 	A 	L A 	N 	T S 	T 	L R 	A 	V 	L R 	R 	G 	V 	S 	Q 	Q

TCATCTCCAGAGTAGCAAACGCACTTGCCAATACATCCACATTGAGAGCTGTCCTCCGAAGAGGTGTATCCCAACAG
```

**80	90**

**100	110	120**

**130	140	150**

Aar_R_1

Aar_R_2

Aar_R_3

Aar_R_4

Aar_R_5

Aar_R_6

Aar_R_7

Aar_R_8

Aar_R_9

Aar_R_10

Aar_R_11

Aar_R_12

Aar_R_13

Aar_R_14

Aar_R_15

```
ATTGCCTCAAGTATATTGCGAAGAGCCGCTCAGACTTTGGCCAGTACTCTCGGAGTCGACGGAAACAACTTGTCCAGA I 	A 	S 	S 	I 	L R 	R 	A 	A 	Q 	T 	L A 	S 	T 	L G 	V 	D G 	N 	N 	L S 	R
ACTGCCTCCAGCGTGGTACAGAGAGCCGCTCAGTCGTTGGCCAGTACTCTCGGAATCGACGGAAACAACTTGTCCAGA T A 	S 	S 	V 	V 	Q R 	A 	A 	Q S 	L A 	S 	T 	L G 	I 	D G 	N 	N 	L S 	R
ACTGCCTCCAGCGTGGTACAGAGAGCCGCTCAGTCGTTGGCCAGTACTCTCGGAGTCGACGGAAACAACTTGTCCAGA T A 	S 	S 	V 	V 	Q R 	A 	A 	Q S 	L A 	S 	T 	L G 	V 	D G 	N 	N 	L S 	R
ACTGCCTCCAGCGTGGTACAGAGAGCCGCTCAGTCGTTGGCCAGTACTCTCGGAGTCGACGGAAACAACTTGTCCAGA T A 	S 	S 	V 	V 	Q R 	A 	A 	Q S 	L A 	S 	T 	L G 	V 	D G 	N 	N 	L S 	R
ACTGCCTCCAGCGTGGTACAGAGAGCCGCTCAGTCGTTGGCCAGTACTCTCGGAGTCGACGGAAACAACTTGTCCAGA T A 	S 	S 	V 	V 	Q R 	A 	A 	Q S 	L A 	S 	T 	L G 	V 	D G 	N 	N 	L S 	R
ACTGCCTCCAGCGTGGTACAGAGAGCCGCTCAGTCGTTGGCCAGTACTCTCGGAGTCGACGGAAACAACTTGTCCAGA T A 	S 	S 	V 	V 	Q R 	A 	A 	Q S 	L A 	S 	T 	L G 	V 	D G 	N 	N 	L S 	R
ACTGCCTCCAGCGTGGTACAGAGAGCCGCTCAGTCGTTGGCCAGTACTCTCGGAGTCGACGGAAACAACTTGTCCAGA T A 	S 	S 	V 	V 	Q R 	A 	A 	Q S 	L A 	S 	T 	L G 	V 	D G 	N 	N 	L S 	R
ACTGCCTCCAGCGTGGTACAGAGAGCCGCTCAGTCGTTGGCCAGTACTCTCGGAGTCGACGGAAACAACTTGTCCAGA T A 	S 	S 	V 	V 	Q R 	A 	A 	Q S 	L A 	S 	T 	L G 	V 	D G 	N 	N 	L S 	R
ACTGCTTCCAGCGTGGTACAGAGAGCCGCTCAGTCGTTGGCCAGTACTCTCGGAGTCGACGGAAACAACTTGTCCAGA T A 	S 	S 	V 	V 	Q R 	A 	A 	Q S 	L A 	S 	T 	L G 	V 	D G 	N 	N 	L S 	R
ACTGCCTCCAGCGTGGTACAGAGAGCCGCTCAGTCGTTGGCCAGTACTCTCGGAGTCGACGGAAACAACTTGTCCAGA T A 	S 	S 	V 	V 	Q R 	A 	A 	Q S 	L A 	S 	T 	L G 	V 	D G 	N 	N 	L S 	R
ACTGCCTCCAGCGTGGTACAGAGAGCCGCTCAGTCGTTGGCCAGTACTCTCGGAGTCGACGGAAACAACTTGTCCAGA T A 	S 	S 	V 	V 	Q R 	A 	A 	Q S 	L A 	S 	T 	L G 	V 	D G 	N 	N 	L S 	R
ACTGCCTCCAGCGTGGTACAGAGAGCCGCTCAGTCGTTGGCCAGTACTCTCGGAGTCGACGGAAACAACTTGTCCAGA T A 	S 	S 	V 	V 	Q R 	A 	A 	Q S 	L A 	S 	T 	L G 	V 	D G 	N 	N 	L S 	R
ACTGCTTCCAGCGTGGTACAGAGAGCCGCTCAGTCGTTGGCCAGTACTCTCGGAGTCGACGGAAACAACTTGTCCAGA T A 	S 	S 	V 	V 	Q R 	A 	A 	Q S 	L A 	S 	T 	L G 	V 	D G 	N 	N 	L S 	R
ACTGCCTCCAGCGTGGTACAGAGAGCCGCTCAGTCGTTGGCCAGTACTCTCGGAGTCGACGGAAACAACTTGTCCAGA T A 	S 	S 	V 	V 	Q R 	A 	A 	Q S 	L A 	S 	T 	L G 	V 	D G 	N 	N 	L S 	R
ACTGCCTCCAGCGTGGTACAGAGAGCCGCTCAGTCGTTGGCCAGTACTCTCGGAGTCGACGGAAACAACTTGTCCAGA

T A 	S 	S 	V 	V 	Q R 	A 	A 	Q S 	L
```

A	S	T	L G	V	D G	N	N	L S	R

Aar_R_16

ACTGCCTCCAGCGTGGTACAGAGAGCCGCTCAGTCGTTGGCCAGTACTCTCGGAGTCGACGGAAACAACTTGTCCAGA

T A	S	S	V	V	Q R

A	A	Q S	L A	S	T	L G	V	D G	N	N	L S	R

Aar_R_17

ACTGCCTCCAGCGTGGTACAGAGAGCCGCTCAGTCGTTGGCCAGTACTCTCGGAGTCGACGGAAACAACTTGTCCAGA

T A	S	S	V	V	Q R	A	A	Q S	L

A	S	T	L G	V	D G	N	N	L S	R

Aar_R_18

ACTGCCTCCAGCGTGGTACAGAGAGCCGCTCAGTCGTTGGCCAGTACTCTCGGAGTCGACGGAAACAACTTGTCCAGA

T A	S	S	V	V	Q R	A	A	Q S	L

A	S	T	L G	V	D G	N	N	L S	R

Aar_R_19

ACTGCCTCCAGCGTGGTACAGAGAGCCGCTCAGTCGTTGGCCAGTACTCTCGGAGTCGACGGAAACAACTTGTCCAGA

T A	S	S	V	V	Q R	A	A	Q S	L A

S	T	L G	V	D G	N	N	L S	R

Aar_R_20

ACTGCCTCCAGCGTGGTACAGAGAGCCGCTCAGTCGTTGGCCAGTACTCTCGGAGTCGACGGAAACAACTTGTCCAGA T A	S	S	V	V	Q R	A	A	Q S	L A	S	T	L G	V	D G	N	N	L S	R

**1**

**Supplementary Figure S1**

**160	170	180	190**

**200**

**210	220	230**

Aar_R_1

Aar_R_2

Aar_R_3

Aar_R_4

Aar_R_5

Aar_R_6

Aar_R_7

Aar_R_8

Aar_R_9

Aar_R_10

Aar_R_11

Aar_R_12

Aar_R_13

Aar_R_14

Aar_R_15

```
GTAGCATTACAAGCCATCTCTCAAGTGCCCACAGGGTCTGACACTTCAGCTTACGCTCAAGCCTTCTCTAGTGCGCTC V A 	L Q A I S 	Q V 	P T G S 	D T S A 	Y A 	Q A 	F S S A 	L
GTAGCATTACAAGCCACCTCTCAAGTGCCCACAGGGTCTGACACTTCAGCTTACGCTCAAGCCTTCTCTAGTGCGCTC V A 	L Q A 	T S 	Q V 	P T G S 	D T S A 	Y A 	Q A 	F S S A 	L
GTAGCATTACAAGCCATCTCTCAAGTGCCCACAGGGTCTGACACTTCAGCTTACGCTCAAGCCTTCTCTAGTGCGCTC V A 	L Q A I S 	Q V 	P T G S 	D T S A 	Y A 	Q A 	F S S A 	L
GTAGCATTACAAGCCATCTCTCAAGTGCCCACAGGGTCTGACACTTCAGCTTACGCTCAAGCCTTCTCTAGTGCGCTC V A 	L Q A I S 	Q V 	P T G S 	D T S A 	Y A 	Q A 	F S S A 	L
GTAGCATTACAAGCCATCTCTCAAGTGCCCACAGGGTCTGACACTTCAGCTTACGCTCAAGCCTTCTCTAGTGCGCTC V A 	L Q A I S 	Q V 	P T G S 	D T S A 	Y A 	Q A 	F S S A 	L
GTAGCATTACAAGCCATCTCTCAAGTGCCCACAGGGTCTGACACTTCAGCTTACGCTCAAGCCTTCTCTAGTGCGCTC V A 	L Q A I S 	Q V 	P T G S 	D T S A 	Y A 	Q A 	F S S A 	L
GTAGCATTACAAGCCATCTCTCAAGTGCCCACAGGGTCTGACACTTCAGCTTACGCTCAAGCCTTCTCTAGTGCGCTC V A 	L Q A I S 	Q V 	P T G S 	D T S A 	Y A 	Q A 	F S S A 	L
GTAGCATTACAAGCCATCTCTCAAGTGCCCACAGGGTCTGACACTTCAGCTTACGCTCAAGCCTTCTCTAGTGCACTC V A 	L Q A I S 	Q V 	P T G S 	D T S A 	Y A 	Q A 	F S S A 	L
GTAGCATTACAAGCCATCTCTCAAGTGCCCACAGGGTCTGACACTTCAGCTTACGCTCAAGCCTTCTCTAGTGCGCTC V A 	L Q A I S 	Q V 	P T G S 	D T S A 	Y A 	Q A 	F S S A 	L
GTAGCATTACAAGCCATCTCTCAAGTGCCCACAGGGTCTGACACTTCAGCTTACGCTCAAGCCTTCTCTAGTGCGCTC V A 	L Q A I S 	Q V 	P T G S 	D T S A 	Y A 	Q A 	F S S A 	L
GTAGCATTACAAGCCATCTCTCAAGTGCCCACAGGGTCTGACACTTCAGCTTACGCTCAAGCCTTCTCTAGTGCACTC V A 	L Q A I S 	Q V 	P T G S 	D T S A 	Y A 	Q A 	F S S A 	L
GTAGCATTACAAGCCATCTCTCAAGTGCCCACAGGGTCTGACACTTCAGCTTACGCTCAAGCCTTCTCTAGTGCGCTC V A 	L Q A I S 	Q V 	P T G S 	D T S A 	Y A 	Q A 	F S S A 	L
GTAGCATTACAAGCCATCTCTCAAGTGCCCACAGGGTCTGACACTTCAGCTTACGCTCAAGCCTTCTCTAGTGCGCTC V A 	L Q A I S 	Q V 	P T G S 	D T S A 	Y A 	Q A 	F S S A 	L
GTAGCATTACAAGCCATCTCTCAAGTGCCCACAGGGTCTGACACTTCAGCTTACGCTCAAGCCTTCTCTAGTGCACTC V A 	L Q A I S 	Q V 	P T G S 	D T S A 	Y A 	Q A 	F S S A 	L
GTAGCATTACAAGCCATCTCTCAAGTGCCCACAGGGTCTGACACTTCAGCTTACGCTCAAGCCTTCTCTAGTGCGCTC

V A 	L Q A I S 	Q V 	P T G S 	D T S A 	Y A 	Q
```

A	F S S A	L

Aar_R_16

GTAGCATTACAAGCCATCTCTCAAGTGCCCACAGGGTCTGACACTTCAGCTTACGCTCAAGCCTTCTCTAGTGCGCTC

V A	L Q A I S	Q V	P T G S	D T

S A	Y A	Q A	F S S A	L

Aar_R_17

GTAGCATTACAAGCCATCTCTCAAGTGCCCACAGGGTCTGACACTTCAGCTTACGCTCAAGCCTTCTCTAGTGCGCTC

V A	L Q A I S	Q V	P T G S	D T S A	Y A	Q

A	F S S A	L

Aar_R_18

GTAGCATTACAAGCCATCTCTCAAGTGCCCACAGGGTCTGACACTTCAGCTTACGCTCAAGCCTTCTCTAGTGCGCTC

V A	L Q A I S	Q V	P T G S	D T S A	Y A	Q

A	F S S A	L

Aar_R_19

GTAGCATTACAAGCCATCTCTCAAGTGCCCACAGGGTCTGACACTTCAGCTTACGCTCAAGCCTTCTCTAGTGCGCTC

V A	L Q A I S	Q V	P T G S	D T S A	Y A	Q A

F S S A	L

Aar_R_20

GTAGCATTACAAGCCATCTCTCAAGTGCCCACAGGGTCTGACACTTCAGCTTACGCTCAAGCCTTCTCTAGTGCACTC V A	L Q A I S	Q V	P T G S	D T S A	Y A	Q A	F S S A	L

Aar_R_1

Aar_R_2

Aar_R_3

Aar_R_4

Aar_R_5

Aar_R_6

Aar_R_7

Aar_R_8

Aar_R_9

Aar_R_10

Aar_R_11

Aar_R_12

Aar_R_13

Aar_R_14

Aar_R_15

Aar_R_16

**240	250	260	270	280	290	300	310**

TTCAATGCCGGAGTTCTCAACGCAAGCAACATTGACACATTGGGATCCCGAGTTCTCTCAGCTGTTTTGAACGGAGTA F N A G V	L N A S N I	D T L G S R V	L S A V	L N G V

```
TTCAATGCCGGAGTTCTCAACGCAAGCAACATTGACACATTGGGATCCCGAGTTCTCTCAGCTGTTTTGAACGGAGTA F N A G V 	L N A S N I 	D T L G S R V 	L S A V 	L N G V
TTCAATGCCGGAGTTCTCAACGCAAGCAACATTGACACATTGGGATCCCGAGTTCTCTCAGCTGTTTTGAACGGAGTA F N A G V 	L N A S N I 	D T L G S R V 	L S A V 	L N G V
TTCAATGCCGGAGTTCTCAACGCAAGCAACATTGACACATTGGGATCCCGAGTTCTCTCAGCTGTTTTGAACGGAGTA F N A G V 	L N A S N I 	D T L G S R V 	L S A V 	L N G V
TTCAATGCCGGAATTCTCAATGCAAGCAACATTGACACATTGGGATCCCGAGTTCTCTCAGCTGTTTTGAACGGAGTA F N A G I 	L N A S N I 	D T L G S R V 	L S A V 	L N G V
TTCAATGCCGGAGTTCTCAACGCAAGCAACATTGACACATTGGGATCCCGAGTTCTCTCAGCTGTTTTGAACGGAGTA F N A G V 	L N A S N I 	D T L G S R V 	L S A V 	L N G V
TTCAATGCCGGAGTTCTCAATGCAAGCAACATTGACACATTGGGATCCCGAGTTCTCTCAGCTGTTTTGAACGGAGTA F N A G V 	L N A S N I 	D T L G S R V 	L S A V 	L N G V
TTCAATGCCGGAGTTCTCAATGCAAGCAACATTGACACATTGGGATCCCGAGTTCTCTCAGCTGTTTTGAACGGAGTA F N A G V 	L N A S N I 	D T L G S R V 	L S A V 	L N G V
TTCAATGCCGGAGTTCTCAATGCAAGCAACATTGACACATTGGGATCCCGAGTTCTCTCAGCTGTTTTGAACGGAGTA F N A G V 	L N A S N I 	D T L G S R V 	L S A V 	L N G V
TTCAATGCCGGAGTTCTCAATGCAAGCAACATTGACACATTGGGATCCCGAGTTCTCTCAGCTGTTTTGAACGGAGTA F N A G V 	L N A S N I 	D T L G S R V 	L S A V 	L N G V
TTCAATGCCGGAGTTCTCAATGCAAGCAACATTGACACATTGGGATCCCGAGTTCTCTCAGCTGTTTTGAACGGAGTA F N A G V 	L N A S N I 	D T L G S R V 	L S A V 	L N G V
TTCAATGCCGGAGTTCTCAATGCAAGCAACATTGACACATTGGGATCCCGAGTTCTCTCAGCTGTTTTGAACGGAGTA F N A G V 	L N A S N I 	D T L G S R V 	L S A V 	L N G V
TTCAATGCCGGAGTTCTCAATGCAAGCAACATAGACACATTGGGATCCCGAGTTCTCTCAGCTGTTTTGAACGGAGTA F N A G V 	L N A S N I 	D T L G S R V 	L S A V 	L N G V
TTCAATGCCGGAGTTCTCAATGCAAGCAACATTGACACATTGGGATCCCGAGTTCTCTCAGCTGTTTTGAACGGAGTA F N A G V 	L N A S N I 	D T L G S R V 	L S A V 	L N G V
TTCAATGCCGGAGTTCTCAATGCAAGCAACATTGACACATTGGGATCCCGAGTTCTCTCAGCTGTTTTGAACGGAGTA F N A G V 	L N A S N I 	D T L G S R V 	L S A V 	L N G V
TTCAATGCCGGAGTTCTCTATGCAAGCAACATTGACACATTGGGATCCCGAGTTCTCTCAGCTGTTTTGAACGGAGTA

F N A G V 	L Y A S N I 	D T L G S R V 	L S A V
```

L N G V

Aar_R_17

Aar_R_18

Aar_R_19

Aar_R_20

TTCAATGCCGGAGTTCTCAATGCAAGCAACATTGACACATTGGGATCCCGAGTTCTCTCAGCTGTTTTGAACGGAGTA F N A G V	L N A S N I	D T L G S R V	L S A V	L N G V

```
TTCAATGCCGGAGTTCTCAATGCAAGCAACATTGACACATTGGGATCCCGAGTTCTCTCAGCTGTTTTGAACGGAGTA F N A G V 	L N A S N I 	D T L G S R V 	L S A V 	L N G V
TTCAATGCCGGAGTTCTCAATGCAAGCAACATTGACACATTGGGATCCCGAGTTCTCTCAGCTGTTTTGAACGGAGTA F N A G V 	L N A S N I 	D T L G S R V 	L S A V 	L N G V
TTCAATGCCGGAGTTCTCAATGCAAGCAACATTGACACATTGGGATCCCGAGTTCTCTCAGCTGTTTTGAACGGAGTA F N A G V 	L N A S N I 	D T L G S R V 	L S A V 	L N G V
```

**2**

Supplementary Figure Sl

Aar_R_l

Aar_R_2

Aar_R_3

Aar_R_4

Aar_R_5

Aar_R_6

Aar_R_7

Aar_R_8

Aar_R_9

Aar_R_10

Aar_R_11

Aar_R_12

Aar_R_13

Aar_R_14

Aar_R_15

Aar_R_16

Aar_R_17

Aar_R_18

Aar_R_19

Aar_R_20

**320**

I

**330**

I

**340**

I

**350**

I

**360**

I

**370**

I

**380**

I

**390**

I

Aar_R_l

Aar_R_2

Aar_R_3

Aar_R_4

Aar_R_5

Aar_R_6

Aar_R_7

Aar_R_8

Aar_R_9

Aar_R_10 Aar_R_ll Aar_R_12

Aar_R_13

Aar_R_14

Aar_R_15

Aar_R_16

Aar_R_17

Aar_R_18

Aar_R_20

**400**

I

**410**

I

**420**

I

**430**

I

**440**

I

**450**

I

**460**

I

3

**Supplementary Figure Sl**

Aar_R_1

Aar_R_2

Aar_R_3

Aar_R_4

Aar_R_5

Aar_R_6

Aar_R_7

Aar_R_8

Aar_R_9

Aar_R_10

Aar_R_11

Aar_R_12

Aar_R_13

Aar_R_14

Aar_R_15

Aar_R_16

Aar_R_17

Aar_R_18

Aar_R_19

Aar_R_20

**470	480	490	500	510	520	530	540**

CCTTCTGGCTACACAGGACCAGTGGGTGGCGGAGCGCAGTTCGGTTCTGCTTCAGGCCAAAGCTCTTTCGGTCAGACAPSGYTGPVG G GAQFGSASGQS SFGQTCCTTCTGGCTACACAGGACCAGTGGGTGGCGGAGCGCAGTTCGGTTCTGCTTCAGGCCAAAGCTCTTTCGGTCAGACAPSGYTGPVG G GAQFGSASGQS SFGQTCCTTCTGGCTACACAGGACCAGTGGGTGGCGGAGCGCAGTTCGGTTCTGCTTCAGGCCAAAGCTCTTTCGGTCAGACAPSGYTGPVG G GAQFGSASGQS SFGQTCCTTCTGGCTACACAGGACCAGTGGGTGGCGGAGCGCAGTTCGGTTCTGCTTCAGGCCAAAGCTCTTTCGGTCAGACAPSGYTGPVG G GAQFGSASGQS SFGQTCCTTCTGGCTACACAGGACCAGTGGGTGGCGGAGCGCAGTTCGGTTCTGCTTCAGGCCAAAGCTCTTTCGGTCAGACAPSGYTGPVG G GAQFGSASGQS SFGQTCCTTCTGGCTACACAGGACCAGTGGGTGGCGGAGCGCAGTTCGGTTCTGCTTCAGGCCAAAGCTCTTTCGGTCAGACAPSGYTGPVG G GAQFGSASGQS SFGQTCCTTCTGGCTACACAGGACCAGTGGGTGGCGGAGCGCAGTTCGGTTCAGCTTCAGGCCAAAGCTCTTTCGGTCAGACAPSGYTGPVG G GAQFGSASGQS SFGQTCCTTCTGGCTACACAGGACCAGTGGGTGGCGGAGCGCAGTTCGGTTCTGCTTCAGGCCAAAGCTCTTTCGGTCAGACAPSGYTGPVG G GAQFGSASGQS SFGQTCCTTCTGGATACACAGGACCAGTGGGTGGCGGAGCGCAGTTCGGTTCTGCTTCAGGCCAAAGCTCTTTCGGTCAGACAPSGYTGPVG G GAQFGSASGQS SFGQTCCTTCTGGCTACACAGGACCAGTGGGTGGTGGAGCGCAGTTCGGTTCTGCTTCAGGCCAAAGTTCTTTCGGTCAGACAPSGYTGPVG G GAQFGSASGQS SFGQTTCTTCTGGTTACACAGGACCAAAGGGTGGCGGAGCGCAGTTCGGTTCTGCTTCAGGCCAAAGCTCTTTCGGTCAGACAS SGYTGPKG G GAQFGSASGQS SFGQTCCTTCTGGCTACATAGGACCAGTGGGTGGCGGAGCGCAGTTCGGTTCTGCTTCAGGCCAAAGCTCTTTCGGTCAGACAPSGYIGPVG G GAQFGSASGQS SFGQTCCTTCTGGCTACACAGGACCAGTGGGTGGCGGAGCGCAGTTCGGTTCTGCTTCAGGCCAAAGCTCTTTCGGTCAGACAPSGYTGPVG G GAQFGSASGQS SFGQTCCTTCTGGCTACACAGGACCAGTGGGTGGCGGAGCGCAGTTCGGTTCTGCTTCAGGCCAAAGCTCTTTCGGTCAGACAPSGYTGPVG G GAQFGSASGQS SFGQTCCTTCTGGCTACACAGGACCAGTGGGTGGCGGAGCGCAGTTCGGTTCTGCTTCAGGCCAAAGCTCTTTCGGTCAGACAPSGYTGPVG G GAQFGSASGQS SFGQTCCTTCTGGCTACACAGGACCAAAGGGTGGCGGAGCGCAGTTCGGTTCTGCTTCAGGCCAAAGCTCTTTCGGTCAGACAPSGYTGPKG G GAQFGSASGQS SFGQTCCTTCTGGCTACACAGGACCAGTGGGTGGCGGAGCGCAGTTCGGTTCTGCTTCAGGCCAAAGCTCTTTCGGTCAGACAPSGYTGPVG G GAQFGSASGQS SFGQTCCTTCTGGCTACACAGGACCAGTGGGTGGCGGAGCGCAGTTCGGTTCTGCTTCAGGCCAAAGCTCTTTCGGTCAGACAPSGYTGPVG G GAQFGSASGQS SFGQTCCTTCTGGCTACACAGGACCAGTGGGTGGCGGAGCGCAGTTCGGTTCTGCTTCAGGCCAAAGCTCTTTCGGTCAGACAPSGYTGPVG G GAQFGSASGQS SFGQTCCTTCTGGCTACACAGGACCAGTGGGTGGCGGAGCGCAGTTCGGTTCTTCTTCAGGCCAAAGCTCTTTCGGTCAAACAPSGYTGPVG G GAQGS S SGQS SFGQT

TCAGGATTAACCGCATCTTCTGGCGGACAAGCCGCTTTCGGTGGAACCAGTGGAGCGTCTGCTGGCSGLTAS SG GQA AFG GTSGASAGTCAGGATTTACCGCATCTTCTGGCGGACAAGCCGCTTTCGGTGGAACCAGTGGAGCGTCTGCTGGCSGFTAS SG GQA AFG GTSGASAGTCAGGATTTACCGCATCTTCTGGCGGACAAGCCGCTTTCGGTGGAACCAGTGGAGCGTCTGCTGGCSGFTAS SG GQA AFG GTSGASAGTCAGAATTTACCGCATCTTCTGGCGGACAAGCCGCTTTCGGTGGAACCAGTGGAGCGTCTGCTGGCSEFTAS SG GQA AFG GTSGASAGTCAGGATTTACCGCATCTTCTGGCGGACAAGCCGCTTTCGGTGGAACCAGTGGAGCGTCTGCTGGCSGFTAS SG GQA AFG GTSGASAGTCAGGATTTACCGCATCTTCTGGCGGACAAGCCGCTTTCGGTGGAACCAGTGGAGCGTCTGCTGGCSGFTAS SG GQA AFG GTSGASAGTCAGGATTTACCGCATCTTCTGGCGGACAAGCCGCTTTCGGTGGAACCAGTGGAGCGTCTGCTGGCSGFTAS SG GQA AFG GTSGASAGTCAGGATTTACCGCATCTTCTGGCGGACAAGCCGCTTTCGGTGGAACCAGTGGAGCGTCTGCTGGCSGFTAS SG GQA AFG GTSGASAGTCAGGATTTACCGCATCTTCTGGCGGACAAGCCGCTTTCGGTGGAACCAGTGGAGCGTCTGCTGACSGFTAS SG GQA AFG GTSGASADTCAGGATTTACCGCATCTTCTGGCGGACAAGCCGCTTTCGGTGGAACCAGTGGAGCGTCTGCTGGCSGFTAS SG GQA AFG GTSGASAGTCAGGATTTACCGCATCTTCTGGCGGACAAGCCGCTTTCGGTGGAACCAGTGGAGCGTCTGCTGGCSGFTAS SG GQA AFG GTSGASAGTCAGGATTTACCGCATCTTCTGGCGGACAAGCCGCTTTCGGTGGAACCAGTGGAGCGTCTGCTGGCSGFTAS SG GQA AFG GTSGASAGTCAGGATTTACCGCATCTTCTGGCGGACAAGCCGCTTTCGGTGGAACCAGTGGAGCGTCTGCTGGCSGFTAS SG GQA AFG GTSGASAGTCAGGATTTACCGCATCTTCTGATGGACAAGCCGCTTTCGGTGGAACCAGTGGAGCGTCTGCTGGCSGFTAS SDGQA AFG GTSGASAGTCAGGATTTACCGCATCTTCTGATGGACAAGCCGCTTTCGGTGGAACCAGTGGAGCGTCTGCTGGCSGFTAS SDGQA AFG GTSGASAGTCAGGATTTACCGCATCTTCTAGCGGACAAGCCGCTTTCGGTGGAACCAGYGGAGCGTCTGCTGGCSGFTAS S SGQA AFG GTSGASAGTCAGGATTTACCGCATCTTCTGATGGACAAGCCGCTTTCGGTGGAACCAGTGGAGCGTCTGCTGGCSGFTAS SDGQA AFG GTSGASAGTCAGGATTTACCGCATCTTCTGATGGACAAGCCGCTTTCGGTGGAACCAGTGGAGCGTCTGCTGGCSGFTAS SDGQA AFG GTSGASAGTCAGGATTTACCGCATCTTCTGGCGGACAAGCCGCTTTCGGTGGAACCAGTGGAGCGTCTGCTGGCSGFTAS SG GQA AFG GTSGASAGTCAGGATTTACASGFT

Aar_R_1

Aar_R_2

Aar_R_3

Aar_R_4

Aar_R_5

Aar_R_6

Aar_R_7

Aar_R_8

Aar_R_9

Aar_R_10

Aar_R_11

Aar_R_12

Aar_R_13

Aar_R_14

Aar_R_15

Aar_R_16

Aar_R_17

Aar_R_18

Aar_R_19

Aar_R_20

**550	560	570	580	590	600	612**

4

**Supplementary Figure S1. Nucleotide alignment of the 20 repeat units (Aar_R) from the complete *A. argentata* AcSp1 repetitive region.** Alignment position numbers shown in increments of ten. Frame 1 translation is shown under nucleotide sequences.

Alignment prepared with Geneious v6.1.6 (Biomatters Ltd., Auckland, NZ).

Dsfibroin1a

Frame 1

DsMaSp2

Frame 1

NcMaSp1a

Frame 1

NcMaSp1b

Frame 1

NcMaSp2

Frame 1

NiMaSp2

Frame 1

LgMaSp1

Frame 1

LheMaSp1

Frame 1

LheMaSp2

Frame 1

EaMaSp

Frame 1

AtMaSp2

Frame 1

AapMaSp

Frame 1

UdMiSp

Frame 1

MgMiSp

Frame 1

LheMiSp

Frame 1

AvMiSp

Frame 1

Bcfibroin1

Frame 1

DcMaSp_like

Frame 1

DcMaSp

Frame 1

AargAcsp1Full

Frame 1

LheAcSp1

Frame 1

LgAcSp1

Frame 1

NiFlag

Frame 1

NcFlag

Frame 1

AvFlag

Frame 1

AapTuSp1

Frame 1

NcTuSp1

Frame 1

LheTuSp1

Frame 1

AbTuSp1

Frame 1

AaTuSp1

Frame 1

1	10	20	30	40	50	60	70	80	90	100	110	120	130

?????????????????????????????????????????????????????????????????????????????????????????????????????????????????????????????????????????

? ? ? ? ? ? ? ? ? ? ? ? ? ? ? ? ? ? ? ? ? ? ? ? ? ? ? ? ? ? ? ? ? ? ? ? ? ? ? ? ? ? ? ? ? ?

??????CCTCTGCTTGCTCTA GTAA TTTCCTCA TTGA GTGGGGCTGCAA CA ------------------------------GGCGCAA GTA GCTA TGCA TCGGCGAA CA CCA CA CCTTGGA GGGA CGCA GCGA CA GC

?	?	P	L	L A	L V	I	S	S	L	S	G A	A	T ------------------------------ G A	S	S	Y A	S A	N	T	T	P W	R	D A	A	T A

A TGA CTTGGA CA GCTCGA CTTGCGCTTTCA TTTTTA GCGGTGA TCTGCA CC------------------------------CA GA GCTTGTTTGCTCA GGGTCAA ---AA CA CCCCA TGGTCAA GTA CGGAA CTA GC M	T W	T A	R	L A	L	S	F	L A	V	I	C	T ------------------------------ Q	S	L	F A	Q	G	Q --- N	T	P W	S	S	T	E	L A

A TGA CTTGGA CA GCTCGA CTTGCGCTTTCAA TTTTA GCGGTGCTTTGCA CC------------------------------CA GGGCCTGTTTGCTCA GGGTCAA ---AA CA CCCCA TGGTCAA GTA CGGAA CTA GC M	T W	T A	R	L A	L	S I	L A	V	L	C	T ------------------------------ Q	G	L	F A	Q	G	Q --- N	T	P W	S	S	T	E	L A

A TGA GTTGGTCCA CT---CTA GCTTTA GCGA TTTTA GCGGTGCTTA GCA CC------------------------------CAA TCCA TCTA CGCTTCTGCA CAA GCAA GGTCTCCA TGGA GTGA CA CA GCCA CA GC M	S W	S	T --- L A	L A	I	L A	V	L	S	T ------------------------------ Q	S I	Y A	S A	Q A	R	S	P W	S	D	T A	T A

A TGA GTTGGTCCA CT---CTA GCTTTA GCGA TTA TCGCGGTGCTTA GCA CC------------------------------CA GTGCA TTTTTA TTGCA GGA CAA GCAAA CA CA CCA TGGA GCGA CA CTGCCA CA GC M	S W	S	T --- L A	L A	I	I	A	V	L	S	T ------------------------------ Q	C I	F I	A	G	Q A	N	T	P W	S	D	T A	T A

A TGAA TTTGTCAA CTCGA CTTGCTCTA TCA TTCCTCGTNGTGCTTTGCA CT------------------------------CAA GGTCTGTA TGTTCTGGGA CAA GCAAA CA CTCCA TGGTCTA GTAAA CAAAA TGC M	N	L	S	T R	L A	L	S	F	L V	V	L	C	T ------------------------------ Q	G	L	Y V	L	G	Q A	N	T	P W	S	S	K	Q	N A

A TGA CTTGGTCAA CTCGA CTTGCCTTA TCA TTTCTTTTCGTGCTCTGCA CT------------------------------CA GA GCCTGTA CGCTTTGGCGCAA GCCAA CA CGCCA TGGTCAA GTAAA GCGAA TGC M	T W	S	T R	L A	L	S	F	L	F V	L	C	T ------------------------------ Q	S	L	Y A	L A	Q A	N	T	P W	S	S	K A	N A

A TGAA TTGGTCTA CTCGA CTTGTGTTGTCAA TA CTCGTA GTGCTTTGCA CT------------------------------CA GA GCCTCTGTGCTCTGGGA CAA GCAAA CA CTCCGTGGTCCA GTAAA GAAAA CGC M	N W	S	T R	L V	L	S I	L V	V	L	C	T ------------------------------ Q	S	L	C A	L	G	Q A	N	T	P W	S	S	K	E	N A

A TGA GTTGGA CA GCGCGA CTTGCTCTTCTA TTGCTCTTTGTA GCTTGC---------------------------------CA GGGCTCCA GTTCTCTGGCCTCA CA CA CTA CA CCA TGGA CAAA CCCA GGA CTCGC M	S W	T A	R	L A	L	L	L	L	F V	A	C --------------------------------- Q	G	S	S	S	L A	S	H	T	T	P W	T	N	P	G	L A

A TGAA TTGGTCAA TTCGTCTTGCTCTTTTA GGTTTCGTGGTGCTCA GCA CC------------------------------CAAA CTGTA TTTTCTGCTGGCCA GGGTGCAA CTCCA TGGGA GAA CTCGCAA CTGGC M	N W	S I	R	L A	L	L	G	F V	V	L	S	T ------------------------------ Q	T V	F	S A	G	Q	G A	T	P W	E	N	S	Q	L A

A TGA CTTGGA CTGTGCGA CTA GCTA TCCCCA TTCTC---A TTTTGA TCCTT------------------------------CAA GGGTCAAAA TGTCTTGGA CA GA GCAA C---CCGTGGA CA GA CA CTGCCA CGGC M	T W	T V	R	L A	I	P I	L --- I	L I	L ------------------------------ Q	G	S	K	C	L	G	Q	S	N --- P W	T	D	T A	T A

??????????????????????????????TTCGCT---GTTTTTTGCGCC------------------------------CA GA GCTA CTCA GCTCTGGGGCAA GGA GCCTCA GTA TGGTCAA GCCCCCAAA TGGC

?	?	?	?	?	?	?	?	?	?	F A --- V	F	C A ------------------------------ Q	S	Y	S A	L	G	Q	G A	S V	W	S	S	P	Q M	A

???????????????CGA CTA TCGCTA TTCTTCCTC---GTCCTGTTCA TT------------------------------CA GGGC---TTTA TGGCTCA TGGC------CA TA TCTGGGGGA CTCCA GGA GCTGG

?	?	?	?	? R	L	S	L	F	F	L --- V	L	F I ------------------------------ Q	G --- F M	A	H	G ------ H I	W	G	T	P	G A	G

A TGCA TA TTCCA GCTCA GTTA TCTTTA CTCTTCCTT---CTTCTGTGCGCC------------------------------CA GA GC---TTCGTCTCTTTA GA TGCCGCCA GTGTCTGGGA CA GCA CTGCAA CA GC M	H I	P A	Q	L	S	L	L	F	L --- L	L	C A ------------------------------ Q	S --- F V	S	L	D A	A	S V	W	D	S	T A	T A

A TGCA GTTGCCTA CTCGA TTA TCGTTTTTCTTCCTC---GTCCTGTTCA TT------------------------------CA GGGA TTTTTGTCCCA GA GTCAA ------CCAA TCTGGA CCAA CCCAAA TGCA GC M	Q	L	P	T R	L	S	F	F	F	L --- V	L	F I ------------------------------ Q	G	F	L	S	Q	S	Q ------ P I	W	T	N	P	N A	A

A TGGGTTGGTTAA CTCA TA TCA CTTTGTCGCTTTTCTTCGTTTCA GCGCA GTTCA GC------------------------A TCTCA TCTGCAA GAA CTAAA GTAA CGAA TGTA CCA TGGA CCGA TGAA GCCAAA GG M	G W	L	T	H I	T	L	S	L	F	F V	S A	Q	F	S ------------------------ I	S	S A	R	T	K V	T	N V	P W	T	D	E A	K	G

???????????????CGA CTA CTTTTCCTCGTCTTTGCGA CAA GTGGCGTTCTGGCA CA C---------------------CA GGGCCCTCA T------------A GAAA GA CA CCA TGGGA GA CCCCGGAAA TGGC

?	?	?	?	? R	L	L	F	L V	F A	T	S	G V	L A	H --------------------- Q	G	P	H ------------ R	K	T	P W	E	T	P	E M	A

A TGGGTTGCGTCGTTCGA CTA GTTTTCCTCGCA TTCGCGA CTA CGCAA TGCCTTCTA GCA GGG------------------CAA GGTGTCCA CGGA GCAA TG---GCCAA CA CGCCA TTTGCAAA TAA GA TCA TGGC M	G	C V	V	R	L V	F	L A	F A	T	T	Q	C	L	L A	G ------------------ Q	G V	H	G A	M --- A	N	T	P	F A	N	K I	M	A

A TGAA TTGGTTA CCTA CTCTCGCTTTTGCGA TTCTA CTGCTGTCA GTCCA GTA CGA TGCGGTG------------------CAAA GCGCGTCAA CTCTG------TCCA GGA GTCCTTGGGCCAA TCCGGCCAAA GC M	N W	L	P	T	L A	F A	I	L	L	L	S V	Q	Y	D A	V ------------------ Q	S A	S	T	L ------ S R	S	P W	A	N	P A	K A

A TGAA CTGGTTAA CTA GCCTCA GCTTAA TTTTTA TA TTA GCCTTTGTA CAAAA TGTA CAA GTCGAA GGAA GAAAA ------GGA CA CCA TCA TTCCA GTGGCTCA TCCAA GA GTCCTTGGGCAAA TCCGGCTAA GGC M	N W	L	T	S	L	S	L I	F I	L A	F V	Q	N V	Q V	E	G R	K ------ G	H	H	H	S	S	G	S	S	K	S	P W	A	N	P A	K A

??????????????????????????????????????????????????????????????????????????????????????????????????????TCA TCCAA GA GTCCTTGGGCAAA TCCGGCTAA GGC

?	?	?	?	?	?	?	?	?	?	?	?	?	?	?	?	?	?	?	?	?	?	?	?	?	?	?	?	?	?	?	?	?	?	S	S	K	S	P W	A	N	P A	K A

A TGGCCTGCTTTA CCTCGGCA GTGA TA TTTCTTTTCTTA GCGCA GTGTGCG------------------------------TCGA CGTA CGGAA GGGGGA TTA TA GTCAA CTCCCCTTTCTCAAA CCCTAA CA CA GC M	A	C	F	T	S A	V	I	F	L	F	L A	Q	C A ------------------------------ S	T	Y	G R	G I	I	V	N	S	P	F	S	N	P	N	T A

???GCTTGCTTTA CCTCGGCA GTGA TA TTTCTTTTCTTA GCGCA GTGTGCG------------------------------TCGA CGTA CGGAA GGGGGA TTA TA GCCAA CTCCCCTTTCTCAAA CCCTAA CA CA GC

? A	C	F	T	S A	V	I	F	L	F	L A	Q	C A ------------------------------ S	T	Y	G R	G I	I	A	N	S	P	F	S	N	P	N	T A

?????????????????????????????????????????????????????????????????????????????????????????????????????????????????????????????????????????

? ? ? ? ? ? ? ? ? ? ? ? ? ? ? ? ? ? ? ? ? ? ? ? ? ? ? ? ? ? ? ? ? ? ? ? ? ? ? ? ? ? ? ? ? ?

A TGGTTTGGA TAA CTA GCA TA GCA TGTCTGTTTA CCTTTA TA GCCA CA TTTGGCA GTCAA GTTGGA GCA CAA GGAA CTTCTTTTGCA TCA TCA GCAA CA GCA GGAA TA CGAAA TA TA TTTGGAAA TCCAAA CA CTGC M	V	W	I	T	S I	A	C	L	F	T	F I	A	T	F	G	S	Q V	G A	Q	G	T	S	F A	S	S A	T A	G I	R	N I	F	G	N	P	N	T A

A TGGTTTGGCTGA CAA GCA TA GCGTTTGTTGTGGCTCTTTTA GGA GCA CAA TA CGA CA TCGTGA CTGCT------------CA GGCAA TTTCA GTTGCAA CTCCTGTCCCA TCA GTGTTCA GTA GCCCTA GCCTTGC M	V	W	L	T	S I	A	F V	V	A	L	L	G A	Q	Y	D I	V	T A ------------ Q A	I	S V	A	T	P V	P	S V	F	S	S	P	S	L A

A TGGTTTGGTTGA CAA GTA CTGTGCTCCTA GCA TCCCTTCTGGGAA CA CTTGGA CTTCCA GCC------------------AA TA GTTTA TCA GGA GTTTCTGCCTCGGTCAA TA TTTTCAA TA GCCCCAA TGCA GC M	V	W	L	T	S	T V	L	L A	S	L	L	G	T	L	G	L	P A ------------------ N	S	L	S	G V	S A	S V	N I	F	N	S	P	N A	A

A TGGTTTGGCTCA CTA GCA TA GCGTTCCTCGTA GGCTTTCTGGGA GCA GTGTCGTCT------------------------CA GTCA GTT---GCA GTTA CCGCCGTTCCTTCA GTCTTCA GCA GTCCAAA TTTGGC M	V	W	L	T	S I	A	F	L V	G	F	L	G A	V	S	S ------------------------ Q	S V --- A	V	T A	V	P	S V	F	S	S	P	N	L A

??????????????????????????????????????????????????????????????????????????????????????????GCA GTTA CCGCCGTTCCA TCA GTCTTCA GTA GTCCGAA TTTA GC

?	?	?	?	?	?	?	?	?	?	?	?	?	?	?	?	?	?	?	?	?	?	?	?	?	?	?	?	?	? A	V	T A	V	P	S V	F	S	S	P	N	L A

Dsfibroin1a

Frame 1

DsMaSp2

Frame 1

NcMaSp1a

Frame 1

NcMaSp1b

Frame 1

NcMaSp2

Frame 1

NiMaSp2

Frame 1

LgMaSp1

Frame 1

LheMaSp1

Frame 1

LheMaSp2

Frame 1

EaMaSp

Frame 1

AtMaSp2

Frame 1

AapMaSp

Frame 1

UdMiSp

Frame 1

MgMiSp

Frame 1

LheMiSp

Frame 1

AvMiSp

Frame 1

Bcfibroin1

Frame 1

DcMaSp_like

Frame 1

DcMaSp

Frame 1

AargAcsp1Full

Frame 1

LheAcSp1

Frame 1

LgAcSp1

Frame 1

NiFlag

Frame 1

NcFlag

Frame 1

AvFlag

Frame 1

AapTuSp1

Frame 1

NcTuSp1

Frame 1

LheTuSp1

Frame 1

AbTuSp1

Frame 1

AaTuSp1

Frame 1

140	150	160	170	180	190	200	210	220	230	240	250	260	270

?????????????????????????????????????????????????????????????????????????TTCAA CGA TA TTA GCCA GA CGA TGGTGGA TGCCGTGAAAA GA TTA CA GA GGTCGAA TAA GA GCA

?	?	?	?	?	?	?	?	?	?	?	?	?	?	?	?	?	?	?	?	?	?	?	?	F	N	D I	S	Q	T M	V	D A	V	K R	L	Q R	S	N	K	S

GCAA GCTTTCA TCGGCAA CTTTA TGCAA GCCA TCTGTTA TGA CCCGGTCA TAAA TCCGCA GCAA TGCGA CGA TA TGAA TTCCA TGGCGA GCA CGA TA CTGGCCGGGGTA CAAAA TA TGGCA CGTGA CGGCAAAA TA T Q A	F I	G	N	F M	Q A	I	C	Y	D	P V	I	N	P	Q	Q	C	D	D M	N	S M	A	S	T I	L A	G V	Q	N M	A	R	D	G	K I

A GA TGCA TTCA TTAA TGCA TTCA TGAA CGAA GCTGGAA GAA CTGGA GCTTTTA CTGCGGA TCAA CTTGA TGA TA TGTCTA CCA TCGGA GA CA CAA TTAAAA CGGCGA TGGA CAAAA TGGCGA GAA GCAA CAA GA GTT D A	F I	N A	F M	N	E A	G R	T	G A	F	T A	D	Q	L	D	D M	S	T I	G	D	T I	K	T A	M	D	K M	A	R	S	N	K	S

A GA TGCA TTTA TCAA TGCA TTCTTAAA CGAA GCTGGAA GAA CTGGA GCTTTCA CTGCA GA TCAA CTCGA TGA CA TGTCTA CCA TTGGCGA CA CCCTTAAAA CGGCAA TGGA CAAAA TGGCAA GAA GTAA CAA GA GTT D A	F I	N A	F	L	N	E A	G R	T	G A	F	T A	D	Q	L	D	D M	S	T I	G	D	T	L	K	T A	M	D	K M	A	R	S	N	K	S

A GA TGCCTTCA TTCA GAA TTTCTTA GCA GCGGTTTCGGGAA GTGGA GCTTTCA CTTCA GA TCAA CTTGA TGA CA TGTCCA CGA TCGGCGA TA CCA TCA TGTCA GCAA TGGA CAAAA TGGCTA GAA GTAA CAA GA GCT D A	F I	Q	N	F	L A	A	V	S	G	S	G A	F	T	S	D	Q	L	D	D M	S	T I	G	D	T I	M	S A	M	D	K M	A	R	S	N	K	S

A GA TGCTTTCA TTCA GAA TTTCTTA GGA GCTGTTTCA GGAA GTGGA GCCTTTA CTCCA GA TCAA CTTGA TGA TA TGTCCA CA GTCGGA GA TA CCA TCA TGTCA GCAA TGGA CAAAA TGGCTCGCA GTAA CAA GA GCT D A	F I	Q	N	F	L	G A	V	S	G	S	G A	F	T	P	D	Q	L	D	D M	S	T V	G	D	T I	M	S A	M	D	K M	A	R	S	N	K	S

TGA CGCTTTTA TAA GTGCA TTCA TGA CTGCTCCTTCA CAAA GTGGA GCA TTTTCA TCGGA TCA GA TCGA TGA CA TGTCTGTCA TCA GCAA TA CA TTAA TGGCA GCAA TGGA TAA TA TGGGA ------GGAA GAA TTA D A	F I	S A	F M	T A	P	S	Q	S	G A	F	S	S	D	Q I	D	D M	S V	I	S	N	T	L M	A	A	M	D	N M	G ------ G R	I

TGA TGCTTTTA TCAA TTCCTTTA TTTCGGCA GCTTCGAA TA CTGGA TCCTTCTCCCAA GA TCA GA TGGAA GA TA TGTCA TTGA TTGGTAA TA CA TTAA TGGCA GCAA TGGA TAA TA TGGGT------GGAA GAA TTA D A	F I	N	S	F I	S A	A	S	N	T	G	S	F	S	Q	D	Q M	E	D M	S	L I	G	N	T	L M	A	A	M	D	N M	G ------ G R	I

TGA CGCTTTTA TA GGCGCA TTTA TGAA TGCTGCTTCA CAAA GTGGA GCA TTTTCA TCGGA TCA GA TA GA TGA TA TGTCA GTTA TTA GTAA TA CA TTGA TGGCTGCAA TGGA CAA CA TGGGT------GGAA GAA TCA D A	F I	G A	F M	N A	A	S	Q	S	G A	F	S	S	D	Q I	D	D M	S V	I	S	N	T	L M	A	A	M	D	N M	G ------ G R	I

A GAAAA CTTCA TGAA CA GTTTCA TGCAA GGCCTGA GCTCGA TGCCA GGTTTCA CGGCAA GCCAA TTGGA TGA TA TGTCAA CCA TCGCA CAA TCCA TGGTA CA GTCAA TA CAA TCCTTGGCGGCA CAA GGCA GGA CA T E	N	F M	N	S	F M	Q	G	L	S	S M	P	G	F	T A	S	Q	L	D	D M	S	T I	A	Q	S M	V	Q	S I	Q	S	L A	A	Q	G R	T

GGA GA GCTTCA TCA GCCGTTTTTTAA GA TTCA TA GGA CAAA GCGGA GCTTTTTCCCCAAA CCAA CTGGA TGA TA TGTCTTCTA TTGGA GA CA CCTTGAA GA CTGCAA TTGAAAAAA TGGCTCAAA GCCGAAAAA GTT E	S	F I	S R	F	L R	F I	G	Q	S	G A	F	S	P	N	Q	L	D	D M	S	S I	G	D	T	L	K	T A	I	E	K M	A	Q	S R	K	S

A GAAA GTTTTA TA TCGA GTGTAA TGTCA TCCGTTGCTAA CCA GGGA TGTCTCTCA TA CGA CCAAA TA GA CGA CA TGCAA GCTGTTGGCGA TA CAA TGCTGGCAA CAA TGGA CAA CTTGGTCA GAA GTGGTAA GA GTT E	S	F I	S	S V	M	S	S V	A	N	Q	G	C	L	S	Y	D	Q I	D	D M	Q A	V	G	D	T M	L A	T M	D	N	L V	R	S	G	K	S

CGA GAA CTTCA TGAA CGGCTTCTCCA TGGCTCTTTCGCAA GCTGGA GCA TTCA GTGGGCA GGA GA TGAAA GA CTTCGA TGA TGTCA GA GA TA TCA TGAA CTCTGCAA TGGA CAA GA TGA TAA GGTCCGGGAAAA GTG E	N	F M	N	G	F	S M	A	L	S	Q A	G A	F	S	G	Q	E M	K	D	F	D	D V	R	D I	M	N	S A	M	D	K M	I	R	S	G	K	S

AAAA TCGGTGA CCGGCA GCA TTGTTCAA TGTGCTGGGCA GTCA GGCGTCTTCA GTGGA GA CCA GA TGCA GGA CTTGGGA GA CA TGGCGGA TGCA GTAAA CA GA CA GTTGGA CCGA TTGGGA CCA ---AA TGCA CCT- K	S V	T	G	S I	V	Q	C A	G	Q	S	G V	F	S	G	D	Q M	Q	D	L	G	D M	A	D A	V	N R	Q	L	D R	L	G	P --- N A	P -

TGAA GCTTTTA TTGGAA GCTTCAA TTCTGGCA TGGAAA GA TCTGGA GTGTTGTCA CGGTCTCA GA TGGA TGA TA TCTCA TCCA TTA GCGA CA CCA TAA TA TCTGCCA TTGAAA GA ---AA CCCA ---AA CAA CTCC- E A	F I	G	S	F	N	S	G M	E R	S	G V	L	S R	S	Q M	D	D I	S	S I	S	D	T I	I	S A	I	E R --- N	P --- N	N	S -

AA TGA CCA TGA CCAA CAA CCTGGTCCAA TGTGCGA GTCGGTCA GGTGTGCTCA CA GCCGA TCA GA TGGA CGA CA TGGGAA TGA TGGCA GA CTCTGTTAA CTCGCA GA TGCA GAAAA TGGGA CCA ---AA CCCA CCT- M	T M	T	N	N	L V	Q	C A	S R	S	G V	L	T A	D	Q M	D	D M	G M	M	A	D	S V	N	S	Q M	Q	K M	G	P --- N	P	P -

TAAAAAA TTTCTCA GTA CCTTCTTA GA TTA TGCA TTA GA CCA CGGCCTGTTTCCGCA GCA GGA GA GA GA TGA TTTGGAA GCAA TA TCCCAAAA CCTCA TTCCA GTA TTCCGGAA GA CAA TGGA TTCA GGGGGGAA TG K	K	F	L	S	T	F	L	D	Y A	L	D	H	G	L	F	P	Q	Q	E R	D	D	L	E A	I	S	Q	N	L I	P V	F R	K	T M	D	S	G	G	N

A GAAAA CTTTA TGAA CAA TGTCCGCGAAAA TTTGGAA GCTTCCCGAA TA TTTCCGGA CGA GCTGA TGAAA GA CA TGGA GGCCA TCA CAAA CA CGA TGA TCGCA GCGGTCGA TGGGTTA GAA GCCCAA CA TA GGTCA T E	N	F M	N	N V	R	E	N	L	E A	S R	I	F	P	D	E	L M	K	D M	E A	I	T	N	T M	I	A	A	V	D	G	L	E A	Q	H R	S

GGA GGA CTTTA TGAA TAA GTTCA CTAA CCAA CTGGCGAA CTCA CCCTA CTTCTCGTCGCA GCA GAAA GAA GA TA TGA GTA GCA TCAA GGA CGAA TTAA TCTCA GTGA TCGAAA GTA TGGA TTCTGCTCA TAA GA GCT E	D	F M	N	K	F	T	N	Q	L A	N	S	P	Y	F	S	S	Q	Q	K	E	D M	S	S I	K	D	E	L I	S V	I	E	S M	D	S A	H	K	S

TGGTTCTTTGA TGAA CTGTCTCA TGA GCA GAA TCGCCA GTTCAAA TGTCCTGCCTCAA CAA GA TAAA GAA GA TCTGGAA TCCA TTA TGGA CA CA CTGA TGTCGGCAA TTAAA GGA GCGA GTGCCAAA GGTAAAA GCT G	S	L M	N	C	L M	S R	I	A	S	S	N V	L	P	Q	Q	D	K	E	D	L	E	S I	M	D	T	L M	S A	I	K	G A	S A	K	G	K	S

GAA CGCTTTCA TGAAA TGTTTGA TCCA GAA GA TAA GCA CA TCCCCTGTCTTTCCTCAA CAA GAAAAA GA GGA CA TGGAA GAAA TTGTGGA GA CCA TGA TGTCTGCTTTCA GTA GCA TGA GCA CA TCTGGGGGAA GCA N A	F M	K	C	L I	Q	K I	S	T	S	P V	F	P	Q	Q	E	K	E	D M	E	E I	V	E	T M	M	S A	F	S	S M	S	T	S	G	G	S

GAA CGCA TTCA TGAAA TGTTTAA TCCAAAA GA TA GGCA CA TCR CCTGTA TTCCCTCAA CAA GAAAAA GA GGA CA TGGAA GAAA TTGTGGA GA CCA TGA TGTCTGCTTTCA GTA GTA TGA GTA CA TCTGGGGGAA GCA N A	F M	K	C	L I	Q	K I	G	T	S	P V	F	P	Q	Q	E	K	E	D M	E	E I	V	E	T M	M	S A	F	S	S M	S	T	S	G	G	S

GGAA GCTTTTGCAA GA TCTTTCGTGA GCAA TGTTGTTTCTA GTGGA GAA TTTGGA GCCCAA GGA GCCGAA GA CTTCGA TGA CA TAA TTCAAA GTCTCA TA CA GGCC---CA GA GTA TGGGCAAA GGGCGGCA TGA TA E A	F A	R	S	F V	S	N V	V	S	S	G	E	F	G A	Q	G A	E	D	F	D	D I	I	Q	S	L I	Q A --- Q	S M	G	K	G R	H	D

GGAA GCTTTTGCA CGA TCTTTCGTGA GCAA TA TTGTTTCTA GTGGA GAA TTTGGA GCCCAA GGA GCCGAA GA CTTCGA TGA CA TAA TTCA GA GTCTCA TA CA GGCC---CA GA GCA TGGGCAAA GGGCGGCA TGA TA E A	F A	R	S	F V	S	N I	V	S	S	G	E	F	G A	Q	G A	E	D	F	D	D I	I	Q	S	L I	Q A --- Q	S M	G	K	G R	H	D

??????????????????????????????????????????????????????????????????????????????????????????????CTCA TA CA GGCC---CA GA GCA TGGGCAAA GGCCGGCA CGA CA

?	?	?	?	?	?	?	?	?	?	?	?	?	?	?	?	?	?	?	?	?	?	?	?	?	?	?	?	?	?	?	L I	Q A --- Q	S M	G	K	G R	H	D

AAA TAA CTTCGTTGA CTGTCTTAAA GGA GGAA TTCA GGCA TCCCCA GCTTTCCCA CGA CAA GAA CAA GCTGA TA TCCA GTCAA TTGCAA GTTCCA TA CTTTCA GCT------GGT------AA TA CTGCAA CAAAA T N	N	F V	D	C	L	K	G	G I	Q A	S	P A	F	P R	Q	E	Q A	D I	Q	S I	A	S	S I	L	S A ------ G ------ N	T A	T	K

GA GTGGTTTCCTTGGA TGCCTCA CAA CTGGTA TTGGA CTA TCTCCA GCTTTCCCGTTTCAA GAA CAA CAA GA TTTA GA TGA CTTA GCCAA GGTAA TTCTCTCCGCA GTAA CCA GT------AA TA CTGA CA CCTCAA S	G	F	L	G	C	L	T	T	G I	G	L	S	P A	F	P	F	Q	E	Q	Q	D	L	D	D	L A	K V	I	L	S A	V	T	S ------ N	T	D	T	S

AA CTA GTTTCCTAAA CTGTCTTA GA TCTAA TA TA GAA TCTTCTCCA GCA TTCCCA TTCCAA GAA CA GGCTGA CTTA GA TTCTA TCGCGGAA GTAA TTCTTTCA GA TGTA TCCA GTGTG---AA TA CC---GCAA GC- T	S	F	L	N	C	L R	S	N I	E	S	S	P A	F	P	F	Q	E	Q A	D	L	D	S I	A	E V	I	L	S	D V	S	S V --- N	T --- A	S -

CA GCGGCTTCCTTCAA TGTCTCA CA TTTGGAA TCGGAAA TTCCCCCGCA TTTCCTA CTCAA GAA CAA CAA GA CTTGGA TGCCA TTGCTCA GGTGA TA CTCAA TGCTGTTTCAA GC------AA CA CTGGCGCTA CA G S	G	F	L	Q	C	L	T	F	G I	G	N	S	P A	F	P	T	Q	E	Q	Q	D	L	D A	I	A	Q V	I	L	N A	V	S	S ------ N	T	G A	T

AA GCGGCTTCCTTCA GTGCCTCA CA TTTGGAA TCGGAAA TTCTCCCGCA TTTCCCA CTCAA GAA CAA CAA GA TTTGGA TGCCA TTGCTCAA GTGA TA CTCAA TGCCGTTTCAA CA ------AA TA CTGGCGCCA CA G S	G	F	L	Q	C	L	T	F	G I	G	N	S	P A	F	P	T	Q	E	Q	Q	D	L	D A	I	A	Q V	I	L	N A	V	S	T ------ N	T	G A	T

Dsfibroin1a

Frame 1

DsMaSp2

Frame 1

NcMaSp1a

Frame 1

NcMaSp1b

Frame 1

NcMaSp2

Frame 1

NiMaSp2

Frame 1

LgMaSp1

Frame 1

LheMaSp1

Frame 1

LheMaSp2

Frame 1

EaMaSp

Frame 1

AtMaSp2

Frame 1

AapMaSp

Frame 1

UdMiSp

Frame 1

MgMiSp

Frame 1

LheMiSp

Frame 1

AvMiSp

Frame 1

Bcfibroin1

Frame 1

DcMaSp_like

Frame 1

DcMaSp

Frame 1

AargAcsp1Full

Frame 1

LheAcSp1

Frame 1

LgAcSp1

Frame 1

NiFlag

Frame 1

NcFlag

Frame 1

AvFlag

Frame 1

AapTuSp1

Frame 1

NcTuSp1

Frame 1

LheTuSp1

Frame 1

AbTuSp1

Frame 1

AaTuSp1

Frame 1

280	290	300	310	320	330	340	350	360	370	380	390	400	410

CTGCA CA CAA GCTTCAA GCCA TGAA TA TGGCTTTCGCGTCA TCCA TGGCTGA GA TA GCTTCC------------CAAA GTAA CGA TA TCAA TCGCGCAAAA ---TA TGCTA TGGCA GAA GCAA TGGCA TGTGCTTTT T A	H	K	L	Q A	M	N M	A	F A	S	S M	A	E I	A	S ------------ Q	S	N	D I	N R	A	K --- Y A	M	A	E A	M	A	C A	F

CAAAA GCAAA GCTGCA GGCAA TGAA CA TGGGCTTCGCA TCCGCTA TA GCA GAAA TTGCTCTGGTA GAA ---------GGCGGTGCA CCGGCA GAA ---------AA CGCTGTA GTCAA CGCCA TGCAAA GCGCAA TT S	K A	K	L	Q A	M	N M	G	F A	S A	I	A	E I	A	L V	E --------- G	G A	P A	E --------- N A	V	V	N A	M	Q	S A	I

CAAAA GGAAA GTTGCAA GCTCTTAA CA TGGCA TTCGCTTCA TCAA TGGCGGAAA TTGCTGCA GTA GAA ------CAA GGA GGTTTAA GTGTA GA TGCAAA GA CAAA CGCTA TTGCA GA TTCA TTAAA CTCCGCA TTC S	K	G	K	L	Q A	L	N M	A	F A	S	S M	A	E I	A	A	V	E ------ Q	G	G	L	S V	D A	K	T	N A	I	A	D	S	L	N	S A	F

CA CAA TCAAA GCTGCAA GCTCTAAA CA TGGCCTTCGCTTCA TCAA TGGCGGAAA TTGCTGCA GTGGA G------CAA GGA GGTCTGA GTGTA GCA GAAAAAA CTAA CGCAA TTGCA GA TTCA TTAAA CTCA GCCTTC S	Q	S	K	L	Q A	L	N M	A	F A	S	S M	A	E I	A	A	V	E ------ Q	G	G	L	S V	A	E	K	T	N A	I	A	D	S	L	N	S A	F

CCCAA CA CAAA CTGCAA GCTCTCAA CA TGGCTTTCGCCTCTTCCA TGGCA GAAA TTGCA GCGGTGGA G------CAA GGTGGTA TGA GCA TGGCA GTAAAAA CAAA TGCAA TTGTTGA TGGCCTGAA TTCA GCTTTC S	Q	H	K	L	Q A	L	N M	A	F A	S	S M	A	E I	A	A	V	E ------ Q	G	G M	S M	A	V	K	T	N A	I	V	D	G	L	N	S A	F

CCAAA TCAAAA TTA CAA GCTCTAAA CA TGGCTTTCGCTTCA TCGA TGGCA GAAA TTGCA GCGGTGGAA ------CAA GGTGGTCA GA GCA TGGA TGTCAAAA CAAA TGCAA TTGCCAA TGCTCTA GA TTCA GCTTTT S	K	S	K	L	Q A	L	N M	A	F A	S	S M	A	E I	A	A	V	E ------ Q	G	G	Q	S M	D V	K	T	N A	I	A	N A	L	D	S A	F

CA CCCA CCAAA TTA CAA GCCTTA GA TA TGGCTTTCGCA TCA TCTGTGGCA GAAA TTGCTGCTGTGGAA ------------GGTCAAAA TA TA GGGGTAA CTA CAAA TGCAA TTTCA GA CGCCTTGA CA TCA GCTTTC T	P	T	K	L	Q A	L	D M	A	F A	S	S V	A	E I	A	A	V	E ------------ G	Q	N I	G V	T	T	N A	I	S	D A	L	T	S A	F

CGCCA TCCAAA TTA CA GGCTTTA GA TA TGGCTTTCGCA TCA TCTGTA GCA GAAA TTGCTGCTTCGGAA ------------GGA GGA GA CTTA GGA GTAA CAA CAAA TGCAA TTGCA GA TGCTTTAA CGTCA GCTTTC T	P	S	K	L	Q A	L	D M	A	F A	S	S V	A	E I	A	A	S	E ------------ G	G	D	L	G V	T	T	N A	I	A	D A	L	T	S A	F

CA CAA TCAAAA TTA CA GGCTTTA GA TA TGGCTTTTGCA TCA TCCGTGGCA GAAA TA GCTGTA GCTGA T------------GGCCAAAA CGTTGGA GCCGCTA CGAA TGCCA TA TCA GA CGCA TTA CGGTCA GCCTTC T	Q	S	K	L	Q A	L	D M	A	F A	S	S V	A	E I	A	V	A	D ------------ G	Q	N V	G A	A	T	N A	I	S	D A	L R	S A	F

CA CCGAA TAA GCTGCA GGCCCTTAA CA TGGCTTTTGCA TCTTCGA TGGCA GAAA TCGCGGCA TCCGAA ------GAA GGA GGGGGAA GCCTTTCCA CCAAAA CTA GCTCTA TA GCCA GTGCAA TGTCCAA CGCGTTT S	P	N	K	L	Q A	L	N M	A	F A	S	S M	A	E I	A	A	S	E ------ E	G	G	G	S	L	S	T	K	T	S	S I	A	S A	M	S	N A	F

CTAAA TCGAA GTTGCA GGCA TTAAA CA TGGCA TTTGCTTCCTCAA TGGCCGAAA TTGCTGTA GCA GA G------CA GGGA GGTTTAA GCTTA GAA GCAAAAA CCAA TGCCA TCGCAA GTGCCCTCA GTGCA GCCTTT S	K	S	K	L	Q A	L	N M	A	F A	S	S M	A	E I	A	V	A	E ------ Q	G	G	L	S	L	E A	K	T	N A	I	A	S A	L	S A	A	F

CCA GTCA TA TGTTAAA GGCAA TGAA CA TGGCAA TGGGAA CTTCCA TA GCA GAAA TT---GTCGCTGA T---------GGA GGA GGCAA TCTTGGTA GCAAA GTGA GTTGCA TA TCAAA TGCCTTA TCGTCA GCGTTT S	S	H M	L	K A	M	N M	A	M	G	T	S I	A	E I --- V	A	D --------- G	G	G	N	L	G	S	K V	S	C I	S	N A	L	S	S A	F

GCCGTGGCGCGA TGA GGGCCA TGAA CGCA GCGTTCGGCTCA GCTA TTGCA GAAA TCGTTGCTGCTAA C---------GGCGGAAAA GAA TA CCAA ------A TA GGCGCA GTTCTA GA TGCA GTTA CTAA TA CTCTT G R	G A	M	R	A	M	N A	A	F	G	S A	I	A	E I	V	A	A	N --------- G	G	K	E	Y	Q ------ I	G A	V	L	D A	V	T	N	T	L

--GA TCA CA GA CTTAA GGGA GTGA CTA CCA TGA TGGCCGCCGGAA TA GCCGA T---GCGGCTGTAAA T------TCA CCA GGTCA GA GTCTTGA TGTCA TGA TTAA TA CCA TCA GCGGA TGCA TGA CGCAA GCAA TG

-- D	H R	L	K	G V	T	T M	M	A	A	G I	A	D --- A	A	V	N ------ S	P	G	Q	S	L	D V	M	I	N	T I	S	G	C M	T	Q A	M

--AAA TCGAAA CTGCAA GCTTTAAA CA TGGCTTTTGCA TCA TCA GTTTCGGAAA TTGCGTTCTCTGA G------AA CAA TGGAA TTTCAAA CA GTGCTAAAA TA CAA GCCA TTA TTGA TGCA CTCA GA GGA GCA TTT

-- K	S	K	L	Q A	L	N M	A	F A	S	S V	S	E I	A	F	S	E ------ N	N	G I	S	N	S A	K I	Q A	I	I	D A	L R	G A	F

--CAA CA CA GA CTCA GGGCAA TGAA TA CCGCCA TGGCCGCA GAA GTA GCTGAA ---GTA GTA GCAA CT------TCGCCA CCA CAAA GTTA TTCTGCA GTTTTAAA TA CCA TTGGTGCTTGCTTGA GGGAA TCAA TG

-- Q	H R	L R	A	M	N	T A	M	A	A	E V	A	E --- V	V	A	T ------ S	P	P	Q	S	Y	S A	V	L	N	T I	G A	C	L R	E	S M

CTGCA GCTAA GA TGAA GGCGCTAAA CA TGGCA TTTGCTTCA TCCA TA GCTGAAA TA GCTGTA CA GGA G------GGA GGA GCA GGTTCAA TCGAA GAAAAAA CCCAA GCA GTTTCA GAA GCTCTA GCA CA TGCTTTC A	A	A	K M	K A	L	N M	A	F A	S	S I	A	E I	A	V	Q	E ------ G	G A	G	S I	E	E	K	T	Q A	V	S	E A	L A	H A	F

CA TA TGCGA GCCTGCAA GCCA TGAA CA CGGCA TTCGCTTCGTCCA TGGCTCAA CTGTTTGCAA CCGAA ------CA GGA CTA CGTGGA C------------A CA GAA GTAA TCGCCGGA GCCA TTGGAAAA GCTTA C S	Y A	S	L	Q A	M	N	T A	F A	S	S M	A	Q	L	F A	T	E ------ Q	D	Y V	D ------------ T	E V	I	A	G A	I	G	K A	Y

CA GCCGCGAA GCTGCAA GCCA TGAA CA TGGCA TTCGCA TCTGCGA TTGCTGA CA TA GCGGCAA CCGAA ------GCA TA CGGA GCGGA CA TTTCA TTGGAAA CGTCA GCAA TCGCCAA TGCTCTCTCTGAA GCTTTC S A	A	K	L	Q A	M	N M	A	F A	S A	I	A	D I	A	A	T	E ------ A	Y	G A	D I	S	L	E	T	S A	I	A	N A	L	S	E A	F

CTGCA GCGCA GTTGCA GGCGA TAAA TA TGGCTGTTGCTTCTTCCCTGGCA GAAA TA GTCGTTGCTGAA GA TGCA GGCAA TCAA GCAA GCA TCGCA GTAAA GA CTCA GGCCCTTA CA GGTGCTCTGGGA CAA TGCTTC S A	A	Q	L	Q A	I	N M	A	V	A	S	S	L A	E I	V	V	A	E	D A	G	N	Q A	S I	A	V	K	T	Q A	L	T	G A	L	G	Q	C	F

A TGCGGCAAAA CTA CAA GCCA TGAA TA TGGCA TTTGCTTCA TCTA TGGCGGAA TTA GTTA TTGCCGAA GA TGCTGA TAA CCCA GA CA GTA TA TCCA TTAAAA CA GAA GCTCTGGCAAA GTCTCTTCAA CA GTGCTTC N A	A	K	L	Q A	M	N M	A	F A	S	S M	A	E	L V	I	A	E	D A	D	N	P	D	S I	S I	K	T	E A	L A	K	S	L	Q	Q	C	F

A TGCGGCAAAA CTA CAA GCCA TGAA CA TGGCA TTCGCTTCA TCTA TGGCGGAA TTA GTTA TTGCCGAA GA TGCA GA TAA CCCA GA CA GTA TTTCCA TTAAAA CTGAA GCTCTGGCGAA GTCTCTTCAA CA GTGTTTC N A	A	K	L	Q A	M	N M	A	F A	S	S M	A	E	L V	I	A	E	D A	D	N	P	D	S I	S I	K	T	E A	L A	K	S	L	Q	Q	C	F

CGAA GGCCAA GGCGAAA GCGA TGCA GGTA GCCCTTGCTTCTTCTA TA GCCGAA TTGGTGA TA GCA GAA ------A GCA GCGGA GGCGA TGTGCAA CGCAAAA CCAA TGTTA TCTCCAA CGCTTTGA GAAA CGCCTTG T	K A	K A	K A	M	Q V	A	L A	S	S I	A	E	L V	I	A	E ------ S	S	G	G	D V	Q R	K	T	N V	I	S	N A	L R	N A	L

CGAA GGCCAA GGCGAAA GCGA TGCA GGTA GCCCTTGCTTCTTCTA TA GCCGAA TTGGTTA TTGCA GAA ------A GCA GCGGA GGCGA TGTGCAA CGCAAAA CCAA CGTTA TCTCCAA CGCTTTGA GAAA CGCCTTG T	K A	K A	K A	M	Q V	A	L A	S	S I	A	E	L V	I	A	E ------ S	S	G	G	D V	Q R	K	T	N V	I	S	N A	L R	N A	L

CCAA CGCGAAA GCCAAA GCCA TGCA GA TGGCCCTGGCCTCCTCTA TCGCCGA GCTGA TA GTGGAA GAA ------A GCGGA GGGGTGAA TA TGCA GCA GAA GA CCAA CGCGGCGA TCAA TGCCTTGA GGAA CGCCTTG T	N A	K A	K A	M	Q M	A	L A	S	S I	A	E	L I	V	E	E ------ S	G	G V	N M	Q	Q	K	T	N A	A	I	N A	L R	N A	L

CGAAA GCAA TA GAA CAA GCTTTAA GCA CA GCTTTA GCTTCCTCTCTCGCGGAAA TTGTAA TTA CA GAA ------TCA GGGGGGCA GGA TTA TTCTAAA CAAA TAA CA GA TTTAAA TGGAA TCCTA TCAAA CTGCTTT S	K A	I	E	Q A	L	S	T A	L A	S	S	L A	E I	V	I	T	E ------ S	G	G	Q	D	Y	S	K	Q I	T	D	L	N	G I	L	S	N	C	F

A GTCA GCGA GA GCA CAA GCCTTGA GCA CTGCA TTA GCA TCTTCCTTA GCCGA CCTA CTGA TA TCCGAA ------TCAA GTGGAA GCA GCTA CCAAA CTCAAA TA TCTGCCCTCA CTAA TA TCCTA TCCGA TTGTTTT K	S A	R	A	Q A	L	S	T A	L A	S	S	L A	D	L	L I	S	E ------ S	S	G	S	S	Y	Q	T	Q I	S A	L	T	N I	L	S	D	C	F

--TCA GCAA CA TCTTTA GCGCTAA GTA CTGCTTTA GCA TCGTCGTTGGCTGAA CTA CTTGTCA CTGAA ------TCA GCTGAA GAA GA TA TTGA TAA TCA GGTA GTA GCTTTGTCAA CAA TTCTTTCTCA GTGTTTC

-- S A	T	S	L A	L	S	T A	L A	S	S	L A	E	L	L V	T	E ------ S A	E	E	D I	D	N	Q V	V	A	L	S	T I	L	S	Q	C	F

CA TCGGCCA GA GCTCAA GCTTTAA GTA CA GCGCTTGCA TCTTCTCTGA CA GA TCTGCTCA TTGCA GA G------TCGGCA GAAA GCAA TTA CA GCAA TCA GTTGTCTGAA CTAA CA GGAA TTCTCTCCGA CTGTTTT A	S A	R	A	Q A	L	S	T A	L A	S	S	L	T	D	L	L I	A	E ------ S A	E	S	N	Y	S	N	Q	L	S	E	L	T	G I	L	S	D	C	F

CA TCA GCCA GA GCTCAA GCTTTAA GTA CA GCGCTCGCA TCCTCTCTCA CA GA TCTCCTCA TTGCA GA G------TCGGCTGA GA GCAA CTA CAA TAA TCA GCTTTCTGAA CTAA CA GGAA TA CTTTCCAA CTGTTTT A	S A	R	A	Q A	L	S	T A	L A	S	S	L	T	D	L	L I	A	E ------ S A	E	S	N	Y	N	N	Q	L	S	E	L	T	G I	L	S	N	C	F

Dsfibroin1a

Frame 1

DsMaSp2

Frame 1

NcMaSp1a

Frame 1

NcMaSp1b

Frame 1

NcMaSp2

Frame 1

NiMaSp2

Frame 1

LgMaSp1

Frame 1

LheMaSp1

Frame 1

LheMaSp2

Frame 1

EaMaSp

Frame 1

AtMaSp2

Frame 1

AapMaSp

Frame 1

UdMiSp

Frame 1

MgMiSp

Frame 1

LheMiSp

Frame 1

AvMiSp

Frame 1

Bcfibroin1

Frame 1

DcMaSp_like

Frame 1

DcMaSp

Frame 1

AargAcsp1Full

Frame 1

LheAcSp1

Frame 1

LgAcSp1

Frame 1

NiFlag

Frame 1

NcFlag

Frame 1

AvFlag

Frame 1

AapTuSp1

Frame 1

NcTuSp1

Frame 1

LheTuSp1

Frame 1

AbTuSp1

Frame 1

AaTuSp1

Frame 1

420	430	440	450	460	470	480	490	500	510	520	530	540

CGAAA CTCA TTA GGA TA CGTGGA CGGAAA CTTCGTA CA GGA GGTAA TA GGCA TGGTCGAAA TGTTCA GCCAA GCG---------AA TA TCAA TGA GA TTTCTGTA CTGGCTTCTCCA CAA TCTGTTA CA CGCGTCA G R	N	S	L	G	Y V	D	G	N	F V	Q	E V	I	G M	V	E M	F	S	Q A --------- N I	N	E I	S V	L A	S	P	Q	S V	T R	V	S

TGGCA GA GCA CA GGTTCA CCAAA CA GA TCA TTCA TAAA CGAAA TGAAA GCA TTAA TGAAAA TGCTTGCCCAA ------------CA GTCAAA TTTCGTA TCAA GGA TGA GTA CTCCTGGTTCA GGTTCTA GAA TTTC W	Q	S	T	G	S	P	N R	S	F I	N	E M	K A	L M	K M	L A	Q ------------ Q	S	N	F V	S R	M	S	T	P	G	S	G	S R	I	S

TA CCA GA CAA CTGGA GCA GCCAA CCCA CA GTTTGTCAA TGAAA TCA GAA GCTTAA TTAA CA TGTTTGCCCAA TCT---------TCTGCAAA TGAA GTA TCCCGCTTGTCCTCA CCTGAA GCTA GTTCAA GA GTTTC Y	Q	T	T	G A	A	N	P	Q	F V	N	E I	R	S	L I	N M	F A	Q	S --------- S A	N	E V	S R	L	S	S	P	E A	S	S R	V	S

TA CCAAA CCA CA GGA GCA GTCAA CGTTCA GTTTGTCAA TGAAA TCA GAA GTTTAA TTA GCA TGTTTGCA CAA GCC---------TCTGCAAA TGAA GTA TCCCGTTTGTCTTCTCCTCAA GCTA GTTCAA GA GTTTC Y	Q	T	T	G A	V	N V	Q	F V	N	E I	R	S	L I	S M	F A	Q A --------- S A	N	E V	S R	L	S	S	P	Q A	S	S R	V	S

TA CA TGA CAA CTGGA GCTGCAAA TCCTCA GTTTGTCAA CGAAA TGA GGA GCTTAA TTA GTA TGA TAA GTGCA GCT---------TCGGCTAA TGAA GTA TCTCGTCTGGCTTCTCCA GA TTCA GGCGCTA GA GTTGC Y M	T	T	G A	A	N	P	Q	F V	N	E M	R	S	L I	S M	I	S A	A --------- S A	N	E V	S R	L A	S	P	D	S	G A	R	V	A

TA TA TGA CAA CTGGTTCAA CAAA TCAA CA GTTTGTCAA CGAAA TGA GAA GCTTAA TTAA CA TGTTGA GTGCA GCT---------GCCGTTAA TGAA GTA TCTCGA CTTGCTTCTCCA GA TTCA GGCGCTA GA GTTGC Y M	T	T	G	S	T	N	Q	Q	F V	N	E M	R	S	L I	N M	L	S A	A --------- A	V	N	E V	S R	L A	S	P	D	S	G A	R	V	A

TA TCAAA CAA CA GGGGTA GTTAA TAA CAAA TTTA TCA GCGAAA TTA GAA GTTTGA TTAA TA TGTTTGCA CAA GCG---------TCTGCAAA TGA TGTTTCTGCTTTGGCA GCTCCA GCTA CAA GTGCAA GAA TTTC Y	Q	T	T	G V	V	N	N	K	F I	S	E I	R	S	L I	N M	F A	Q A --------- S A	N	D V	S A	L A	A	P A	T	S A	R	I	S

TA TCAAA CAA CCGGA GTA GTTAA TA GCA GA TTTA TAA GCGAAA TTA GAA GTTTGA TTGGCA TGTTTGCA CA GGCA ---------TCTGCCAA CGA TGTA TCTGCTTTGGCA GCTCCA GCTA CAA GCGCGA GAA TTTC Y	Q	T	T	G V	V	N	S R	F I	S	E I	R	S	L I	G M	F A	Q A --------- S A	N	D V	S A	L A	A	P A	T	S A	R	I	S

TA TCAAA CTA CCGGA GTGGTAAA CAA TCAA TTTA TTA CTGGGA TAA GTA GCCTAA TTGGCA TGTTTGCCCAA GTA ---------TCA GGCAA TGAA GTTTCA GCTTTA TCTTCA CCGA CAA CGCA TGCTA GAA TTTC Y	Q	T	T	G V	V	N	N	Q	F I	T	G I	S	S	L I	G M	F A	Q V --------- S	G	N	E V	S A	L	S	S	P	T	T	H A	R	I	S

CTGCAAA CAA CTGGA GTGGTAAA CCAA CCGTTCA TAAA TGAAA TAA CTCA GCTCGTTA GCA TGTTTGCTCAA GCA ---------GGTA TGAA TGA TGTCA GTCGCCTCTCA TCGCCTTCCGCA GTA TCTCGA GTTTC L	Q	T	T	G V	V	N	Q	P	F I	N	E I	T	Q	L V	S M	F A	Q A --------- G M	N	D V	S R	L	S	S	P	S A	V	S R	V	S

TTGGAAA CCA CTGGCTA TGTAAA CCAA CA GTTTGTCAA TGAAA TAAAAA CA TTAA TA TTTA TGA TCGCTCA GGCA ---------TCA TCAAA TGAAA TTTCTCGA CTTTCTTCTCCTCAA GCCA GTTCTCGA GTTTC L	E	T	T	G	Y V	N	Q	Q	F V	N	E I	K	T	L I	F M	I	A	Q A --------- S	S	N	E I	S R	L	S	S	P	Q A	S	S R	V	S

CTA CAAA CCA CTGGTTCA GTAAA TA CA CA GTTTGTAAA TGAAA TTGTA TCTCTCA TCA GTA TGTTCGCTCAA GCA ---------GA TA CAAA CGAA GTCTCTCGA CTTTCA TCA CCTTCA TCAA GTTCTA GA GTA TC L	Q	T	T	G	S V	N	T	Q	F V	N	E I	V	S	L I	S M	F A	Q A --------- D	T	N	E V	S R	L	S	S	P	S	S	S	S R	V	S

CTA CA GCTGA CCGGAAA TGCTGA TAA CGGTTTTCTCAA TGAAA TCA GTCGA CTTA TCA CGCTA TTTA GCA GTGTA ---------GAA GCAAA CGA CGTAAA CCGAA TTGTA TCTGCTCCA GCTGTCAA CCGAA TGA G L	Q	L	T	G	N A	D	N	G	F	L	N	E I	S R	L I	T	L	F	S	S V --------- E A	N	D V	N R	I	V	S A	P A	V	N R	M	S

TCTCAA GCGGTA GGTTA CGTCGA CCA GA CA TTAA TAA GA GAA GTGGCA GAAA TGGTAAA CA TGCTTGCAAA CGAA ---------AA TGCGAA TGCA GTAAA TCGA TTGTCTA GTGCCGAA GCA GCAA GCA GA GTA TC S	Q A	V	G	Y V	D	Q	T	L I	R	E V	A	E M	V	N M	L A	N	E --------- N A	N A	V	N R	L	S	S A	E A	A	S R	V	S

CTGCAAA CTA TTGGAA CGGTTGA TCAAA CCTTTTTGAA TGAAA TTTCTA GTCTCGTTAAAA TGTTTTCA CA GGTTTCA ------GCA GAAAA TGCCGTTTCA CGA TTAA GTTCTCCTTCTTCCTCGTCTA GAA TAA G L	Q	T I	G	T V	D	Q	T	F	L	N	E I	S	S	L V	K M	F	S	Q V	S ------ A	E	N A	V	S R	L	S	S	P	S	S	S	S R	I	S

A TGCAA GCGA CA GGCTCCGTCGA CAA TGCA TTCA CAAA TGAA GTAA TGCAA TTGGTAAAAA TGTTA TCTGCGGA T---------A GCGCGAA TGAA GTAAA TCGCTTGTCTA GTGCTGAA GCTGCCA GTA GA GTA TC M	Q A	T	G	S V	D	N A	F	T	N	E V	M	Q	L V	K M	L	S A	D --------- S A	N	E V	N R	L	S	S A	E A	A	S R	V	S

CTTCAAA CTA CA GGTTCCGTAAA TA TA CAA TTCA TCAAA GAAA TCA GA GCCCTCA TAA CCCTTTTCGCTAAA GAA GGA ------CAA GA CAA CGAAA CAA GCGGTTTGA CTTCA GA GGCTGCAAA GGAAA GGA TTTC L	Q	T	T	G	S V	N I	Q	F I	K	E I	R	A	L I	T	L	F A	K	E	G ------ Q	D	N	E	T	S	G	L	T	S	E A	A	K	E R	I	S

CA GCA GA TTA CCGGA TA CGAAAA TCCA CA CCTA GCTTCCGA GGTCA CGA GGTTGA TCCAA CTTTTCCGCGAA GAA GA TGA C---CTA GAAAA CGAA GTA GCTGGTA GTGGTGAA CTGTCCGTTGCTTCAA GAA TA TC Q	Q I	T	G	Y	E	N	P	H	L A	S	E V	T R	L I	Q	L	F R	E	E	D	D --- L	E	N	E V	A	G	S	G	E	L	S V	A	S R	I	S

CTGCA GA CAA CCGGCGTA GTGAA CAA GCGTTTCA TCA GTGA GA TA CA GGA GCTGA TTTA CA TGTTCGCA CA GGA CGCCTCGGTGCAAA GCAA CGAAA TCGCA CTTCTCGTCTCTGCTGA TGCA CCTTCAA GAA TTGC L	Q	T	T	G V	V	N	K R	F I	S	E I	Q	E	L I	Y M	F A	Q	D A	S V	Q	S	N	E I	A	L	L V	S A	D A	P	S R	I	A

CAA GCA GTCA TGGGCA CA GTCGA CA GGAAA TTCA TCAA TGAAA TAAA TGA CTTGA TAA CAA TGTTTGCAAAA GAA GCTGCCA GTGAAA GCAA CGAAA TA GTTGGA TTAA GA TCTGGTTCTGCTGCTTCTA GAA TCA G Q A	V	M	G	T V	D R	K	F I	N	E I	N	D	L I	T M	F A	K	E A	A	S	E	S	N	E I	V	G	L R	S	G	S A	A	S R	I	R

AAA TCAA CA TTGGGCTCA GTGAA CA GGCA TTTCA TTGCA GA GA TTAAA GA TCTGA TTGGAA TGTTTGCCA GA GAA GCTGCA ---GCTA TGGAA GA GGCCCAA GGTTTAAAA TCTCCTCAA GCAA GTTCCA GGA TCAA K	S	T	L	G	S V	N R	H	F I	A	E I	K	D	L I	G M	F A	R	E A	A --- A	M	E	E A	Q	G	L	K	S	P	Q A	S	S R	I	N

AAA TCAA CA TTGGGTTCA GTGAA TA GGCA TTTCA TTGCTGA GA TTAAA GA TCTGA TTGGAA TGTTTGCCA GA GAA GCTGCC---GCTA TGGAA GA TA CTCAA GGTCTGAAR TCTCCTSAA GCAA GTTCGA GGA TTAA K	S	T	L	G	S V	N R	H	F I	A	E I	K	D	L I	G M	F A	R	E A	A --- A	M	E	D	T	Q	G	L	K	S	P Q/E A	S	S R	I	N

A TGTCTA CAA CTGGAA GCCCGAA CGAA GA GTTCGTCCA TGAA GTTCAA GA CCTCA TCCA GA TGTTA TCTCAA GAA ---------CA GA TCAA CGA GGTA ---------------------------TCGCGTGTTCC M	S	T	T	G	S	P	N	E	E	F V	H	E V	Q	D	L I	Q M	L	S	Q	E --------- Q I	N	E V --------------------------- S R	V	P

A TGTCTA CAA CA GGCA GCCCAAA CGAA GA GTTCGTCCA TGAA GTTCAA GA CCTCA TCCA GA TGTTA TCTCAA GAA ---------CA GA TCAA CGA GGTA ---------------------------TCGCGTGTTCC M	S	T	T	G	S	P	N	E	E	F V	H	E V	Q	D	L I	Q M	L	S	Q	E --------- Q I	N	E V --------------------------- S R	V	P

A GA TCCA CTTCTGGA CAA GTGGA TGA GGGCTTTGTCA GCGAAA TCGTTGAA CTCGTCAA CCTA TTCTCTCAA GA G---------CA GTTTAA CGA GGTG---------------------------TCTA GA CTTCC R	S	T	S	G	Q V	D	E	G	F V	S	E I	V	E	L V	N	L	F	S	Q	E --------- Q	F	N	E V --------------------------- S R	L	P

A TCCAAA CAA CA GGA GTA GAAAA CAAAA GA TTCGTAAA CA GCA TA CAAAA TCTCA TAA GA CTGCTTGCA GAAA GC---------GCA GTCTCA GAAA CAA CTGGA CTTTCTTCTGCTTCTGCA TCTTCTCGTGTAAA I	Q	T	T	G V	E	N	K R	F V	N	S I	Q	N	L I	R	L	L A	E	S --------- A	V	S	E	T	T	G	L	S	S A	S A	S	S R	V	N

GTCA CAA CAA CTGGA TCAAA CAA TCCTGCA TTTGTA TCAA GA GTTCAAA CA CTTA TA GGA GTGCTTTCTCAAA GC---------A GCA GTAA TGCAA TTTCCGGCTTA TCA TCA GCGA GTGCGA GTGCAA GA GTTGG V	T	T	T	G	S	N	N	P A	F V	S R	V	Q	T	L I	G V	L	S	Q	S --------- S	S	N A	I	S	G	L	S	S A	S A	S A	R	V	G

GTA GAAA CCA CA GGA TCTCCCAA CCCA GCGTTTGTA GCAA GTGTAAAA TCGCTA CTTGGA GTA TTA TCA CA GTCT---------GCAA GCAA TTA TGAA GCTGGA TTGGCTTCTA CTGCA GCAA CTTCAA GAA TTAA V	E	T	T	G	S	P	N	P A	F V	A	S V	K	S	L	L	G V	L	S	Q	S --------- A	S	N	Y	E A	G	L A	S	T A	A	T	S R	I	N

A TCCAAA CTA CTGGA TCGGA CAA CCCA GCA TTTGTGTCCA GAA TTCAA TCTCTCA TTTCA GTGCTTTCCCA GAA T---------GCA GA TA CAAA TA TA TCA GGCTTA GGCTCA TCTGCTGCCA CTGCCA GGGTGA G I	Q	T	T	G	S	D	N	P A	F V	S R	I	Q	S	L I	S V	L	S	Q	N --------- A	D	T	N I	S	G	L	G	S	S A	A	T A	R	V	S

A TCCAAA CCA CTGGA TCGGA TAA TCCA GCA TTTGTA TCCA GAA TTCA GTCGCTCA TA TCA GTGCTTTCTCA GAA T---------A CTGA TGTAAA TA TA TCTGGCTTA GGTTCCTCTGCTGCCTCTGCCA GA GTGA G I	Q	T	T	G	S	D	N	P A	F V	S R	I	Q	S	L I	S V	L	S	Q	N --------- T	D V	N I	S	G	L	G	S	S A	A	S A	R	V	S

Dsfibroin1a

Frame 1

DsMaSp2

Frame 1

NcMaSp1a

Frame 1

NcMaSp1b

Frame 1

NcMaSp2

Frame 1

NiMaSp2

Frame 1

LgMaSp1

Frame 1

LheMaSp1

Frame 1

LheMaSp2

Frame 1

EaMaSp

Frame 1

AtMaSp2

Frame 1

AapMaSp

Frame 1

UdMiSp

Frame 1

MgMiSp

Frame 1

LheMiSp

Frame 1

AvMiSp

Frame 1

Bcfibroin1

Frame 1

DcMaSp_like

Frame 1

DcMaSp

Frame 1

AargAcsp1Full

Frame 1

LheAcSp1

Frame 1

LgAcSp1

Frame 1

NiFlag

Frame 1

NcFlag

Frame 1

AvFlag

Frame 1

AapTuSp1

Frame 1

NcTuSp1

Frame 1

LheTuSp1

Frame 1

AbTuSp1

Frame 1

AaTuSp1

Frame 1

550	560	570	580	590	600	610	620	630	640	650	660	670	680

TGCTTTA GTA CCAAA TTTG---------A TA TCTGGCGGAA GTTTTAA CTCA ------AA TGCTGTTTCTGCTGTA CTGCCTGGCCTTA CTTCCCA GA TTAAA GCA GCAA CA ---GGA TCTAA CGGA TGTGA TTCTT A	L V	P	N	L --------- I	S	G	G	S	F	N	S ------ N A	V	S A	V	L	P	G	L	T	S	Q I	K A	A	T --- G	S	N	G	C	D	S

TAA CGCTGTGTCAAA CA TT---------TTA TCCTCTGGTGTTA GTTCGTCC------TCTGGTCTGTCAAA CGCTA TTTCAAA TA TA TCTTCCTCGA TTA GTGCTTCAAA TCCTGGTCTTTCA GGGTGTGA CGTTC N A	V	S	N I --------- L	S	S	G V	S	S	S ------ S	G	L	S	N A	I	S	N I	S	S	S I	S A	S	N	P	G	L	S	G	C	D V

A TCA GCTGTTTCTAA CTTG---------GTTTCAA GTGGTCCTA CTAA TTCT------GCGGCA CTTTCAA GTA CCA TCA GTAA TGTTGTTTCA CAAA TTGGCGCCA GCAA TCCTGGTCTTTCTGGA TGCGA TGTTC S A	V	S	N	L --------- V	S	S	G	P	T	N	S ------ A	A	L	S	S	T I	S	N V	V	S	Q I	G A	S	N	P	G	L	S	G	C	D V

A TCA GCTGTTTCCAA CTTG---------GTTGCAA GTGGTCCTA CTAA TTCT------GCGGCCTTGTCAA GTA CAA TCA GTAA CGTGGTTTCA CAAA TA GGCGCCA GCAA TCCTGGTCTTTCTGGA TGTGA TGTCC S A	V	S	N	L --------- V	A	S	G	P	T	N	S ------ A	A	L	S	S	T I	S	N V	V	S	Q I	G A	S	N	P	G	L	S	G	C	D V

A TCA GCTGTTTCTAA CTTG---------GTA TCCA GTGGCCCAA CTA GCTCT------GCTGCCTTA TCAA GTGTTA TCA GTAA CGCTGTGTCTCAAA TTGGCGCAA GTAA TCCTGGTCTCTCTGGTTGCGA TGTCC S A	V	S	N	L --------- V	S	S	G	P	T	S	S ------ A	A	L	S	S V	I	S	N A	V	S	Q I	G A	S	N	P	G	L	S	G	C	D V

A TCTGCTGTTTCTAA CTTG---------GTA TCCA GTGGTCCAA CTA GCTCT------GCTGCCTTA TCAA GCGTCA TCA GTAA CGCTGTGTCTCAAA TTGGA GCCA GTAA TCCTGGTCTCTCTGGTTGCGA TGTCC S A	V	S	N	L --------- V	S	S	G	P	T	S	S ------ A	A	L	S	S V	I	S	N A	V	S	Q I	G A	S	N	P	G	L	S	G	C	D V

TTCA CA TGCCTTAA CA CTT---------CTTTCCAA TGGGCCAA CTAA TCCT------GCTTCTA TTTCAAA TGTAA TTA GTAA TGCTGTTTCCCAAA TTA GTTCAA GCAA TCCGGGA TA TTCTTCA TGTGA CA TTC S	H A	L	T	L --------- L	S	N	G	P	T	N	P ------ A	S I	S	N V	I	S	N A	V	S	Q I	S	S	S	N	P	G	Y	S	S	C	D I

TTCA CA CGCCTCA GCTCTT---------CTTTCAAA TGGA CCTA CTAA CCCT------GCTTCTA TTTCAAA CGTTA TTA GTAA TGCTGTA TCCCAAA TTA GTTCCA GCAA TCCA GGA GCGTCTGCGTGTGA TGTTC S	H A	S A	L --------- L	S	N	G	P	T	N	P ------ A	S I	S	N V	I	S	N A	V	S	Q I	S	S	S	N	P	G A	S A	C	D V

TTCCCA TGCA TCAA CTCTA ---------TTA TCAA GTGGGCCAA CTAA TGCG------GCA GCTCTTTCTAA TGTCA TTA GTAA TGCCGTTTCCCAA GTCA GTGCAA GTAA TCCA GGA TCTTCCTCTTGTGA TGTCC S	H A	S	T	L --------- L	S	S	G	P	T	N A ------ A	A	L	S	N V	I	S	N A	V	S	Q V	S A	S	N	P	G	S	S	S	C	D V

TTCA GCA GTTTCTA GCTTG---------GTTTCAAA TGGTCAA GTGAA TA TG------GCA GCGTTA CCTAA TA TCA TTTCCAA CA TTTCTTCTTCTGTCA GTGCA TCTGCTCCTGGTGCTTCTGGA TGTGA GGTCA S A	V	S	S	L --------- V	S	N	G	Q V	N M ------ A	A	L	P	N I	I	S	N I	S	S	S V	S A	S A	P	G A	S	G	C	E V

A TCA GCTGTGTCAA CTTTG---------GTGTCGA GCGGTCCTA CGAA TCCT------GCCTCA CTCTCTAA TGCTA TCA GTA GCGTTGTA TCA CAA GTCA GTGCAA GTAA TCCTGGTCTTTCTGGTTGTGA TGTA C S A	V	S	T	L --------- V	S	S	G	P	T	N	P ------ A	S	L	S	N A	I	S	S V	V	S	Q V	S A	S	N	P	G	L	S	G	C	D V

CTCTGCCGTTTCTGGA TTA ---------TTA CCTAA TGGTAA CTTTAA CTTA ------GGAAA CTTGCCCGGCA TTGTA TCAAA TCTTTCGTCTTCAA TT---GCTTCTTCT---GGTCTCTCCGGCTGTGAAAA TT S A	V	S	G	L --------- L	P	N	G	N	F	N	L ------ G	N	L	P	G I	V	S	N	L	S	S	S I --- A	S	S --- G	L	S	G	C	E	N

CGCA GCA TCTTCAA CTCTT---------GTTTCTAA CGGCGCTTTTAA CGTC------GGTGCTCTTGGTTCAA CGA TTTCA GA TA TGGCTGCA CA GA TCCAA GCTGGTTCA CAA GGA CTTTCCA GTGCA GAA GCAA A	A	S	S	T	L --------- V	S	N	G A	F	N V ------ G A	L	G	S	T I	S	D M	A	A	Q I	Q A	G	S	Q	G	L	S	S A	E A

CTCTAA TGTTGCA GCTCTC---------GCCTCCGGTGGCCCT---------------GCTGCCCTTGCCAA TGTAA TGGGAAA TA TCTA CTCA GGTGTT---GCTTCTTCT---GGTGTTTCTTCTGGTGAA GCTC S	N V	A	A	L --------- A	S	G	G	P --------------- A	A	L A	N V	M	G	N I	Y	S	G V --- A	S	S --- G V	S	S	G	E A

CTCTGCA GCGTCAA GCCTT---------GCAA CTGGTGGA GTGTTGAA TTCT------GCTGCA TTGCCTTCGGTA GTTTCTAA TA TGA TGTCCCAA GTTTCTGCTA GCA GTCCTGGAA TGTCA TCAA GTGAA GTTG S A	A	S	S	L --------- A	T	G	G V	L	N	S ------ A	A	L	P	S V	V	S	N M	M	S	Q V	S A	S	S	P	G M	S	S	S	E V

CTCTAA TA TTGCA GCTA TT---------GCA TCTGGTGGTGCT---------------TCCGCCCTCCCCA GTGTTA TTTCAAA TA TTTA CTCA GGTGTCGTTGCTTCT------GGTGTTTCTTCTAA TGAA GCTC S	N I	A	A	I --------- A	S	G	G A --------------- S A	L	P	S V	I	S	N I	Y	S	G V	V	A	S ------ G V	S	S	N	E A

TTCGA TCA TA GCA TCA CTGTTA TCA GCAAAA TCTTCCCA GGA TTTTAA TGCC------TTA CTTCTCTCAAA CA TTCTCCCTTCCTTAA TA TCTAAAA TTTCGCA GCGTGCGTCA GGA TTA TCCCCAA CA GAAA TGG S I	I	A	S	L	L	S A	K	S	S	Q	D	F	N A ------ L	L	L	S	N I	L	P	S	L I	S	K I	S	Q R	A	S	G	L	S	P	T	E M

CA CCA TGGCA TCA TCTA TGA GTTCCCTGTTGTCA TCCAA CTTTTCTCCA GTTA TCTTTTCTTCTCTTGCTAA CGTGA TA GCCAA TGGA GCA TCTGCAA TTGCTGTCGCTCA TCCTGAA CTTTCTGGCGCTGGTGTTC T M	A	S	S M	S	S	L	L	S	S	N	F	S	P V	I	F	S	S	L A	N V	I	A	N	G A	S A	I	A	V	A	H	P	E	L	S	G A	G V

TTCGGTTTCGTCA TCTCTCAA CA GTGTTGTGTCTTCCGGTGCTTCA GCAA GTA GCTTTGCTTCTA TTGCAA CCCA GA TTGCTGA TGTA GCA GGTCA GGTGTCTTCA GTTCA CCCTGAA CTGTCAA GCCTTGAA GTCC S V	S	S	S	L	N	S V	V	S	S	G A	S A	S	S	F A	S I	A	T	Q I	A	D V	A	G	Q V	S	S V	H	P	E	L	S	S	L	E V

GCAA TTAA CTTCA TCTGTGA CGAA TGCGGTC------GGCCCCAA TGGA GTTGA TGCTAA TGCA CTTGCCCGTA GTCTTCAA TCTA GTTTCTCGAA TCTG---A GAA GTTCC---GGAA TGTCCTCTA GCGA TGCTA Q	L	T	S	S V	T	N A	V ------ G	P	N	G V	D A	N A	L A	R	S	L	Q	S	S	F	S	N	L --- R	S	S --- G M	S	S	S	D A

CCGCCTTTCTTCGTCTGTTGTAAA CGCA CTT------GGA CCTAA TGGCTTGGA CA TCAA TAA CTTTTCTGA TGGTCTGCGAA CTA CTCTTTCTCA GCTT---TCCA GTTCA ---GGCTTGTCTAAAAA GGAA GCGG R	L	S	S	S V	V	N A	L ------ G	P	N	G	L	D I	N	N	F	S	D	G	L R	T	T	L	S	Q	L --- S	S	S --- G	L	S	K	K	E A

TCGCCTCA CTTCGTCTGTTGTAAA TGCYCTC------GGA CCTAA TGGCTTGGA TGTCAA CAA CTTTTCTAA TGGCTTGCGW GCTA CTCTTTCA CA GCTG---TCTA GTTCA ---GGTTTGTCTAAAAA GGAA GCGG R	L	T	S	S V	V	N A	L ------ G	P	N	G	L	D V	N	N	F	S	N	G	L R	A	T	L	S	Q	L --- S	S	S --- G	L	S	K	K	E A

CGA TA TGGTGAA TGGTA TAA TGA GTGCTA TGCAA GGA TCTGGTTTTAA CTA C------CAAA TGTTTGGCAA TA TGCTA TCA CAA TA TTCTTCTGGTTCA GGGTCA TGCAA TCCAAA TAA TGTTAA T------GTTT D M	V	N	G I	M	S A	M	Q	G	S	G	F	N	Y ------ Q M	F	G	N M	L	S	Q	Y	S	S	G	S	G	S	C	N	P	N	N V	N ------ V

CGA TA TGGTGAA TGGTA TAA TGA GTGCTA TGCAA GGA TCTGGTTTTAA CTA C------CAAA TGTTTGGTAA TA TGCTA TCA CAA TA TTCGTCTGGTTCA GGAA CA TGCAA TCCAAA TAA TGTTAA T------GTTT D M	V	N	G I	M	S A	M	Q	G	S	G	F	N	Y ------ Q M	F	G	N M	L	S	Q	Y	S	S	G	S	G	T	C	N	P	N	N V	N ------ V

A TCTTTGGTGAA TGGA CTGA TGGGCTCTA TGCAA CCAA CCGGA TTTAA CTA T------CA GAA CTTCGGAAA TGTTCTGTCTCA GTA CGCCA CA GGCTCCGGAA CA TGCAA CA GCAA CGA TGTGAA T------CTGC S	L V	N	G	L M	G	S M	Q	P	T	G	F	N	Y ------ Q	N	F	G	N V	L	S	Q	Y A	T	G	S	G	T	C	N	S	N	D V	N ------ L

TA GTTTA GCTTCA TCA GTA GCTTCTGCTA TA GCCTCTGGT---CAA GCTCTAA GTGCGGA TA GTTTTGCAAAA TCGTTA TTGA TCCAA GCTTCCCAAA TTCAAA GTA GTGCA CCTA GTTTTAAA GCA GA TGA TGTA G S	L A	S	S V	A	S A	I	A	S	G --- Q A	L	S A	D	S	F A	K	S	L	L I	Q A	S	Q I	Q	S	S A	P	S	F	K A	D	D V

CA GTTTA GCTCAA TCTCTGGCGTCTGCA TTGTCGA CTTCTCGA GGTA CTTTGA GTTTA TCAA CCTTCTTGAA TCTCCTCTCTCCGA TTTCGTCA GAAA TT---CGA GCCAA TA CTTCTCTTGA TGGAA CGCA GGCGA S	L A	Q	S	L A	S A	L	S	T	S R	G	T	L	S	L	S	T	F	L	N	L	L	S	P I	S	S	E I --- R	A	N	T	S	L	D	G	T	Q A

TGA CA TTGCA CAAA GTTTA TCTTCAA CTCTA TCTTCA GGA TCGCAA ---TTA GCTCCA GA TAA TGTA CTTCCTGGTCTCA TTCAA CTGTCTTCA TCCA TTCAAA GCGGAAA TCCTGA TTTA GA CCCTGCTGGTGTTT D I	A	Q	S	L	S	S	T	L	S	S	G	S	Q --- L A	P	D	N V	L	P	G	L I	Q	L	S	S	S I	Q	S	G	N	P	D	L	D	P A	G V

TA GTTTA GCCAA CTCCTTTGCTTCTGCGA TTTCTTCA TCTGGA GGTTCCCTCA GTGTTCCAA CCTTCTTGAA TCTTCTTTCA TCCGTTGGGGCCCAA GTT---A GTA GTA GCA GTTCTTTGA GTTCCTTGGAA GTTA S	L A	N	S	F A	S A	I	S	S	S	G	G	S	L	S V	P	T	F	L	N	L	L	S	S V	G A	Q V --- S	S	S	S	S	L	S	S	L	E V

TA GTTTA GCCAA TTCCGTTGCTTCTGCTA TTTCTTCA TCTGGA GGCTCCCTCA GTGTTCCAA CCTTCTTGAA TTTTCTTTCA TCCGTTGGGGCTCAA GTT---A GCA GTA GTA GTTCTTTGAA TTCCTCCGAA GTTA S	L A	N	S V	A	S A	I	S	S	S	G	G	S	L	S V	P	T	F	L	N	F	L	S	S V	G A	Q V --- S	S	S	S	S	L	N	S	S	E V

Dsfibroin1a

Frame 1

DsMaSp2

Frame 1

NcMaSp1a

Frame 1

NcMaSp1b

Frame 1

NcMaSp2

Frame 1

NiMaSp2

Frame 1

LgMaSp1

Frame 1

LheMaSp1

Frame 1

LheMaSp2

Frame 1

EaMaSp

Frame 1

AtMaSp2

Frame 1

AapMaSp

Frame 1

UdMiSp

Frame 1

MgMiSp

Frame 1

LheMiSp

Frame 1

AvMiSp

Frame 1

Bcfibroin1

Frame 1

DcMaSp_like

Frame 1

DcMaSp

Frame 1

AargAcsp1Full

Frame 1

LheAcSp1

Frame 1

LgAcSp1

Frame 1

NiFlag

Frame 1

NcFlag

Frame 1

AvFlag

Frame 1

AapTuSp1

Frame 1

NcTuSp1

Frame 1

LheTuSp1

Frame 1

AbTuSp1

Frame 1

AaTuSp1

Frame 1

690	700	710	720	730	740	750	760	770	780	790	800	810	820

TTGTCA TA GCTCTCCTTGA GA TTGTTTCA GCTTTGGGTCA CCTA TTGAA TAA TTCGA TGA TT---AA TGTTAA TGCA GCAA GTGTTCCTGTTA GCCCTCCT------------CAA TGGCTA TA T F V	I	A	L	L	E I	V	S A	L	G	H	L	L	N	N	S M	I --- N V	N A	A	S V	P V	S	P	P ------------ Q W	L	Y

TTGTTCAA GTTTTGTTGGAA GTGA TTTCGGCTCTGGTGCA TA TTCTGGGTTCA GCA TCTGTTGGGCAA GTT------GGTTCTTCCCCA CAAAA TGCTCA GA TGGTTGCGGCTAA TGCTGTGGCAAA TGCA TTTTCT L V	Q V	L	L	E V	I	S A	L V	H I	L	G	S A	S V	G	Q V ------ G	S	S	P	Q	N A	Q M	V	A	A	N A	V	A	N A	F	S

TCGTCCAA GCTCTCCTTGA GGTTGTTTCTGCTCTCA TCCA TA TCTTA GGA TCTTCCA GCA TCGGCCAA GTTAA TTA TGGTTCA GCTGGTCAA GCCA CTCA GA TCGTTGGC---CAA TCAA TTTA TCAA GCTCTTGGT L V	Q A	L	L	E V	V	S A	L I	H I	L	G	S	S	S I	G	Q V	N	Y	G	S A	G	Q A	T	Q I	V	G --- Q	S I	Y	Q A	L	G

TCA TTCAA GCTCTTCTCGA GGTTGTTTCTGCTCTTA TCCA CA TCTTA GGTTCTTCCA GCA TCGGCCAA GTTAA CTA TGGTTCCGCTGGA CAA GCCA CTCA GA TCGTTGGT---CAA TCA GTTTA TCAA GCCCTA GGT L I	Q A	L	L	E V	V	S A	L I	H I	L	G	S	S	S I	G	Q V	N	Y	G	S A	G	Q A	T	Q I	V	G --- Q	S V	Y	Q A	L	G

TCA TTCAA GCTCTCTTGGAAA TCGTTTCTGCTTGTGTAA CCA TCCTTTCTTCA TCCA GCA TTGGTCAA GTTAA TTA TGGA GCGGCTTCTCA GTTCGCCCAA GTTGTCGGC---CAA TCTGTTTTGA GTGCA TTT L I	Q A	L	L	E I	V	S A	C V	T I	L	S	S	S	S I	G	Q V	N	Y	G A	A	S	Q	F A	Q V	V	G --- Q	S V	L	S A	F

TCA TTCAA GCTCTCTTGGAAA TCGTTTCGGCTTGTGTAA CCA TTCTTTCTTCA TCTA GCA TTGGTCAA GTTAA TTA TGGA GCGGCT??????????????????????????????????????????????????? L I	Q A	L	L	E I	V	S A	C V	T I	L	S	S	S	S I	G	Q V	N	Y	G A	A	?	?	?	?	?	?	?	?	?	?	?	?	?	?	?	?	?

TTGTA CAA GCTCTTCTTGAA CTA GTTA CTGCA TTA CTCA CTA TAA TTGGGTCCTCTAA TGTTAA TGA TA TAAA TTA TGGTTCGTCA GGTCA GTA TGCCCAAA TGGTTTCT---CA GTCA GTA CA GAA TGTGTTTGGA L V	Q A	L	L	E	L V	T A	L	L	T I	I	G	S	S	N V	N	D I	N	Y	G	S	S	G	Q	Y A	Q M	V	S --- Q	S V	Q	N V	F	G

TCGTTCAA GCTCTTCTTGAA CTTGTTA CTGCTTTGCTCA CCA TTA TTGGA TCA TCAAA TA TTGGCA GTGTTAA TTA TGA TTCTTCA GGCCAA TA TGCGCAA GTTGTTA CT---CAA TCTGTTCAAAA TGCA TTCGCT L V	Q A	L	L	E	L V	T A	L	L	T I	I	G	S	S	N I	G	S V	N	Y	D	S	S	G	Q	Y A	Q V	V	T --- Q	S V	Q	N A	F A

TTGTTCAA GCA CTTCTTGAAA TAA TTA CTGCA TTAA TTA GTA TA CTA GA TTCCTCTA GTGTTGGA CAA GTTAA TTA CGGTTCTTCA GGA CA GTA TGCA CAAA TTGTA GGG---CA GTCTA TGCAA CA GGCTA TGGGG L V	Q A	L	L	E I	I	T A	L I	S I	L	D	S	S	S V	G	Q V	N	Y	G	S	S	G	Q	Y A	Q I	V	G --- Q	S M	Q	Q A	M	G

TA GTGCAA GCTCTA CTCGAA GTCA TCA CTGCTCTTGTTCAAA TCGTTA GTTCTTCTA GTGTTGGA TA TA TTAA TCCA TCTGCTGTGAA CCAAA TTA CTAA TGTTGTTGCT---AA TGCCA TGGCTCAA GTAA TGGGC I	V	Q A	L	L	E V	I	T A	L V	Q I	V	S	S	S	S V	G	Y I	N	P	S A	V	N	Q I	T	N V	V	A --- N A	M	A	Q V	M	G

TCGTCCAA GCA TTGCTGGAAA TTGTA TCCGCCTTGGTA CA TA TCCTTGGA TCTTCTA GCA TTGGGCAAA TTAA TTA CGCCGCTTCTTCTCA GTA TGCGCAAA TGGTTGG???????????????????????????? L V	Q A	L	L	E I	V	S A	L V	H I	L	G	S	S	S I	G	Q I	N	Y A	A	S	S	Q	Y A	Q M	V	G	?	?	?	?	?	?	?	?	?

TA GTGCAA GTTCTCA TTGA GGTTGTTTCTGCTCTTGTGCA TA TTTTA GGA TCTGCCAA CA TTGGAAA CA TTAA TA TGAA TGCA GCA TCA TCTA CTGCTGCTGCTGTTGGT---CA GGCAA TA GTAAA TGGGTTGTA C L V	Q V	L I	E V	V	S A	L V	H I	L	G	S A	N I	G	N I	N M	N A	A	S	S	T A	A	A	V	G --- Q A	I	V	N	G	L	Y

CTGTGCAA GCTCTTCTTGAA GTAA TTTCGGTTCTTA CA CA CA TGCTCA GTTCGGCAAA CA TTGGA TA CGTCGA TTTCA GTCGTGTTGGA GA TTCCGCA TCTGCTGTCTCT---CAA TCAA TG------GCCTA TGCC T V	Q A	L	L	E V	I	S V	L	T	H M	L	S	S A	N I	G	Y V	D	F	S R	V	G	D	S A	S A	V	S --- Q	S M ------ A	Y A

TTGTTCA GGCCCTTTTGGAA GTCA TA TCTGCA CTTGTTCA TTTGTTGA GCAA TGCTTCTA TTGGTAA CGTTA GCTCA GCTGGA TTA GGTAA TA CAA TGA GTCTTGTT------CA GA GTA CGGTA GGTGCGTA CGCC L V	Q A	L	L	E V	I	S A	L V	H	L	L	S	N A	S I	G	N V	S	S A	G	L	G	N	T M	S	L V ------ Q	S	T V	G A	Y A

TTA TCCAA GCCCTA CTTGAA TTGGTGTCTTCTCTTA TA CA TA TTCTCA GCTCTGCTAA CA TCGGTCAA GTCGA CTTCAA CTCTGTTGGAAA TA CA GCTGCTGTA GTTGGT---CAA TCA CTT---GGGGCA GCTTTA V	I	Q A	L	L	E	L V	S	S	L I	H I	L	S	S A	N I	G	Q V	D	F	N	S V	G	N	T A	A	V	V	G --- Q	S	L --- G A	A	L

TGA TTCAA GCTCTGTTGGAA CTCCTTTCCGCA CTTGTTCA TGTTTTAA GCA GTGCCTCTA TCGGTAA TGTTA GCTCA GTA GGA GTA GA TA GTA CA TTGAA TGTTGTTCA G---GA TTCA GTA ---GGCCAA TA TGTA L I	Q A	L	L	E	L	L	S A	L V	H V	L	S	S A	S I	G	N V	S	S V	G V	D	S	T	L	N V	V	Q --- D	S V --- G	Q	Y V

TAA CGGAA GCTCTCCTA GAA GTA TTGGCTGGTTGTA TGGAAA TCCTGTCTTCA TTCAA TGTTGGA GCA CA GA GTA TTTCTTCA TCTA GAA CA TCA TCTAA TGCTCTGGTT---CA GTCAA TA TCTAA CCAA TTTTCA V	T	E A	L	L	E V	L A	G	C M	E I	L	S	S	F	N V	G A	Q	S I	S	S	S R	T	S	S	N A	L V --- Q	S I	S	N	Q	F	S

TTA CGGAA GCCTTGTTGGA GGCCCTGGTA GCA TTTTGCTA CGCTCA TGCTGGA TCT---A TTGCTTCTGCGA GCGA CGTTAA TCTA GCTGTAA CCA CTTTGGCA TCCA CG---A TGCA GGGTTCTTTGCCT L	T	E A	L	L	E A	L V	A	F	C	Y A	H A	G	S --- I	A	S A	S	D V	N	L A	V	T	T	L A	S	T --- M	Q	G	S	L	P

TTGTTGAA GCCTTGA TTGA GA TTA TTGTGGCGCTTGTA CA TGCCCA GTCTGGTGGT---GCTTCCTCA GCAA GTGCCA GTGCA TCCGCCA GTGCG---CTGGCCTCA ------CA GTTA GGGCA GCTCTTTGGC L V	E A	L I	E I	I	V	A	L V	H A	Q	S	G	G --- A	S	S A	S A	S A	S A	S A --- L A	S ------ Q	L	G	Q	L	F	G

AAA TTGAA GTTTTGCTA GAAA CCA TA GTCA GTCTGCTGCAA CTTTTGA GCAA CA CA CA GA TTCGA GGA GTGAA TCCGGCAA CGGCA TCTTCCGTGGCCAA TTCTGCTGCC---A GA TCCTTTGAA TTA GTTTTA GCT K I	E V	L	L	E	T I	V	S	L	L	Q	L	L	S	N	T	Q I	R	G V	N	P A	T A	S	S V	A	N	S A	A --- R	S	F	E	L V	L A

CTA TCGAAA CCCTCA TGGAA GCAA TGGTTGCTCTGTTA CAA GTTCTAAA TTCA GCA CAA GTAAA TCAA GTTGA TA CA TCTTCTA CTGTTGTAA CAA GTA GTTCA CTTGCC---AAA GCTTTA TCTTCTTTGTTT A	I	E	T	L M	E A	M	V	A	L	L	Q V	L	N	S A	Q V	N	Q V	D	T	S	S	T V	V	T	S	S	S	L A --- K A	L	S	S	L	F

CTA ?????????????????????????????????????????????????????????????????????????????????????????????????????????????????????????????????????? A	?	?	?	?	?	?	?	?	?	?	?	?	?	?	?	?	?	?	?	?	?	?	?	?	?	?	?	?	?	?	?	?	?	?	?	?	?	?	?	?	?	?	?	?	?

TGA TGGA TGCTTTGTTA ---------GCTGCTTTG---CA CTGTCTAA GTAA CCA CGGA TCA TCA TCTTTTGCA CCTTCTCCAA CTCCGGCTGCTA TGA GTGCTTA TTCT---AA TTCTGTA GGAA GAA TGTTCGCT L M	D A	L	L --------- A	A	L --- H	C	L	S	N	H	G	S	S	S	F A	P	S	P	T	P A	A	M	S A	Y	S --- N	S V	G R	M	F A

TGA TGGA TGCTTTGTTA ---------GCTGCTTTG---CA CTGTCTAA GTAA CCA CGGA TCA TCA TCTTTTGCA CCTTCTCCAA CTCCGGCTGCTA TGA GTGCGTA TTCT---AA TTCTGTA GGAA GAA TGTTCGCT L M	D A	L	L --------- A	A	L --- H	C	L	S	N	H	G	S	S	S	F A	P	S	P	T	P A	A	M	S A	Y	S --- N	S V	G R	M	F A

TGA TGGA CGCGCTTA TG---------GCGGCTTTG---CA TTGCCTGTCTTA C---GGA TCCGGTTCTGTTCCGTCTA CTCCGA CTTA TTCTGCGA TGTCTGCCTA CAA C---CA GTCTA TCCGTA GAA TGTTTA CA L M	D A	L M --------- A	A	L --- H	C	L	S	Y --- G	S	G	S V	P	S	T	P	T	Y	S A	M	S A	Y	N --- Q	S I	R	R	M	F	T

TCCA CGAA TCTCTCCTTGAA GGAA TTTCA GCTCTTA TCCAA GTTA TCAA CA GCA GTTA TGGTTCA CCTTTGA GTTTGTCCAA TGCCCA GA CTGTTAA CGCTGGA CTTGTA ---AA CTA TTTCCTTGTT V	H	E	S	L	L	E	G I	S A	L I	Q V	I	N	S	S	Y	G	S	P	L	S	L	S	N A	Q	T V	N A	G	L V --- N	Y	F	L V

CTGTTGAA GCTTTA CTGGAA GCTTTA GCTGCTCTCCTGCAA GTTA TCAA TGGA GCA CA GA TAA CCGA TGTCAA TGTTTCTA GCGTCCCCA GCGTGAA TGCA GCCCTGGCT---TCTGCTCTTGTTGCT T V	E A	L	L	E A	L A	A	L	L	Q V	I	N	G A	Q I	T	D V	N V	S	S V	P	S V	N A	A	L A --- S A	L V	A

TGA TCGA GTCA TTA TTA GAA TA CA CTTCCGCA CTTTTA GCTCTTCTTCAAAA CGCTCAAA TTA CAA CTTA TGA TGCTGCGA CTTTA CCTGCA TTCAA TA CA GCTCTTGTA ---AA TTA CCTTGTTCCCCTTGTT L I	E	S	L	L	E	Y	T	S A	L	L A	L	L	Q	N A	Q I	T	T	Y	D A	A	T	L	P A	F	N	T A	L V --- N	Y	L V	P	L V

CAAA CGAA GTTTTA CTTGAA GCTA TTGCGGCTCTCTTGCAA GTTA TCAA CGGA GGTTCAA TAA CA TCA GTTGA TCTTA GA TA CGTTCCGAA TGCTCA GCA GGA TTTGGTG---AA CGCTTTA TCTGGT T	N	E V	L	L	E A	I	A	A	L	L	Q V	I	N	G	G	S I	T	S V	D	L R	Y V	P	N A	Q	Q	D	L V --- N A	L	S	G

CAAA CGAA GTGTTA CTTGAA GCTA TCGCGGCA CTCTTGCAA GTCCTCAA CGGA GCTCAAA TAA CA TCA GTTAA TCTGA GAAA CGTTCCGAA TGCTCAA CA GGCTTTGGTG---CAA GCA TTGTCTGGT T	N	E V	L	L	E A	I	A	A	L	L	Q V	L	N	G A	Q I	T	S V	N	L R	N V	P	N A	Q	Q A	L V --- Q A	L	S	G

Dsfibroin1a

Frame 1

DsMaSpZ Frame 1

NcMaSp1a

Frame 1

NcMaSp1b

Frame 1

NcMaSpZ Frame 1

NiMaSpZ Frame 1

LgMaSp1

Frame 1

LheMaSp1

Frame 1

LheMaSpZ Frame 1

EaMaSp

Frame 1

AtMaSpZ Frame 1

AapMaSp

Frame 1

UdMiSp

Frame 1

MgMiSp

Frame 1

LheMiSp

Frame 1

AvMiSp

Frame 1

Bcfibroin1

Frame 1

DcMaSp_like

Frame 1

DcMaSp

Frame 1

AargAcsp1Full

Frame 1

LheAcSp1

Frame 1

LgAcSp1

Frame 1

NiFiag

Frame 1

NcFiag

Frame 1

AvFiag

Frame 1

AapTuSp1

Frame 1

NcTuSp1

Frame 1

LheTuSp1

Frame 1

AbTuSp1

Frame 1

AaTuSp1

Frame 1

830	840

??????????????????

??????

-

GGT

G

GGT

G

GGA

G

```
GGT

GGATTAAATGCAGCTGCT G 	N A  A  A
```

??????????????????

TAT

y

TAT

y

TAT

y

**Supplementary Figure S2. Nucleotide alignment of 30 concatenated AcSp1 N and C terminal coding regions.** Alignment position numbers shown in increments of ten. Frame 1 translation is shown under nucleotide sequences. Alignment positions 1–510 encompass the N-terminal coding region, 511–840 the C-terminal coding region.

Alignment prepared with Geneious v6.1.6 (Biomatters Ltd., Auckland, NZ) and available on TreeBASE ([http://purl.org/phylo/treebase/phylows/study/TB2:](http://purl.org/phylo/treebase/phylows/study/TB2%3AS15355))S15355). Abbreviatons and accession numbers in Table S2.

Aar_R_1

Frame 1

Aar_R_2

Frame 1

Aar_R_3

Frame 1

Aar_R_4

Frame 1

Aar_R_5

Frame 1

Aar_R_6

Frame 1

Aar_R_7

Frame 1

Aar_R_8

Frame 1

Aar_R_9

Frame 1

1	10	20	30	40	50	60	70	80	90	100	110

C T TA T C T C CA G A G TA G CA A A CG CA C T T T C CA A TA CG T C CA CA T TG A G G A CA G T T C T C CG CA G A G G TG TG T C C CA A CA G A T TG C C T CA A G TA TA T TG CG A A G A G C CG C T CA G A C T T L I	S	R	V	A	N	A	L S	N	T S	T	L R	T V	L R	R	G	V	S	Q	Q I	A	S	S	I	L R	R	A	A	Q	T

C T CA T C T C CA G A G TA G CA A A CG CA C T TG C CA A TA CA T C CA CA T TG A G A G C TG T C C T C CG A A G A G G TG TA T C C CA A CA G A C TG C C T C CA G CG TG G TA CA G A G A G C CG C T CA G T CG T L I	S	R	V	A	N	A	L A	N	T S	T	L R	A	V	L R	R	G	V	S	Q	Q	T A	S	S	V	V	Q R	A	A	Q S

C T CA T C T C CA G A G TA G CA A A CG CA C T TG C CA A TA CA T C CA CA T TG A G A G C TG T C C T C CG A A G A G G TG TA T C C CA A CA G A C TG C C T C CA G CG TG G TA CA G A G A G C CG C T CA G T CG T L I	S	R	V	A	N	A	L A	N	T S	T	L R	A	V	L R	R	G	V	S	Q	Q	T A	S	S	V	V	Q R	A	A	Q S

C T CA T C T C CA G A G TA G CA A A CG CA C T TG C CA A TA CA T C CA CA T TG A G A G C TG T C C T C CG A A G A G G TG TA T C C CA A CA G A C TG C C T C CA G CG TG G TA CA G A G A G C CG C T CA G T CG T L I	S	R	V	A	N	A	L A	N	T S	T	L R	A	V	L R	R	G	V	S	Q	Q	T A	S	S	V	V	Q R	A	A	Q S

C T CA T C T C CA G A G TA G CA A A CG CA C T TG C CA A TA CA T C CA CA T TG A A A G C TG T T C T C CG A A G A G G TG TA T C C CA A CA G A C TG C C T C CA G CG TG G TA CA G A G A G C CG C T CA G T CG T L I	S	R	V	A	N	A	L A	N	T S	T	L K	A	V	L R	R	G	V	S	Q	Q	T A	S	S	V	V	Q R	A	A	Q S

C T CA T C T C CA G A G TA G CA A A CG CA C T TG C CA A TA CA T C CA CA T TG A G A G C TG T C C T C CG A A G A G G TG TA T C C CA A CA G A C TG C C T C CA G CG TG G TA CA G A G A G C CG C T CA G T CG T L I	S	R	V	A	N	A	L A	N	T S	T	L R	A	V	L R	R	G	V	S	Q	Q	T A	S	S	V	V	Q R	A	A	Q S

C T CA T C T C CA G A G TA G CA A A CG CA C T TG C CA A TA CA T C CA CA T TG A A A G C TG T C C T C CG A A G A G G TG TA T C C CA A CA G A C TG C C T C CA G CG TG G TA CA G A G A G C CG C T CA G T CG T L I	S	R	V	A	N	A	L A	N	T S	T	L K	A	V	L R	R	G	V	S	Q	Q	T A	S	S	V	V	Q R	A	A	Q S

C T CA T C T C CA G A G TA G CA A A CG CA C T TG C CA A TA CA T C CA CA T TG A G A G C TG T C T T C CG A A G A G G TG TA T C C CA A CA G A C TG C C T C CA G CG TG G TA CA G A G A G C CG C T CA G T CG T L I	S	R	V	A	N	A	L A	N	T S	T	L R	A	V	F R	R	G	V	S	Q	Q	T A	S	S	V	V	Q R	A	A	Q S

C T CA T C T C CA G A G TA G CA A A CG CA C T TG C CA A TA CA T C CA CA T TG A G A G C TG T C C T C CG A A G A G G TG TA T C C CA A CA G A C TG C T T C CA G CG TG G TA CA G A G A G C CG C T CA G T CG T L I	S	R	V	A	N	A	L A	N	T S	T	L R	A	V	L R	R	G	V	S	Q	Q	T A	S	S	V	V	Q R	A	A	Q S

Aar_R_10	C T CA T C T C CA G A G TA G CA A A CG CA C T TG C CA A TA CA T C CA CA T TG A G A G C TG T C C T C CG A A G A G G TG TA T C C CA A CA G A C TG C C T C CA G CG TG G TA CA G A G A G C CG C T CA G T CG T

Frame 1

L I	S	R	V	A	N	A	L A	N	T S	T	L R	A	V	L R	R	G	V	S	Q	Q	T A	S	S	V	V	Q R	A	A	Q S

Aar_R_11	C T CA T C T C CA G A G TA G CA A A CG CA C T TG C CA A TA CA T C CA CA T TG A G A G C TG T C C T C CG A A G A G G TG TA T C C CA A CA G A C TG C C T C CA G CG TG G TA CA G A G A G C CG C T CA G T CG T

Frame 1

L I	S	R	V	A	N	A	L A	N	T S	T	L R	A	V	L R	R	G	V	S	Q	Q	T A	S	S	V	V	Q R	A	A	Q S

Aar_R_12	C T CA T C T C CG G A G TA G CA A A CG CA C T TG C CA A TA CA T C CA CA T TG A G A G C TG T C T T C CG A A G A G G TG TA T C C CA A CA G A C TG C C T C CA G CG TG G TA CA G A G A G C CG C T CA G T CG T

Frame 1

L I	S	G	V	A	N	A	L A	N	T S	T	L R	A	V	F R	R	G	V	S	Q	Q	T A	S	S	V	V	Q R	A	A	Q S

Aar_R_13	C T CA T C T C CA G A G TA G CA A A CG CA C T TG C CA A TA CA T C CA CA T TG A G A G C TG T C C T C CG A A G A G G TG TA T C C CA A CA G A C TG C T T C CA G CG TG G TA CA G A G A G C CG C T CA G T CG T

Frame 1

L I	S	R	V	A	N	A	L A	N	T S	T	L R	A	V	L R	R	G	V	S	Q	Q	T A	S	S	V	V	Q R	A	A	Q S

Aar_R_14	C T CA T C T C CA G A G TA G CA A A CG CA C T TG C CA A TA CA T C CA CA T TG A G A G C TG T C C T C CG A A G A G G TG TA T C C CA A CA G A C TG C C T C CA G CG TG G TA CA G A G A G C CG C T CA G T CG T

Frame 1

L I	S	R	V	A	N	A	L A	N	T S	T	L R	A	V	L R	R	G	V	S	Q	Q	T A	S	S	V	V	Q R	A	A	Q S

Aar_R_15	C T CA T C T C CA G A G TA G CA A A CG CA C T TG C CA A TA CA T C CA CA T TG A G A G C TG T C C T C CG A A G A G G TG TA T C C CA A CA G A C TG C C T C CA G CG TG G TA CA G A G A G C CG C T CA G T CG T

Frame 1

L I	S	R	V	A	N	A	L A	N	T S	T	L R	A	V	L R	R	G	V	S	Q	Q	T A	S	S	V	V	Q R	A	A	Q S

Aar_R_16	C T CA T C T C CA G A G TA G CA A A CG CA C T TG C CA A TA CA T C CA CA T TG A G A G C TG T C C T C CG A A G A G G TG TA T C C CA A CA G A C TG C C T C CA G CG TG G TA CA G A G A G C CG C T CA G T CG T

Frame 1

L I	S	R	V	A	N	A	L A	N	T S	T	L R	A	V	L R	R	G	V	S	Q	Q	T A	S	S	V	V	Q R	A	A	Q S

Aar_R_17	C T CA T C T C CA G A G TA G CA A A CG CA C T TG C CA A TA CA T C CA CA T TG A G A G C TG T C C T C CG A A G A G G TG TA T C C CA A CA G A C TG C C T C CA G CG TG G TA CA G A G A G C CG C T CA G T CG T

Frame 1

L I	S	R	V	A	N	A	L A	N	T S	T	L R	A	V	L R	R	G	V	S	Q	Q	T A	S	S	V	V	Q R	A	A	Q S

Aar_R_18	C T CA T C T C CA G A G TA G CA A A CG CA C T TG C CA A TA CA T C CA CA T TG A G A G C TG T C C T C CG A A G A G G TG TA T C C CA A CA G A C TG C C T C CA G CG TG G TA CA G A G A G C CG C T CA G T CG T

Frame 1

L I	S	R	V	A	N	A	L A	N	T S	T	L R	A	V	L R	R	G	V	S	Q	Q	T A	S	S	V	V	Q R	A	A	Q S

Aar_R_19	C T CA T C T C CA G A G TA G CA A A CG CA C T TG C CA A TA CA T C CA CA T TG A G A G C TG T C C T C CG A A G A G G TG TA T C C CA A CA G A C TG C C T C CA G CG TG G TA CA G A G A G C CG C T CA G T CG T

Frame 1

L I	S	R	V	A	N	A	L A	N	T S	T	L R	A	V	L R	R	G	V	S	Q	Q	T A	S	S	V	V	Q R	A	A	Q S

Aar_R_20	C T CA T C T C CA G A G TA G CA A A CG CA C T TG C CA A TA CA T C CA CA T TG A G A G C TG T C C T C CG A A G A G G TG TA T C C CA A CA G A C TG C C T C CA G CG TG G TA CA G A G A G C CG C T CA G T CG T

Frame 1

At_R_1

Frame 1

At_R_2

Frame 1

At_R_3

Frame 1

At_R_4

Frame 1

At_R_5

Frame 1

At_R_6

Frame 1

At_R_7

Frame 1

At_R_8

Frame 1

At_R_9

Frame 1

At_R_10

Frame 1

At_R_11

Frame 1

At_R_12

Frame 1

At_R_13

Frame 1

At_R_14

Frame 1

Av_R_1

Frame 1

Av_R_2

Frame 1

Av_R_3

Frame 1

Av_R_4

Frame 1

Av_R_5

Frame 1

Av_R_6

Frame 1

Av_R_7

Frame 1

Av_R_8

Frame 1

Av_R_9

Frame 1

Av_R_10

Frame 1

L I	S	R	V	A	N	A	L A	N	T S	T	L R	A	V	L R	R	G	V	S	Q	Q	T A	S	S	V	V	Q R	A	A	Q S

N N N N N N N N N N N N N N N N N N N N N N N N N N N N N N N N N N N N N N N N N N N N N N N N N N N N N N N N N N N N N N N N N N N N N N N N N N N N N N N N N N N N N N N N N N N N N N N N N N N N N N N N N N N N N N N N N N N

? ? ? ? ? ? ? ? ? ? ? ? ? ? ? ? ? ? ? ? ? ? ? ? ? ? ? ? ? ? ? ? ? ? ? ? ? ?

C T TA T C T C CA G A G TA G CA A A CG CA C T TG C CA A TA CA T CA A CA T TG A G A A C TG T C C T CA G A A C TG G TG TA T C C CA A CA G A T TG C C T C CA G CG TG G TA CA G A G A G C CG C T CA G T CG T L I	S	R	V	A	N	A	L A	N	T S	T	L R	T V	L R	T G	V	S	Q	Q I	A	S	S	V	V	Q R	A	A	Q S

C T TA T C T C CA G A G TA G CA A A CG CA C T TG C CA A TA CA T CA A CA T TG A G A A C TG T C C T CA G A A C TG G TG TA T C C CA A CA G A T TG C C T C CA G CG TG G TA CA G A G A G C CG C T CA G T CG T L I	S	R	V	A	N	A	L A	N	T S	T	L R	T V	L R	T G	V	S	Q	Q I	A	S	S	V	V	Q R	A	A	Q S

C T TA T C T C CA G A G TA G CA A A CG CA C T TG C CA A TA CA T CA A CA T TG A G A A C TG T C C T CA G A A C TG G TG TA T C C CA A CA G A T TG C C T C CA G CG TG G TA CA G A G A G C CG C T CA G T CG T L I	S	R	V	A	N	A	L A	N	T S	T	L R	T V	L R	T G	V	S	Q	Q I	A	S	S	V	V	Q R	A	A	Q S

C T TA T C T C CA G A G TA G CA A A CG CA C T TG C CA A TA CA T CA A CA T TG A G A A C TG T C C T CA G A A C TG G TG TA T C C CA A CA G A T TG C C T C CA G CG TG G TA CA G A G A G C CG C T CA G T CG T L I	S	R	V	A	N	A	L A	N	T S	T	L R	T V	L R	T G	V	S	Q	Q I	A	S	S	V	V	Q R	A	A	Q S

C T TA T C T C CA G A G TA G CA A A CG CA C T TG C CA A TA CA T CA A CA T TG A G A A C TG T C C T CA G A A C TG G TG TA T C C CA A CA G A T TG C C T C CA G CG TG G TA CA G A G A G C CG C T CA G T CG T L I	S	R	V	A	N	A	L A	N	T S	T	L R	T V	L R	T G	V	S	Q	Q I	A	S	S	V	V	Q R	A	A	Q S

C T TA T C T C CA G A G TA G CA A A CG CA C T TG C CA A TA CA T CA A CA T TG A G A A C TG T C C T CA G A A C TG G TG TA T C C CA A CA G A T TG C C T C CA G CG TG G TA CA G A G A G C CG C T CA G T CG T L I	S	R	V	A	N	A	L A	N	T S	T	L R	T V	L R	T G	V	S	Q	Q I	A	S	S	V	V	Q R	A	A	Q S

C T TA T C T C CA G A G TA G CA A A CG CA C T TG C CA A TA CA T CA A CA T TG A G A A C TG T C C T CA G A A C TG G TG TA T C C CA A CA G A T TG C C T C CA G CG TG G TA CA G A G A G C CG C T CA G T CG T L I	S	R	V	A	N	A	L A	N	T S	T	L R	T V	L R	T G	V	S	Q	Q I	A	S	S	V	V	Q R	A	A	Q S

C T TA T C T C CA G A G TA G CA A A CG CA C T TG C CA A TA CA T CA A CA T TG A G A A C TG T C C T CA G A A C TG G TG TA T C C CA A CA G A T TG C C T C CA G CG TG G TA CA G A G A G C CG C T CA G T CG T L I	S	R	V	A	N	A	L A	N	T S	T	L R	T V	L R	T G	V	S	Q	Q I	A	S	S	V	V	Q R	A	A	Q S

C T TA T C T C CA G A G TA G CA A A CG CA C T TG C CA A TA CA T CA A CA T TG A G A A C TG T C C T CA G A A C TG G TG TA T C C CA A CA G A T TG C C T C CA G CG TG G TA CA G A G A G C CG C T CA G T CG T L I	S	R	V	A	N	A	L A	N	T S	T	L R	T V	L R	T G	V	S	Q	Q I	A	S	S	V	V	Q R	A	A	Q S

C T TA T C T C CA G A G TA G CA A A CG CA C T TG C CA A TA CA T CA A CA T TG A G A A C TG T C C T CA G A A C TG G TG TA T C C CA A CA G A T TG C C T C CA G CG TG G TA CA G A G A G C CG C T CA G T CG T L I	S	R	V	A	N	A	L A	N	T S	T	L R	T V	L R	T G	V	S	Q	Q I	A	S	S	V	V	Q R	A	A	Q S

C T TA T C T C CA G A G TA G CA A A CG CA C T TG C CA A TA CA T CA A CA T TG A G A A C TG T C C T CA G A A C TG G TG TA T C C CA A CA G A T TG C C T C CA G CG TG G TA CA G A G A G C CG C T CA G T CG T L I	S	R	V	A	N	A	L A	N	T S	T	L R	T V	L R	T G	V	S	Q	Q I	A	S	S	V	V	Q R	A	A	Q S

C T TA T C T C CA G A G TA G CA A A CG CA C T TG C CA A TA CA T CA A CA T TG A G A A C TG T C C T CA G A A C TG G TG TA T C C CA A CA G A T TG C C T C CA G CG TG G TA CA G A G A G C CG C T CA G T CG T L I	S	R	V	A	N	A	L A	N	T S	T	L R	T V	L R	T G	V	S	Q	Q I	A	S	S	V	V	Q R	A	A	Q S

C T TA T C T C CA G A G TA G CA A A CG CA C T TG C CA A TA CA T CA A CA T TG A G A A C TG T C C T CA G A A C TG G TG TA T C C CA A CA G A T TG C C T C CA G CG TG G TA CA G A G A G C CG C T CA G T CG T L I	S	R	V	A	N	A	L A	N	T S	T	L R	T V	L R	T G	V	S	Q	Q I	A	S	S	V	V	Q R	A	A	Q S

N N N N N N N N N N N N N N N N N N N N N N N N N N N N N N N N N N N N N N N N N N N N N N N N N N N N N N N N N N N N N N N N N N N N N N N N N N N N N N N N N N N N N N N N N N N N N N N N N N N N N N N N N N N N N N N N N N N

? ? ? ? ? ? ? ? ? ? ? ? ? ? ? ? ? ? ? ? ? ? ? ? ? ? ? ? ? ? ? ? ? ? ? ? ? ?

C T TA T C T C CA G A A TA G C TA A CG CA C TG G CG A A TA CA T CA A CA C TG A G A T CG G T C C T CA G A A C CG G TG TA T C C CA A CA G A T TG C C T C TA G CG TG G TA CA G A G A A C CA T C CA G A C C T L I	S	R	I	A	N	A	L A	N	T S	T	L R	S	V	L R	T G	V	S	Q	Q I	A	S	S	V	V	Q R	T I	Q	T

C T TA T C T C CA G A A TA G C TA A CG CA C TG G CG A A TA CA T CA A CA C TG A G A T CG G T C C T CA G A A C CG G TG TA T C C CA A CA G A C TG C C T C TA G CG TG G TA CA G A G A A C CA T C CA G A C C T L I	S	R	I	A	N	A	L A	N	T S	T	L R	S	V	L R	T G	V	S	Q	Q	T A	S	S	V	V	Q R	T I	Q	T

C T TA T C T C CA G A A TA G C TA A CG CA C TG G CG A A TA CA T CA A CA C TG A G A T CG G T C C T CA G A A C CG G TG TA T C C CA A CA G A C TG C C T C TA G CG TG G TA CA G A G A G C CA T C CA G A C C T L I	S	R	I	A	N	A	L A	N	T S	T	L R	S	V	L R	T G	V	S	Q	Q	T A	S	S	V	V	Q R	A	I	Q	T

C T TA T C T C CA G A A TA G C TA A CG CA C TG G CG A A TA CA T CA A CA C TG A G A T CG G T C C T CA G A A C CG G TG TA T C C CA A CA G A C TG C C T C TA G CG TG G TA CA G A G A A C CA T C CA G A C C T L I	S	R	I	A	N	A	L A	N	T S	T	L R	S	V	L R	T G	V	S	Q	Q	T A	S	S	V	V	Q R	T I	Q	T

C T TA T C T C CA G A A TA G C TA A CG CA C TG G CG A A TA CA T CA A CA C TG A G A T CG G T C C T CA G A A C CG G TG TA T C C CA A CA G A C TG C C T C TA G CG TG G TA CA G A G A G C CA T C CA G A C C T L I	S	R	I	A	N	A	L A	N	T S	T	L R	S	V	L R	T G	V	S	Q	Q	T A	S	S	V	V	Q R	A	I	Q	T

C T TA T C T C CA G A A TA G C TA A CG CA C TG G CG A A TA CA T CA A CA C TG A G A T CG G T C C T CA G A A C CG G TG TA T C C CA A CA G A C TG C C T C TA G CG TG G TA CA G A G A A C CA T C CA G A C C T L I	S	R	I	A	N	A	L A	N	T S	T	L R	S	V	L R	T G	V	S	Q	Q	T A	S	S	V	V	Q R	T I	Q	T

C T TA T C T C CA G A A TA G C TA A CG CA C TG G CG A A TA CA T CA A CA C TG A G A T CG G T C C T CA G A A C CG G TG TA T C C CA A CA G A T TG C C T C TA G CG TG G TA CA G A G A A C CA T C CA G A C C T L I	S	R	I	A	N	A	L A	N	T S	T	L R	S	V	L R	T G	V	S	Q	Q I	A	S	S	V	V	Q R	T I	Q	T

C T TA T C T C CA G A A TA G C TA A CG CA C TG G CG A A TA CA T CA A CA C TG A G A T CG G T C C T CA G A A C CG G TG TA T C C CA A CA G A C TG C C T CA A G CG TG G TA CA G A G A A C CA T C CA G A C C T L I	S	R	I	A	N	A	L A	N	T S	T	L R	S	V	L R	T G	V	S	Q	Q	T A	S	S	V	V	Q R	T I	Q	T

C T TA T C T C CA G A A TA G C TA A CG CA C TG G CG A A TA CA T CA A CA C TG A G A T CG G T C C T CA G A A C CG G TG TA T C C CA A CA G A C TG C C T C TA G CG TG G TA CA G A G A A C CA T C CA G A C C T L I	S	R	I	A	N	A	L A	N	T S	T	L R	S	V	L R	T G	V	S	Q	Q	T A	S	S	V	V	Q R	T I	Q	T

Aam_R_1	N N N N N N N N N N N N N N N N N N N N N N N N N N N N N N N N N N N N N N N N N N N N N N N N N N N N N N N N N N N N N N N N N N N N N N N N N N N N N N N N N N N N N N N N N N N N N N N N N N N N N N N N N N N N N N N N N N N

Frame 1

? ? ? ? ? ? ? ? ? ? ? ? ? ? ? ? ? ? ? ? ? ? ? ? ? ? ? ? ? ? ? ? ? ? ? ? ? ?

Aam_R_2	C T TG TG T C CA G A G TA G CA A A CG CA C T TG C CA A TA CA T CA A CA T TG A G A G C TG T C C T CA G A A G A G G TG TA T C C CA A CA G A CG G C C T C CA G CG TG G TA CA G A G A G C TG T T CA G T C T T

Frame 1

L V	S	R	V	A	N	A	L A	N	T S	T	L R	A	V	L R	R	G	V	S	Q	Q	T A	S	S	V	V	Q R	A	V	Q S

Aam_R_3	C T TG TG T C CA G A G TA G CA A A CG CA C T TG C CA A TA CA T CA A CA T TG A G A G C TG T C C T CA G A A G A G G TG TA T C C CA A CA G A CG G C C T C CA G CG TG G TA CA G A G A G C TG T T CA G T C T T

Frame 1

L V	S	R	V	A	N	A	L A	N	T S	T	L R	A	V	L R	R	G	V	S	Q	Q	T A	S	S	V	V	Q R	A	V	Q S

Aam_R_4	C T TG TG T C CA G A G TA G CA A A CG CA C T TG C CA A TA CA T CA A CA T TG A G A G C TG T C C T CA G A A G A G G TG TA T C C C CA CA G A CG G C C T C CA G CG TG G TA CA G A G A G C CG T T CA G T C T T

Frame 1

L V	S	R	V	A	N	A	L A	N	T S	T	L R	A	V	L R	R	G	V	S	P	Q	T A	S	S	V	V	Q R	A	V	Q S

Aam_R_5	C T TG TG T C CA G A G TA G CA A A CG CA C T TG C CA A TA CA T CA A CA T TG A G A G C TG T C C T CA G A A G A G G TG TA T C C CA A CA G A CG G C C T C CA G CG TG G TA CA G A G A G C CG T T CA G T C T T

Frame 1

L V	S	R	V	A	N	A	L A	N	T S	T	L R	A	V	L R	R	G	V	S	Q	Q	T A	S	S	V	V	Q R	A	V	Q S

Aam_R_6	C TG G TG T C CA G A G TA G CA A A CG CA C T TG C CA A TA CA T CA A CA T TG A G A G C TG T C C T CA G A A G A G G TG TA T C C CA A CA G A CG G C C T C CA G CG TG G T T CA G A G A G C CG T T CA G T C T T

Frame 1

L/ M V	S	R	V	A	N	A	L A	N	T S	T	L R	A	V	L R	R	G	V	S	Q	Q	T A	S	S	V	V	Q R	A	V	Q S

Aar_R_1

Frame 1

Aar_R_2

Frame 1

Aar_R_3

Frame 1

Aar_R_4

Frame 1

Aar_R_5

Frame 1

Aar_R_6

Frame 1

Aar_R_7

Frame 1

Aar_R_8

Frame 1

Aar_R_9

Frame 1

120	130	140	150	160	170	180	190	200	210	220	230

TG G C CA G TA C T C T CG G A G T CG A CG G A A A CA A C T TG T C CA G A G TA G CA T TA CA A G C CA T C T C T CA A G TG C C CA CA G G G T C TG A CA C T T CA G C T TA CG C T CA A G C C T T C T C TA G TG C L A	S	T	L G	V	D G	N	N	L S	R	V	A	L	Q A	I	S	Q V	P	T G	S	D	T S	A	Y A	Q A	F S	S	A

TG G C CA G TA C T C T CG G A A T CG A CG G A A A CA A C T TG T C CA G A G TA G CA T TA CA A G C CA C C T C T CA A G TG C C CA CA G G G T C TG A CA C T T CA G C T TA CG C T CA A G C C T T C T C TA G TG C L A	S	T	L G	I	D G	N	N	L S	R	V	A	L	Q A	T S	Q V	P	T G	S	D	T S	A	Y A	Q A	F S	S	A

TG G C CA G TA C T C T CG G A G T CG A CG G A A A CA A C T TG T C CA G A G TA G CA T TA CA A G C CA T C T C T CA A G TG C C CA CA G G G T C TG A CA C T T CA G C T TA CG C T CA A G C C T T C T C TA G TG C L A	S	T	L G	V	D G	N	N	L S	R	V	A	L	Q A	I	S	Q V	P	T G	S	D	T S	A	Y A	Q A	F S	S	A

TG G C CA G TA C T C T CG G A G T CG A CG G A A A CA A C T TG T C CA G A G TA G CA T TA CA A G C CA T C T C T CA A G TG C C CA CA G G G T C TG A CA C T T CA G C T TA CG C T CA A G C C T T C T C TA G TG C L A	S	T	L G	V	D G	N	N	L S	R	V	A	L	Q A	I	S	Q V	P	T G	S	D	T S	A	Y A	Q A	F S	S	A

TG G C CA G TA C T C T CG G A G T CG A CG G A A A CA A C T TG T C CA G A G TA G CA T TA CA A G C CA T C T C T CA A G TG C C CA CA G G G T C TG A CA C T T CA G C T TA CG C T CA A G C C T T C T C TA G TG C L A	S	T	L G	V	D G	N	N	L S	R	V	A	L	Q A	I	S	Q V	P	T G	S	D	T S	A	Y A	Q A	F S	S	A

TG G C CA G TA C T C T CG G A G T CG A CG G A A A CA A C T TG T C CA G A G TA G CA T TA CA A G C CA T C T C T CA A G TG C C CA CA G G G T C TG A CA C T T CA G C T TA CG C T CA A G C C T T C T C TA G TG C L A	S	T	L G	V	D G	N	N	L S	R	V	A	L	Q A	I	S	Q V	P	T G	S	D	T S	A	Y A	Q A	F S	S	A

TG G C CA G TA C T C T CG G A G T CG A CG G A A A CA A C T TG T C CA G A G TA G CA T TA CA A G C CA T C T C T CA A G TG C C CA CA G G G T C TG A CA C T T CA G C T TA CG C T CA A G C C T T C T C TA G TG C L A	S	T	L G	V	D G	N	N	L S	R	V	A	L	Q A	I	S	Q V	P	T G	S	D	T S	A	Y A	Q A	F S	S	A

TG G C CA G TA C T C T CG G A G T CG A CG G A A A CA A C T TG T C CA G A G TA G CA T TA CA A G C CA T C T C T CA A G TG C C CA CA G G G T C TG A CA C T T CA G C T TA CG C T CA A G C C T T C T C TA G TG C L A	S	T	L G	V	D G	N	N	L S	R	V	A	L	Q A	I	S	Q V	P	T G	S	D	T S	A	Y A	Q A	F S	S	A

TG G C CA G TA C T C T CG G A G T CG A CG G A A A CA A C T TG T C CA G A G TA G CA T TA CA A G C CA T C T C T CA A G TG C C CA CA G G G T C TG A CA C T T CA G C T TA CG C T CA A G C C T T C T C TA G TG C L A	S	T	L G	V	D G	N	N	L S	R	V	A	L	Q A	I	S	Q V	P	T G	S	D	T S	A	Y A	Q A	F S	S	A

Aar_R_10	TG G C CA G TA C T C T CG G A G T CG A CG G A A A CA A C T TG T C CA G A G TA G CA T TA CA A G C CA T C T C T CA A G TG C C CA CA G G G T C TG A CA C T T CA G C T TA CG C T CA A G C C T T C T C TA G TG C

Frame 1

L A	S	T	L G	V	D G	N	N	L S	R	V	A	L	Q A	I	S	Q V	P	T G	S	D	T S	A	Y A	Q A	F S	S	A

Aar_R_11	TG G C CA G TA C T C T CG G A G T CG A CG G A A A CA A C T TG T C CA G A G TA G CA T TA CA A G C CA T C T C T CA A G TG C C CA CA G G G T C TG A CA C T T CA G C T TA CG C T CA A G C C T T C T C TA G TG C

Frame 1

L A	S	T	L G	V	D G	N	N	L S	R	V	A	L	Q A	I	S	Q V	P	T G	S	D	T S	A	Y A	Q A	F S	S	A

Aar_R_12	TG G C CA G TA C T C T CG G A G T CG A CG G A A A CA A C T TG T C CA G A G TA G CA T TA CA A G C CA T C T C T CA A G TG C C CA CA G G G T C TG A CA C T T CA G C T TA CG C T CA A G C C T T C T C TA G TG C

Frame 1

L A	S	T	L G	V	D G	N	N	L S	R	V	A	L	Q A	I	S	Q V	P	T G	S	D	T S	A	Y A	Q A	F S	S	A

Aar_R_13	TG G C CA G TA C T C T CG G A G T CG A CG G A A A CA A C T TG T C CA G A G TA G CA T TA CA A G C CA T C T C T CA A G TG C C CA CA G G G T C TG A CA C T T CA G C T TA CG C T CA A G C C T T C T C TA G TG C

Frame 1

L A	S	T	L G	V	D G	N	N	L S	R	V	A	L	Q A	I	S	Q V	P	T G	S	D	T S	A	Y A	Q A	F S	S	A

Aar_R_14	TG G C CA G TA C T C T CG G A G T CG A CG G A A A CA A C T TG T C CA G A G TA G CA T TA CA A G C CA T C T C T CA A G TG C C CA CA G G G T C TG A CA C T T CA G C T TA CG C T CA A G C C T T C T C TA G TG C

Frame 1

L A	S	T	L G	V	D G	N	N	L S	R	V	A	L	Q A	I	S	Q V	P	T G	S	D	T S	A	Y A	Q A	F S	S	A

Aar_R_15	TG G C CA G TA C T C T CG G A G T CG A CG G A A A CA A C T TG T C CA G A G TA G CA T TA CA A G C CA T C T C T CA A G TG C C CA CA G G G T C TG A CA C T T CA G C T TA CG C T CA A G C C T T C T C TA G TG C

Frame 1

L A	S	T	L G	V	D G	N	N	L S	R	V	A	L	Q A	I	S	Q V	P	T G	S	D	T S	A	Y A	Q A	F S	S	A

Aar_R_16	TG G C CA G TA C T C T CG G A G T CG A CG G A A A CA A C T TG T C CA G A G TA G CA T TA CA A G C CA T C T C T CA A G TG C C CA CA G G G T C TG A CA C T T CA G C T TA CG C T CA A G C C T T C T C TA G TG C

Frame 1

L A	S	T	L G	V	D G	N	N	L S	R	V	A	L	Q A	I	S	Q V	P	T G	S	D	T S	A	Y A	Q A	F S	S	A

Aar_R_17	TG G C CA G TA C T C T CG G A G T CG A CG G A A A CA A C T TG T C CA G A G TA G CA T TA CA A G C CA T C T C T CA A G TG C C CA CA G G G T C TG A CA C T T CA G C T TA CG C T CA A G C C T T C T C TA G TG C

Frame 1

L A	S	T	L G	V	D G	N	N	L S	R	V	A	L	Q A	I	S	Q V	P	T G	S	D	T S	A	Y A	Q A	F S	S	A

Aar_R_18	TG G C CA G TA C T C T CG G A G T CG A CG G A A A CA A C T TG T C CA G A G TA G CA T TA CA A G C CA T C T C T CA A G TG C C CA CA G G G T C TG A CA C T T CA G C T TA CG C T CA A G C C T T C T C TA G TG C

Frame 1

L A	S	T	L G	V	D G	N	N	L S	R	V	A	L	Q A	I	S	Q V	P	T G	S	D	T S	A	Y A	Q A	F S	S	A

Aar_R_19	TG G C CA G TA C T C T CG G A G T CG A CG G A A A CA A C T TG T C CA G A G TA G CA T TA CA A G C CA T C T C T CA A G TG C C CA CA G G G T C TG A CA C T T CA G C T TA CG C T CA A G C C T T C T C TA G TG C

Frame 1

L A	S	T	L G	V	D G	N	N	L S	R	V	A	L	Q A	I	S	Q V	P	T G	S	D	T S	A	Y A	Q A	F S	S	A

Aar_R_20	TG G C CA G TA C T C T CG G A G T CG A CG G A A A CA A C T TG T C CA G A G TA G CA T TA CA A G C CA T C T C T CA A G TG C C CA CA G G G T C TG A CA C T T CA G C T TA CG C T CA A G C C T T C T C TA G TG C

Frame 1

At_R_1

Frame 1

At_R_2

Frame 1

At_R_3

Frame 1

At_R_4

Frame 1

At_R_5

Frame 1

At_R_6

Frame 1

At_R_7

Frame 1

At_R_8

Frame 1

At_R_9

Frame 1

At_R_10

Frame 1

At_R_11

Frame 1

At_R_12

Frame 1

At_R_13

Frame 1

At_R_14

Frame 1

Av_R_1

Frame 1

Av_R_2

Frame 1

Av_R_3

Frame 1

Av_R_4

Frame 1

Av_R_5

Frame 1

Av_R_6

Frame 1

Av_R_7

Frame 1

Av_R_8

Frame 1

Av_R_9

Frame 1

Av_R_10

Frame 1

L A	S	T	L G	V	D G	N	N	L S	R	V	A	L	Q A	I	S	Q V	P	T G	S	D	T S	A	Y A	Q A	F S	S	A

N N N N N N N N N N N N N N N N N N N N N N N N N N N N N N N N N N N N N N N N N N N N N N N N N N N N N N N N N N N N N N N N N N N N N N N N N N N N N N N N N N N N N N N N N N N N N N N N N N N N N N N N N N N T C TA G TG C

?	?	?	?	?	?	?	?	?	?	?	?	?	?	?	?	?	?	?	?	?	?	?	?	?	?	?	?	?	?	?	?	?	?	?	? S	S	A

TG G C CA G TA C T C T CG G A G T CG A CG G A A A TA A C T TG G C CA G A T T CG CG G TA CA G G C CG T C T C T CG A C TG C C CG C CG G A T CA G A CA C T T C TG C T TA CG C T CA A G CA T T C T C TA G TG C L A	S	T	L G	V	D G	N	N	L A	R	F A	V	Q A	V	S	R	L	P A	G	S	D	T S	A	Y A	Q A	F S	S	A

TG G C CA G TA C T C T CG G A G T CG A CG G A A A TA A C T TG G C CA G A T T CG CG G TA CA G G C CG T C T C T CG A C TG C C CG C CG G A T CA G A CA C T T C TG C T TA CG C T CA A G CA T T C T C TA G TG C L A	S	T	L G	V	D G	N	N	L A	R	F A	V	Q A	V	S	R	L	P A	G	S	D	T S	A	Y A	Q A	F S	S	A

TG G C CA G TA C T C T CG G A G T CG A CG G A A A TA A C T TG G C CA G A T T CG CG G TA CA G G C CG T C T C T CG A C TG C C CG C CG G A T CA G A CA C T T C TG C T TA CG C T CA A G CA T T C T C TA G TG C L A	S	T	L G	V	D G	N	N	L A	R	F A	V	Q A	V	S	R	L	P A	G	S	D	T S	A	Y A	Q A	F S	S	A

TG G C CA G TA C T C T CG G A G T CG A CG G A A A TA A C T TG G C CA G A T T CG CG G TA CA G G C CG T C T C T CG A C TG C C CG C CG G A T CA G A CA C T T C TG C T TA CG C T CA A G CA T T C T C TA G TG C L A	S	T	L G	V	D G	N	N	L A	R	F A	V	Q A	V	S	R	L	P A	G	S	D	T S	A	Y A	Q A	F S	S	A

TG G C CA G TA C T C T CG G A G T CG A CG G A A A TA A C T TG G C CA G A T T CG CG G TA CA G G C CG T C T C T CG A C TG C C CG C CG G A T CA G A CA C T T C TG C T TA CG C T CA A G CA T T C T C TA G TG C L A	S	T	L G	V	D G	N	N	L A	R	F A	V	Q A	V	S	R	L	P A	G	S	D	T S	A	Y A	Q A	F S	S	A

TG G C CA G TA C T C T CG G A G T CG A CG G A A A TA A C T TG G C CA G A T T CG CG G TA CA G G C CG T C T C T CG A C TG C C CG C CG G A T CA G A CA C T T C TG C T TA CG C T CA A G CA T T C T C TA G TG C L A	S	T	L G	V	D G	N	N	L A	R	F A	V	Q A	V	S	R	L	P A	G	S	D	T S	A	Y A	Q A	F S	S	A

TG G C CA G TA C T C T CG G A G T CG A CG G A A A TA A C T TG G C CA G A T T CG CG G TA CA G G C CG T C T C T CG A C TG C C CG C CG G A T CA G A CA C T T C TG C T TA CG C T CA A G CA T T C T C TA G TG C L A	S	T	L G	V	D G	N	N	L A	R	F A	V	Q A	V	S	R	L	P A	G	S	D	T S	A	Y A	Q A	F S	S	A

TG G C CA G TA C T C T CG G A G T CG A CG G A A A TA A C T TG G C CA G A T T CG CG G TA CA G G C CG T C T C T CG A C TG C C CG C CG G A T CA G A CA C T T C TG C T TA CG C T CA A G CA T T C T C TA G TG C L A	S	T	L G	V	D G	N	N	L A	R	F A	V	Q A	V	S	R	L	P A	G	S	D	T S	A	Y A	Q A	F S	S	A

TG G C CA G TA C T C T CG G A G T CG A CG G A A A TA A C T TG G C CA G A T T CG CG G TA CA G G C CG T C T C T CG A C TG C C CG C CG G A T CA G A CA C T T C TG C T TA CG C T CA A G CA T T C T C TA G TG C L A	S	T	L G	V	D G	N	N	L A	R	F A	V	Q A	V	S	R	L	P A	G	S	D	T S	A	Y A	Q A	F S	S	A

TG G C CA G TA C T C T CG G A G T CG A CG G A A A TA A C T TG G C CA G A T T CG CG G TA CA G G C CG T C T C T CG A C TG C C CG C CG G A T CA G A CA C T T C TG C T TA CG C T CA A G CA T T C T C TA G TG C L A	S	T	L G	V	D G	N	N	L A	R	F A	V	Q A	V	S	R	L	P A	G	S	D	T S	A	Y A	Q A	F S	S	A

TG G C CA G TA C T C T CG G A G T CG A CG G A A A TA A C T TG G C CA G A T T CG CG G TA CA G G C CG T C T C T CG A C TG C C CG C CG G A T CA G A CA C T T C TG C T TA CG C T CA A G CA T T C T C TA G TG C L A	S	T	L G	V	D G	N	N	L A	R	F A	V	Q A	V	S	R	L	P A	G	S	D	T S	A	Y A	Q A	F S	S	A

TG G C CA G TA C T C T CG G A G T CG A CG G A A A TA A C T TG G C CA G A T T CG CG G TA CA G G C CG T C T C T CG A C TG C C CG C CG G A T CA G A CA C T T C TG C T TA CG C T CA A G CA T T C T C TA G TG C L A	S	T	L G	V	D G	N	N	L A	R	F A	V	Q A	V	S	R	L	P A	G	S	D	T S	A	Y A	Q A	F S	S	A

TG G C CA G TA C T C T CG G A G T CG A CG G A A A TA A C T TG G C CA G A T T CG CG G TA CA G G C CG T C T C T CG A C TG C C CG C CG G A T CA G A CA C T T C TG C T TA CG C T CA A G CA T T C T C TA G TG C L A	S	T	L G	V	D G	N	N	L A	R	F A	V	Q A	V	S	R	L	P A	G	S	D	T S	A	Y A	Q A	F S	S	A

N N N N N N N N N N N N N N N N N N N N N N N N N N N N N N N N N N N N N N N N N N N N N N N N N N N N N N N N N N N N N N N N N N N N N N N N N N N N N N N N N N N N N N N N N N N N N N N N N N N N N N N N N N N N N N N N N N N

? ? ? ? ? ? ? ? ? ? ? ? ? ? ? ? ? ? ? ? ? ? ? ? ? ? ? ? ? ? ? ? ? ? ? ? ? ? ?

TG G C TA G TA A T C T CG G CA T CG A CG G A A A TA A C T TG T C CA G A A TA G CG T TA CA A G C CA T C T C T CA A G T C C C CG CG G G T T C TG A CA C T T C TG C T TA CG C T CA A G CA T T T T C TA C TG C L A	S	N	L G	I	D G	N	N	L S	R	I	A	L	Q A	I	S	Q V	P A	G	S	D	T S	A	Y A	Q A	F S	T A

TG G C TA G TA A T C T CG G CA T CG A CG G A A A TA A C T TG T C CA G A A TA G CG T TA CA A G C CA T C T C T CA A G T C C C CG CG G G T T C TG A CA C T T C TG C T TA CG C T CA A G CA T T T T C TA C TG C L A	S	N	L G	I	D G	N	N	L S	R	I	A	L	Q A	I	S	Q V	P A	G	S	D	T S	A	Y A	Q A	F S	T A

TG G C TA G TA A T C T CG G CA T CG A CG G A A A TA A C T TG T C CA G A A TA G CG T TA CA A G C CA T C T C T CA A G T C C C CG CG G G T T C TG A CA C T T C TG C T TA CG C T CA A G CA T T T T C TA C TG C L A	S	N	L G	I	D G	N	N	L S	R	I	A	L	Q A	I	S	Q V	P A	G	S	D	T S	A	Y A	Q A	F S	T A

TG G C TA G TA A T C T CG G CA T CG A CG G A A A TA A C T TG T C CA G A A TA G CG T TA CA A G C CA T C T C T CA A G T C C C CG CG G G T T C TG A CA C T T C TG C T TA CG C T CA A G CA T T T T C TA C TG C L A	S	N	L G	I	D G	N	N	L S	R	I	A	L	Q A	I	S	Q V	P A	G	S	D	T S	A	Y A	Q A	F S	T A

TG G C TA G TA A T C T CG G C C T CG A CG G A A A TA A C T TG T C CA G A A TA G CG T TA CA A G C CA T C T C T CA A G T C C C CG CG G G T T T TG A CG C T T C TG C T TA CG C T CA A G CA T T T T C TA C TG C L A	S	N	L G	L	D G	N	N	L S	R	I	A	L	Q A	I	S	Q V	P A	G	F	D A	S	A	Y A	Q A	F S	T A

TG G C TA G TA A T C T CG G CA T CG A CG G A A A TA A C T TG T C CA G A A TA G CG T TA CA A G C CA T C T C T CA A G T C C C CG CG G G T T C TG A CA C T T C TG C T TA CG C T CA A G CA T T T T C TA C TG C L A	S	N	L G	I	D G	N	N	L S	R	I	A	L	Q A	I	S	Q V	P A	G	S	D	T S	A	Y A	Q A	F S	T A

TG G C TA G TA A T C T CG G CA T CG A CG G A A A TA A C T TG T C CA G A A TA G CG T TA CA A G C CA T C T C T CA A G T C C C CG CG G G T T C TG A CA C T T C TG C T TA CG C T CA A G CA T T T T C TA C TG C L A	S	N	L G	I	D G	N	N	L S	R	I	A	L	Q A	I	S	Q V	P A	G	S	D	T S	A	Y A	Q A	F S	T A

TG G C TA G TA A T C T CG G CA T CG A CG G A A A TA A C T TG T C CA G A A TA G CG T TA CA A G C CA T C T C T CA A G T C C C CG CG G G T T C TG A CA C T T C TG C T TA CG C T CA A G CA T T T T C TA C TG C L A	S	N	L G	I	D G	N	N	L S	R	I	A	L	Q A	I	S	Q V	P A	G	S	D	T S	A	Y A	Q A	F S	T A

TG G C TA G TA A T C T CG G CA T CG A CG G A A A TA A C T TG T C CA G A A TA G CG T TA CA A G C CA T C T C T CA A G T C C C CG CG G G T T C TG A CA C T T C TG C T TA CG C T CA A G CA T T T T C TA C TG C L A	S	N	L G	I	D G	N	N	L S	R	I	A	L	Q A	I	S	Q V	P A	G	S	D	T S	A	Y A	Q A	F S	T A

Aam_R_1	N N N N N N N N N N N N N N N N N N N N N N N N N N N N N N N N N N N N N N N N N N N N N N N N N N N N N N N N N N N N N N N N N N N N N N N N N N N N N N N N N N N N N N N N N N N N N N N N N N N N N N N N N N N N N N N N N N N

Frame 1

? ? ? ? ? ? ? ? ? ? ? ? ? ? ? ? ? ? ? ? ? ? ? ? ? ? ? ? ? ? ? ? ? ? ? ? ? ? ?

Aam_R_2	TG G C CG G TA C T C T CG G A G T CG A CG G A A A TA G C T TG T C CA G A A TA G CG T TA CA G G C CA T C T C T CA A T TG C C CG G A G G G T CA G A CA C T T C TG C T TA CG C T CA A G CA T T C T C TA G TG C

Frame 1

L A	G	T	L G	V	D G	N	S	L S	R	I	A	L	Q A	I	S	Q	L	P G	G	S	D	T S	A	Y A	Q A	F S	S	A

Aam_R_3	TG G C CG G TA C T C T CG G A G T CG A CG G A A A CA G C T TG T C CA G A A TA G CG T TA CA G G C CA T C T C T CA A T TG C C CG CA G G G T C TG A CA C T T C TG C T TA CG C T CA A G CA T T C T C TA G TG C

Frame 1

L A	G	T	L G	V	D G	N	S	L S	R	I	A	L	Q A	I	S	Q	L	P A	G	S	D	T S	A	Y A	Q A	F S	S	A

Aam_R_4	TG G C CG G TA C T C T CG G A G T CG A CG G A A A CA G C T TG T C CA G A A TA G CG T TA CA G G C CA T C T C T CA A T TG C C CG CA G G G T C TG A CA C T T C TG C T TA CG C T CA A G CA T T C T C TA G TG C

Frame 1

L A	G	T	L G	V	D G	N	S	L S	R	I	A	L	Q A	I	S	Q	L	P A	G	S	D	T S	A	Y A	Q A	F S	S	A

Aam_R_5	TG G C CG G TA C T C T CG G A G T CG A CG G A A A CA G C T TG T C CA G A A TA G CG T TA CA G G C CA T C T C T CA A T TG C C CG CA G G G T C TG A CA C T T C TG C T TA CG C T CA A G CA T T C T C TA G TG C

Frame 1

L A	G	T	L G	V	D G	N	S	L S	R	I	A	L	Q A	I	S	Q	L	P A	G	S	D	T S	A	Y A	Q A	F S	S	A

Aam_R_6	TG G C CG G TA C T C T CG G A G T CG A CG G A A A CA G C T TG T C CA G A A TA G CG T TA CA G G C CA T C T C T CA A T TG C C CG CA G G G T C TG A CA C T T C TG C T TA CG C T CA A G CA T T C T C TA G TG C

Frame 1

L A	G	T	L G	V	D G	N	S	L S	R	I	A	L	Q A	I	S	Q	L	P A	G	S	D	T S	A	Y A	Q A	F S	S	A

Aar_R_1

Frame 1

Aar_R_2

Frame 1

Aar_R_3

Frame 1

Aar_R_4

Frame 1

Aar_R_5

Frame 1

Aar_R_6

Frame 1

Aar_R_7

Frame 1

Aar_R_8

Frame 1

Aar_R_9

Frame 1

240	250	260	270	280	290	300	310	320	330	340

G C T C T T CA A TG C CG G A G T T C T CA A CG CA A G CA A CA T TG A CA CA T TG G G A T C C CG A G T T C T C T CA G C TG T T T TG A A CG G A G TA T CA A G TG CG G CG CA A G G T C T TG G CA T CA A TG TA L	F N	A	G	V	L N	A	S	N	I	D	T	L G	S	R	V	L S	A	V	L N	G	V	S	S	A	A	Q G	L G	I	N	V

G C T C T T CA A TG C CG G A G T T C T CA A CG CA A G CA A CA T TG A CA CA T TG G G A T C C CG A G T T C T C T CA G C TG T T T TG A A CG G A G TA T CA A G TG CG G CG CA A G G T C T TG G CA T CA A TG TA L	F N	A	G	V	L N	A	S	N	I	D	T	L G	S	R	V	L S	A	V	L N	G	V	S	S	A	A	Q G	L G	I	N	V

G C T C T T CA A TG C CG G A G T T C T CA A CG CA A G CA A CA T TG A CA CA T TG G G A T C C CG A G T T C T C T CA G C TG T T T TG A A CG G A G TA T CA A G TG CG G CG CA A G G T C T TG G CA T CA A TG TA L	F N	A	G	V	L N	A	S	N	I	D	T	L G	S	R	V	L S	A	V	L N	G	V	S	S	A	A	Q G	L G	I	N	V

G C T C T T CA A TG C CG G A G T T C T CA A CG CA A G CA A CA T TG A CA CA T TG G G A T C C CG A G T T C T C T CA G C TG T T T TG A A CG G A G TA T CA A G TG CG G CG CA A G G T C T TG G CA T CA A TG TA L	F N	A	G	V	L N	A	S	N	I	D	T	L G	S	R	V	L S	A	V	L N	G	V	S	S	A	A	Q G	L G	I	N	V

G C T C T T CA A TG C CG G A A T T C T CA A TG CA A G CA A CA T TG A CA CA T TG G G A T C C CG A G T T C T C T CA G C TG T T T TG A A CG G A G TA T CA A G TG CG G CG CA A G G T C T TG G CA T CA A TG TA L	F N	A	G	I	L N	A	S	N	I	D	T	L G	S	R	V	L S	A	V	L N	G	V	S	S	A	A	Q G	L G	I	N	V

G C T C T T CA A TG C CG G A G T T C T CA A CG CA A G CA A CA T TG A CA CA T TG G G A T C C CG A G T T C T C T CA G C TG T T T TG A A CG G A G TA T CA A G TG CG G CG CA A G G T C T TG G CA T CA A TG TA L	F N	A	G	V	L N	A	S	N	I	D	T	L G	S	R	V	L S	A	V	L N	G	V	S	S	A	A	Q G	L G	I	N	V

G C T C T T CA A TG C CG G A G T T C T CA A TG CA A G CA A CA T TG A CA CA T TG G G A T C C CG A G T T C T C T CA G C TG T T T TG A A CG G A G TA T CA A G TG CG G CG CA A G G T C T TG G CA T CA A TG TA L	F N	A	G	V	L N	A	S	N	I	D	T	L G	S	R	V	L S	A	V	L N	G	V	S	S	A	A	Q G	L G	I	N	V

A C T C T T CA A TG C CG G A G T T C T CA A TG CA A G CA A CA T TG A CA CA T TG G G A T C C CG A G T T C T C T CA G C TG T T T TG A A CG G A G TA T CA A G TG CG G CG CA A G G T C T TG G CA T CA A TG TA L	F N	A	G	V	L N	A	S	N	I	D	T	L G	S	R	V	L S	A	V	L N	G	V	S	S	A	A	Q G	L G	I	N	V

G C T C T T CA A TG C CG G A G T T C T CA A TG CA A G CA A CA T TG A CA CA T TG G G A T C C CG A G T T C T C T CA G C TG T T T TG A A CG G A G TA T CA A G TG CG G CG CA A G G T C T TG G CA T CA A TG TA L	F N	A	G	V	L N	A	S	N	I	D	T	L G	S	R	V	L S	A	V	L N	G	V	S	S	A	A	Q G	L G	I	N	V

Aar_R_10	G C T C T T CA A TG C CG G A G T T C T CA A TG CA A G CA A CA T TG A CA CA T TG G G A T C C CG A G T T C T C T CA G C TG T T T TG A A CG G A G TA T CA A G TG CG G CG CA A G G T C T TG G CA T CA A TG TA

Frame 1

L	F N	A	G	V	L N	A	S	N	I	D	T	L G	S	R	V	L S	A	V	L N	G	V	S	S	A	A	Q G	L G	I	N	V

Aar_R_11	A C T C T T CA A TG C CG G A G T T C T CA A TG CA A G CA A CA T TG A CA CA T TG G G A T C C CG A G T T C T C T CA G C TG T T T TG A A CG G A G TA T CA A G TG CG G CG CA A G G T C T TG G CA T CA A TG TA

Frame 1

L	F N	A	G	V	L N	A	S	N	I	D	T	L G	S	R	V	L S	A	V	L N	G	V	S	S	A	A	Q G	L G	I	N	V

Aar_R_12	G C T C T T CA A TG C CG G A G T T C T CA A TG CA A G CA A CA T TG A CA CA T TG G G A T C C CG A G T T C T C T CA G C TG T T T TG A A CG G A G TA T CA A G TG CG G CG CA A G G T C T TG G CA T CA A TG TA

Frame 1

L	F N	A	G	V	L N	A	S	N	I	D	T	L G	S	R	V	L S	A	V	L N	G	V	S	S	A	A	Q G	L G	I	N	V

Aar_R_13	G C T C T T CA A TG C CG G A G T T C T CA A TG CA A G CA A CA TA G A CA CA T TG G G A T C C CG A G T T C T C T CA G C TG T T T TG A A CG G A G TA T CA A G TG CG G CG CA A G G T C T TG G CA T CA A TG TA

Frame 1

L	F N	A	G	V	L N	A	S	N	I	D	T	L G	S	R	V	L S	A	V	L N	G	V	S	S	A	A	Q G	L G	I	N	V

Aar_R_14	A C T C T T CA A TG C CG G A G T T C T CA A TG CA A G CA A CA T TG A CA CA T TG G G A T C C CG A G T T C T C T CA G C TG T T T TG A A CG G A G TA T CA A G TG CG G CG CA A G G T C T TG G CA T CA A TG TA

Frame 1

L	F N	A	G	V	L N	A	S	N	I	D	T	L G	S	R	V	L S	A	V	L N	G	V	S	S	A	A	Q G	L G	I	N	V

Aar_R_15	G C T C T T CA A TG C CG G A G T T C T CA A TG CA A G CA A CA T TG A CA CA T TG G G A T C C CG A G T T C T C T CA G C TG T T T TG A A CG G A G TA T CA A G TG CG G CG CA A G G T C T TG G CA T CA A TG TA

Frame 1

L	F N	A	G	V	L N	A	S	N	I	D	T	L G	S	R	V	L S	A	V	L N	G	V	S	S	A	A	Q G	L G	I	N	V

Aar_R_16	G C T C T T CA A TG C CG G A G T T C T C TA TG CA A G CA A CA T TG A CA CA T TG G G A T C C CG A G T T C T C T CA G C TG T T T TG A A CG G A G TA T CA A G TG CG G CG CA A G G T C T TG G CA T CA A TG TA

Frame 1

L	F N	A	G	V	L	Y A	S	N	I	D	T	L G	S	R	V	L S	A	V	L N	G	V	S	S	A	A	Q G	L G	I	N	V

Aar_R_17	G C T C T T CA A TG C CG G A G T T C T CA A TG CA A G CA A CA T TG A CA CA T TG G G A T C C CG A G T T C T C T CA G C TG T T T TG A A CG G A G TA T CA A G TG CG G CG CA A G G T C T TG G CA T CA A TG TA

Frame 1

L	F N	A	G	V	L N	A	S	N	I	D	T	L G	S	R	V	L S	A	V	L N	G	V	S	S	A	A	Q G	L G	I	N	V

Aar_R_18	G C T C T T CA A TG C CG G A G T T C T CA A TG CA A G CA A CA T TG A CA CA T TG G G A T C C CG A G T T C T C T CA G C TG T T T TG A A CG G A G TA T CA A G TG CG G CG CA A G G T C T TG G CA T CA A TG TA

Frame 1

L	F N	A	G	V	L N	A	S	N	I	D	T	L G	S	R	V	L S	A	V	L N	G	V	S	S	A	A	Q G	L G	I	N	V

Aar_R_19	G C T C T T CA A TG C CG G A G T T C T CA A TG CA A G CA A CA T TG A CA CA T TG G G A T C C CG A G T T C T C T CA G C TG T T T TG A A CG G A G TA T CA A G TG CG G CG CA A G G T C T TG G CA T CA A TG TA

Frame 1

L	F N	A	G	V	L N	A	S	N	I	D	T	L G	S	R	V	L S	A	V	L N	G	V	S	S	A	A	Q G	L G	I	N	V

Aar_R_20	A C T C T T CA A TG C CG G A G T T C T CA A TG CA A G CA A CA T TG A CA CA T TG G G A T C C CG A G T T C T C T CA G C TG T T T TG A A CG G A G TA T CA A G TG CG G CG CA A G G T C T TG G CA T CA A TG TA

Frame 1

At_R_1

Frame 1

At_R_2

Frame 1

At_R_3

Frame 1

At_R_4

Frame 1

At_R_5

Frame 1

At_R_6

Frame 1

At_R_7

Frame 1

At_R_8

Frame 1

At_R_9

Frame 1

At_R_10

Frame 1

At_R_11

Frame 1

At_R_12

Frame 1

At_R_13

Frame 1

At_R_14

Frame 1

Av_R_1

Frame 1

Av_R_2

Frame 1

Av_R_3

Frame 1

Av_R_4

Frame 1

Av_R_5

Frame 1

Av_R_6

Frame 1

Av_R_7

Frame 1

Av_R_8

Frame 1

Av_R_9

Frame 1

Av_R_10

Frame 1

L	F N	A	G	V	L N	A	S	N	I	D	T	L G	S	R	V	L S	A	V	L N	G	V	S	S	A	A	Q G	L G	I	N	V

G C T C T T CA A TG C CG G A G T T C T CA A TG CA A G CA A CA T TG A CA CA T TG G G A T C C CG A G T T C T C T CA G CA C T T T TG A A CG G A G TA T CA A G TG CG G CG CA A G G T C T TG G CA T CA A TG TA L	F N	A	G	V	L N	A	S	N	I	D	T	L G	S	R	V	L S	A	L	L N	G	V	S	S	A	A	Q G	L G	I	N	V

G C T C T T CA A TG C CG G A G T T C T CA A TG CA A G CA A CA T TG A CA CA T TG G G A T C C CG A G T T C T C T CA G CA C T T T TG A A CG G A G TA T CA A G TG CG G CG CA A G G T C T TG G CA T CA A TG TA L	F N	A	G	V	L N	A	S	N	I	D	T	L G	S	R	V	L S	A	L	L N	G	V	S	S	A	A	Q G	L G	I	N	V

G C T C T T CA A TG C CG G A G T T C T CA A TG CA A G CA A CA T TG A CA CA T TG G G A T C C CG A G T T C T C T CA G CA C T T T TG A A CG G A G TA T CA A G TG CG G CG CA A G G T C T TG G CA T CA A TG TA L	F N	A	G	V	L N	A	S	N	I	D	T	L G	S	R	V	L S	A	L	L N	G	V	S	S	A	A	Q G	L G	I	N	V

G C T C T T CA A TG C CG G A G T T C T CA A TG CA A G CA A CA T TG A CA CA T TG G G A T C C CG A G T T C T C T CA G CA C T T T TG A A CG G A G TA T CA A G TG CG G CG CA A G G T C T TG G CA T CA A TG TA L	F N	A	G	V	L N	A	S	N	I	D	T	L G	S	R	V	L S	A	L	L N	G	V	S	S	A	A	Q G	L G	I	N	V

G C T C T T CA A TG C CG G A G T T C T CA A TG CA A G CA A CA T TG A CA CA T TG G G A T C C CG A G T T C T C T CA G CA C T T T TG A A CG G A G TA T CA A G TG CG G CG CA A G G T C T TG G CA T CA A TG TA L	F N	A	G	V	L N	A	S	N	I	D	T	L G	S	R	V	L S	A	L	L N	G	V	S	S	A	A	Q G	L G	I	N	V

G C T C T T CA A TG C CG G A G T T C T CA A TG CA A G CA A CA T TG A CA CA T TG G G A T C C CG A G T T C T C T CA G CA C T T T TG A A CG G A G TA T CA A G TG CG G CG CA A G G T C T TG G CA T CA A TG TA L	F N	A	G	V	L N	A	S	N	I	D	T	L G	S	R	V	L S	A	L	L N	G	V	S	S	A	A	Q G	L G	I	N	V

G C T C T T CA A TG C CG G A G T T C T CA A TG CA A G CA A CA T TG A CA CA T TG G G A T C C CG A G T T C T C T CA G CA C T T T TG A A CG G A G TA T CA A G TG CG G CG CA A G G T C T TG G CA T CA A TG TA L	F N	A	G	V	L N	A	S	N	I	D	T	L G	S	R	V	L S	A	L	L N	G	V	S	S	A	A	Q G	L G	I	N	V

G C T C T T CA A TG C CG G A G T T C T CA A TG CA A G CA A CA T TG A CA CA T TG G G A T C C CG A G T T C T C T CA G CA C T T T TG A A CG G A G TA T CA A G TG CG G CG CA A G G T C T TG G CA T CA A TG TA L	F N	A	G	V	L N	A	S	N	I	D	T	L G	S	R	V	L S	A	L	L N	G	V	S	S	A	A	Q G	L G	I	N	V

G C T C T T CA A TG C CG G A G T T C T CA A TG CA A G CA A CA T TG A CA CA T TG G G A T C C CG A G T T C T C T CA G CA C T C T TG A A CG G A G TA T CA A G TG CG G CG CA A G G T C T TG G CA T CA A TG TA L	F N	A	G	V	L N	A	S	N	I	D	T	L G	S	R	V	L S	A	L	L N	G	V	S	S	A	A	Q G	L G	I	N	V

G C T C T T CA A TG C CG G A G T T C T CA A TG CA A G CA A CA T TG A CA CA T TG G G A T C C CG A G T T C T C T CA G CA C T T T TG A A CG G A G TA T CA A G TG CG G CG CA A G G T C T TG G CA T CA A TG TA L	F N	A	G	V	L N	A	S	N	I	D	T	L G	S	R	V	L S	A	L	L N	G	V	S	S	A	A	Q G	L G	I	N	V

G C T C T T CA A TG C CG G A G T T C T CA A TG CA A G CA A CA T TG A CA CA T TG G G A T C C CG A G T T C T C T CA G CA C T T T TG A A CG G A G TA T CA A G TG CG G CG CA A G G T C T TG G CA T CA A TG TA L	F N	A	G	V	L N	A	S	N	I	D	T	L G	S	R	V	L S	A	L	L N	G	V	S	S	A	A	Q G	L G	I	N	V

G C T C T T CA A TG C CG G A G T T C T CA A TG CA A G CA A CA T TG A CA CA T TG G G A T C C CG A G T T C T C T CA G CA C T T T TG A A CG G A G TA T CA A G TG CG G CG CA A G G T C T TG G CA T CA A TG TA L	F N	A	G	V	L N	A	S	N	I	D	T	L G	S	R	V	L S	A	L	L N	G	V	S	S	A	A	Q G	L G	I	N	V

G C T C T T CA A TG C CG G A G T T C T CA A TG CA A G CA A CA T TG A CA CA T TG G G A T C C CG A G T T C T C T CA G CA C T T T TG A A CG G A G TA T CA A G TG CG G CG CA A G G T C T TG G CA T CA A TG TA L	F N	A	G	V	L N	A	S	N	I	D	T	L G	S	R	V	L S	A	L	L N	G	V	S	S	A	A	Q G	L G	I	N	V

G C T C T T CA A TG C CG G A G T T C T CA A TG CA A G CA A CA T TG A CA CA T TG G G A T C C CG A G T T C T C T CA G CA C T T T TG A A CG G A G TA T CA A G TG CG G CG CA A G G T C T TG G CA T CA A TG TA L	F N	A	G	V	L N	A	S	N	I	D	T	L G	S	R	V	L S	A	L	L N	G	V	S	S	A	A	Q G	L G	I	N	V

N N N N N N N N N N N N N N N N N N N N N N N N N N N N N N N N N N N N N N N N N N N N N N N N N N N N N N N N N N N N N N N N N N N N N N N N N N N N N N N N N N N N N N N N N N N N N N N N N N N N N N N N N N N N N N N N N N N

? ? ? ? ? ? ? ? ? ? ? ? ? ? ? ? ? ? ? ? ? ? ? ? ? ? ? ? ? ? ? ? ? ? ? ? ? ?

C T TG G T CA C CG G TG G A G T T C TG A A CG CA A A CA A CG T TG A CA CA T TG G G A T C C CA A G TA C T C T CA G CA G T T T TG A A CG G A G TA T CA A G TG CG G CG CA A G G C C T TG G A A T CA A TG TA L V	T G	G	V	L N	A	N	N	V	D	T	L G	S	Q V	L S	A	V	L N	G	V	S	S	A	A	Q G	L G	I	N	V

C T TG G T CA C CG G TG G A G T T C TG A A CG CA A A CA A CG T TG A CA CA T TG G G A T C C CA A G TA C T C T CA G CA G T T T TG A A CG G A G TA T CA A G TG CG G CG CA A G G C C T TG G A A T CA A TG TA L V	T G	G	V	L N	A	N	N	V	D	T	L G	S	Q V	L S	A	V	L N	G	V	S	S	A	A	Q G	L G	I	N	V

C T TG G T CA C CG G TG G A G T T C TG A A CG CA A A CA A CG T TG A CA CA T TG G G A T C C CA A G TA C T C T CA G CA G T T T TG A A CG G A G TA T CA A G TG CG G CG CA A G G C C T TG G A A T CA A TG TA L V	T G	G	V	L N	A	N	N	V	D	T	L G	S	Q V	L S	A	V	L N	G	V	S	S	A	A	Q G	L G	I	N	V

C T TG G T CA C CG G TG G A G T T C TG A A CG CA A A CA A CG T TG A CA CA T TG G G A T C C CA A G TA C T C T CA G CA G T T T TG A A CG G A G TA T CA A G TG CG G CG CA A G G C C T TG G A A T CA A TG TA L V	T G	G	V	L N	A	N	N	V	D	T	L G	S	Q V	L S	A	V	L N	G	V	S	S	A	A	Q G	L G	I	N	V

C T TG G T CA C CG G TG G A G T T C TG A A CG CA A A CA A CG T TG A CA CA T TG G G A T C C CA A G TA C T C T CA G CA G T T T TG A A CG G A G TA T CA A G TG CG G CG CA A G G C C T TG G A A T CA A TG TA L V	T G	G	V	L N	A	N	N	V	D	T	L G	S	Q V	L S	A	V	L N	G	V	S	S	A	A	Q G	L G	I	N	V

C T TG G T CA C CG G TG G A G T T C TG A A CG CA A A CA A CG T TG A CA CA T TG G G A T C C CA A G TA C T C T CA G CA G T T T TG A G CG G A G TA T CA A G TG CG G CG CA A G G C C T TG G A A T CA A TG TA L V	T G	G	V	L N	A	N	N	V	D	T	L G	S	Q V	L S	A	V	L S	G	V	S	S	A	A	Q G	L G	I	N	V

C T TG G T CA C CG G TG G A G T T C TG A A CG CA A A CA A CG T TG A CA CA T TG G G A T C C CA A G TA C T C T CA G CA G T T T TG A A CG G A G TA T CA A G TG CG G CG CA A G G C C T TG G A A T CA A TG TA L V	T G	G	V	L N	A	N	N	V	D	T	L G	S	Q V	L S	A	V	L N	G	V	S	S	A	A	Q G	L G	I	N	V

C T TG G T CA C CG G TG G A G T T C TG A A CG CA A A CA A CG T TG A CA CA T TG G G A T C C CA A G TA C T C T CA G CA G T T T TG A A CG G A G TA T CA A G TG CG G CG CA A G G C C T TG G A A T CA A TG TA L V	T G	G	V	L N	A	N	N	V	D	T	L G	S	Q V	L S	A	V	L N	G	V	S	S	A	A	Q G	L G	I	N	V

C T TG G T CA C CG G TG G A G T T C TG A A CG CA A A CA A CG T TG A CA CA T TG G G A T C C CA A G TA C T C T CA G CA G T T T TG A A CG G A G TA T CA A G TG CG G CG CA A G G C C T TG G A A T CA A TG TA L V	T G	G	V	L N	A	N	N	V	D	T	L G	S	Q V	L S	A	V	L N	G	V	S	S	A	A	Q G	L G	I	N	V

Aam_R_1	N N N N N N N N N N N N N N N N N N N N N N N N N N N N N N N N N N N N N N N N N N N N N N N N N N N N N N N N N N N N N N N N N N N N N N N N N N N N N N N N N N N N N N N N N N N N N N N N N N N N N N N N N N N N N N N N N N N

Frame 1

? ? ? ? ? ? ? ? ? ? ? ? ? ? ? ? ? ? ? ? ? ? ? ? ? ? ? ? ? ? ? ? ? ? ? ? ? ?

Aam_R_2	G C TG T T CA A TG C CG G A G T T C TG A A TG CA A G CA A CA T TG A CA CA T TG G G A T C C CG A G T T C TG T CA G CA G T T T TG A A CG G A G TA T CA A G TG CG G C T CA A G G TA T TG G C C T CA A TG TA

Frame 1

L	F N	A	G	V	L N	A	S	N	I	D	T	L G	S	R	V	L S	A	V	L N	G	V	S	S	A	A	Q G	I	G	L N	V

Aam_R_3	G C TG G T CA A TG C CG G A G T T C TG A A TG CA A G CA A CA T TG A CA CA T TG G G A T C C CG A G T T C TG T CA G CA G T T T TG A A CG G A G TA T CA A G TG CG G C T CA A G G TA T TG G C C T CA A TG TA

Frame 1

L V	N	A	G	V	L N	A	S	N	I	D	T	L G	S	R	V	L S	A	V	L N	G	V	S	S	A	A	Q G	I	G	L N	V

Aam_R_4	G C TG G T CA A TG C CG G A G T T C TG A G TG CA A G CA A CA T TG A CA CA T TG G G A T C C CG A G T T C TG T CA G CA G T T T TG A A CG G A G TA T CA A G TG CG G C T CA A G G TA T TG G C C T CA A TG TA

Frame 1

L V	N	A	G	V	L S	A	S	N	I	D	T	L G	S	R	V	L S	A	V	L N	G	V	S	S	A	A	Q G	I	G	L N	V

Aam_R_5	G C TG G T CA A TG C CG G A G T T C TG A A TG CA A G CA A CA T TG A CA CA T TG G G A T C C CG A G T T C TG T CA G CA G T T T TG A A CG G A G TA T CA A G TG CG G C T CA A G G TA T TG G C C T CA A TG TA

Frame 1

L V	N	A	G	V	L N	A	S	N	I	D	T	L G	S	R	V	L S	A	V	L N	G	V	S	S	A	A	Q G	I	G	L N	V

Aam_R_6	G C TG G T CA A TG C CG G A G T T C TG A A TG CA A G CA A CA T TG A CA CA T TG G G A T C C CG A G T T C TG T CA G CA G T T T TG A A CG G A G TA T CA A G TG CG G C T CA A G G TA T TG G C C T CA A TG TA

Frame 1

L V	N	A	G	V	L N	A	S	N	I	D	T	L G	S	R	V	L S	A	V	L N	G	V	S	S	A	A	Q G	I	G	L N	V

Aar_R_1

Frame 1

Aar_R_2

Frame 1

Aar_R_3

Frame 1

Aar_R_4

Frame 1

Aar_R_5

Frame 1

Aar_R_6

Frame 1

Aar_R_7

Frame 1

Aar_R_8

Frame 1

Aar_R_9

Frame 1

350	360	370	380	390	400	410	420	430	440	450	460

G A TA C CG G CA G TG TA CA G A G TG A CA T TA G T T C CA G TA G CA G C T T C C T C T CA A C CA G C T CG T C TG CG T C CA G T T T C T C C T C T CA G G CA T CA - - - G C T T C T T C TA C CA G CG G TG C TG D	T G	S	V	Q S	D I	S	S	S	S	S	F	L S	T S	S	S	A	S	S	F S	S	Q	A	S - - - A	S	S	T S	G	A

G A TA C CG G TA G TG TA CA G A G TG A CA T TA G T T C CA G TA G CA G C T T C C T C T CA A C CA G C T CG T C TG CG T C CA G T T T C T C C T C T CA G G CA T CA - - - G C T T C T T C TA C CA G CG G TG C TG D	T G	S	V	Q S	D I	S	S	S	S	S	F	L S	T S	S	S	A	S	S	F S	S	Q	A	S - - - A	S	S	T S	G	A

G A TA C CG G CA G TG TA CA G A G TG A CA T TA G T T C CA G TA G CA G C T T C C T C T CA A C CA G C T CG T C TG CG T C CA G T T T C T C C T C T CA G G CA T CA - - - G C T T C T T C TA C CA G CG G TG C TG D	T G	S	V	Q S	D I	S	S	S	S	S	F	L S	T S	S	S	A	S	S	F S	S	Q	A	S - - - A	S	S	T S	G	A

G A TA C CG G CA G TG TA CA G A G TG A CA T TA G T T C CA G TA G CA G C T T C C T C T CA A C CA G C T CG T C TG CG T C CA G T T T C T C C T C T CA G G CA T CA - - - G C T T C T T C TA C CA G CG G TG C TG D	T G	S	V	Q S	D I	S	S	S	S	S	F	L S	T S	S	S	A	S	S	F S	S	Q	A	S - - - A	S	S	T S	G	A

G A TA C CG G CA G TG TA CA G A G TG A CA T TA G T T C CA G TA G CA G C T T C C T C T CA A C CA G C T CG T C TG CG T C CA G T T T C T C C T C T CA G G CA T CA - - - G C T T C T T C TA C CA G CG G TG C TG D	T G	S	V	Q S	D I	S	S	S	S	S	F	L S	T S	S	S	A	S	S	F S	S	Q	A	S - - - A	S	S	T S	G	A

G A TA C CG G CA G TG TA CA G A G A G A CA T TA G T T C CA G TA G CA G C T T C C T C T CA A C CA G C T CG T C TG CG T C CA G T T T C T C C T C T CA G G CA T CA - - - G C T T C T T C TA C CA G CG G TG C TG D	T G	S	V	Q R	D I	S	S	S	S	S	F	L S	T S	S	S	A	S	S	F S	S	Q	A	S - - - A	S	S	T S	G	A

G A TA C CG G CA G TG TA CA G A G TG A CA T TA G T T C CA G TA G CA G C T T C C T C T CA A C CA G C T CG T C TG CG T C CA G T T T C T C C T C T CA G G CA T CA - - - G C T T C T T C TA C CA G CG G TG C TG D	T G	S	V	Q S	D I	S	S	S	S	S	F	L S	T S	S	S	A	S	S	F S	S	Q	A	S - - - A	S	S	T S	G	A

G A TA C CG G CA G TG TA CA G A G TG A CA T TA G T T C CA G TA G CA G C T T C C T C T CA A C CA G C T CG T C TG CG T C CA G T T T C T C C T C T CA G G CA T CA - - - G C T T C T T C TA C CA G CG G TG C TG D	T G	S	V	Q S	D I	S	S	S	S	S	F	L S	T S	S	S	A	S	S	F S	S	Q	A	S - - - A	S	S	T S	G	A

G A TA C CG G CA G TG TA CA G A G TG A CA T TA G T T C CA G TA G CA G C T T C C T C T CA A C CA G C T CG T C TG CG T C CA G T T T C T C C T C T CA G G CA T CA - - - G C T T C T T C TA C CA G CG G TG C TG D	T G	S	V	Q S	D I	S	S	S	S	S	F	L S	T S	S	S	A	S	S	F S	S	Q	A	S - - - A	S	S	T S	G	A

Aar_R_10	A A TA C CG G CA G TG TA CA G A G TG A CA T TA G T T C CA G TA G TA G C T T C C T C T CA A C CA G C T CG T C TG CG T C CA G T T T C T C C T C T CA G G CA T CA - - - G C T T C T T C TA C CA G CG G TG C TG

Frame 1

N	T G	S	V	Q S	D I	S	S	S	S	S	F	L S	T S	S	S	A	S	S	F S	S	Q A	S - - - A	S	S	T S	G	A

Aar_R_11	G A TA C CG G CA G TG TG CA G A G TG A CA T TA G T T C CA G TA G CA G C T T C C T C T CA A C CA G C T CG T C TG CG T C CA G T T T C T C C T C T CA G G CA T CA - - - G C T T C T T C TA C CA G CG G TG C TG

Frame 1

D	T G	S	V	Q S	D I	S	S	S	S	S	F	L S	T S	S	S	A	S	S	F S	S	Q A	S - - - A	S	S	T S	G	A

Aar_R_12	G A TA C CG G CA G TG TA CA G A G TG A CA T TA G T T C CA G TA G CA G C T T C C T C T CA A C CA G C T CG T C TG CG T C CA G T T T C T C CA C T CA G G CA T CA - - - G C T T C T T C TA C CA G CG G TG C TG

Frame 1

D	T G	S	V	Q S	D I	S	S	S	S	S	F	L S	T S	S	S	A	S	S	F S	T	Q A	S - - - A	S	S	T S	G	A

Aar_R_13	G A TA C CG G CA G TG TG CA G A G TG A CA T TA G T T C CA G TA G CA G C T T C C T C T CA A C CA G C T CG T C TG CG T C CA G T T T C T C C T C T CA G G CA T CA - - - G C T T C T T C TA C CA G CG G TG C TG

Frame 1

D	T G	S	V	Q S	D I	S	S	S	S	S	F	L S	T S	S	S	A	S	S	F S	S	Q A	S - - - A	S	S	T S	G	A

Aar_R_14	G A TA C CG G CA G TG TG CA G A G TG A CA T TA G T T C CA G TA G CA G C T T C C T C T CA A C CA G C T CG T C TG CG T C CA G T T T C T C C T C T CA G G CA T CA - - - G C T T C T T C TA C CA G CG G TG C TG

Frame 1

D	T G	S	V	Q S	D I	S	S	S	S	S	F	L S	T S	S	S	A	S	S	F S	S	Q A	S - - - A	S	S	T S	G	A

Aar_R_15	G A TA C CG G CA G TG TG CA G A G TG A CA T TA G T T C CA G TA G CA G C T T C C T C T CA A C CA G C T CG T C TG CG T C CA G T T T C T C C T C T CA G G CA T CA - - - G C T T C T T C T T C CG G CG G TG C TG

Frame 1

D	T G	S	V	Q S	D I	S	S	S	S	S	F	L S	T S	S	S	A	S	S	F S	S	Q A	S - - - A	S	S	S	G	G	A

Aar_R_16	G A TA C CG G CA G TG TA CA G A G TG A CA T TA G T T C CA G TA G CA G C T T C C T C T CA A C CA G C T CG T C TG CG T C CA G T T T C T C C T C T CA G G CA T CA - - - G C T T C T T C TA C CA G CG G TG C TG

Frame 1

D	T G	S	V	Q S	D I	S	S	S	S	S	F	L S	T S	S	S	A	S	S	F S	S	Q A	S - - - A	S	S	T S	G	A

Aar_R_17	G A TA C CG G CA G TG TG CA G A G TG A CA T TA G T T C CA G TA G CA G C T T C C T C T CA A C CA G C T CG T C TG CG T C CA G T T T C T C C T C T CA G G CA T CA - - - G C T T C T T C TA C CA G CG G TG C TG

Frame 1

D	T G	S	V	Q S	D I	S	S	S	S	S	F	L S	T S	S	S	A	S	S	F S	S	Q A	S - - - A	S	S	T S	G	A

Aar_R_18	G A TA C CG G CA G TG TG CA G A G TG A CA T TA G T T C CA G TA G CA G C T T C C T C T CA A C CA G C T CG T C TG CG T C CA G T T T C T C C T C T CA G G CA T CA - - - G C T T C T T C TA C CA G CG G TG C TG

Frame 1

D	T G	S	V	Q S	D I	S	S	S	S	S	F	L S	T S	S	S	A	S	S	F S	S	Q A	S - - - A	S	S	T S	G	A

Aar_R_19	G A TA C CG G CA G TG TG CA G A G TG A CA T TA G T T C CA G TA G CA G C T T C C T C T CA A C CA G C T CG T C TG CG T C CA G T T T C T C C T C T CA G G CA T CA - - - G C T T C T T C TA C CA G CG G TG C TG

Frame 1

D	T G	S	V	Q S	D I	S	S	S	S	S	F	L S	T S	S	S	A	S	S	F S	S	Q A	S - - - A	S	S	T S	G	A

Aar_R_20	G A TA C CG G CA G TG TA CA G A G TG A CA T TA G T T C CA G TA G CA G C T T C C T C T CA A C CA G C T CG T C TG CG T C CA G TA T C T C C T C T CA G G TA T CA - - - G C T T C T T C TA C CA G CG G TG C TG

Frame 1

At_R_1

Frame 1

At_R_2

Frame 1

At_R_3

Frame 1

At_R_4

Frame 1

At_R_5

Frame 1

At_R_6

Frame 1

At_R_7

Frame 1

At_R_8

Frame 1

At_R_9

Frame 1

At_R_10

Frame 1

At_R_11

Frame 1

At_R_12

Frame 1

At_R_13

Frame 1

At_R_14

Frame 1

Av_R_1

Frame 1

Av_R_2

Frame 1

Av_R_3

Frame 1

Av_R_4

Frame 1

Av_R_5

Frame 1

Av_R_6

Frame 1

Av_R_7

Frame 1

Av_R_8

Frame 1

Av_R_9

Frame 1

Av_R_10

Frame 1

D	T G	S	V	Q S	D I	S	S	S	S	S	F	L S	T S	S	S	A	S	S	I	S	S	Q V	S - - - A	S	S	T S	G	A

G A TA G CG G CA G TG TA CA A A G TG A CA T TA G T T C CA G TA G CA G C T T C C T C T CA A CA A G C T CG T C T T CG G C CA G T TA C- - - T C T CA G G CA T CA - - - G C T T C T T CG A C CA G CG G TG C CG D S	G	S	V	Q S	D I	S	S	S	S	S	F	L S	T S	S	S	S	A	S	Y - - - S	Q A	S - - - A	S	S	T S	G	A

G A TA G CG G CA G TG TA CA A A G TG A CA T TA G T T C CA G TA G CA G C T T C C T C T CA A CA A G C T CG T C T T CG G C CA G T TA C- - - T C T CA G G CA T CA - - - G C T T C T T CG A C CA G CG G TG C CG D S	G	S	V	Q S	D I	S	S	S	S	S	F	L S	T S	S	S	S	A	S	Y - - - S	Q A	S - - - A	S	S	T S	G	A

G A TA G CG G CA G TG TA CA A A G TG A CA T TA G T T C CA G TA G TA G C T T C C T C T CA A CA A G C T CG T C T T CG G C CA G T TA C- - - T C T CA G G CA T CA - - - G C T T C T T CG A C CA G CG G TG C CG D S	G	S	V	Q S	D I	S	S	S	S	S	F	L S	T S	S	S	S	A	S	Y - - - S	Q A	S - - - A	S	S	T S	G	A

G A TA G CG G CA G TG TA CA A A G TG A CA T TA G T T C CA G TA G CA G C T T C C T C T CA A CA A G C T CG T C T T CG G C CA G T TA C- - - T C T CA G G CA T CA - - - G C T T C T T CG A C CA G CG G TG C CG D S	G	S	V	Q S	D I	S	S	S	S	S	F	L S	T S	S	S	S	A	S	Y - - - S	Q A	S - - - A	S	S	T S	G	A

G A TA G CG G CA G TG TA CA A A G TG A CA T TA G T T C CA G TA G CA G C T T C C T C T CA A CA A G C T CG T C T T CG G C CA G T TA C- - - T C T CA G G CA T CA - - - G C T T C T T CG A C CA G CG G TG C CG D S	G	S	V	Q S	D I	S	S	S	S	S	F	L S	T S	S	S	S	A	S	Y - - - S	Q A	S - - - A	S	S	T S	G	A

G A TA G CG G CA G TG TA CA A A G TG A CA T TA G T T C CA G TA G CA G C T T C C T C T CA A CA A G C T CG T C T T CG G C CA G T TA C- - - T C T CA G G CA T CA - - - G C T T C T T CG A C CA G CG G TA C CG D S	G	S	V	Q S	D I	S	S	S	S	S	F	L S	T S	S	S	S	A	S	Y - - - S	Q A	S - - - A	S	S	T S	G	T

G A TA G CG G CA G TG TA CA A A G TG A CA T TA G T T C CA G TA G CA G C T T C C T C T CA A CA A G C T CG T C T T CG G C CA G T TA C- - - T C T CA G G CA T CA - - - G C T T C T T CG A C CA G CG G TG C CG D S	G	S	V	Q S	D I	S	S	S	S	S	F	L S	T S	S	S	S	A	S	Y - - - S	Q A	S - - - A	S	S	T S	G	A

G A TA G CG G CA G TG TA CA A A G TG A CA T TA G T T C CA G TA G CA G C T T C C T C T CA A CA A G C T CG T C T T CG G C CA G T TA C- - - T C T CA G G CA T CA - - - G C T T C T T CG A C CA G CG G TG C CG D S	G	S	V	Q S	D I	S	S	S	S	S	F	L S	T S	S	S	S	A	S	Y - - - S	Q A	S - - - A	S	S	T S	G	A

G A TA G CG G CA G TG TA CA A A G TG A CA T TA G T T C CA G TA G CA G C T T C C T C T CA A CA A G C T CG T C T T CG G C CA G T TA C- - - T C T CA G G CA T CA - - - G C T T C T T CG A C CA G CG G TG C CG D S	G	S	V	Q S	D I	S	S	S	S	S	F	L S	T S	S	S	S	A	S	Y - - - S	Q A	S - - - A	S	S	T S	G	A

G A TA G CG G CA G TG TA CA A A G TG A CA T TA G T T C CA G TA G CA G C T T C C T C T CA A CA A G C T CG T C T T CG G C CA G T TA C- - - T C T CA G G CA T CA - - - G C T T C T T CG A C CA G CG G TG C CG D S	G	S	V	Q S	D I	S	S	S	S	S	F	L S	T S	S	S	S	A	S	Y - - - S	Q A	S - - - A	S	S	T S	G	A

G A TA G CG G CA G TG TA CA A A G TG A CA T TA G T T C CA G TA G CA G C T T C C T C T CA A CA A G C T CG T C T T CG G C CA G T TA C- - - T C T CA G G CA T CA - - - G C T T C T T CG A C CA G CG G TG C CG D S	G	S	V	Q S	D I	S	S	S	S	S	F	L S	T S	S	S	S	A	S	Y - - - S	Q A	S - - - A	S	S	T S	G	A

G A TA G CG G CA G TG TA CA A A G TG A CA T TA G T T C CA G TA G CA G C T T C C T C T CA A CA A G C T CG T C T T CG G C CA G T TA C- - - T C T CA G G CA T CA - - - G C T T C T T CG A C CA G CG G TG C CG D S	G	S	V	Q S	D I	S	S	S	S	S	F	L S	T S	S	S	S	A	S	Y - - - S	Q A	S - - - A	S	S	T S	G	A

G A TA G CG G CA G TG TA CA A A G TG A CA T TA G T T C CA G TA G CA G C T T C C T C T CA A CA A G C T CG T C T T CG G C CA G T TA C- - - T C T CA G G CA T TA - - - G C T T C T T CG A C CA G CG G TG C CG D S	G	S	V	Q S	D I	S	S	S	S	S	F	L S	T S	S	S	S	A	S	Y - - - S	Q A	L - - - A	S	S	T S	G	A

G A TA G CG G CA G TG TA CA A A G TG A CA T TA G T T C CA G TA G CA G C T T C C T C T CA A CA A G C T CG T C T T CG G C CA G T TA C- - - T C T CA G G CA T CA - - - G C T T C T T CG A C CA G CG G TG C CG D S	G	S	V	Q S	D I	S	S	S	S	S	F	L S	T S	S	S	S	A	S	Y - - - S	Q A	S - - - A	S	S	T S	G	A

N N N N N N N N N N N N N N N N N N N N N N N N N N N N N N N N N N N N N N N N N N N N N N N N N N N N N N N N N N N N N N N N N N N N N N N N N N N N N N N N N N N N N N N N N N N N N N N N N N N N N N N N N N N N N N N N N N N

? ? ? ? ? ? ? ? ? ? ? ? ? ? ? ? ? ? ? ? ? ? ? ? ? ? ? ? ? ? ? ? ? ? ? ? ? ?

G A CA C TG G A A G TG TA CA A A G TG A CA T T CG T T C CA G CA G TA G C T C C C TG T CA A CA A G T T C T T CG T C TG C CA G T T T C- - - T C T CA G A C CA G TG G TG CA G C T T CG A CA A C TG G T- - - - D	T G	S	V	Q S	D I	R	S	S	S	S	S	L S	T S	S	S	S	A	S	F - - - S	Q	T S	G	A	A	S	T	T G - - - -

G A CA C TG G A A G TG TA CA A A G TG A CA T T CG T T C CA G CA G TA G C T C C C TG T CA A CA A G T T C T T CG T C TG C CA G T T T C- - - T C T CA G A C CA G TG G TG CA G C T T CG A CA A C TG G T- - - - D	T G	S	V	Q S	D I	R	S	S	S	S	S	L S	T S	S	S	S	A	S	F - - - S	Q	T S	G	A	A	S	T	T G - - - -

G A CA C TG G A A G TG TA CA A A G TG A CA T T CG T T C CA G CA G TA G C T C C C TG T CA A CA A G T T C T T CG T C TG C CA G T T T C- - - T C T CA G A C CA G TG G TG CA G C T T CG A CA A C TG G T- - - - D	T G	S	V	Q S	D I	R	S	S	S	S	S	L S	T S	S	S	S	A	S	F - - - S	Q	T S	G	A	A	S	T	T G - - - -

G A CA C TG G A A G TG TA CA A A G TG A CA T T CG T T C CA G CA G TA G C T C C C TG T CA A CA A G T T C T T CG T C TG C CA G T T T C- - - T C T CA G A C CA G TG G TG CA G C T T CG A CA A C TG G T- - - - D	T G	S	V	Q S	D I	R	S	S	S	S	S	L S	T S	S	S	S	A	S	F - - - S	Q	T S	G	A	A	S	T	T G - - - -

G A CA C TG G A A G TG TA CA A A G TG A CA T T CG T T C CA G CA G TA G C T C C C TG T CA A CA A G T T C T T CG T C TG C CA G T T T C- - - T C T CA G A C CA G TG G TG CA G C T T CG A CA A C TG G T- - - - D	T G	S	V	Q S	D I	R	S	S	S	S	S	L S	T S	S	S	S	A	S	F - - - S	Q	T S	G	A	A	S	T	T G - - - -

G A CA C TG G A A G TG TA CA A A G TG A CA T T CG T T C CA G CA G TA G C T C C C TG T CA A CA A G T T C T T CG T C TG C CA G T T T C- - - T C T CA G A C CA G TG G TG CA G C T T CG A CA A C TG G T- - - - D	T G	S	V	Q S	D I	R	S	S	S	S	S	L S	T S	S	S	S	A	S	F - - - S	Q	T S	G	A	A	S	T	T G - - - -

G A CA C TG G A A G TG TA CA A A G TG A CA T T CG T T C CA G CA G TA G C T C C C TG T CA A CA A G T T C T T CG T C TG C CA G T T T C- - - T C T CA G A C CA G TG G TG CA G C T T CG A CA A C TG G T- - - - D	T G	S	V	Q S	D I	R	S	S	S	S	S	L S	T S	S	S	S	A	S	F - - - S	Q	T S	G	A	A	S	T	T G - - - -

G A CA C TG G A A G TG TA CA A A G TG A CA T T CG T T C CA G CA G TA G C T C C C TG T CA A CA A G T T C T T CG T C TG C CA G T T T C- - - T C T CA G A C CA G TG G TG CA G C T T CG A CA A C TG G T- - - - D	T G	S	V	Q S	D I	R	S	S	S	S	S	L S	T S	S	S	S	A	S	F - - - S	Q	T S	G	A	A	S	T	T G - - - -

G A CA C TG G A A G TG TA CA A A G TG A CA T T CG T T C CA G CA G TA G C T C C C TG T CA A CA A G T T C T T CG T C TG C CA G T T T C- - - T C T CA G A C CA G TG G TG CA G C T T CG A CA A C TG G T- - - - D	T G	S	V	Q S	D I	R	S	S	S	S	S	L S	T S	S	S	S	A	S	F - - - S	Q	T S	G	A	A	S	T	T G - - - -

Aam_R_1	N N N N N N N N N N N N N N N N N N N N N G A CA T TA G T T C CA G CA G TA G C T T C C T C T CA A G TA G C T CG T C T T CG G C TA G T T T C- - - T C T CA G A CA T CA - - - G C T T C T T C TA C CA G CG G TG C TG

Frame 1

?	?	?	?	?	?	?	D I	S	S	S	S	S	F	L S	S	S	S	S	S	A	S	F - - - S	Q	T S - - - A	S	S	T S	G	A

Aam_R_2	G A TA C CG G CA G TG TA CA A A G CG A CA T TA G T T C CA G CA G TA G C T T C C T C T CA A G TA G C T CG T C T T CG G C TA G T T T C- - - T C T CA G A CA T CA - - - G C T T C T T C TA C CA G CG G TG C TG

Frame 1

D	T G	S	V	Q S	D I	S	S	S	S	S	F	L S	S	S	S	S	S	A	S	F - - - S	Q	T S - - - A	S	S	T S	G	A

Aam_R_3	G A TA C CG G CA G TG TA CA A A G CG A CA T TA G T T C CA G CA G TA G C T T C C T C T CA A G TA G C T CG T C C T CG G C TA G T T T C- - - T C T CA G A CA T CA - - - G C T T C T T C TA C CA G CG G TG C TG

Frame 1

D	T G	S	V	Q S	D I	S	S	S	S	S	F	L S	S	S	S	S	S	A	S	F - - - S	Q	T S - - - A	S	S	T S	G	A

Aam_R_4	G A TA C CG G CA G TG TA CA A A G TG A CA T TA G T T C CA G CA G TA G C T T C C T C T CA A G CA G C T CG T C T T CG T C TA G TA T C- - - T C C CA G A CA T CA - - - G C T T C T T C TA C CA G CG G TG C TG

Frame 1

D	T G	S	V	Q S	D I	S	S	S	S	S	F	L S	S	S	S	S	S	S	S	I - - - S	Q	T S - - - A	S	S	T S	G	A

Aam_R_5	G A TA G CG G CA G TA TA CA A A G TG A CA T TA G T T C CA G CA G TA G C T T C C T C T CA A G CA G C T CG T C T T CG T C TA G TA T C- - - T C C CA G A CA T CA - - - G C T T C T T C TA C CA G CG G TG C TG

Frame 1

D S	G	S	I	Q S	D I	S	S	S	S	S	F	L S	S	S	S	S	S	S	S	I - - - S	Q	T S - - - A	S	S	T S	G	A

Aam_R_6	G A TA G CG G CA G TN N N N N N N N N N N N N N N N N N N N N N N N N N N N N N N N N N N N N N N N N N N N N N N N N N N N N N N N N N N N N N N N N N N N N N N N N N N N N N N N N N N N N N N N N N N N N N N N N N N N N N N

Frame 1

D S	G	S	?	?	?	?	?	?	?	?	?	?	?	?	?	?	?	?	?	?	?	?	?	?	?	?	?	?	?	?	?	?	?	?	?	?

Aar_R_1

Frame 1

Aar_R_2

Frame 1

Aar_R_3

Frame 1

Aar_R_4

Frame 1

Aar_R_5

Frame 1

Aar_R_6

Frame 1

Aar_R_7

Frame 1

Aar_R_8

Frame 1

Aar_R_9

Frame 1

470	480	490	500	510	520	530	540	550	560	570

G A TA CA C CG G A - - - - - - - - - - - - - - - - - - - - - - - - - - - - - - - - - - - - C C T T C TG G C TA CA CA G G A C CA G TG G G TG G CG G A G CG CA G T T CG G T T C TG C T T CA G G C CA A A G C T C T T T G	Y	T G - - - - - - - - - - - - - - - - - - - - - - - - - - - - - - - - - - - - P S	G	Y	T G	P V	G	G	G	A	Q	F G	S	A	S	G	Q S	S	F

G C TA CA C CG G A - - - - - - - - - - - - - - - - - - - - - - - - - - - - - - - - - - - - C C T T C TG G C TA CA CA G G A C CA G TG G G TG G CG G A G CG CA G T T CG G T T C TG C T T CA G G C CA A A G C T C T T T G	Y	T G - - - - - - - - - - - - - - - - - - - - - - - - - - - - - - - - - - - - P S	G	Y	T G	P V	G	G	G	A	Q	F G	S	A	S	G	Q S	S	F

G C TA CA C CG G A - - - - - - - - - - - - - - - - - - - - - - - - - - - - - - - - - - - - C C T T C TG G C TA CA CA G G A C CA G TG G G TG G CG G A G CG CA G T T CG G T T C TG C T T CA G G C CA A A G C T C T T T G	Y	T G - - - - - - - - - - - - - - - - - - - - - - - - - - - - - - - - - - - - P S	G	Y	T G	P V	G	G	G	A	Q	F G	S	A	S	G	Q S	S	F

G C TA CA C CG G A - - - - - - - - - - - - - - - - - - - - - - - - - - - - - - - - - - - - C C T T C TG G C TA CA CA G G A C CA G TG G G TG G CG G A G CG CA G T T CG G T T C TG C T T CA G G C CA A A G C T C T T T G	Y	T G - - - - - - - - - - - - - - - - - - - - - - - - - - - - - - - - - - - - P S	G	Y	T G	P V	G	G	G	A	Q	F G	S	A	S	G	Q S	S	F

G C TA CA C CG G A - - - - - - - - - - - - - - - - - - - - - - - - - - - - - - - - - - - - C C T T C TG G C TA CA CA G G A C CA G TG G G TG G CG G A G CG CA G T T CG G T T C TG C T T CA G G C CA A A G C T C T T T G	Y	T G - - - - - - - - - - - - - - - - - - - - - - - - - - - - - - - - - - - - P S	G	Y	T G	P V	G	G	G	A	Q	F G	S	A	S	G	Q S	S	F

G C TA CA C CG G A - - - - - - - - - - - - - - - - - - - - - - - - - - - - - - - - - - - - C C T T C TG G C TA CA CA G G A C CA G TG G G TG G CG G A G CG CA G T T CG G T T C TG C T T CA G G C CA A A G C T C T T T G	Y	T G - - - - - - - - - - - - - - - - - - - - - - - - - - - - - - - - - - - - P S	G	Y	T G	P V	G	G	G	A	Q	F G	S	A	S	G	Q S	S	F

G C TA CA C CG G A - - - - - - - - - - - - - - - - - - - - - - - - - - - - - - - - - - - - C C T T C TG G C TA CA CA G G A C CA G TG G G TG G CG G A G CG CA G T T CG G T T CA G C T T CA G G C CA A A G C T C T T T G	Y	T G - - - - - - - - - - - - - - - - - - - - - - - - - - - - - - - - - - - - P S	G	Y	T G	P V	G	G	G	A	Q	F G	S	A	S	G	Q S	S	F

G C TA CA C CG G A - - - - - - - - - - - - - - - - - - - - - - - - - - - - - - - - - - - - C C T T C TG G C TA CA CA G G A C CA G TG G G TG G CG G A G CG CA G T T CG G T T C TG C T T CA G G C CA A A G C T C T T T G	Y	T G - - - - - - - - - - - - - - - - - - - - - - - - - - - - - - - - - - - - P S	G	Y	T G	P V	G	G	G	A	Q	F G	S	A	S	G	Q S	S	F

G C TA CA C CG G A - - - - - - - - - - - - - - - - - - - - - - - - - - - - - - - - - - - - C C T T C TG G A TA CA CA G G A C CA G TG G G TG G CG G A G CG CA G T T CG G T T C TG C T T CA G G C CA A A G C T C T T T G	Y	T G - - - - - - - - - - - - - - - - - - - - - - - - - - - - - - - - - - - - P S	G	Y	T G	P V	G	G	G	A	Q	F G	S	A	S	G	Q S	S	F

Aar_R_10	G C TA CA C CG G A - - - - - - - - - - - - - - - - - - - - - - - - - - - - - - - - - - - - C C T T C TG G C TA CA CA G G A C CA G TG G G TG G TG G A G CG CA G T T CG G T T C TG C T T CA G G C CA A A G T T C T T T

Frame 1

G	Y	T G - - - - - - - - - - - - - - - - - - - - - - - - - - - - - - - - - - - - P S	G	Y	T G	P V	G	G	G	A	Q	F G	S	A	S	G	Q S	S	F

Aar_R_11	G C TA CA C CG G A - - - - - - - - - - - - - - - - - - - - - - - - - - - - - - - - - - - - T C T T C TG G T TA CA CA G G A C CA A A G G G TG G CG G A G CG CA G T T CG G T T C TG C T T CA G G C CA A A G C T C T T T

Frame 1

G	Y	T G - - - - - - - - - - - - - - - - - - - - - - - - - - - - - - - - - - - - S	S	G	Y	T G	P K	G	G	G	A	Q	F G	S	A	S	G	Q S	S	F

Aar_R_12	G C TA CA C CG G A - - - - - - - - - - - - - - - - - - - - - - - - - - - - - - - - - - - - C C T T C TG G C TA CA TA G G A C CA G TG G G TG G CG G A G CG CA G T T CG G T T C TG C T T CA G G C CA A A G C T C T T T

Frame 1

G	Y	T G - - - - - - - - - - - - - - - - - - - - - - - - - - - - - - - - - - - - P S	G	Y I	G	P V	G	G	G	A	Q	F G	S	A	S	G	Q S	S	F

Aar_R_13	G C TA CA C CG G A - - - - - - - - - - - - - - - - - - - - - - - - - - - - - - - - - - - - C C T T C TG G C TA CA CA G G A C CA G TG G G TG G CG G A G CG CA G T T CG G T T C TG C T T CA G G C CA A A G C T C T T T

Frame 1

G	Y	T G - - - - - - - - - - - - - - - - - - - - - - - - - - - - - - - - - - - - P S	G	Y	T G	P V	G	G	G	A	Q	F G	S	A	S	G	Q S	S	F

Aar_R_14	G C TA CA C CG G A - - - - - - - - - - - - - - - - - - - - - - - - - - - - - - - - - - - - C C T T C TG G C TA CA CA G G A C CA G TG G G TG G CG G A G CG CA G T T CG G T T C TG C T T CA G G C CA A A G C T C T T T

Frame 1

G	Y	T G - - - - - - - - - - - - - - - - - - - - - - - - - - - - - - - - - - - - P S	G	Y	T G	P V	G	G	G	A	Q	F G	S	A	S	G	Q S	S	F

Aar_R_15	G C TA CA C CG A A - - - - - - - - - - - - - - - - - - - - - - - - - - - - - - - - - - - - C C T T C TG G C TA CA CA G G A C CA G TG G G TG G CG G A G CG CA G T T CG G T T C TG C T T CA G G C CA A A G C T C T T T

Frame 1

G	Y	T	E - - - - - - - - - - - - - - - - - - - - - - - - - - - - - - - - - - - - P S	G	Y	T G	P V	G	G	G	A	Q	F G	S	A	S	G	Q S	S	F

Aar_R_16	G C TA CA C CG G A - - - - - - - - - - - - - - - - - - - - - - - - - - - - - - - - - - - - C C T T C TG G C TA CA CA G G A C CA A A G G G TG G CG G A G CG CA G T T CG G T T C TG C T T CA G G C CA A A G C T C T T T

Frame 1

G	Y	T G - - - - - - - - - - - - - - - - - - - - - - - - - - - - - - - - - - - - P S	G	Y	T G	P K	G	G	G	A	Q	F G	S	A	S	G	Q S	S	F

Aar_R_17	G C TA CA C CG G A - - - - - - - - - - - - - - - - - - - - - - - - - - - - - - - - - - - - C C T T C TG G C TA CA CA G G A C CA G TG G G TG G CG G A G CG CA G T T CG G T T C TG C T T CA G G C CA A A G C T C T T T

Frame 1

G	Y	T G - - - - - - - - - - - - - - - - - - - - - - - - - - - - - - - - - - - - P S	G	Y	T G	P V	G	G	G	A	Q	F G	S	A	S	G	Q S	S	F

Aar_R_18	G C TA CA C CG A A - - - - - - - - - - - - - - - - - - - - - - - - - - - - - - - - - - - - C C T T C TG G C TA CA CA G G A C CA G TG G G TG G CG G A G CG CA G T T CG G T T C TG C T T CA G G C CA A A G C T C T T T

Frame 1

G	Y	T	E - - - - - - - - - - - - - - - - - - - - - - - - - - - - - - - - - - - - P S	G	Y	T G	P V	G	G	G	A	Q	F G	S	A	S	G	Q S	S	F

Aar_R_19	G C TA CA C CG G A - - - - - - - - - - - - - - - - - - - - - - - - - - - - - - - - - - - - C C T T C TG G C TA CA CA G G A C CA G TG G G TG G CG G A G CG CA G T T CG G T T C TG C T T CA G G C CA A A G C T C T T T

Frame 1

G	Y	T G - - - - - - - - - - - - - - - - - - - - - - - - - - - - - - - - - - - - P S	G	Y	T G	P V	G	G	G	A	Q	F G	S	A	S	G	Q S	S	F

Aar_R_20	G C TA CA C CG G A - - - - - - - - - - - - - - - - - - - - - - - - - - - - - - - - - - - - C C T T C TG G C TA CA CA G G A C CA G TG G G TG G CG G A G CG CA G T T CG G T T C T T C T T CA G G C CA A A G C T C T T T

Frame 1

At_R_1

Frame 1

At_R_2

Frame 1

At_R_3

Frame 1

At_R_4

Frame 1

At_R_5

Frame 1

At_R_6

Frame 1

At_R_7

Frame 1

At_R_8

Frame 1

At_R_9

Frame 1

At_R_10

Frame 1

At_R_11

Frame 1

At_R_12

Frame 1

At_R_13

Frame 1

At_R_14

Frame 1

Av_R_1

Frame 1

Av_R_2

Frame 1

Av_R_3

Frame 1

Av_R_4

Frame 1

Av_R_5

Frame 1

Av_R_6

Frame 1

Av_R_7

Frame 1

Av_R_8

Frame 1

Av_R_9

Frame 1

Av_R_10

Frame 1

G	Y	T G - - - - - - - - - - - - - - - - - - - - - - - - - - - - - - - - - - - - P S	G	Y	T G	P V	G	G	G	A	Q	F G	S	S	S	G	Q S	S	F

G A TA CA CA G G A C C T T C TG G A C C T T C C- - - - - - A C TG G A - - - - - - - - - C C T T C TG G C TA C C C TG G G C C T T TG G G TG G CG G A G CG C CG - - - - - - - - - - - - - - - - - - - - - - - - - - - T T G	Y	T G	P S	G	P S - - - - - - T G - - - - - - - - - P S	G	Y	P G	P	L G	G	G	A	P - - - - - - - - - - - - - - - - - - - - - - - - - - - F

G A TA CA CA G G A C C T T C TG G A C C T T C C- - - - - - A C TG G A - - - - - - - - - C C T T C TG G C TA C C C TG G G C T T T TG G G TG G CG G A G CG C C T- - - - - - - - - - - - - - - - - - - - - - - - - - - T T G	Y	T G	P S	G	P S - - - - - - T G - - - - - - - - - P S	G	Y	P G	L	L G	G	G	A	P - - - - - - - - - - - - - - - - - - - - - - - - - - - F

G A TA CA CA G G A C C T T C TG G A C C T T C C- - - - - - A C TG G A - - - - - - - - - C C T T C TG G C TA C C C TG G G C C T T TG G G TG G CG G A G CG C CG - - - - - - - - - - - - - - - - - - - - - - - - - - - T T G	Y	T G	P S	G	P S - - - - - - T G - - - - - - - - - P S	G	Y	P G	P	L G	G	G	A	P - - - - - - - - - - - - - - - - - - - - - - - - - - - F

G A TA CA CA G G A C C T T C TG G A C C T T C C- - - - - - A C TG G A - - - - - - - - - C C T T C TG G C TA C C C TG G G C C T T TG G G TG G CG G A G CG C CG - - - - - - - - - - - - - - - - - - - - - - - - - - - T T G	Y	T G	P S	G	P S - - - - - - T G - - - - - - - - - P S	G	Y	P G	P	L G	G	G	A	P - - - - - - - - - - - - - - - - - - - - - - - - - - - F

G A TA CA CA G G A C C T T C TG G A C C T T C C- - - - - - A C TG G A - - - - - - - - - C C T T C TG G C TA C C C TG G G C C T T TG G G TG G CG G A G CG C CG - - - - - - - - - - - - - - - - - - - - - - - - - - - T T G	Y	T G	P S	G	P S - - - - - - T G - - - - - - - - - P S	G	Y	P G	P	L G	G	G	A	P - - - - - - - - - - - - - - - - - - - - - - - - - - - F

G A TA CA CA G G A C C T T C TG G A C C T T C C- - - - - - A C TG G A - - - - - - - - - C C T T C TG G C TA C C C TG G G C C T T TG G G TG G CG G A G CG C CG - - - - - - - - - - - - - - - - - - - - - - - - - - - T T G	Y	T G	P S	G	P S - - - - - - T G - - - - - - - - - P S	G	Y	P G	P	L G	G	G	A	P - - - - - - - - - - - - - - - - - - - - - - - - - - - F

G A TA CA CA G G A C C T T C TG G A C C T T C C- - - - - - A C TG G A - - - - - - - - - C C T T C TG G C TA C C C TG G G C C T T TG G G TG G CG G A G CG C CG - - - - - - - - - - - - - - - - - - - - - - - - - - - T T G	Y	T G	P S	G	P S - - - - - - T G - - - - - - - - - P S	G	Y	P G	P	L G	G	G	A	P - - - - - - - - - - - - - - - - - - - - - - - - - - - F

G A TA CA CA G G A C C T T C TG G A C C T T C C- - - - - - A C TG G A - - - - - - - - - C C T T C TG G C TA C C C TG G G C C T T TG G G TG G CG G A G CG C CG - - - - - - - - - - - - - - - - - - - - - - - - - - - T T G	Y	T G	P S	G	P S - - - - - - T G - - - - - - - - - P S	G	Y	P G	P	L G	G	G	A	P - - - - - - - - - - - - - - - - - - - - - - - - - - - F

G A TA CA CA G G A C C T T C TG G A C C T T C C- - - - - - A C TG G A - - - - - - - - - C C T T C TG G C TA C C C TG G G C C T T TG G G TG G CG G A G CG C CG - - - - - - - - - - - - - - - - - - - - - - - - - - - T T G	Y	T G	P S	G	P S - - - - - - T G - - - - - - - - - P S	G	Y	P G	P	L G	G	G	A	P - - - - - - - - - - - - - - - - - - - - - - - - - - - F

G A TA CA CA G G A C C T T C TG G A C C T T C C- - - - - - A C TG G A - - - - - - - - - C C T T C TG G C TA C C C TG G G C C T T TG G G TG G CG G A G CG C CG - - - - - - - - - - - - - - - - - - - - - - - - - - - T T G	Y	T G	P S	G	P S - - - - - - T G - - - - - - - - - P S	G	Y	P G	P	L G	G	G	A	P - - - - - - - - - - - - - - - - - - - - - - - - - - - F

G A TA CA CA G G A C C T T C TG G A C C T T C C- - - - - - A C TG G A - - - - - - - - - C C T T C TG G C TA C C C TG G G C C T T TG G G TG G CG G A G CG C CG - - - - - - - - - - - - - - - - - - - - - - - - - - - T T G	Y	T G	P S	G	P S - - - - - - T G - - - - - - - - - P S	G	Y	P G	P	L G	G	G	A	P - - - - - - - - - - - - - - - - - - - - - - - - - - - F

G A TA CA CA G G A C C T T C TG G A C C T T C C- - - - - - A C TG G A - - - - - - - - - C C T T C TG G C TA C C C TG G G C C T T TG G G TG G CG G A G CG C CG - - - - - - - - - - - - - - - - - - - - - - - - - - - T T G	Y	T G	P S	G	P S - - - - - - T G - - - - - - - - - P S	G	Y	P G	P	L G	G	G	A	P - - - - - - - - - - - - - - - - - - - - - - - - - - - F

G A TA CA CA G G A C C T T C TG G A C C T T C C- - - - - - A C TG G A - - - - - - - - - C C T T C TG G C TA C C C TG G G C C T T TG G G TG G CG G A G CG C CG - - - - - - - - - - - - - - - - - - - - - - - - - - - T T G	Y	T G	P S	G	P S - - - - - - T G - - - - - - - - - P S	G	Y	P G	P	L G	G	G	A	P - - - - - - - - - - - - - - - - - - - - - - - - - - - F

G A TA CA CA G G A C C T T C TG G A C C T T C C- - - - - - A C TG G A - - - - - - - - - C C T T C TG G C TA C C C TG G G C C T T TG A G TG G CG G A G CG T CG - - - - - - - - - - - - - - - - - - - - - - - - - - - T T G	Y	T G	P S	G	P S - - - - - - T G - - - - - - - - - P S	G	Y	P G	P	L S	G	G	A	S - - - - - - - - - - - - - - - - - - - - - - - - - - - F

N N N N N N N N N N N N N N N N N N N N N N N N N N N N N N N N N N N N N N N N N N N N N N N N N N N N N N N N N N N N N N N N N N N N N N N N N N N N N N N N N N N N N N N N N N N N N N N N N N N N N N N N N N N N N N T C T T T

?	?	?	?	?	?	?	?	?	?	?	?	?	?	?	?	?	?	?	?	?	?	?	?	?	?	?	?	?	?	?	?	?	?	?	?	? S	F

- - T T CA CA G G G - - - G C TG G T- - - - - - G G C TA C- - - - - - - - - - - - - - - C C TG G TG G A - - - G C TG G T C C T T TG G G TG G CG G A G TA - - - - - - G G C T C T T TG A CA G G C CA A A C C T C T T T

- - F	T G - - - A	G - - - - - - G	Y - - - - - - - - - - - - - - - P G	G - - - A	G	P	L G	G	G	V - - - - - - G	S	L	T G	Q	T S	F

- - T T CA CA G G G - - - G C TG G T- - - - - - G G C TA C- - - - - - - - - - - - - - - C C TG G TG G A - - - G C TG G T C C T T TG G G TG G CG G A G TA - - - - - - G G C T C T T TG A CA G G C CA A A C C T C T T T

- - F	T G - - - A	G - - - - - - G	Y - - - - - - - - - - - - - - - P G	G - - - A	G	P	L G	G	G	V - - - - - - G	S	L	T G	Q	T S	F

- - T T CA CA G G G - - - G C TG G T- - - - - - G G C TA C- - - - - - - - - - - - - - - C C TG G TG G A - - - G C TG G T C C T T TG G G TG G CG G A G TA - - - - - - G G C T C T T TG A CA G G C CA A A C C T C T T T

- - F	T G - - - A	G - - - - - - G	Y - - - - - - - - - - - - - - - P G	G - - - A	G	P	L G	G	G	V - - - - - - G	S	L	T G	Q	T S	F

- - T T CA CA G G G - - - G C TG G T- - - - - - G G C TA C- - - - - - - - - - - - - - - C C TG G TG G A - - - G C TG G T C C T T TG G G TG G CG G A G TA - - - - - - G G C T C T T TG A CA G G C CA A A C C T C T T T

- - F	T G - - - A	G - - - - - - G	Y - - - - - - - - - - - - - - - P G	G - - - A	G	P	L G	G	G	V - - - - - - G	S	L	T G	Q	T S	F

- - T T CA CA G G G - - - G C TG G T- - - - - - G G C TA C- - - - - - - - - - - - - - - C C TG G TG G A - - - G C TG G T C C T T TG G G TG G CG G A G TA - - - - - - G G C T C T T TG A CA G G C CA A A C C T C T T T

- - F	T G - - - A	G - - - - - - G	Y - - - - - - - - - - - - - - - P G	G - - - A	G	P	L G	G	G	V - - - - - - G	S	L	T G	Q	T S	F

- - T T CA CA G G G - - - G CG G G T- - - - - - G G C TA C- - - - - - - - - - - - - - - C C TG G TG G A - - - G C TG G T C C T T TG G G TG G CG G A G TA - - - - - - G G C T C T T TG A CA G G C CA TA C C T C T T T

- - F	T G - - - A	G - - - - - - G	Y - - - - - - - - - - - - - - - P G	G - - - A	G	P	L G	G	G	V - - - - - - G	S	L	T G	H	T S	F

- - T T CA CA G G G - - - G C TG G T- - - - - - G G C TA C- - - - - - - - - - - - - - - C C TG G TG G A - - - G C TG G T C C T T TG G G TG G CG G A G TA - - - - - - G G C T C T T TG A CA G G C CA A A C C T C T T T

- - F	T G - - - A	G - - - - - - G	Y - - - - - - - - - - - - - - - P G	G - - - A	G	P	L G	G	G	V - - - - - - G	S	L	T G	Q	T S	F

- - T T CA CA G G G - - - G C TG G T- - - - - - G G C TA C- - - - - - - - - - - - - - - C C TG G TG G A - - - G C TG G T C C T T TG G G TG G CG G A G TA - - - - - - G G C T C T T TG A CA G G C CA A A C C T C T T T

- - F	T G - - - A	G - - - - - - G	Y - - - - - - - - - - - - - - - P G	G - - - A	G	P	L G	G	G	V - - - - - - G	S	L	T G	Q	T S	F

- - T T CA CA G G G - - - G C TG G T- - - - - - G G C TA C- - - - - - - - - - - - - - - C C TG G TG G A - - - G C TG G T C C T T TG G G TG G CG G A G TA - - - - - - G G C T CA T T TG G A G G T CA A A C C T C T T T

- - F	T G - - - A	G - - - - - - G	Y - - - - - - - - - - - - - - - P G	G - - - A	G	P	L G	G	G	V - - - - - - G	S	F G	G	Q	T S	F

Aam_R_1	G C TA CA CA G CG C C TG C TG G A C C T T C TG G C TA CA CA G G G C C T T C TG G A C C T T C TG G C TA CA C TG G G C C TG TG G G TG G CG CA G CG CA G T T CG G T T C TG T C T CA G G C CA A A C C T C T T T

Frame 1

G	Y	T A	P A	G	P S	G	Y	T G	P S	G	P S	G	Y	T G	P V	G	G	A	A	Q	F G	S	V	S	G	Q	T S	F

Aam_R_2	G C TA CA CA G CG C C TG C TG G A C C T T C TG G C TA CA CA G G G C C T T C TG G A C C T T C TG G C TA CA C TG G G C C TG TG G G TG G CG CA G CG CA G T T CG G T T C TG T C T CA G G C CA A A C C T C T T T

Frame 1

G	Y	T A	P A	G	P S	G	Y	T G	P S	G	P S	G	Y	T G	P V	G	G	A	A	Q	F G	S	V	S	G	Q	T S	F

Aam_R_3	G C TA CA CA G G G C C T T C TG G A C C T T C TG G C TA CA C TG G G C C TG C TG G A C C T T C TG G C TA CA C TG G G C C TG TG G G TG G CG CA G CG CA G T T CG G T T C TG T C T CA G G C CA A A C C T C T T T

Frame 1

G	Y	T G	P S	G	P S	G	Y	T G	P A	G	P S	G	Y	T G	P V	G	G	A	A	Q	F G	S	V	S	G	Q	T S	F

Aam_R_4	G C TA CA CA G G G C C TG C TG G A C C T T C TG G C TA CA CA G G G C C T T C TG G A C C T T C TG G C TA CA C TG G G C C TG TG G G TG G CG CA G CG CA G T T CG G T T C TG T T T CA G G C CA A A C C T C T T T

Frame 1

G	Y	T G	P A	G	P S	G	Y	T G	P S	G	P S	G	Y	T G	P V	G	G	A	A	Q	F G	S	V	S	G	Q	T S	F

Aam_R_5	G C TA CA CA G G G C C TG C TG G A C C T T C TG G C TA CA CA G G G C C T T C TG G A C C T T C TG G C TA CA C TG G G C C TG TG G G TG G CG CA G CG CA G T T CG G T T C TG T T T CA G G C CA A A C C T C T T T

Frame 1

G	Y	T G	P A	G	P S	G	Y	T G	P S	G	P S	G	Y	T G	P V	G	G	A	A	Q	F G	S	V	S	G	Q	T S	F

Aam_R_6	N N N N N N N N N N N N N N N N N N N N N N N N N N N N N N N N N N N N N N N N N N N N N N N N N N N N N N N N N N N N N N N N N N N N N N N N N N N N N N N N N N N N N N N N N N N N N N N N N N N N N N N N N N N N N N N N N N N

Frame 1

? ? ? ? ? ? ? ? ? ? ? ? ? ? ? ? ? ? ? ? ? ? ? ? ? ? ? ? ? ? ? ? ? ? ? ? ? ? ?

Aar_R_1

Frame 1

Aar_R_2

Frame 1

Aar_R_3

Frame 1

Aar_R_4

Frame 1

Aar_R_5

Frame 1

Aar_R_6

Frame 1

Aar_R_7

Frame 1

Aar_R_8

Frame 1

Aar_R_9

Frame 1

580	590	600	610	620	630	640	651

CG G T CA G A CA T CA G G A T TA A C CG CA T C T T C TG G CG G A CA A G C CG C T T T CG G TG G A A C CA G TG G A G CG T C TG C TG G C G	Q	T S	G	L	T A	S	S	G	G	Q A	A	F G	G	T S	G	A	S	A	G

CG G T CA G A CA T CA G G A T T TA C CG CA T C T T C TG G CG G A CA A G C CG C T T T CG G TG G A A C CA G TG G A G CG T C TG C TG G C G	Q	T S	G	F	T A	S	S	G	G	Q A	A	F G	G	T S	G	A	S	A	G

CG G T CA G A CA T CA G G A T T TA C CG CA T C T T C TG G CG G A CA A G C CG C T T T CG G TG G A A C CA G TG G A G CG T C TG C TG G C G	Q	T S	G	F	T A	S	S	G	G	Q A	A	F G	G	T S	G	A	S	A	G

CG G T CA G A CA T CA G A A T T TA C CG CA T C T T C TG G CG G A CA A G C CG C T T T CG G TG G A A C CA G TG G A G CG T C TG C TG G C G	Q	T S	E	F	T A	S	S	G	G	Q A	A	F G	G	T S	G	A	S	A	G

CG G T CA G A CA T CA G G A T T TA C CG CA T C T T C TG G CG G A CA A G C CG C T T T CG G TG G A A C CA G TG G A G CG T C TG C TG G C G	Q	T S	G	F	T A	S	S	G	G	Q A	A	F G	G	T S	G	A	S	A	G

CG G T CA G A CA T CA G G A T T TA C CG CA T C T T C TG G CG G A CA A G C CG C T T T CG G TG G A A C CA G TG G A G CG T C TG C TG G C G	Q	T S	G	F	T A	S	S	G	G	Q A	A	F G	G	T S	G	A	S	A	G

CG G T CA G A CA T CA G G A T T TA C CG CA T C T T C TG G CG G A CA A G C CG C T T T CG G TG G A A C CA G TG G A G CG T C TG C TG G C G	Q	T S	G	F	T A	S	S	G	G	Q A	A	F G	G	T S	G	A	S	A	G

CG G T CA G A CA T CA G G A T T TA C CG CA T C T T C TG G CG G A CA A G C CG C T T T CG G TG G A A C CA G TG G A G CG T C TG C TG G C G	Q	T S	G	F	T A	S	S	G	G	Q A	A	F G	G	T S	G	A	S	A	G

CG G T CA G A CA T CA G G A T T TA C CG CA T C T T C TG G CG G A CA A G C CG C T T T CG G TG G A A C CA G TG G A G CG T C TG C TG A C G	Q	T S	G	F	T A	S	S	G	G	Q A	A	F G	G	T S	G	A	S	A	D

Aar_R_10	CG G T CA G A CA T CA G G A T T TA C CG CA T C T T C TG G CG G A CA A G C CG C T T T CG G TG G A A C CA G TG G A G CG T C TG C TG G C

Frame 1

G	Q	T S	G	F	T A	S	S	G	G	Q A	A	F G	G	T S	G	A	S	A	G

Aar_R_11	CG G T CA G A CA T CA G G A T T TA C CG CA T C T T C TG G CG G A CA A G C CG C T T T CG G TG G A A C CA G TG G A G CG T C TG C TG G C

Frame 1

G	Q	T S	G	F	T A	S	S	G	G	Q A	A	F G	G	T S	G	A	S	A	G

Aar_R_12	CG G T CA G A CA T CA G G A T T TA C CG CA T C T T C TG G CG G A CA A G C CG C T T T CG G TG G A A C CA G TG G A G CG T C TG C TG G C

Frame 1

G	Q	T S	G	F	T A	S	S	G	G	Q A	A	F G	G	T S	G	A	S	A	G

Aar_R_13	CG G T CA G A CA T CA G G A T T TA C CG CA T C T T C TG G CG G A CA A G C CG C T T T CG G TG G A A C CA G TG G A G CG T C TG C TG G C

Frame 1

G	Q	T S	G	F	T A	S	S	G	G	Q A	A	F G	G	T S	G	A	S	A	G

Aar_R_14	CG G T CA G A CA T CA G G A T T TA C CG CA T C T T C TG A TG G A CA A G C CG C T T T CG G TG G A A C CA G TG G A G CG T C TG C TG G C

Frame 1

G	Q	T S	G	F	T A	S	S	D G	Q A	A	F G	G	T S	G	A	S	A	G

Aar_R_15	CG G T CA G A CA T CA G G A T T TA C CG CA T C T T C TG A TG G A CA A G C CG C T T T CG G TG G A A C CA G TG G A G CG T C TG C TG G C

Frame 1

G	Q	T S	G	F	T A	S	S	D G	Q A	A	F G	G	T S	G	A	S	A	G

Aar_R_16	CG G T CA G A CA T CA G G A T T TA C CG CA T C T T C TA G CG G A CA A G C CG C T T T CG G TG G A A C CA G YG G A G CG T C TG C TG G C

Frame 1

G	Q	T S	G	F	T A	S	S	S	G	Q A	A	F G	G	T S	G	A	S	A	G

Aar_R_17	CG G T CA G A CA T CA G G A T T TA C CG CA T C T T C TG A TG G A CA A G C CG C T T T CG G TG G A A C CA G TG G A G CG T C TG C TG G C

Frame 1

G	Q	T S	G	F	T A	S	S	D G	Q A	A	F G	G	T S	G	A	S	A	G

Aar_R_18	CG G T CA G A CA T CA G G A T T TA C CG CA T C T T C TG A TG G A CA A G C CG C T T T CG G TG G A A C CA G TG G A G CG T C TG C TG G C

Frame 1

G	Q	T S	G	F	T A	S	S	D G	Q A	A	F G	G	T S	G	A	S	A	G

Aar_R_19	CG G T CA G A CA T CA G G A T T TA C CG CA T C T T C TG G CG G A CA A G C CG C T T T CG G TG G A A C CA G TG G A G CG T C TG C TG G C

Frame 1

G	Q	T S	G	F	T A	S	S	G	G	Q A	A	F G	G	T S	G	A	S	A	G

Aar_R_20	CG G T CA A A CA T CA G G A T T TA CA N N N N N N N N N N N N N N N N N N N N N N N N N N N N N N N N N N N N N N N N N N N N N N N N N N N N N N

Frame 1

At_R_1

Frame 1

At_R_2

Frame 1

At_R_3

Frame 1

At_R_4

Frame 1

At_R_5

Frame 1

At_R_6

Frame 1

At_R_7

Frame 1

At_R_8

Frame 1

At_R_9

Frame 1

At_R_10

Frame 1

At_R_11

Frame 1

At_R_12

Frame 1

At_R_13

Frame 1

At_R_14

Frame 1

Av_R_1

Frame 1

Av_R_2

Frame 1

Av_R_3

Frame 1

Av_R_4

Frame 1

Av_R_5

Frame 1

Av_R_6

Frame 1

Av_R_7

Frame 1

Av_R_8

Frame 1

Av_R_9

Frame 1

Av_R_10

Frame 1

G	Q	T S	G	F	T	?	?	?	?	?	?	?	?	?	?	?	?	?	?	?	?	?	?

CG G T CA A - - - T CA G G C T T TG G CG G A T C TG A TG G C C C T CA A G G TG G A T T CG G TG C CA CA G G TG G A G CG T C TG C TG G C G	Q - - - S	G	F G	G	S	D G	P	Q G	G	F G	A	T G	G	A	S	A	G

CG G T CA A - - - T CA G G C T T TG G CG G A T C TG A TG G C C C T CA A G G TG G A T T CG G TG C CA CA G G TG G A G CG T C TG C TG G C G	Q - - - S	G	F G	G	S	D G	P	Q G	G	F G	A	T G	G	A	S	A	G

CG G T CA A - - - T CA G G C T T TG G CG G A T C TG A TG G C C C T CA A G G TG G A T T CG G TG C CA CA G G TG G A G CG T C TG C TG G C G	Q - - - S	G	F G	G	S	D G	P	Q G	G	F G	A	T G	G	A	S	A	G

CG G T CA A - - - T CA G G C T T TG G CG G A T C TG C TG G C C C T CA A G G TG G A T T CG G TG C CA CA G G TG G A G CG T C TG C TG G C G	Q - - - S	G	F G	G	S	A	G	P	Q G	G	F G	A	T G	G	A	S	A	G

CG G T CA A - - - T CA G G C T T TG G CG G A T C TG C TG G C C C T CA A G G TG G A T T CG G TG C CA CA G G TG G A G CG T C TG C TG G C G	Q - - - S	G	F G	G	S	A	G	P	Q G	G	F G	A	T G	G	A	S	A	G

CG G T CA A - - - T CA G G C T T TG G CG G A T C TG C TG G C C C T CA A G G TG G A T T CG G TG C CA CA G G TG G A G CG T C TG C TG G C G	Q - - - S	G	F G	G	S	A	G	P	Q G	G	F G	A	T G	G	A	S	A	G

CG G T CA A - - - T CA G G C T T TG G CG G A T C TG C TG G C C C T CA A G G TG G A T T CG G TG C CA CA G G TG G A G CG T C TG C TG G C G	Q - - - S	G	F G	G	S	A	G	P	Q G	G	F G	A	T G	G	A	S	A	G

CG G T CA A - - - T CA G G C T T TG G CG G A T C TG A TG G C C C T CA A G G TG G A T T CG G TG C CA CA G G TG G A G CG T C TG C TG G C G	Q - - - S	G	F G	G	S	D G	P	Q G	G	F G	A	T G	G	A	S	A	G

CG G T CA A - - - T CA G G C T T TG G CG G A T C TG C TG G C C C T CA A G G TG G A T T CG G TG C CA CA G G TG G A G CG T C TG C TG G C G	Q - - - S	G	F G	G	S	A	G	P	Q G	G	F G	A	T G	G	A	S	A	G

CG G T CA A - - - T CA G G C T T TG G CG G A T C TG C TG G C C C T CA A G G TG G A T T CG G TG C CA CA G G TG G A G CG T C TG C TG G C G	Q - - - S	G	F G	G	S	A	G	P	Q G	G	F G	A	T G	G	A	S	A	G

CG G T CA A - - - T CA G G C T T TG G CG G A T C TG C TG G C C C T CA A G G TG G A T T CG G TG C CA CA G G TG G A G CG T C TG C TG G C G	Q - - - S	G	F G	G	S	A	G	P	Q G	G	F G	A	T G	G	A	S	A	G

CG G T CA A - - - T CA G G C T T TG G CG G A T C TG C TG G C C C T CA A G G TG G A T T CG G TG C CA CA G G TG G A G CG T C TG C TG G C G	Q - - - S	G	F G	G	S	A	G	P	Q G	G	F G	A	T G	G	A	S	A	G

CG G T CA A - - - T CA G G C T T TG G CG G A T C TG C TG G C C C T CA A G G TG G A T T CG G TG C CA CA G G TG G A G CG T C TG C TG G C G	Q - - - S	G	F G	G	S	A	G	P	Q G	G	F G	A	T G	G	A	S	A	G

CG G C- - - - - - T C TG G CN N N N N N N N N N N N N N N N N N N N N N N N N N N N N N N N N N N N N N N N N N N N N N N N N N N N N N N N N N N N G - - - - - - S	G	?	?	?	?	?	?	?	?	?	?	?	?	?	?	?	?	?	?	?	?

CG G T CA A A CA T CA G G C T T TA C T T CA A C TG C TG G CG C C CA A G G A G G T T T CG G T C CA A CA A C TG G CG CG CA A T C TG C C G	Q	T S	G	F	T S	T A	G	A	Q G	G	F G	P	T	T G	A	Q S	A

CG G T CA A A CA T CA G G C T T TA C T T CA A C TG C TG G CG C C CA A G G A G G T T T CG G T C CA A CA A C TG G CG CG CA A T C TG C C G	Q	T S	G	F	T S	T A	G	A	Q G	G	F G	P	T	T G	A	Q S	A

CG G T CA A A CA T CA G G C T T TA C T T CA A C TG C TG G TG C C CA A G G CG G T T T CG G T C CA A CA A C TG G CG CG CA A T C TG C C G	Q	T S	G	F	T S	T A	G	A	Q G	G	F G	P	T	T G	A	Q S	A

CG G T CA A A CA T CA G G C T T TA C T T C TA C TG C TG G TG C C CA A G G A G G T T T CG G T C CA A CA A C TG G CG CG CA A T C TG C C G	Q	T S	G	F	T S	T A	G	A	Q G	G	F G	P	T	T G	A	Q S	A

CG G T CA A A CA T CA G G C T T TA C T T CA A C TG C TG G TG C C CA A G G CG G T T T CG G T C CA A CA A C TG G A G CG CA A T C TG C C G	Q	T S	G	F	T S	T A	G	A	Q G	G	F G	P	T	T G	A	Q S	A

CG G T CA A A CA T CA G G C T T TA C T T CA A C TG C TG G CG C C CA A G G A G G T T T CG G T C CA A CA A C TG G CG CG CA A T C TG C C G	Q	T S	G	F	T S	T A	G	A	Q G	G	F G	P	T	T G	A	Q S	A

CG G T CA A A CA T CA G G C T T TA C T T CA A C TG C TG G TG C C CA A G G CG G T T T CG G T C CA A CA A C TG G CG CG CA A T C TG C C G	Q	T S	G	F	T S	T A	G	A	Q G	G	F G	P	T	T G	A	Q S	A

CG G T CA A A CA T CA G G C T T TA C T T CA A C TG C TG G CG C C CA A G G A G G T T T CG G T C CA A TA A C TG G CG CG CA A T C TG C C G	Q	T S	G	F	T S	T A	G	A	Q G	G	F G	P I	T G	A	Q S	A

CG G T CA A A CA T CA G G C T T TA C T T CA A C TG C TG G CG C C CA A G G A G G T T T CG G T C CA A CA A C TG G CG CG CA A T C TG C C G	Q	T S	G	F	T S	T A	G	A	Q G	G	F G	P	T	T G	A	Q S	A

CG G T CA A A CA T CA G G C T TG A C C T C T T C TG C TG C TA G C CA A T CG G A T T T CN N N N N N N N N N N N N N N N N N N N N N N N N N N G	Q	T S	G	L	T S	S	A	A	S	Q S	D	F	?	?	?	?	?	?	?	?	?

Aam_R_1	CG G T CA A A CA T CA G G C T T TA C CG CA T C TA C TG G CG G A CA A G C CG CG T T TG G TG CA A CA A G TG G A T CG T C TG C TG G C

Frame 1

G	Q	T S	G	F	T A	S	T G	G	Q A	A	F G	A	T S	G	S	S	A	G

Aam_R_2	CG G T CA A A CA T CA G G C T T TA C CG CA T C TA C TG G CG G A CA A G C CG CG T T TG G TG CA A CA A G TG G A T CG T C TG C TG G C

Frame 1

G	Q	T S	G	F	T A	S	T G	G	Q A	A	F G	A	T S	G	S	S	A	G

Aam_R_3	CG G T CA A A CA T CA G G C T T TA C CG CA T C TA C TG G CG G A CA A G C CG CG T T TG G TG CA A CA A G TG G A T CG T C TG C TG G C

Frame 1

G	Q	T S	G	F	T A	S	T G	G	Q A	A	F G	A	T S	G	S	S	A	G

Aam_R_4	CG G T CA A A CA T CA G G C T T TA C CG CA T C TA C TG G CG G A CA A G C CG CG T T TG G TG CA A CA A G TG G A T CG T C TG C TG G C

Frame 1

G	Q	T S	G	F	T A	S	T G	G	Q A	A	F G	A	T S	G	S	S	A	G

Aam_R_5	CG G T CA A A CA T CA G G C T T TA C CG CA T C TA C TG G CG G A CA A G C CG CG T T TG G TG CA A CA A G TG G A T CG T C TG C TG G C

Frame 1

G	Q	T S	G	F	T A	S	T G	G	Q A	A	F G	A	T S	G	S	S	A	G

Aam_R_6	N N N N N N N N N N N N N N N N N N N N N N N N N N N N N N N N N N N N N N N N N N N N N N N N N N N N N N N N N N N N N N N N N N N N N N N N N N N N

Frame 1

? ? ? ? ? ? ? ? ? ? ? ? ? ? ? ? ? ? ? ? ? ? ? ? ?

**Supplementary Figure S3. Nucleotide alignment of Araneidae *AcSp1* iterated repeats.** Alignment position numbers shown in increments of ten. Frame 1 translation is shown under nucleotide sequences. Alignment prepared with Geneious v6.1.6 (Biomatters Ltd., Auckland, NZ) and available on TreeBASE ([http://purl.org/phylo/treebase/phylows/study/TB2:](http://purl.org/phylo/treebase/phylows/study/TB2%3AS15355))S15355). Abbreviations and accession numbers in Table S3.

>Aar_NtermV2

AGTCCAGTACGATGCGGTGCAAAGCGCGTCAACTCTGTCCAGGAGTCCTTGGGCCAATCCGGCCAAAGCTGGTTCTTTGATGAACTGTCTCATGAGCAGAAT CGCCAGTTCAAATGTCCTACCTCAACAAGATAAAGAAGATCTGGAATCCATTATGGACACACTGATGTCGGCAATTAAAGGAGCGAGTGCCAAAGGTAAAAG CTCTGCAGCTCAATTGAAGGCAATCAATATGGCGGTTGCATCTTCCCTGGCAGAAATAGTCGTTGCCGAAGATGCAGGAAACCAGGCCAGCATCGCAGTAAA GACTCAGGCCCTTACAGGTGCTCTGGGACAGTGCTTCCAAGCAGTCATGGGCACTGTCGACAGGAAATTCATCAATGAAATAAACGACTTGATAACAATGTT TGCAAAAGAAGCTGCCAGTGAAAGCAACGAAATACCAGATCAAGAATCTTCCTCCGCAAGCGGATCATCTGCTTCAACGTCGTATCAAGCAGCAAGCCAA--

-------GGCTCGTTTCAAACTACAGGTGGAATAAGTACATACTCTCAACCTCAAGTTTCACAGCCAGAATCATTC--------------------------

-GGAGCTGGG------CAACAGATATTACAACCTTCT-----------------------------------------------------------------

----------------------GGCTACACTGGACCTTCCGGGTACACCGGACCTTCTAGCAACAATGGGACTGTGGGCGGCGGAGCGCAG------TTC--

------------------- GGTTCCGTTTCAGGCCAAACCTCTTACGGTCAAACGTCAGGATCTGCCGCATCTACTGGAGGCCAAGCCTCTTTCGGTGCACCAAGCGGAGCGTCAGCTGGC

>Aar_NtermV3

AGTCCAGTACGATGCGGTGCAAAGCGCGTCACCTCTGTCCAGGAGTCCTTGGGCCAATCCGGCCAAAGCTGGTTCTTTTATGAACTGTCTAATGAGCAGAAT CGCCAGTTCAAATGTCCTGCCTCAACAAGATAAAGAAGATCTGGAATCCATTATGGACACACTGATGTCGGCAATTAAAGGAGCGAGTGCCAAAGGTAAAAG CTCTGCAGCTCAATTGAAGGCAATCAATATGGCGGTTGCATCTTCCCTGGCAGAAATAGTCGTTGCCGAAGATGCAGGAAACCAGGCCAGCATCGCAGTAAA GACTCAGGCCCTTACAGGTGCTCTGGGACAGTGCTTCCAAGCAGTCATGGGCACTGTCGACAGGAAATTCATCAATGAAATAAACGACTTGATAACAATGTT TGCAAAAGAAGCTGCCAGTGAAAGCAACGAAATACCAGATCAAGAATCTTCCTCCGCAAGCGGATCATCTGCTTCAACGTCGTATCAAGCAGCAAGCCAA--

-------GGCTCGTTTCAAACTACAGGTGGAATAAGTACATACTCTCAACCTCAAGTTTCACAGCCAGAATCATTC--------------------------

-GGAGCTGGG------CAACAGATATTACAACCTTCT-----------------------------------------------------------------

----------------------GGCTACACTGGACCTTCCGGGTACACCGGACCTTCTAGCAACAATGGGACTGTGGGCGGCGGAGCGCAG------TTC--

------------------- GGTTCCGTTTCAGGCCAAACCTCTTACGGTCAAACGTCAGGATCTGCCGCATCTACTGGAGGCCAAGCCTCTTTCGGTGCACCAAGCGGAGCGTCAGCTGGC

>Aar_NtermV4

AGTCCAGTACGATGCGGTGCAAAGCGCGTCAACTCTGTCCAGGAGTCCTTGGGCCAATCCGGCCAAAGCTGGTTCTTTGATGAACTGTCTCATGAGCAGAAT CGCCAGTTCAAATGTCCTACCTCAACAAGATAAAGAAGATCTGGAATCCATTATGGACACACTGATGTCGGCAATTAAAGGAGCGAGTGCCAAAGGTAAAAG CTCTGCAGCTCAATTGAAGGCAATCAATATGGCGGTTGCATCTTCCCTGGCAGAAATAGTCGTTGCCGAAGATGCAGGAAACCAGGCCAGCATCGCAGTAAA GACTCAGGCCCTTACAGGTGCTCTGGGACAGTGCTTCCAAGCAGTCATGGGCACTGTCGACAGGAAATTCATCAATGAAATAAACGACTTGATAACAATGTT TGCAAAAGAAGCTGCCAGTGAAAGCAACGAAATACCAGATCAAGAATCTTTCTCCGCAAGCGGATCATCTGCTTCATCGTCGTTTCAAGCAGTTAGCCAA--

-------GGCTCGTTTCAAACTACAGGTGGAATAAGTACATACTCTCAACCTCAAGTTTCACAGCCAGAATCATTC--------------------------

-GGAGCTGGG------CAACAGATATTACAACCTTCT-----------------------------------------------------------------

----------------------GGCTACACTGGACCTTCCGGGTACACCGGACTTTCTAGCAACAATGGGACTGTGGGCGGCGGAGCGCAG------TTC--

------------------- GGTTCCGTTTCAGGCCAAACCTCTTACGGTCAAACGTCAGGATCTGCCGCATCTACTGGAGGCCAAGCCTCTTTCGGTGCACCAAGCGGAGCGTCAGCTGGC

>Aar_NtermV7

AGTCCAGTACGATGCGGTGCAAAGCGCGTCAACTCTGTCCAGGAGTCCTTGGGCCAATCCRGCCAAAGCTGRTTCTTTGATGAACTGTCTCATGAGCAGAAT CGCCAGTTCAAATGYCCTGCCTCAACAAGATAAAGAAGATCTGGAATCCATTATGGACACACTGATGTCGGCAATTAAAGGAGCGAGTGCCAAAGGTAAAAG CTCTGCAGCGCAGTTGCAGGCGATAAATATGGCTGTTGCTTCTTCCCTGGCAGAAATAGTCGTTGCTGAAGATGCAGGCAATCAAGCAAGCATCGCAGTAAA GACTCAGGCCCTTACAGGTGCTCTGGGACAATGCTTCCAAGCAGYCATGGGCACAGTCGACAGGAAATTCATCAATGAAATAAATGACTTGATAACAATGTT TGCAAAAGAAGCTGCCAGTGAAAGCAACGAAATACCAGATCAAGAATCTTCCTCCGCAAGCGGATCATCTGCTTCAACGTCGTATCAAGCAGCAAGCCAA--

-------GGCTCGTTTCAAACTACAGGTGGAATAAGTACATACYCTCAACCTCAAGTTTCACAGCCAGAATCATTC--------------------------

-GGAGCTGGG------CAACAGATATTACAACCTTCT-----------------------------------------------------------------

----------------------GGCTACACTGGACCTTCCGGGTACACCGGACCTTCTAGCAACAATGGGACTGTGGGCGGCGGAGCGCAG------TTC--

------------------- GGTTCCGTTTCAGGYCAAACCTCTTACGGTCAAACGTCAGGATCTGCCGCATCTACTGGAGGCCAAGCCTCTTTCGGTGCACCAAGCGGAGCGTCAGCTGGC

>At_NtermV14

AGTACAGTACGATGSRGYGMAAAGYGCKTCWSCTWYSTCRAGRAGTCCYTGGGCCAATCCAGCYAAAGCAGGTKCGTTGATGAAYTGTCTSATYAACARAAT TGCCAGCTCMAATGTCTTGCCTCAACAAGAAAAAGAAGACTTGGAATCCATYATGGACACACTGATGTCTGCWATAAAAGGTGCRAGTGCCAAAGGTAAAAG CTCTGCAGCACAGTTGCAGGCSATYAATATGGCAGTTGCATCTTCCCTGGCAGAAYTAGTCGTTGCTGAAGAYGCWGGCAATCAGGCAAGCATMGCAGTAAA GACGCAGGCCCTTWCAGGWGCKCTSSRGCAGTGTTTCCRAGCAGTCATGGGAACGGTCGATAGGAAATTCATCAACGAAATAAATGATTTGATAAGAATGTT TGCTAGAGAAGCAGCCAGCGAAACGAATGAAATACCAGACCAAGGTGCTTTATATGGAGCCGGTTCTTCAGCCTCATCATCATTTCAAGCAACAGGTCAAAA CTACCAAGACTCGTCTCGAACAGCTAGTGGA-----------------------------------------------------------------------

-------------------------------------------TCAACTGATTCTTCAACCAGTGATACTGCAATTCGCACCACA-----------------

----------------------GGATATACAGGCTACACAGGACAACAGTTCGGATCA---------------GTTTCAGGC------ CAAACATCTTACGATCAGGGATCGGGCTATACAGGATCTGCTGCCGGACAAGGAGGTTACGGACAAACATCTGGTTTTGGCGGACCCGCTGGAGGACCGGGC GCTTTCGGCCCCACCACTGGAGCGTCAGCCGGC

>Aar_NtermV1

AGTCCAGTACGATGCGGTGCAAAGCGCGTCAACTCTGTCCAGGAGTCCTTGGGCCAATCCGGCCAAAGCTGGTTCTTTGATGAACTGTCTCATGAGCAGAAT CGCCAGTTCAAATGTCCTGCCTCAACAAGATAAAGAAGATCTGGAATCCATTATGGACACACTGATGTCGGCAATTAAAGGAGCGAGTGCCAAAGGTAAAAG CTCTGCAGCGCAGTTGCAGGCGATAAATATGGCTGTTGCTTCTTCCCTGGCAGAAATAGTCGTTGCTGAAGATGCAGGCAATCAAGCAAGCATCGCAGTAAA GACTCAGGCCCTTACAGGTGCTCTGGGACAATGCTTCCAAGCAGTCATGGGCACAGTCGACAGGAAATTCATCAATGAAATAAATGACTTGATAACAATGTT TGCAAAAGAAGCTGCCAGTGAAAGCAACGAAATACCAGATCAAGAATCTTTCTCCGCAAGCGGATCATCTGCTTCAACGTCGTATCAAGCAGCAAGCCAAAG TTTCCAAGGGTCATCACAAACAACAGGCGGAATTAGTACATATCCCCAGCCTCAAGTTTCGCAATCAGCAACATTCGGCATTGGGTCAGTTGGAAATACTGG TGGTGCTGGATCATATGGTGGGGGATTGCAATCAGGCTCAGTAACCGGTGGAACTTCTTTTGGTCAAACATCCACTTTATCAACTAGTTTGTCTCAGACAGG AGGTATATCTACCAGTGGGGCCGGTTACAACGGACCAGTTGGATACAGAGGGCCT------------------GTTGGTGGAGGATCACAG------TTC--

-------------------GGAACTGATTCAAGCCAAACAG-------------------------------------------------------------

-------------------

>Aar_NtermV5

AGTCCAGTACGATGCGGTGCAAAGCGCGTCAACTCTGTCCAGGAGTCCTTGGGCCAATCCGGCC>AarGCTGGTTCTTTGATGAACTGTCTCATGAGCAGAA TCGCCAGTTCAAATGTCCTGCCTCAACAAGATAAAGAAGATCTGGAATCCATTATGGACACACTGATGTCGGCAATTAAAGGAGCGAGTGCCAAAGGTAAAA GCTCTGCAGCTCAATTGAAGGCARTCAATATGGCGGTTGCATCTTCCCTGGCAGAAATAGTCGTTGCCGAAGATGCAGGAAACCAGGCCAGCATCGCAGTAA AGACTCAGGCCCTTACAGGTGCTCTGGGACAGTGCTTCCAAGCAGTCATGGGCACTGTCGACAGGAAATTCATCAATGAAATAAACGACTTGATAACAATGT TTGCAAAAGAAGCTGCCAGTGAAAGCAACGAAATACCAGATCAAGAATCTTTCTCCGCAAGCGGATCATCTGCTTCAACGTCGTATCAAGCAGCAAGCCAAA GTTTCCAAGGGTCATCACAAACAACAGGCGGAATTAGTACATATCCCCAGCCTCAAGTTTCGCAATCAGCAACATTCGGCATTGGGTCAGTTGGAAATACTG GTGGTGCTGGATCATATGGTGGGGGATTGCAATCAGGCTCAGTAACCGGTGGAACTTCTTTTGGTCAAACATCCACTTTATCAACTAGTTTGTCTCAGACAG GAGGTATATCTACCAGTGGGGCCGGTTACAACGGACCAGTTGGATACAGAGGGCCT------------------GTTGGTGGAGGATCACAG------TTC-

--------------------GGAACTGATTCAAGCCAAACAG------------------------------------------------------------

--------------------

>Aar_NtermV6

AGTCCAGTACGATGCGGTGCAAAGCGCGTCAACTCTGTCCAGGAGTCCTTGGGCCAATCCGGCCAAAGCTGGTTCTTTGATGAACTGTCTCATGAGCAGAAT CGCCAGTTCAAATGTCCTACCTCAACAAGATAAAGAAGATCTGGAATCCATTATGGACACACTGATGTCGGCAATTAAAGGAGCGAGTGCCAAAGGTAAAAG CTCTGCAGCTCAATTGAAGGCAATCAATATGGCGGTTGCATCTTCCCTGGCAGAAATAGTCGTTGCCGAAGATGCAGGAAACCAGGCCAGCATCGCAGTAAA GACTCAGGCCCTTACAGGTGCTCTGGGACAGTGCTTCCAAGCAGTCATGGGCACTGTCGACAGGAAATTCATCAATGAAATAAACGACTTGATAACAATGTT TGCAAAAGAAGCTGCCAGTGAAAGCAACGAAATACCAGATCAAGAATCTTTCTCCGCAAGCGGATCATCTGCTTCAACGTCGTATCAAGCAGCAAGCCAAAG TTTCCAAGGGTCATCACAAACAACAGGCGGAATTAGTACATATCCCCAGCCTCAAGTTTCGCAATCAGCAACATTCGGCATTGGGTCAGTTGGAAATACTGG TGGTGCTGGATCATATGGTGGGGGATTGCAATCAGGCTCAGTAACCGGTGGAACTTCTTTTGGTCAAACATCCACTTTATCAACTAGTTTGTCTCAGACAGG AGGTATATCTACCAGTGGGGCCGGTTACAACGGACCAGTTGGATACAGAGGGCCT------------------GTTGGTGGAGGATCACAG------TTC--

-------------------GGAACTGATTCAAGCCAAACAG-------------------------------------------------------------

-------------------

>Aau_NtermV1

GGTACAGTACGATGCGGTGCAAAGCGCGTCACCTTTGTCAAGGAGTCCTTGGGCCAATCCAGCCAAAGCAGGATCGTTGATGAACTGTCTTATCAATAGAAT CTCTAGCTCAAATGTCTTGCCTCAACAAGATAAAGAAGACTTGGAATCCATTATGGACACACTGATGTCTGCAATAACAGGGGCCAGTGCGAAAGGTAAAAG CTCTGCAGCGCAGTTGCAGGCCATCAATATGGCAGTTGCATCTTCCCTGGCAGAACTAGTCGTTGCCGAAGACGCAGGCAATCAGGCAAGCATTGCAGTAAA GACGCAGGCCCTTTCAGGAGCACTGCAGCAATGTTTCCAAGCAGTGATGGGCACGGTCGACCGAAAATTCATCAACGAAATAAACGATTTGATAACAATGTT TGCCAGAGAAGCTGCCAGCGAAAGTAATGAAATTCCAGACCAAGCATCATTCTCCGCAAGCGGATCATCTGCTGCATCTTCCTACCAAGCAACAAGTCAAAG TTTCCAAGGATCATCTCAAACTACAGGCGGATTTAGTTCATACTCCCAACCTCAAGTTCCGCAGCCAGCAACGTTCGACACTGGCTCAGTCGGATACACTGG TGGAGCTGGGACATATGATGGAGGATTACAATCAGGCTCCATTACAGGCGGAACTTCTTTCGGTCAAACAACTGCTGCATCAACCAGTTTCTCTCAAACAGG AGCTTCAACTGCTAGTGGCGCCGGTTACACGGGGCCTACTGGCTATACTGGACCT------------------GTGGGTGGAGGATCACAG------TTC--

-------------------GGTTCTGATTCCGGCCAAACTGCTAGT--------------------------------------------------------

-------------------

>Aau_NtermV2

GGTACAGTACGATGCGGTGCAAAGCGCGTCACCTTTGTCAAGGAGTCCTTGGGCCAATCCAGCCAAAGCAGGATCGTTGATGAACTGTCTTATCAACAGAAT CTCTAGCTCAAATGTCTTGCCTCAACAAGATAAAGAAGACTTGGAATCCATTATGGACACACTGATGTCTGCAATAACAGGGGCCAGTGCGAAAGGTAAAAG CTCTGCAGCGCAGTTGCAGGCCATCAATATGGCAGTTGCATCTTCCCTGGCAGAACTAGTCGTTGCCGAAGACGCAGGCAATCAGGCAAGCATTGCAGTAAA GACGCAGGCCCTTTCAGGAGCACTGCAGCAATGTTTCCAAGCAGTGATGGGCACGGTCGACCGAAAATTCATCAACGAAATAAACGATTTGATAACAATGTT TGCCAGAGAAGCTGCCAGCGAAAGTAATGAAATTCCAGACCAAGCATCATTCTCCGCAAGCGGATCATCTGCTGCATCTTCCTACCAAGCAACAAGTCAAAG TTTCCAAGGATCATCTCAAACTACAGGCGGATTTAGTTCATACTCCCAACCTCAAGTTCCGCAGCCAGCAACGTTCGACACTGGCTCAGTCGGATACACTGG TGGAGCTGGGACATATGATGGAGGATTACAATCAGGCTCCATTACAGGCGGAACTTCTTTCGGTCAAACAACTGCTGCATCAACCAGTTTCTCTCAAACAGG AGCTTCAACTGCTAGTGGCGCCGGTTACACGGGGCCTACTGGCTATACTGGACCT------------------GTGGGTGGAGGATCACAG------TTC--

-------------------GGTTCTGATTCCGGCCAAACTGCTAGT--------------------------------------------------------

-------------------

>Aau_NtermV4

GGTACAGTACGATGCGGTGCAAAGCGCGTCACCTTTGTCAAGGAGTCCTTGGGCCAATCCAGCCAAAGCAGGATCGTTGATGAACTGTCTTATCAAYAGAAT CTCTAGCTCAAATGTCTTGCCTCAACAAGATAAAGAAGACTTGGAATCCATTATGGACACACTGATGTCTGCAATAACAGGGGCCAGTGCGAAAGGTAAAAG CTCTGCAGCGCAGTTGCAGGCCATCAATATGGCAGTTGCATCTTCCCTGGCAGAACTAGTCGTTGCCGAAGACGCAGGCAATCAGGCAAGCATTGCAGTAAA GACGCAGGCCCTTTCAGGAGCACTGCAGCAATGTTTCCAAGCAGTGATGGGCACGGTCGACCGAAAATTCATCAACGAAATAAACGATTTGATAACAATGTT TGCCAGAGAAGCTGCCAGCGAAAGTAATGAAATTCCAGACCAAGGATCATTCTCCGCAAGCGGATCATCTGCTGCATCTTCCTACCAAGCAACAAGTCAAAG TTTCCAAGGATCATCTCAAACTACAGGCGGATTTAGTTCATACTCCCAACCTCAAGTTCCGCAGCCAGCAACGTTCGACACTGGCTCAGTCGGATACACTGG TGGAGCTGGGACATATGATGGAGGATTACAATCAGGCTCCATTACAGGCGGAACTTCTTTCGGTCAAACAACTGCTGCATCAACCAGTTTCTCTCAAACAGG AGCTTCAACTGCTAGTGGCGCCGGTTACACGGGGCCTACTGGCTATACTGGACCT------------------GTGGGTGGAGGATCACAG------TTC--

-------------------GGTTCTGATTCCGGCCAAACTGCTAGT--------------------------------------------------------

-------------------

>Aau_NtermV5

GGTACAGTACGATGCGGTGCAAAGCGCGTCACCTTTGTCAAGGAGTCCTTGGGCCAATCCAGCCAAAGCAGGATCGTTGATGAACTGTCTTATCAATAGAAT CTCTAGCTCAAATGTCTTGCCTCAACAAGATAAAGAAGACTTGGAATCCATTATGGACACACTGATGTCTGCAATAACAGGGGCCAGTGCGAAAGGTAAAAG CTCTGCAGCGCAGTTGCAGGCCATCAATATGGCAGTTGCATCTTCCCTGGCAGAACTAGTCGTTGCCGAAGACGCAGGCAATCAGGCAAGCATTGCAGTAAA GACGCAGGCCCTTTCAGGAGCACTGCAGCAATGTTTCCAAGCAGTGATGGGCACGGTCGACCGAAAATTCATCAACGAAATAAACGATTTGATAACAATGTT TGCCAGAGAAGCTGCCAGCGAAAGTAATGAAATTCCAGACCAAGGATCATTCTCCGCAAGCGGATCATCTGCTGCATCTTCCTACCAAGCAACAAGTCAAAG TTTCCAAGGATCATCTCAAACTACAGGCGGATTTAGTTCATACTCCCAACCTCAAGTTCCGCAGCCAGCAACGTTCGACACTGGTTCAGTCGGATACACTGG TGGAGCTGGGACATATGTTGGAGGATTACAATCAGGCTCCATTACAGGCGGAACTTCTTTCGGTCAAACAACTGCTGCATCAACCAGTTTCTCTCAAACAGG AGCTTCAACTGCTAGTGGCGCCGGTTACACGGGGCCTACTGGCTATACTGGACCT------------------GTGGGTGGAGGATCACAG------TTC--

-------------------GGTTCTGATTCCGGCCAAACTGCTAGT--------------------------------------------------------

-------------------

>Aau_NtermV6

GGTACAGTACGATGCGGTGCAAAGCGCGTCACCTTTGTCAAGGAGTCCTTGGGCCAATCCAGCCAAAGCAGGATCGTTGATGAACTGTCTTATCAATAGAAT CTCTAGCTCAAATGTCTTGCCTCAACAAGATAAAGARGACTTGGAATCCATTATGGACACACTGATGTCTGCAATAACAGGGGCCAGTGCGAAAGGTAAAAG CTCTGCAGCGCAGTTGCAGGCCATCAATATGGCAGTTGCATCTTCCCTGGCAGAACTAGTCGTTGCCGAAGACGCAGGCAATCAGGCAAGCATTGCAGTAAA GACGCAGGCCCTTTCAGGAGCACTGCAGCAATGTTTCCAAGCAGTGATGGGCACGGTCGACCGAAAATTCATCAACGAAATAAACGATTTGATAACAATGTT TGCCAGAGAAGCTGCCAGCGAAAGTAATGAAATTCCAGACCAAGCATCATTCTCCGCAAGCGGATCATCTGCTGCATCTTCCTACCAAGCAACAAGTCRAAG TTTCCAAGGATCATCTCAAACTACAGGCGGATTTAGTTCATACTCCCAACCTCAAGTTCCGCAGYCAGCAACGTTCGACACTGGTTCAGTCGGATACACTGG TGGAGCTGGGACATATGTTGGAGGATTACAATCAGGCTCCATTACAGGCGGAACTTCTTTCGGTCAAACAACTGCTGCATCAACCAGTTTCTCTCAAACAGG AGCTTCAACTGCTAGTGGCGCCGGTTACACGGGGCCTACTGGCTATACTGGACCT------------------GTGGGTGGAGGATCACAG------TTC--

-------------------GGTTCTGATTCCGGCCAAACTGCTAGT--------------------------------------------------------

-------------------

>Aau_NtermV3

GGTACAGTACGATGCGGTGCAAAGCGCGTCACCTTTGTCAAGGAGTCCTTGGGCCAATCCAGCCAAAGCAGGATCGTTGATGAACTGTCTTATCAACAGAAT CTCTAGCTCAAATGTCTTGCCTCAACAAGATAAAGAAGACTTGGAATCCATTATGGACACACTGATGTCTGCAATAACAGGGGCCAGTGCGAAAGGTAAAAG CTCTGCAGCGCAGTTGCAGGCCATCAATATGGCAGTTGCATCTTCCCTGGCAGAACTAGTCGTTGCCGAAGACGCAGGCAATCAGGCAAGCATTGCAGTAAA GACGCAGGCCCTTTCAGGAGCACTGCAGCAATGTTTCCAAGCAGTGATGGGCACGGTCGACCGAAAATTCATCAACGAAATAAACGATTTGATAACAATGTT TGCCAGAGAAGCTGCCAGCGAAAGTAATGAAATTCCAGACCAAGGATCATTCTCCGCAAGCGGATCATCTGCTGCATCTTCCTACCAAGCAACAAGTCAAAG TTTCCAAGGATCATCTCAAACTACAGGCGGATTTAGTTCATACTCCCAACCTCAAGTTCCGCAGCCAGCAACGTTCGACACTGGTTCAGTCGGATACACTGG TGGAGCTGGGACATATGTTGGAGGATTACAATCAGGCTCCATTACAGGCGGAACTTCTTTCGGTCAAACAACTGCTGCATCAACCAGTTTCTCTCAAACAGG AGCTTCAACTGCTAGTGGCGCCGGTTACACGGGGCCTACTGGCTATACTGGACCT------------------GTGGGTGGAGGATCACAG------TTC--

-------------------GGTTCTGATTCCGGCCAAACTGCTAGT--------------------------------------------------------

-------------------

>At_NtermV6

AGTACAGTACGATGCGGTGCAAAGTGCTTCACCTATCTCGAGGAGCCCTTGGGCCAATCCAGCTAAAGCAGGTTCGTTGATGAACTGTCTCATTAACAGAAT TGCCAGCTCCAATGTCTTGCCTCAACAAGAAAAAGAAGACTTGGAATCCATTATGGACACACTGATGTCTGCTATAAAAGGTGCGAGTGCCAAAGGTAAAAG CTCTGCAGCTCAGTTGCAGGCTATTAATATGGCAGTTGCATCTTCCCTAGCTGAATTAGTCGTTGCTGAAGACGCTGGCAATCAGGCAAGCATCGCAGTAAA GACGCAGGCCCTTACAGGTGCCCTGGGGCAGTGTTTCCAAGCGGTCATGGGCACAGTCGACAGGAAATTCATCAACGAAATAAACGATTTGATAACAATGTT TGCCAGAGAAGCTGCAAGTGACAGCAATGAAATTGAAGACCAAGGATCATTCTCCACAAGTGGATCATCAACTTCATCGTCGTATCAAGCAGCAAGTCAGAG TTTA------------------------------------------------------------------------ GGCATTGGGTCAGTCGGTTACACTGGTGGTGCTGGGCCATATGGGGGAGGACTTCAGTCAAGCTCATTTTCAGGCGGAACTTCTTTCGGTCAAACCGCAGCT GCATCAACCAGTTTCTCTCAGACAGGAGCTACTTCTACGAGCGGTGACAGTTACACAGGGCCTGTAGGCTATAGTGGACCT------------------ CTGGGTGGAGGAGCACAG------TTC---------------------GGTTCTGTTTCCAGCCAAACAGCTAGT---------------------------

------------------------------------------------

>At_NtermV8

AGTACAGTACGATGCGGTGCAAAGTGCTTCGACTATCTCGAGGAGTCCTTGGGCCAATCCAGCTAAAGCAGGTTCGTTGATGAACTGTCTCATTAACAGAAT TGCCAGCTCCAATGTCTTGCCTCAACAAGAAAAAGAAGACTTGGAATCCATTATGGACACACTGATGTCTGCTATAAAAGGTGCAAGTGCCAAAGGTAAAAG CTCTGCAGCACAGTTGCAGGCTATTAATATGGCAGTTGCATCTTCCCTGGCAGAACTAGTCGTTGCTGAAGATGCAGGCAATCAGGCAAGCATCGCAGTAAA GACGCAGGCCCTTTCAGGAGCTCTACAGCAGTGTTTCCAAGCGGTCATGGGCACAGTCGACCGGAAATTCATCAACGAAATAAACGATTTGATAACAATGTT TGCCAGAGAAGCTGCAAGTGAAAGCAATGAAATTCAAGATCAAGGATCATTCTCCACAAGTGGATCATCAGCTTCATCATCGTATCAAGCAGCAAGTCAGAG TTCA------------------------------------------------------------------------ GGCATTGGGTCAGTCGGATACACTGGTGGTGCTGGGCCATATGGTGGAGGACTTCAATCAAGCTCATTTTCAGGCGGAACTTCTTTCGGTCAAACCGCAGCT GCATCAACCAGTTTCTCTCAGACAGGAGCTACTTCTACGAGCGGTGACAGTTACACAGGGCCTGTAGGCTATAGTGGACCT------------------ CTGGGTGGAGGAGCACAG------TTC---------------------GGTTCTGTTTCCAGCCAAACAGCTAGT---------------------------

------------------------------------------------

>At_NtermV9

AGTACAGTACGATGCGGTGCAAAGTGCTTCACCTATCTCGAGGAGTCCTTGGGCCAATCCAGCTAAAGCAGGTTCGTTGATGAACTGTCTCATTAACAGAAT TGCCAGCTCCAATGTCTTGCCTCAACAAGAAAAAGAAGACTTGGAATCCATTATGGACACACTGATGTCTGCTATAAAAGGTGCGAGTGCCAAAGGTAAAAG CTCTGCAGCACAGTTGCAGGCTATTAATATGGCAGTTGCATCTTCCCTGGCAGAACTAGTCGTTGCTGAAGATGCAGGCAATCAGGCAAGCATCGCAGTAAA GACGCAGGCCCTTTCAGGAGCTCTCCAGCAGTGTTTCCAAGCGGTCATGGGCACAGTCGACCGGAAATTCATCAACGAAATAAACGATTTGATAACAATGTT TGCCAGAGAAGCTGCAAGTGAAAGCAATGAAATTCAAGATCAAGGATCATTCTCCACAAGTGGATCATCAGCTTCATCATCGTATCAAGCAGCAAGTCAGAG TTCA------------------------------------------------------------------------ GGCATTGGGTCAGTCGGATACACTGGTGGTGCTGGGCCATATGGTGGRGGACTTCAATCAAGCTCATTTTCAGGCGGAACTTCTTTCGGTCAAACCGCAGCT GCATCAACCAGTTTCTCTCAGACAGGAGCTACTTCTACGAGCGGTGACAGTTACACAGGGCCTGTAGGCTATAGTGGACCT------------------ CTGGGTGGAGGAGCACAG------TTC---------------------GGTTCTGTTTCCAGCCAAACAGCTAGT---------------------------

------------------------------------------------

>At_NtermV10

AGTACAGTACGATGCGGTGCAAAGTGCTTCACCTATCTCGAGGAGTCCTTGGGCCAATCCAGCTAAAGCAGGTTCGTTGATGAACTGTCTCATTAACAGAAT TGCCAGCTCCAATGTCTTGCCTCAACAAGAAAAAGAAGACTTGGAATCCATTATGGACACACTGATGTCTGCTATAAAAGGTGCGAGTGCCAAAGGTAAAAG CTCTGCAGCACAGTTGCAGGCTATTAATATGGCAGTTGCATCTTCCCTGGCAGAACTAGTCGTTGCTGAAGATGCAGGCAATCAGGCAAGCATCGCAGTAAA GACGCAGGCCCTTTCAGGAGCTCTCCAGCAGTGTTTCCAAGCGGTCATGGGCACAGTCGACCGGAAATTCATCAACGAAATAAACGATTTGATAACAATGTT TGCCAGAGAAGCTGCAAGTGAAAGCAATGACATTCAAGATCAAGGATCATTCTCCACAAGTGGATCATCAGCTTCATCATCGTATCAAGCAGCAAGTCAGAG TTCA------------------------------------------------------------------------ GGCATTGGGTCAGTCGGATACACTGGTGGTGCTGGGCCATATGGTGGGGGACTTCAATCAAGCTCATTTTCAGGCGGAACTTCTTTCGGTCAAACCGCAGCT GCATCAACCAGTTTCTCTCAGACAGGAGCTACTTCTACGAGCGGTGACAGTTACACAGGCCCTGTTGGCTATAGTGGACCT------------------ CTGGGTGGAGGAGCACAG------TTC---------------------GGTTCTGCTTCCAGCCAAACAGCTAGT---------------------------

------------------------------------------------

>At_NtermV11

AGTACAGTACGATGCGGTGCAAAGTGCTTCACCTATCTCGAGGAGTCCTTGGGCCAATCCAGCTAAAGCAGGTTCGTTGATGAACTGTCTCATTAACAGAAT TGCCAGCTCCAATGTCTTGCCTCAACAAGAAAAAGAAGACTTGGAATCCATTATGGACACACTGATGTCTGCTATAAAAGGTGCGAGTGCCAAAGGTAAAAG CTCTGCAGCACAGTTGCAGGCTATTAATATGGCAGTTGCATCTTCCCTGGCAGAACTAGTCGTTGCTGAAGATGCAGGCAATCAGGCAAGCATCGCAGTAAA GACGCAGGCCCTTTCAGGAGCTCTCCAGCAGTGTTTCCAAGCGGTCATGGGCACAGTCGACCGGAAATTCATCAACGAAATAAACGATTTGATAACAATGTT TGCCAGAGAAGCTGCAAGTGAAAGCAATGAAATTCAAGATCAAGGATCATTCTCCACAAGTGGATCATCAGCTTCATCATCGTATCAAGCAGCAAGTCAGAG TTCA------------------------------------------------------------------------ GGCATTGGGTCAGTCGGATCCACTGGTGGTGCTGGGCCATATGGTGGGGGACTTCAATCAAGCTCATTTTCAAGCGGAACTTCTTTCGGTCAAACCGCAGCT GCATCAACCAGTTTCTCTCAGACAGGAGCTACTTCTACGAGCGGTGACAGTTACACAGGCCCTGTTGGCTATAGTGGACCT------------------ CTGGGTGGAGGAGAACAG------CTC---------------------GGTTCTGCTTCCAGCCAAACAGCTAGT---------------------------

------------------------------------------------

>At_NtermV12

AGTACAGTACGATGCGGTGCAAAGTGCTTCACCTATCTCGAGGAGCCCTTGGGCCAATCCAGCTAAAGCAGGTTCGTTGATGAACTGTCTCATTAACAGAAT TGCCAGCTCCAATGTCTTGCCTCAACAAGAAAAAGAAGACTTGGAATCCATTATGGACACACTGATGTCTGCTATAAAAGGTGCGAGTGCCAAAGGTAAAAG CTCTGCAGCTCAGTTGCAGGCTATTAATATGGCAGTTGCATCTTCCCTAGCTGAATTAGTCGTTGCTGAAGACGCTGGCAATCAGGCAAGCATCGCAGTAAA GACGCAGGCCCTTTCAGGAGCTCTMCAGCAGTGYTTCCAAGCGGTCATGGGCACAGTCGACCGGAAATTCATCAACGAAATAAACGATTTGATAACAATGTT TGCCAGAGAAGCTGCAAGTGAAAGCAATGAMATTCAAGATCAAGGATCATTCTCCACAAGTGGATCATCAGCTTCATCATCGTATCAAGCAGCAAGTCAGAG TTCA------------------------------------------------------------------------ GGCATTGGGTCAGTCGGATACACTGGTGGTGCTGGGCCATATGGTGGRGGACTTCAATCAAGCTCATTTTCAGGCGGAACTTCTTTCGGTCAAACCGCAGCT GCATCAACCAGTTTCTCTCAGACAGGAGCTACTTCTACGAGCGGTGACAGTTACACAGGGCCTGTAGGCTATAGTGGACCT------------------ CTGGGTGGAGGAGCACAG------TTC---------------------GGTTCTGTTTCCAGCCAAACAGCTAGT---------------------------

------------------------------------------------

>At_NtermV13

AGTACAGTACGATGCGGTGCAAAGTGCTTCACCTATCTCGAGGAGYCCTTGGGCCAATCCAGCTAAAGCAGGTTCGTTGATGAACYGTCTCATTAACAGAAT TGCCAGCTCCRATGTCTTGCCTCAACAAGAAAAAGAAGACTTGGAATCCATTATGGACACACTGATGTCTGCTATAAAAGGTGCGAGTGCCAAAGGTAAAAG CTCTGCAGCWCAGTTGCAGGCTATTAATATGGCAGTTGCATCTTCCCTAGCTGAATTAGTCGTTGCTGAAGACGCTGGCAATCAGGCAAGCATCGCAGTAAA GACGCAGGCCCTTACAGGTGCCCTGGGGCAGTGTTTCCAAGCGGTCATGGGCACAGTCGACAGGAAATTCATCAACGAAATAAACGATTTGATAACAATGTT TGCCAGAGAAGCTGCAAGTGAAAGCAATGAAATTCAAGATCAAGGATCATTCTCCACAAGTGGATCATCAGCTTCATCATCGTATCAAGCAGCAAGTCAGAG TTCA------------------------------------------------------------------------ GGCATTGGGTCAGTCGGATACACTGGTGGTGCTGGGCCATATGGTGGAGGACTTCAATCAAGCTCATTTTCAGGCGGAACTTCTTTCGGTCAAACCGCAGCT GCATCAACCAGTTTCTCTCAGACAGGAGCTACTTCTACGAGCGGTGACAGTTACACAGGGCCTGTAGGCTATAGTGGACCT------------------ CTGGGTGGAGGAGCACAG------TTC---------------------GGTTCTGTTTCCAGCCAAACAGCTAGT---------------------------

------------------------------------------------

>At_NtermV2

AGTACAGTACGATGCGGTGCAAAGTGCTTCACCTATCTCGAGGAGCCCTTGGGCCAATCCAGCTAAAGCAGGTTCGTTGATGAACTGTCTCATTAACAGAAT TGCCAGCTCCAATGTCTTGCCTCAACAAGAAAAAGAAGACTTGGAATCCATTATGGACACACTGATGTCTGCTATAAAAGGTGCGAGTGCCAAAGGTAAAAG CTCTGCAGCTCAGTTGCAGGCTATTAATATGGCAGTTGCATCTTCCCTAGCTGAATTAGTCGTTGCTGAAGACGCTGGCAATCAGGCAAGCATCGCAGTAAA GACGCAGGCCCTTACAGGTGCCCTGGGGCAGTGTTTCCAAGCGGTCATGGGCACAGTCGACAGGAAATTCATCAACGAAATAAACGATTTGATAACAATGTT TGCCAGAGAAGCTGCAAGTGACAGCAATGAAATTGAAGACCAAGGATCATTCACCACAAGTGGATCATCAGCTTCATCGTCGTATCAAGCAGCAAGTCAGAG TTTA------------------------------------------------------------------------ GGCATTGGGTCAGTCGGTTACACTGGTGGTGCTGGGCCATATGGGGGAGGACTTCAGTCAAGCTCATTTTCAGGCGGAACTTCTTTCGGTCAAACCGCAGCT GCATCAACCAGTTTCTCTCAGACAGGAGCTACTTCTACGAGCGGGGACAGTTAT------CCTGGTAGCTATATTGGAACT------------------ CTGGGTGGAGGAGCACAG------TTC---------------------GGTTCTGCTTCCAGCCAAACAGCTAGT---------------------------

------------------------------------------------

>At_NtermV3

AGTACAGTACGATGCGGTGCAAAGTGCTTCACCTATCTCGAGGAGTCCTTGGGCCAATCCAGCTAAAGCAGGTTCGTTGATGAACTGTCTCATTAACAGAAT TGCCAGCTCCAATGTCTTGCCTCAACAAGRARWAGAAGACTTGGAATCCATTATGGACACACTGATGTCTGCTATAAAAGGTGCGAGTGCCAAAGGTAAAAG CTCTGCAGCWCAGTTGCAGGCTATYAATATGGCAGTTGCATCTTCCCTAGCTGAATTAGTCGTTGCTGAAGACGCTGGCAATCAGGCAAGCATCGCAGTAAA GACGCAGGCCCTTACAGGTGCCCTGGGGCAGTGTTTCCAAGCGGTCATGGGCACAGTCGACAGGAAATTCATCRACGAAATAAACGATTTGATAACAATGTT TGCCAGAGAAGCTGCAAGTGACAGCAATGAAATTGAAGACCAAGGATCATTCACCACAAGTGGATCATCAGCTTCATCGTCGTATCAAGCAGCAAGTCAGAG TTTA------------------------------------------------------------------------ GGCATTGGGTCAGTCGGTTACACTGGTGGTGCTGGGCCATATGGGGGAGGACTTCAGTCAAGCTCATTTTCAGGCGGAACTTCTTTCGGTCAAACCGCAGCT GCATCAACCAGTTTCTCTCAGACAGGAGCTACTTCTACGAGCGGGGACAGTTAT------CCTGGTAGCTATRTTGGAACT------------------ CTGGGTGGAGGAGCACAG------TTC---------------------GGTTCTGCTTCCAGCCAAACAGCTAGT---------------------------

------------------------------------------------

>At_NtermV4

AGTACAGTACGATGCGGTGCAAAGTGCTTCACCTATCTCGAGGAGTCCTTGGGCCAATCCAGCTAAAGCAGGTTCGTTGATGAACTGTCTCATTAACAGAAT TGCCAGCTCCAATGTCTTGCCTCAACAAGAAAAAGAAGACTTGGAATCCATTATGGACACACTGATGTCTGCTATAAAAGGTGCGAGTGCCAAAGGTAAAAG CTCTGCAGCTCAGTTGCAGGCTATTAATATGGCAGTTGCATCTTCCCTAGCTGAATTAGTCGTTGCTGAAGACGCTGGCAATCAGGCAAGCATCGCAGTAAA GACGCAGGCCCTTACAGGTGCCCTGGGGCAGTGTTTCCAAGCGGTCATGGGCACAGTCGACAGGAAATTCATCAACGAAATAAACGATTTGATAACAATGTT TGCCAGAGAAGCTGCAAGTGACAGCAATGAAATTGAAGACCAAGGATCATTCTCCACAAGTGGATCATCAACTTCATCGTCGTATCAAGCAGCAAGTCAGAG TTTA------------------------------------------------------------------------ GGCATTGGGTCAGTCGGTTACACTGGTGGTGCTGGGCCATATGGGGGAGGACTTCAGTCAAGCTCATTTTCAGGCGGAACTTCTTTCGGTCAAACCGCAGCT GCATCAACCAGTTTCTCTCAGACAGGAGCTACTTCTACGAGCGGGGACAGTTAT------CCTGGTAGCTATATTGGAACT------------------ CTGGGTGGAGGAGCACAG------TTC---------------------GGTTCTGCTTCCAGCCAAACAGCTAGT---------------------------

------------------------------------------------

>At_NtermV5

AGTACAGTACGATGCGGTGCAAAGTGCTTCGACTATCTCGAGGAGTCCTTGGGCCAATCCAGCTAAAGCAGGTTCGTTGATGAACTGTCTCATTAACAGAAT TGCCAGCTCCAATGTCTTGCCTCAACAAGAAAAAGAAGACTTGGAATCCATTATGGACACACTGATGTCTGCTATAAAAGGTGCGAGTGCCAAAGGTAAAAG CTCTGCAGCTCAGTTGCAGGCTATTAATATGGCAGTTGCATCTTCCCTAGCTGAATTAGTCGTTGCTGAAGACGCTGGCAATCAGGCAAGCATCGCAGTAAA GACGCAGGCCCTTACAGGTGCCCTGGGGCAGTGTTTCCAAGCGGTCATGGGCACAGTCGACAGGAAATTCATCAACGAAATAAACGATTTGATAACAATGTT TGCCAGAGAAGCTGCAAGTGACAGCAATGAAATTGAAGACCAAGGATCATTCTCCACAAGTGGATCATCAACTTCATCGTCGTATCAAGCAGCAAGTCAGAG TTTA------------------------------------------------------------------------ GGCATTGGGTCAGTCGGTTACACTGGTGGTGCTGGGCCATATGGGGGAGGACTTCAGTCAAGCTCATTTTCAGGCGGAACTTCTTTCGGTCAAACCGCAGCT GCATCAACCAGTTTCTCTCAGACAGGAGCTACTTCTACGAGCGGGGACAGTTAT------CCTGGTAGCTATATTGGAACT------------------ CTGGGTGGAGGAGCACAG------TTC---------------------GGTTCTGCTTCCAGCCAAACAGCTAGT---------------------------

------------------------------------------------

>At_NtermV1

AGTACAGTACGATGCGGTGCAAAGTGCTTCACCTATCTCGAGGAGCCCTTGGGCCAATCCAGCTAAAGCAGGTTCGTTGATGAACTGTCTCATTAACAGAAT TGCCAGCTCCAATGTCTTGCCTCAACAAGAAAAAGAAGACTTGGAATCCATTATGGACACACTGATGTCTGCTATAAAAGGTGCGAGTGCCAAAGGTAAAAG CTCTGCAGCTCAGTTGCAGGCTATTAATATGGCAGTTGCATCTTCCCTAGCTGAATTAGTCGTTGCTGAAGACGCTGGCAATCAGGCAAGCATCGCAGTAAA GACGCAGGCCCTTACAGGTGCCCTGGGGCAGTGTTTCCAAGCGGTCATGGGCACAGTCGACAGGAAATTCATCAACGAAATAAACGATTTGATAACAATGTT TGCCAGAGAAGCTGCAAGTGACAGCAATGAAATTGAAGACCAAGGATCATTCTCCACAAGTGGATCATCAACTTCATCGTCGTATCAAGCAGCAAGTCAGAG TTTA------------------------------------------------------------------------ GGCATTGGGTCAGTCGGTTACACTGGTGGTGCTGGGCCATATGGGGGAGGACTTCAGTCAAGCTCATTTTCAGGCGGAACTTCTTTCGGTCAAACCGCAGCT GCATCAACCAGTTTCTCTCAGACAGGAGCTACTTCTACGAGCGGGGACAGTTAT------CCTGGTAGCTATATTGGAACT------------------ CTGGGTGGAGGAGCACAG------TTC---------------------GGTTCTGCTTCCAGCCAAACAGCTAGT---------------------------

------------------------------------------------

>At_NtermV7

AGTACAGTACGATGCGGTGCAAAGTGCTTCACCTATCTCGAGGAGYCCTTGGGCCAATCCAGCTAAAGCAGGTTCGTTGATGAACTGTCTCATTAACAGAAT TGCCAGCTCCAATGTCTTGCCTCAACAAGAAAAAGAAGACTTGGAATCCATTATGGACACACTGATGTCTGCTATAAAAGGTGCGAGTGCCAAAGGTAAAAG CTCTGCAGCACAGTTGCAGGCTATTAATATGGCAGTTGCATCTTCCCTGGCAGAACTAGTCGTTGCTGAAGATGCAGGCAATCAGGCAAGCATCGCAGTAAA GACGCAGGCCCTTTCAGGAGCTCTACAGCAGTGTTTCCAAGCGGTCATGGGCACAGTCGACCGGAAATTCATCAACGAAATAAACGATTTGATAACAATGTT TGCCAGAGAAGCTGCAAGTGAMAGCAATGAAATTSAAGAYCAAGGATCATTCTCCACAAGTGGATCATCARCTTCATCGTCGTATCAAGCAGCAAGTCAGAG TTTA------------------------------------------------------------------------ GGCATTGGGTCAGTCGGTTACACTGGTGGTGCTGGGCCATATGGGGGAGGACTTCAGTCAAGCTCATTTTCAGGCGGAACTTCTTTCGGTCAAACCGCAGCT GCATCAACCAGTTTCTCTCAGACAGGAGCTACTTCTACGAGCGGGGACAGTTAT------CCTGGTAGCTATATTGGAACT------------------ CTGGGTGGAGGAGCACAG------TTC---------------------GGTTCTGCTTCCAGCCAAACAGCTAGT---------------------------

------------------------------------------------

**Supplementary Figure S4. Nucleotide alignment of *AcSp1* N-terminal coding variants from PCR survey of individual *Argiope***

**genomes.** Nucleotide alignment in FASTA format. Alignment available on TreeBASE ([http://purl.org/phylo/treebase/phylows/study/TB2:S15355](http://purl.org/phylo/treebase/phylows/study/TB2%3AS15355))). Abbreviations: *A. argentata (*Aarg), *A. aurantia* (Aau), and *A. trifasciata*

(At).

>Av_Cterm

TCCCAAGTACTCTCAGCAGTTTTGAACGGAGTATCAAGTGCGGCGCAAGGCCTTGGAATCAATGTAGACACTGGAAGTGTACAAAGTGACATTCGTTC CAGCAGTAGCTCCCTGTCAACAAGTTCTTCGTCTGCCAGTTTC---TCTCAGACCAGTGGTGCAGCTTCGACA---ACTGGTTTCACA--------- GGGGCTGGTGGCTAC-----------------------------------------------------------------------------------

----------------CCTGGTGGAGCTGGTCCTTTGGGTGGCGGAGTAGGCTCATTTGGAGGT------

CAAACCTCTTTCGGTCAAACATCAGGCTTGACCTCTTCTGCTGCTAGCCAATCGGATTTCACTCAAGCTAGTGGCTTTGTGTCATCTGCTACCAGTCA AGGTGCTTTTGGTCAAACGTCGGGTATTGCTTCATTTGGTGCTGGACCATCGACTGGATTATCGGTGAGATCTACTCTTAATTCGCCCAATGGATTGA GGTCGGGTTCGGCTGCAGCTAGAATCAGCCAATTGACATCATCTGTAAGGAATGCGATCGGTCCCAATGGCGTTGATGCTAATGCTCTGGCCCGTAGT CTTCAAGCAAGTTTCTCGAGTCTTCGAAGT

>Aar_CtermV2

TCCCGAGTCCTGTCAGGAGTTTTGAACGGAGTATCAAGAGCTGCACAAGACCTTGGCTTGAATGTAGACACCAGCAATGTACAAAATGACATTAGTTC CAGCAATAGCTTCCTCTCATCCAGTTCCACC---ACCAGTTAC---TCTCAGACAGGAGGTTCT--- ACTTCCAGTGCTGCTTCAACTCGCACCTCAGGATCTACAGGCTACACCGGAGAACAG------------ TACGGATCTGCATCCAGCCAAAGATCCTACGGTCAAACA------------------------------------TCAGGCTATACCGGATCT--- ACTGCCGGA------------------------ CAAACCGGTTACGGACAAGCATCTGGCTACACCGGATCTTCTGAAAGCCAAAGAGGTTATGGTCAATCAAGTGGTTCGACATCTCAACTTGCCTTT--

------------------------------------- GGTGCTGGACAATCTGCTGGAGCTTCCCTGACAGGTGCTCTTAACTCACCTGTTGGATTGAGATCTGGTTCTGCTGCTTCTAGAATTAATCAATTAGC ATCATCTGTGACAAATGCGGTCGGCCCCAACGGCGTGGATGCTAATGCTCTTGCTCAAAGTCTCCAATCTAGTTTCTCGGCACTCAGAAGC

>Aar_CtermV1

TCCCGAGTTCTCTCAGCTGTTTTGAACGGAGTATCAAGTGCGGCGCAAGGTCTTGGCATCAATGTAGATACCGGCAGTGTACAGAGTGACATTAGTTC CAGTAGCAGCTTCCTCTCAACCAGCTCGTCTGCGTCCAGTATCTCCTCTCAGGTATCAGCTTCTTCTACCAGCGGTGCTGGCTACACC--------- GGACCTTCTGGCTAC-----------------------------------------------------------------------------------

------------------------- ACAGGACCAGTGGGTGGCGGAGCGCAGTTCGGTTCTTCTTCAGGCCAAAGCTCTTTCGGTCAAACATCAGGATTTACAACTTCCGCTGGC--------

-CTCTTAGGAGCCACGTCTGGA------TCG---CTG---TCT--------------------------------------- GGTGCTGGACAATCTGCTGGAGTATCTTTAACGTCTTCTCTTAATTCGCCCGTTGGATTAAGATCTGGTTCTGCTGCTTCTAGAATCAGGCAATTAAC TTCATCTGTGACGAATGCGGTCGGCCCCAATGGAGTTGATGCTAATGCACTTGCCCGTAGTCTTCAATCTAGTTTCTCGAATCTGAGAAGT

>Aar_CtermV6

TCCCGAGTTCTGTCAGCAGTTTTGAACGGAGTATCAAGTGCGGCGCAAGGTCTAGGCATCAATGTAGACACTGGCAGTGTACAAAGTGACATTCGTTC CAGCAGTGGCTTCCTGTCAACAAGCACTTCGTCGACCAGTTTC---TCTCAGACATCAGCTTCTTCTGCCGGTGGAGCTGGCTACTCG--------- GGACCTTCTGGCTACAAT--------------------------------------------------------------- GGATCTTCCGGGTACACC--------- GGACCGTCTGGCTACACTGGGCCTGTGGGCGGCGGAGCGCAGTTCGGTTTCGTTTCAGGCCAAACCTCTTACGGTCAAACGTCAGGATCTGCCGCATC TACTGGAGGCCAAGGTGTTTTAGGTCAACCAATTGGGGTACCATCTAGCCTCACCGCT--------------------------------------- GGTGTTGGACAAACGGCTGATGTATCAGTAATTTCTTCTCTTAACTCACCTGTTGGATTGAGATCTGGTTCTGCTGCTTCTAGAATTAATCAACTAGC ATCATCTGTGACAAATGCGGTCGGACCCAACGGCGTGGATGCTAATGTTCTTGCTCAAAGTCTCCAATCTAGTTTCTCGGCACTCAGAAGC

>Aar_CtermV5

TCCCGAGTTCTGTCAGCAGTTTTGAACGGAGTATCAAGTGCGGCGCAAGGTCTAGGCATCAATGTAGACACTGGCAGTGTACAAAGTGACATTCGTTC CAGCAGTGGCTTCCTGTCAACAAGCACTTCGTCGACCAGTTTC---TCTCAGACATCAGCTTCTTCTGCCGGTGGAGCTGGCTACTCG--------- GGACCTTCTGGCTACAAT--------------------------------------------------------------- GGATCTTCCGGGTACACC--------- GGACCGTCTGGCTACACTGGGCCTGTGGGCGGCGGAGCGCAGTTCGGTTTCGTTTCAGGCCAAACCTCTTACGGTCAAACGTCAGGATCTGCCGCATC TACTGGAGGCCAAGGTGTTTTAGGTCAACCAATTGGGGTACCATCTAGCCTCACCGCT--------------------------------------- GGTGTTGGACTAACGGCTGATGTATCAGTAATTTCTTCTCTTAACTCACCTGTTGGATTGAGATCTGGTTCTGCTGCTTCTAGAATTAATCAACTAGC ATCATCTGTGACAAATGCGGTCGGACCCAACGGCGTGGATGCTAATGTTCTTGCTCAAAGTCTCCAATCTAGTTTCTCGGCACTCAGAAGC

>Aar_CtermV4

TCCCGAGTTCTGTCAGCAGTTTTGAACGGAGTATCAAGTGCGGCGCAAGGTCTAGGCATCAATGTAGACACTGGCAGTGTACAAAGTGACATTCGTTC CAGCAGTGGCTTCCTGTCAACAAGCACTTCGTCGACCAGTTTC---TCTCAGGCATCAGCTTCTTCTGCCGGTGGAGCTGGCTACTCG--------- GGACCTTCTGGCTACAAT--------------------------------------------------------------- GGATCTTCCGGGTACACC--------- GGACCGTCTGGCTACACTGGGCCTGTGGGCGGCGGAGCGCAGTTCGGTTTCGTTTCAGGCCAAACCTCTTACGGTCAAACGTCAGGATCTGCCGCATC TACTGGAGGCCAAGGTGTTTTAGGTCAACCAATTGGGGTACCATCTAGCCTCACCGCT--------------------------------------- GGTGTTGGACAAACGGCTGATGTATCAGTAATTTCTTCTCTTAACTCACCTGTTGGATTGAGATCTGGTTCTGCTGCTTCTAGAATTAATCAACTAGC ATCATCTGTGACAAATGCGGTCGGACCCAACGGCGTGGATGCTAATGTTCTTGCTCAAAGTCTCCAATCTAGTTTCTCGGCACTCAGAAGC

>Aar_CtermV3

TCCCGAGTTCTGTCAGCAGTTTTGAACGGAGTATCAAGTGCGGCGCAAGGTCTAGGCATCAATGTAGACACTGGCAGTGTACAAAGTGACATTCGTTC CAGCAGTGGCTTCCTGTCAACAAGCACTTCGTCGACCAGTTTC---TCTCAGGCATCAGCTTCTTCTGCCGGTGGAGCTGGCTACTCG--------- GGACCTTCTGGCTACAAT--------------------------------------------------------------- GGACCTTCCGGGTACACC--------- GGACCTTCTGGCTACACTGGGCCTGTGGGCGGCGGAGCGCAGTTCGGTTCCGTTTCAGGCCAAACCTCTTACGGTCAAACGTCAGGATCTGCCGCATC TACTGGAGGCCAAGGTGTTTTAGGTCAACCAATTGGGGTACCATCTAGCCTCACCGCT--------------------------------------- GGTGTTGGACAAACGGCTGATGTATCAGTAATTTCTTCTCTTAACTCACCTGTTGGATTGAGATCTGGTTCTGCTGCTTCTAGAATTAATCAACTAGC ATCATCTGTGACAAATGCGGTCGGACCCAACGGCGTGGATGCTAATGTTCTTGCTCAAAGTCTCCAATCTAGTTTCTCGGCACTCAGAAGC

>Aar_CtermV7

TCCCGAGTTCTGTCAGCAGTTTTGAACGGAGTATCAAGTGCGGCGCAAGGTCTAGGCATCAATGTAGACACTGGCAGTGTACAAAGTGACATTCGTTC CAGCAGTGGCTTCCTGTCAACAAGCACTTCGTCGACCAGTTTC---TCTCAGGCATCAGCTTCTTCTGCCGGTGGAGCTGGCTACTCG--------- GGACCTTCTGGCTACAAT--------------------------------------------------------------- GGACCTTCCGGGTACACC--------- GGACCTTCTGGCTACACTGGGCCTGTGGGCGGCGGAGCGCAGTTCGGTTCCGTTTCAGGCCAAACCTCTTACGGTCAAACGTCAGGATCTGCCGCATC TACTGGAGGCCAAGGTGTTTTAGGTCAACCAATTGGGGTACCATCTAGCCTCACCGCT--------------------------------------- GGTGTTGGACTAACGGCTGATGTATCAGTAATTTCTTCTCTTAACTCACCTGTTGGATTGAGATCTGGTTCTGCTGCTTCTAGAATTAATCAACTAGC ATCATCTGTGACAAATGCGGTCGGACCCAACGGCGTGGATGCTAATGTTCTTGCTCAAAGTCTCCAATCTAGTTTCTCGGCACTCAGAAGC

>Aau_CtermV1

TCCCGAGTTCTGTCAGCAGTTTTGAACGGAGTATCAAGTGCGGCGCAAGGTGTTGGCATCAATGTAGATTCCGGCAGTGTACAAAGTGACATTAGTTC CAGTAGCAGCTTCCTCTCAACAAGCTCGTCTGCGTCCAGTTTC--- TCTCAGACATCAGCTTCTTCTACCAGCGGTGCTGGTTACACAGGGCCTTCTGGACCTTCTGGCTACACAGGGCCTGCT--------------------

----------------------------------

GGACCTTCTGGCTACACAGGGCCTGCTGGACCTTCTGGTTACACTGGGCCTGTGGGTGGCGGAGCGCAGTTCGGTTCTGTTTCAGGCCAATCCTCTTT CGGTCAAACATTAGGTGTTACGGCTTCTGCTGGCGCTCCAGGTGGTTTG------CTGGGTGGCGCCCCATCTAGCGTGACCTCT-------------

-------------------------- GGTGCCGGACAGTCGGGTGGAGTATCTCTCATATCTTCTCTTAATTCACCCGTTGGATTGAGATCTGGTTCTGCTGCTTCTAGAATTAGTCAATTAAC ATCCTCCGTAACAAATGCGGTCGGCCCCAATGGTGTTGATGCTAACGCTCTTGCCCGAAGTCTTCAATCTAGTTTCTCGGCACTGAGAAGC

>Aau_CtermV2

TCCCGAGTTCTGTCAGCAGTTTTGAACGGAGTATCAAGTGCGGCGCAAGGTGTTGGCATCAATGTAGATACCGGCAGTGTACAAAGTGACATTAGTTC CAGTAGCAGCTTCCTCTCAACAAGCTCGTCTGCGGCCAGTTTC--- TCTCAGACATCAGCTTCTTCTACCAGCGGTGCTGGTTACACAGGGCCTGCTGGACCTTCTGGCTACACAGGGCCTGCTGGACCTTCAGTCTACACAGG GCCTGCTGGACCTTCAGGCTACACAGGGCCTGCTGGACCTTCAGGCTACACAGGGCCCTCTGGACCGTCTGGCTACACTGGGCCTGTAGGTGGCGGA-

--------GGCGTCGTTTCAGGCCAAACCTCTTTCGGTCAAACTACAGGTTTTAAGGCGTCTGCTGGCGCTGAAGGAGTATTC------ CCTGGTGGCGTCCCATCTAGTCTGACCTCT--------------------------------------- GGTGCCGGACAGTCGGCTGGAGTATCTCTCATATCTTCTCTTAATTCTCCCGTTGGATTGAGATCTGGTTCTGCTGCTTCTAGAATTAGTCAATTAAC ATCCTCCGTAACAAATGCTGTCGGACCCAATGGTGTTGATGCTAATGCTCTTGCCCGAAGTCTTCAATCTAGTTTCTCGGCACTCAGAAGC

>At_CtermV6

TCCCGAGTTCTCTCAGCACTTTTGAACGGAGTATCAAGTGCGGCGCAAGGTCTTGGCATCAATGTAGATAGCGGCAGTGTACAAAGTGACATTAGTTC CAGTAGCAGCTTCCTCTCAACAAGCTCGTCTTCGGCCAGTTAC---TCTCAGGCATCAGCTTCTTCGACCAGCGGTGCCGGATACACA--------- GGACCTTCT------------------------------------------------------------------------GGACCTTCC------ ACT---------GGACCGTCTGGCTACCCTGGGCCTTTGAGTGGCGGAGCGTCGTTCGGC------ TCTGGCCAAACTTCTTTCGGTCAAACATCAGCCTTTGCCGCATCT-----------------------------------------------------

------------------------------------------- GGTGCTGGACAATCGGCTGGAGTATCTGTTATATCTTCTCTTAATTCACCCGTTGGATTGAGGTCTGCTTCTGCTGCTTCTAGACTTAGTCAATTAAC ATCATCCATAACGAATGCAGTTGGTGCCAATGGCGTTGATGCTAATTCTCTTGCCCGTAGTCTTCAATCTAGTTTCTCGAT

>At_CtermV7

TCCCGAGTTCTCTCAGCACTTTTGAACGGAGTATCAAGTGCGGCGCAAGGTCTTGGCATCAATGTAGATAGCGGCAGTGTACAAAGTGACATTAGTTC CAGTAGCAGCTTCCTCTCAACAAGCTCGTCTTCGGCCAGTTAC---TCTCAGGCATCAGCTTCTTCGASCAGCGGTGCCGGATACACA--------- GGACCTTCT------------------------------------------------------------------------GGACCTTCC------ ACT---------GGACCKTCTGGCTACCCTGGGCCTTTGAGTGGCGGAGCGTCGTTCGGC------ TCTGGCCAAACTTCTTTCGGTCAAACATCAGCCTTTGCCGCATCT-----------------------------------------------------

-------------------------------------------

GGTGCTGGACAATCGGCTGGAGTATCTGTTATATCTTCTCTTAATTCACCCGTTGGATTGAGGTCTGCTTCTGCTGCTTCTAGACTTAGTCAATTAAC ATCATCCATAACGAATGCAGTTGGTGCCAATGGTGTTGATGCTAATTCTCTTGCCCGTAGTCTTCAATCTAGTTTCTCGGCACTCAGAAGC

>At_CtermPubMed

TCCCGAGTTCTCTCAGCACTTTTGAACGGAGTATCAAGTGCGGCGCAAGGTCTTGGCATCAATGTAGATAGCGGCAGTGTACAAAGTGACATTAGTTC CAGTAGCAGCTTCCTCTCAACAAGCTCGTCTTCGGCCAGTTAC---TCTCAGGCATCAGCTTCTTCGACCAGCGGTGCCGGATACACA--------- GGACCTTCT------------------------------------------------------------------------GGACCTTCC------ ACT---------GGACCTTCTGGCTACCCTGGGCCTTTGAGTGGCGGAGCGTCGTTCGGC------ TCTGGCCAATCTTCTTTCGGTCAAACTTCAGCCTTTTCCGCATCT-----------------------------------------------------

------------------------------------------- GGTGCTGGACAATCGGCTGGAGTATCTGTTATATCTTCTCTTAATTCACCCGTTGGATTGAGGTCTGCTTCTGCTGCTTCTAGACTTAGTCAATTAAC ATCATCCATAACGAATGCAGTTGGTGCCAATGGTGTTGATGCTAATTCTCTTGCCCGTAGTCTTCAATCTAGTTTCTCGGCACTCAGAAGC

>At_CtermV8

TCCCGAGTTCTCTCAGCACTTTTGAACGGAGTATCAAGTGCGGCGCAAGGTCTTGGCATCAATGTAGATAGCGGCAGTGTACAAAGTGACATTAGTTC CAGTAGCAGCTTCCTCTCAACAAGCTCGTCTTCGGCCAGTTAC---TCTCAGGCATCAGCTTCTTCGAGCAGCGGTGCCGGATACACA--------- GGACCTTCT------------------------------------------------------------------------GGACCTTCC------ ACT---------GGACCTTCTGGCTACCCTGGGCCTTTGAGTGGCGGAGCGTCGTTCGGC------ TCTGGCCAATCTTCTTTCGGTCAAACTTCAGCCTTTTCCGCATCT-----------------------------------------------------

------------------------------------------- GGTGCTGGACAATCGGCTGGAGTATCTGTTATATCTTCTCTTAATTCACCCGTTGGATTGAGGTCTGCTTCTGCTGCTTCTAGACTTAGTCAATTAAC ATCATCCATAACGAATGCAGTTGGTGCCAATGGTGTTGATGCTAATTCTCTTGCCCGTAGTCTTCAATCTAGTTTCTCGGCACTCAGAAGC

>At_CtermV9

TCCCGAGTTCTCTCAGCACTTTTGAACGGAGTATCAAGTGCGGCGCAAGGTCTTGGCATCAATGTAGATAGCGGCAGTGTACAAAGTGACATTAGTTC CAGTAGCAGCTTCCTCTCAACAAGCTCGTCTTCGGCCAGTTAC---TCTCAGGCATCAGCTTCTTCGACCAGCGGTGCCGGATACACA--------- GGACCTTCT------------------------------------------------------------------------GGACCTTCC------ ACT---------GGACCGTCTGGCTACCCTGGGCCTTTGAGTGGCGGAGCGTCGTTCGGC------ TCTGGCCAATCTTCTTTCGGTCAAACGTCAGCCTTTTCCGCATCT-----------------------------------------------------

-------------------------------------------

GGTGCTGGACAATCGGCTGGAGTATCTGTTATATCTTCTCTTAATTCACCCGTTGGATTGAGGTCTCCTTCTGCTGCTTCTAGACTTAGTCAATTAAC ATCATCCATAACGAATGCAGTTGGTGCCAATGGTGTTGATGCTAATTCTCTTGCCCGTAGTCTTCAATCTAGTTTCTCGGCACTYAGAAGC

>At_CtermV5

TCCCGAGTTCTCTCAGCACTTTTGAACGGAGTATCAAGTGCGGCGCAAGGTCTTGGCATCAATGTAGATAGCGGCAGTGTACAAAGTGACATTAGTTC CAGTAGCAGCTTCCTCTCAACAAGCTCGYCTGCGGCCAGTTAC---TCTCAGACATCAGCTTCTTCGACCAGCGGTGCCGGATACACA--------- GGACCTTCT------------------------------------------------------------------------GGACCTTCC--------

-------------------RGCTACCCTGGGCCTTTGAGTGGCGGAGCGTCGTTCGGC------

TCTGGCCAAACTTCTTTCGGTCAAACATCAGCCTTTGCCGCATCT-----------------------------------------------------

------------------------------------------- GGTGCTGGACAATCGGCTGGAGTATCTGTTATATCTTCTCTTAATTCACCCGTTGGATTGAGGTCTGCTTCTGCTGCTTCTAGACTTAGTCAATTAAC ATCATCCATAACGAATGCAGTTGGTGCCAATGGCGTTGATGCTAATTCTCTTGCCCGTAGTCTTCAATCTAGTTTCTCGATCTT

>At_CtermV4

TCCCGAGTTCTCTCAGCACTTTTGAACGGAGTATCAAGTGCGGCGCAAGGTCTTGGCATCAATGTAGATAGCGGCAGTGTACAAAGTGACATTAGTTC CAGTAGCAGCTTCCTCTCAACAAGCTCGTCTGCGGCCAGTTAC---TCTCAGACATCAGCTTCTTCGACCAGCGGTGCCGGATACACA--------- GGACCTTCT------------------------------------------------------------------------GGACCTTCC--------

-------------------GGCTACCCTGGGCCTTTGAGTGGCGGAGCGTCGTTCGGC------ TCTGGCCAAACTTCTTTCGGTCAAACATCAGCCTTTGCCGCATCT-----------------------------------------------------

------------------------------------------- GGTGCTGGACAATCGGCTGGAGTATCTGTTATATCTTCTCTTAATTCACCCGTTGGATTGAGGTCTGCTTCTGCTGCTTCTAGACTTAGTCAATTAAC ATCATCCATAACGAATGCAGTTGGTGCCAATGGCGTTGATGCTAATTCTCTTGCCCGTAGTCTTCAATCTAGTTTCTCGGCACTTAGAAGC

>At_CtermV3

TCCCGAGTTCTGTCAGCAGTTTTGAACGGAGTATCAAGTGCGGCGCAAGGTCTTGGCATCAATGTAGATACCGGCAGTGTACAAAGTGACATTAGTTC CAGTAGCAGCTTCCTCTCAACAAGCTCGTCTGCGGCCAGTTAC---TCTCAGACATCAGCTTCTTCGACCAGCGGTGCCGGATACACA--------- GGACCTTCT------------------------------------------------------------------------GGACCTTCC--------

-------------------GGCTACCCTGGGCCTTTGAGTGGCGGAGCGTCGTTCGGC------

TCTGGCCAAACTTCTTTCGGTCAAACATCAGCCTTTGCCGCATCT-----------------------------------------------------

------------------------------------------- GGTGCTGGACAATCGGCTGGAGTATCTGTTATATCTTCTCTTAATTCACCCGTTGGATTGAGGTCTGCTTCTGCTGCTTCTAGACTTAGTCAATTAAC ATCATCCATAACGAATGCAGTTGGTGCCAATGGCGTTGATGCTAATTCTCTTGCCCGTAGTCTTCAATCTAGTTTCTCGGCACTCAGAAGC

>At_CtermV1

TCCCGAGTTCTGTCAGCAGTTTTGAACGGAGTATCAAGTGCGGCGCAAGGTCTTGGCATCAATGTAGATACCGGCAGTGTACAAAGTGACATTAGTTC CAGTAGCAGCTTCCTCTCAACAAGCTCGTCTGCGGCCAGTTAC---TCTCAGACATCAGCTTCTTCGACCAGCGGTGCCGGATACACA--------- GGACCTTCT------------------------------------------------------------------------GGACCTTCC--------

-------------------GGCTACCCTGGGCCTTTGAGTGGCGGAGCGTCGTTCGGC------

TCTGGCCAAACTTCTTTCGGTCAAACATCAGCCTTTGCCGCATCT-----------------------------------------------------

------------------------------------------- GGTGCTGGACAATCGGCTGGAGTATCTGTTATATCTTCTCTTAATTCACCCGTTGGATTGAGGTCTGCTTCTGCTGCTTCTAGACTTAGTCAATTAAC ATCATCCATAACGAATGCAGTTGGTGCCAATGGCGTTGATGCTAATTCTCTTGCCCGTAGTCTTCAATCTAGTTTCTCGGCACTTAGAAGC

>At_CtermV2

TCCCGAGTTCTGTCAGCAGTTTTGAACGGAGTATCAAGTGCGGCGCAAGGTCTTGGCATCAATGTAGATACCGGCAGTGTACAAAGTGACATTAGTTC CAGTAGCAGCTTCCTCTCAACAAGCTCGTCTGCGGCCAGTTAC---TCTCAGACATCAGCTTCTTCGACCAGCGGTGCCGGATACACA--------- GGACCTTCT------------------------------------------------------------------------GGACCTTCC--------

-------------------GGCTACCCTGGGCCTTTGAGTGGCGGAGCGTCGTTCGGC------ TCTGGCCAAACTTCTTTCGGTCAAACATCAGCCTTTGCCGCATCT-----------------------------------------------------

------------------------------------------- GGTGCTGGACAATCGGCTGGAGTATCTGTTATATCTTCTCTTAATTCACCCGTTGGATTGAGGTCTGCTTCTGCTGCTTCTAGACTTAGTCAATTAAC ATCATCCATAACGAATGCAGTTGGTGCCAATGGTGTTGATGCTAATTCTCTTGCCCGTAGTCTTCAATCTAGTTTCTCGGCACTCAGAAGC

>At_CtermV10

TCCCGAGTTCTCTCAGCACTTTTGAACGGAGTATCAAGTGCGGCGCAAGGTCTTGGCATCAATGTAGATAGCGGCAGTGTACAAAGTGACATTAGTTC CAGTAGCAGCTTCCTCTCAACAAGCTCGTCTTCGGCCAGTTAC---TCTCAGGCATCAGCTTCTTCGACCAGCGGTGCCGGATACACA--------- GGACCTTCT-----------------------------------------------------------------------------------------

-------------------GGCTACCCTGGGCCTTTGAGTAGCGGAGTGTCGTTCGGC------

TCAGGGCAATCTTCTTTCGGTCAAACATCAGCTCTTTCCGCATCT-----------------------------------------------------

------------------------------------------- GGTGCTGGACAATCGGCTGGAGTATCTGTTATATCTTCTCTTAATTCACCTGTTGGATTGAGGTCTGCTTCTGCTGCTTCTAGACTTAGTCAATTAAC ATCATCCATAACGAATGCAGTTGGTGCCAATGGTGTTGATGCTAATTCTCTTGCCCGTAGTCTTCAATCTAGTTTCTCGGCACTCAGAAGC

>At_CtermV11

TCCCGAGTTCTCTCAGCACTTTTGAACGGAGTATCAAGTGCGGCGCAAGGTCTTGGCATCAATGTAGATAGCGGCAGTGTACAAAGTGACATTAGTTC CAGTAGCAGCTTCCTCTCAACAAGCTCGTCTTCGGCCAGTTAC---TCTCAGGCATCAGCTTCTTCGACCAGCGGTGCCGGATACACA--------- GGACCTTCT-----------------------------------------------------------------------------------------

-------------------GGCTACCCTGGGCCTTTGAGTAGCGGAGCGTCGTTCGGC------

TCAGGGCAATCTTCTTTCGGTCAAACATCAGCCCTTTCCGCATCT-----------------------------------------------------

------------------------------------------- GGTGCTGGACAATCGGCTGGAGTATCTGTTATATCTTCTCTTAATTCACCCGTTGGATTGAGGTCTGCTTCTGCTGCTTCTAGACTTAGTCAATTAAC ATCATCCATAACGAATGCAGTTGGTGCCAATGGTGTTGATGCTAATTCTCTTGCCCGTAGTCTTCAATCTAGTTTCTCGRCACTCAGAAGC

**Supplementary Figure S5. Nucleotide alignment of *AcSp1* C-terminal coding variants from PCR survey of individual *Argiope* genomes.** Nucleotide alignment in FASTA format. Alignment available on TreeBASE ([http://purl.org/phylo/treebase/phylows/study/TB2:S15355](http://purl.org/phylo/treebase/phylows/study/TB2%3AS15355))). Abbreviations: *A. argentata (*Aarg), *A. aurantia* (Aau), *A. trifasciata* (At),

and *Araneus ventricous* (Av).

>Aarg

GGCTACACCGGACCTTCTGGCTACACAGGA--------- CCAGTGGGTGGCGGAGCGCAGTTCGGTTCTGCTTCAGGCCAAAGCTCTTTCGGT------------ CAGACATCAGGATTTACCGCATCTTCTGGCGGACAAGCCGCTTTCGGTGGAACCAGTGGAGCGTCTGCTGGCCTCATCTCCAGAGTAGC AAACGCACTTGCCAATACATCCACATTGAGAGCTGTCCTCCGAAGAGGTGTATCCCAACAGACTGCCTCCAGCGTGGTACAGAGAGCCG CTCAGTCGTTGGCCAGTACTCTCGGAGTCGACGGAAACAACTTGTCCAGAGTAGCATTACAAGCCATCTCTCAAGTGCCCACAGGGTCT GACACTTCAGCTTACGCTCAAGCCTTCTCTAGTGCGCTCTTCAATGCCGGAGTTCTCAATGCAAGCAACATTGACACATTGGGATCCCG AGTTCTCTCAGCTGTTTTGAACGGAGTATCAAGTGCGGCGCAAGGTCTTGGCATCAATGTAGAT

>Aaur

GGTTACACAGGGCCTGCTGGACCTTCTGGCTACACAGGGCCTGCTGGACCTTCTGGTTACACTGGGCCTGTGGGTGGCGGAGCGCAGTT TGGTTCGGTTTCAGGTCAAACATCAGGCTTTACCGCATCTACTGGCGGACAAGCCGCTTTCGGTACAACAAGTGGAGCGTCTTCTGGCC TTATCTCCAGAGTAGCAAACGCACTTGCCAATACATCAACATTGAGAACTGTCCTCAGAAGAGGTGTATCCCAACAGATTGCCTCCAGC GTGGTACAGAGAGCCGCTCAGTCGTTGGCCAGTACTCTGGGAGTCGACGGAAATAACTTCTCCAGAATAGCATTACAAGCCATCTCTCA AGTGCCCGCAGGGTCTGACACTTCTGCTTACGCTCAAGCATTCTCTAGTGCGCTGTTCAATGCCGGAGTTCTGAATGCAAGCAACATTG ACTCATTGGGATCCCGAGTTCTGTCAGCAGTTTTGAACGGAGTATCAAGTGCGGCGCAAGGTGTTGGCATCAATGTAGAT

>Atri

GGATACACAGGACCTTCTGGACCTTCC--------------- ACTGGACCTTCTGGCTACCCTGGGCCTTTGGGTGGCGGAGCGCCGTTCGGT------------ CAAACATCAGGCTTTGGCGGATCTGTTGGCCCTCAAGGTGGATTCGGTGCCACAGGTGGAGCGTCTGCTGGCCTTATCTCCAGAGTAGC AAACGCACTTGCCAATACATCAACATTGAGAACTGTCCTCAGAAGAGGTGTATCTCAACAGATTGCCTCCAGCGTGGTACAGAGAGCCG CTCAGTCGTTGGCCAGTACTCTCGGAGTCGACGGAAATAACTTGGCCAGAATAGCATTACAAGCCATCTCTCAAGTGCCCTCAGGGTCT GACACTTCTGCTTACGCTCAGGCATTCTCTAGTGCGCTGTTCAATGCCGGAGTTCTGAATGCAAGCAACATTGACTCATTGGGATCCCG AGTTCTGTCAGCAGTTTTGAACGGAGTATCAAGTGCGGCGCAAGGTCTTGGCATCAATGTAGAT

>Av

GGTTTCACAGGGGCTGGTGGCTACCCTGGT------ GGAGCTGGTCCTTTGGGTGGCGGAGTAGGCTCTTTGACAGGCCAAACCTCTTTCGGT------------ CAAACATCAGGCTTTACTTCAACTGCTGGYGCCCAAGGAGGTTTCGGTCCAACAACTGGCGCGCAATCTGCCCTTATCTCCAGAATAGC TAACGCACTGGCGAATACATCAACACTGAGATCGGTCCTCAGAACCGGTGTATCCCAACAGACTGCCTCTAGCGTGGTACAGAGAACCA TCCAGACCTTGGCTAGTAATCTCGGCATCGACGGAAATAACTTGTCCAGAATAGCGTTACAAGCCATCTCTCAAGTCCCCGCGGGTTCT GACACTTCTGCTTACGCTCAAGCATTTTCTACTGCCTTGGTCACCGGTGGAGTTCTGAACGCAAACAACGTTGACACATTGGGATCCCA AGTACTCTCAGCAGTTTTGAACGGAGTATCAAGTGCGGCGCAAGGCCTTGGAATCAATGTAGAC

**Supplementary Figure S6. Nucleotide alignment of *AcSp1* repeat region from PCR survey of individual *Argiope* genomes.** Nucleotide

alignment in FASTA format. Alignment available on TreeBASE ([http://purl.org/phylo/treebase/phylows/study/TB2:S15355).](http://purl.org/phylo/treebase/phylows/study/TB2%3AS15355))

Abbreviations: *A. argentata* (Aarg), *A. aurantia* (Aau), *A. trifasciata* (At), and *Araneus ventricosus* (Av).
